# Introducing SuFNucs: Sulfamoyl-Fluoride-Functionalized Nucleosides That Undergo Sulfur Fluoride Exchange Reaction

**DOI:** 10.1021/acs.orglett.2c02034

**Published:** 2022-06-30

**Authors:** Mikołaj Chrominski, Kamil Ziemkiewicz, Joanna Kowalska, Jacek Jemielity

**Affiliations:** † Centre of New Technologies University of Warsaw, Banacha 2c, 02-097 Warsaw, Poland; ‡ Division of Biophysics, Institute of Experimental Physics, Faculty of Physics, University of Warsaw, Pasteura 5, 02-093 Warsaw, Poland

## Abstract

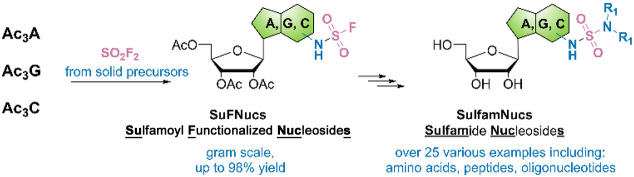

The reaction between ribonucleosides and ex situ generated sulfonyl fluoride has been developed. The reaction takes place at the −NH_2_ groups of nucleobases, and the resulting nucleosides are equipped with a sulfamoyl fluoride moiety, dubbed SuFNucs. These species undergo a selective sulfur fluoride exchange (SuFEx) reaction with various amines, leading to sulfamide-functionalized derivatives of adenosine, guanosine, and cytidine (SulfamNucs). The scope and examples of further SuFNucs fuctionalization leading to nucleotides, oligonucleotides, and peptide–nucleoside conjugates are presented.

Unlike other sulfur(VI) electrophiles, the −SO_2_F group is an extraordinarily stable unit, even under harsh conditions. Nevertheless, it is possible to controllably activate the S^VI^–F bond, transforming fluoride into a good leaving group and enabling reactions with various nucleophiles. Different conditions have been explored to achieve this purpose (appropriate acid/base environment, presence of R_3_Si^+^ as a mediator, properly selected catalyst, and constrained environment) and used in versatile applications. S^VI^–F-containing compounds and their chemical behavior have been known and utilized for nearly a century, but their huge potential was recognized and systematized in 2014 by Sharpless and co-workers under the term sulfur(VI) fluoride exchange reaction (SuFEx, Figure 1A).^1^ SuFEx reactions were immediately included in the click chemistry paradigm,^2^ as the S^VI^–F unit in organic compounds can be used as a molecular connector in a predictable manner under mild conditions. Since this breakthrough, the expansion of SuFEx-type reactions covered general organic chemistry,^3,4^ material/polymer science,^5^ chemical biology,^6^ bioconjugate chemistry,^7^ and medicinal chemistry including drug discovery.^8,9^ The SuFEx approach requires installation of a −SO_2_F group in the molecule of interest. The emerging demand for diverse molecules containing the S^VI^–F motif can be satisfied thanks to so-called SuFEx hubs–reagents that can introduce the SO_2_F group into the target molecule (Figure 1B). The simplest SuFEx hub is sulfonyl fluoride (SO_2_F_2_, bp = −55 °C),^3^ which, in spite of its high reactivity toward phenols and secondary amines, has limited application toward the functionalization of primary amines. Moreover, SO_2_F_2_ cannot be used in laboratories lacking proper equipment to handle harmful gases, and its availability is limited by regulations. These drawbacks have been solved by the development of a solid SO_2_F^+^ donor fluorosulfuryl imidazolium triflate salt (SuFExIT, Figure 1B)^10^ and [4-(acetylamino)phenyl]imidodisulfuryl difluoride (AISF, Figure 1B)^11^ which are stable, are easy to handle, ensure precise control over the reagent ratio, and show expanded reactivity (compared to the parent gas). Alternatively, SO_2_F_2_ can be generated ex situ from the solid precursors^10,12,13^ [for example sulfonyl diimidazole (SDI) and KF, Figure 1C] and delivered into the reaction mixture using specially designed, commercially available laboratory glassware (COware, Figure 1D). This set of reagents and fine-tuned conditions for their reaction with various organic structures allows quick access to the vast spectrum of SuFExable compounds for further investigations including drugs^9^ and other biologically active molecules.^14^

**Figure 1:**
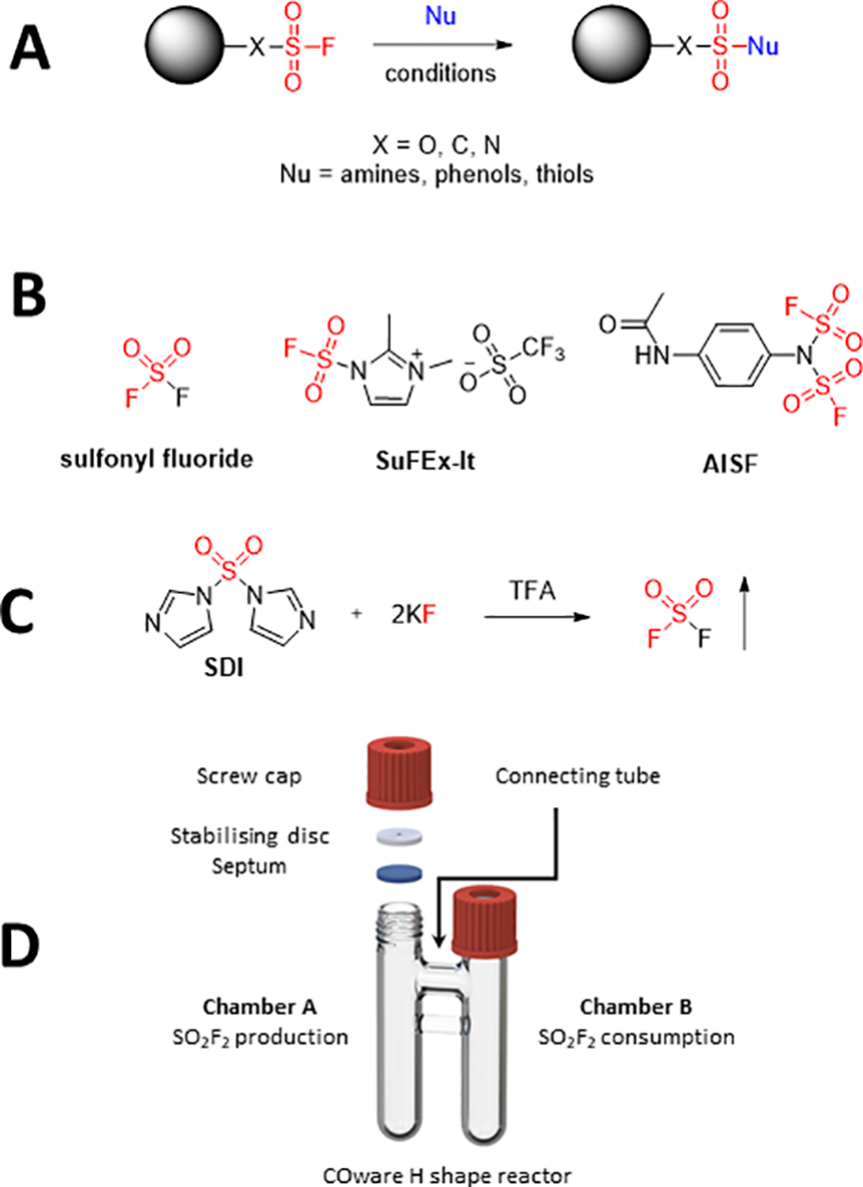
(A) General scheme of the sulfur(VI) fluoride exchange (SuFEx) reaction, (B) examples of SO_2_F^+^ donors (SuFEx hubs), (C) generation of SO_2_F_2_ from solid and liquid precursors, and (D) COware reactor used in this study for ex situ SO_2_F_2_ generation.

Although SuFEx reactions already established their usefulness in bioconjugate chemistry and medicinal chemistry, there is only a limited number of nucleosides and nucleotide derivatives functionalized with SuFexable units or involved in SuFEx-type chemistry.^15^ This gap in the area of SuFEx chemistry inspired us to investigate the possibility of introducing the −SO_2_F motif into nucleosides and investigate the potential of such derivatives as novel, ready-to-functionalize building blocks.

We aimed to add SuFEx-able groups to natural ribonucleosides bearing an −NH_2_ group at the nucleobase unit, namely, adenosine (A), guanosine (G), and cytidine (C). We expected that their successful reaction with an electrophilic SO_2_F^+^ donor will result in sulfamoyl-fluoride-functionalized nucleosides, which can be excellent starting materials for the synthesis of yet unexplored sulfamide-bearing nucleosides. To the best of our knowledge, the only example of nucleobases possessing a sulfamide group was synthesized using pentachlorophenyl sulfamate and was limited to simple −SO_2_–NH_2_ functionality.^16^ In preliminary experiments, we have tested SuFExIT^10^ as a SO_2_F group donor. This reagent has been shown as a superior SuFEx hub for primary amine derivatization, and before its introduction synthetic access to the sulfamoyl fluorides was limited.^17^ Unfortunately, by treating A, G, C, Ac_3_A, Ac_3_G, and Ac_3_C with this reagent in various solvents, we obtained complex reaction mixtures that were difficult to separate into individual components. Neither cooling the reaction mixture nor portionwise addition of the reagents changed this outcome. After this initial failure, we turned our attention into ex situ generation of SO_2_F_2_.^12^ This approach uses a two chamber H-shaped reactor designed by the Skrydstrup group (Figure 1D)^18^ for safe generation of precise amounts of gaseous reagents including CO, HCl, HCN, H_2_, and SO_2_^19^ from solid and liquid substrates. Using this technique, SO_2_F_2_ can be generated in a one-reactor chamber by treating a mixture of SDI and KF with TFA upon vigorous mixing (Figure 1C).^12^ The generated SO_2_F_2_ diffuses under pressure into the second reactor chamber containing the solution of the substrate via a connecting tube and enters the SuFEx reaction. It was found to be very efficient in the preparation of a wide variety of fluorosulfates from phenols (when triethylamine/DCM was used), while amine groups remained unreacted.^12^ Keeping that in mind, we decided to manipulate the reported conditions to check if the proper selection of the base/solvent system and control over SO_2_F_2_ administration would allow us to perform amine to sulfamoyl fluoride transformation. For practical reasons (solubility and TLC-based monitoring of the reaction progress), we started to test this strategy using Ac_3_A as a model substrate and DCM as a solvent using 3 equiv of SO_2_F_2_ precursors (Scheme 1).

**Scheme 1:**
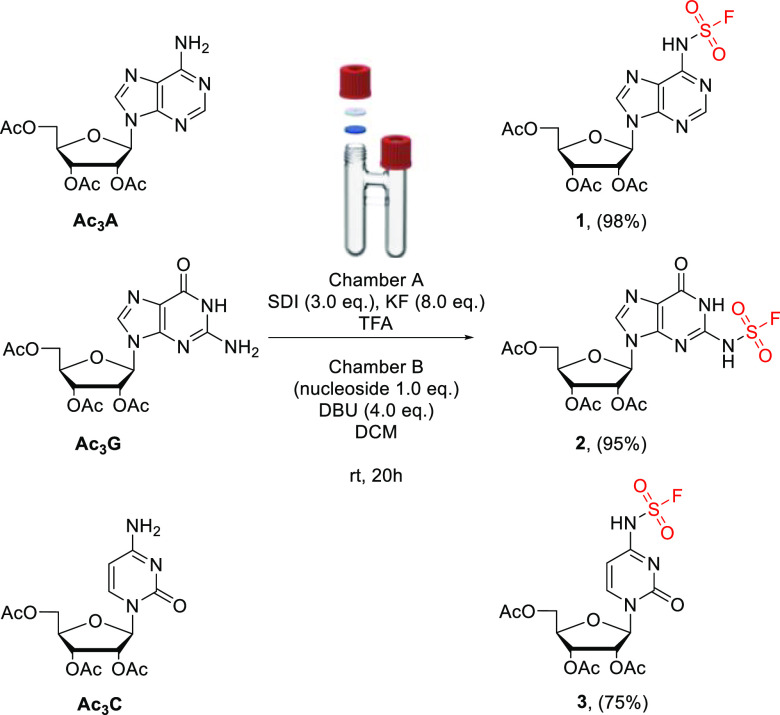
Synthesis of Sulfamoyl-Functionalized Nucleosides (SuFNucs) **1**–**3** via ex situ generated SO_2_F_2_

When no base was present in the reaction mixture or weak base was used, the starting material was fully recovered, but in the presence of DBU (4 equiv) the overnight reaction resulted in full conversion of the starting material and highly selective formation of the expected product. The reaction proceeded equally well in any solvent tested (see Table S1), and the product **1** was isolated in nearly quantitative yields and excellent purity only by extraction techniques (to remove DBU and its salts). In the case of Ac_3_G and Ac_3_C, the same reaction conditions and purification method worked alike, giving **2** and **3** in 95% and 75% yield, respectively (Scheme 1, see SI for details).

The structures of the products were confirmed by a set of NMR analyses, which indicated the presence of a fluorine nucleus with chemical shifts in the range characteristic for sulfamoyl fluorides: δ_F_ = 53.93 ppm for **1** (recorded in CDCl_3_), δ_F_ = 50.89 ppm for **2** (recorded in DMSO-*d*_6_), and δ_F_ = 52.49 ppm for **3** (recorded in CDCl_3_). The ^13^C NMR analysis of **2** revealed a spin–spin coupling between C2 carbon and fluorine (^3^*J*_C–F_ = 3 Hz), confirming that the reaction took place at the N2 position.

The reaction scale up did not generate any losses in selectivity and maintained high yields and purity, allowing for the preparation of larger quantities (up to 3 mmol/>1 g in one batch when 100 mL of reactor was applied, see Figure S1) of SuFNucs **1**–**3** without compromising the reaction performance.

After establishing a practical method for the preparation of SuFNucs **1**–**3**, we investigated their reactivity toward amines. Heating **1** with selected amines (benzyl amine, morpholine, and toluidine) in the presence of TEA in various solvents^10^ did not lead to the formation of the desired product, while slight degradation of the staring material was observed. Hence, we focused on the approach toward reacting “SuFExable” units with amines developed by am Ende and Ball.^20,21^ In this concept, the activation of the −SO_2_F group is achieved by the addition of a stoichiometric amount of Lewis acid in combination with a proper amine mediator. Application of Ca(NTf_2_)_2_ and DABCO has allowed us to transform sulfamoyl fluorides, fluorosulfates, and sulfonyl fluorides into respective sulfamides, sulfamates, and sulfonamides by applying a single set of optimized conditions.^21^ To our delight, the model reaction between **1** and benzyl amine in the presence of the Ca(NTf_2_)_2_/DABCO system resulted in the formation of the expected sulfamide along with some unidentified polar byproducts, which could be easily removed during workup (see Table S2 and Figure S2 for details).

The yield and selectivity were affected by the nature of the solvent used (Table S2 and Figure S2), with DCM and MeCN performing the best. This finding opened an entry to a wide range of different sulfamide-modified nucleosides (SulfamNucs, Figure 2). The reaction worked well for ammonia (**4a**, 30%) and simple primary amines like methyl amine (**4b**, 41%), *t*-Bu amine (**4c**, 55%), cyclopropyl amine (**4d**, 44%), benzyl amine (**4f**, 50%), and adamantine (**4e**, 45%). The utility of aniline derivatives was demonstrated by 4-ethynylaniline (**4g**, 76%). Amines possessing an additional functional group, which can be used for further transformations, gave high yields of valuable compounds **4h** (69%) and **4i** (70%) functionalized with terminal alkyne and azide, respectively. The yields were not affected upon scaleup to 2 mmol of the starting material. A conjugate with an amino acid derivative, methyl ester of glycine, was also obtained in good yield (**4j**, 46%). Secondary amines reacted equally well, giving pyrrolidine (**4k**), morpholine (**4l**), and proline methyl ester (**4m**) SulfamNucs derivatives in 70%, 64%, and 43% yield, respectively. It is worth mentioning that some of the amines (volatile ones and amino acids) were used as hydrochlorides, which did not affect the yields if a proper excess of DABCO was applied. The functionalization of guanosine derivative **2** also gave satisfying results, after a slight amendment of the reaction conditions was applied to overcome solubility issues (use of the DCM/THF mixture as a solvent and lowering the concentration). Yields of the respective SulfamNucs were slightly lower compared to adenosine derivatives; nevertheless, the simplicity of workup and purification allowed us to obtain a collection of SulfamNucs **5a**–**g** (Figure 2) with simple and more complex substituents (for additional information, see Figure S3). In the case of cytidine, the reactivity under the established conditions was similar to adenosine. Compound **3** reacted smoothly with different primary and secondary amines (Scheme 2) in moderate to good yields, keeping the workup/isolation of SulfamNucs **6a**–**e** simple and not particularly laborious.

**Figure 2:**
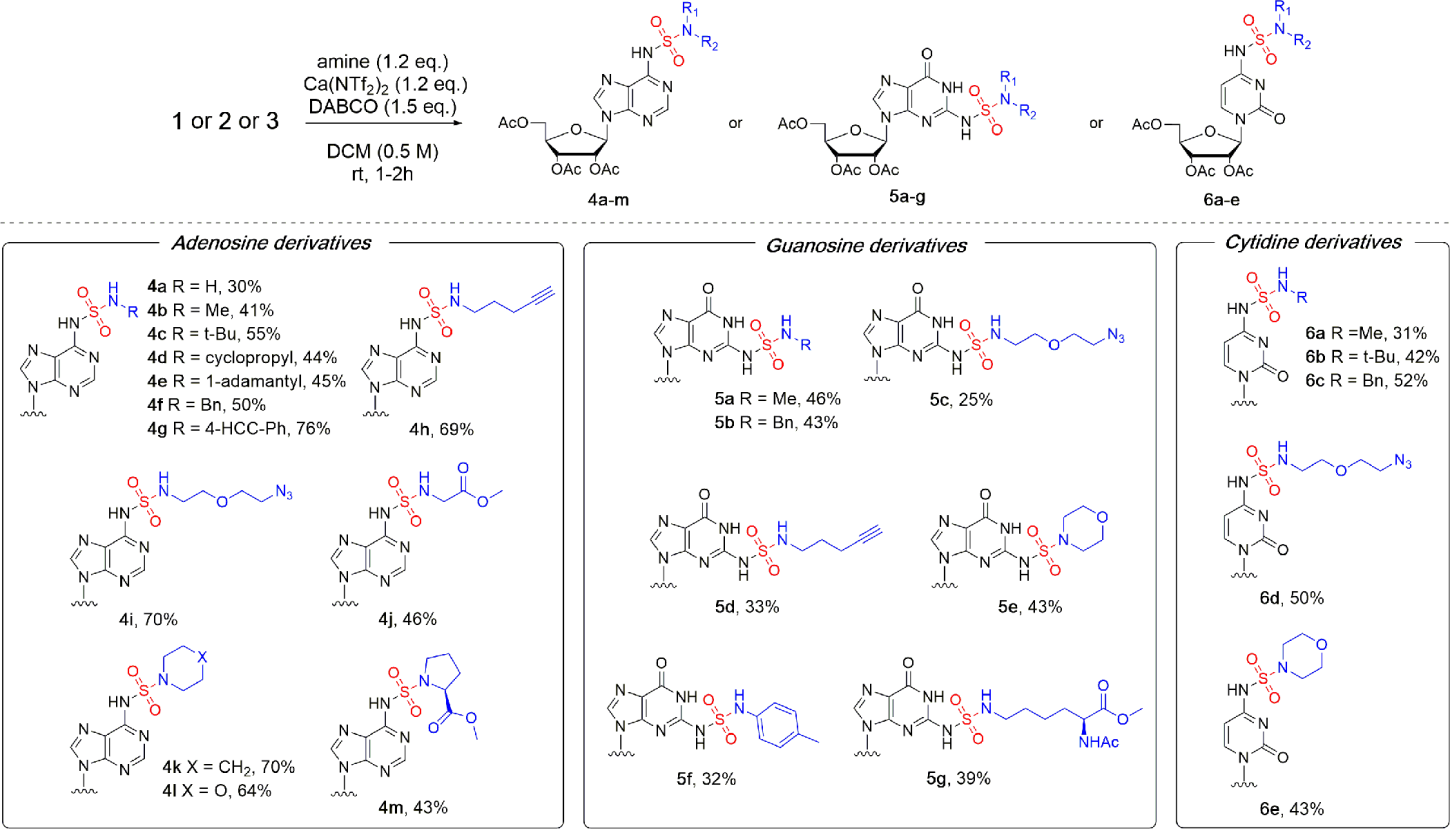
Scope of Ca(NTf_2_)_2_/DABCO-mediated SuFEx reaction between SuFNucs and amines.

**Scheme 2:**
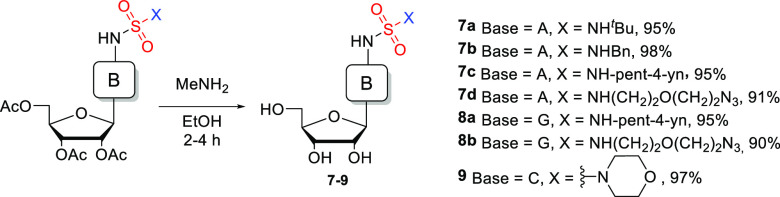
SulfamNucs **7**–**9** Obtained upon Deprotection of Acetylated SulfamNucs **4**–**6**

The deprotection of SulfamNucs was tested with a range of standard reagents (ammonia, MeNH_2_ in MeOH, and MeNH_2_ in EtOH). Methylamine in absolute ethanol (33%) was the most effective, leading to unprotected nucleosides in high yields and only minimal workup necessary (Scheme 2; see SI for details).

To demonstrate the reactivity of SuFNucs toward more complex molecules, we reacted **1** with a short peptide-containing lysine (Scheme 3). Due to the solubility issues, the reaction was performed in DMSO, under high dilution and with an excess of **1** relative to the peptide. After 4 h of incubation, the whole peptide was consumed, and the desired conjugate was easily isolated using RP-HPLC. Deprotection of OH groups was performed as described above and lead selectively to the peptide–nucleotide conjugate **10** connected via sulfamide linkage in 24% overall yield (the conditions and workup/isolation procedure were not optimized).

**Scheme 3:**
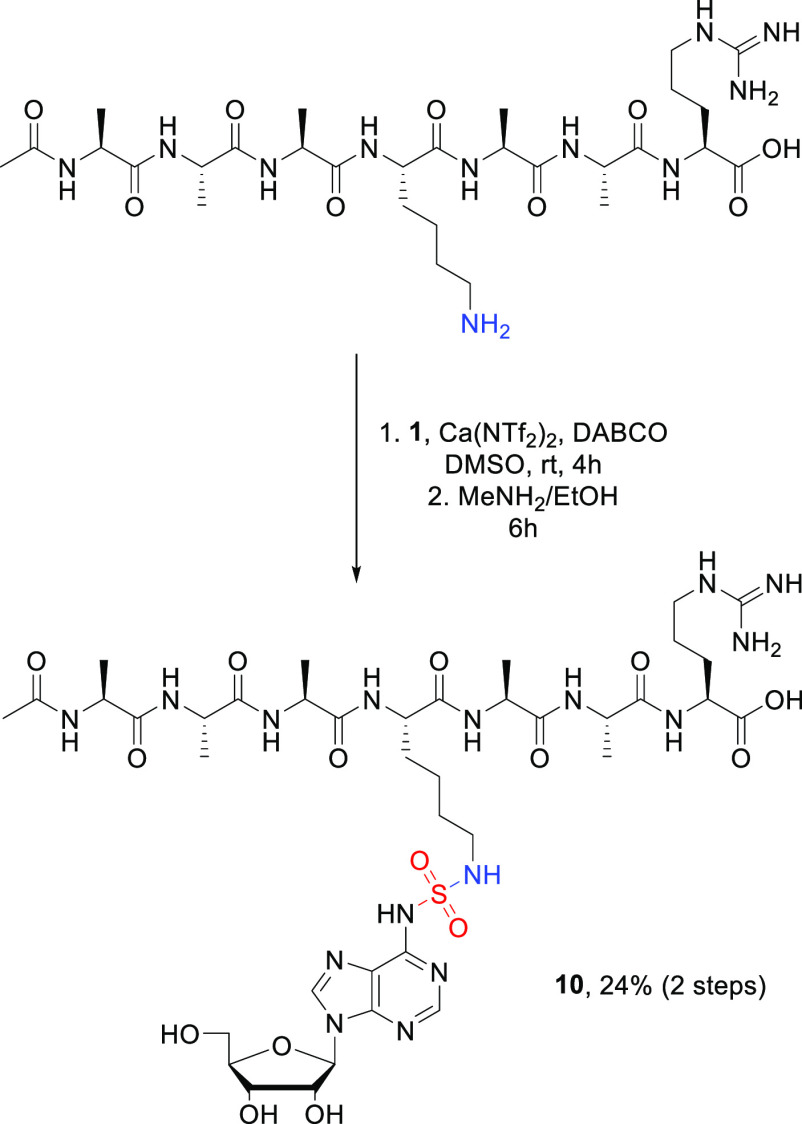
Conjugation of SuFNuc **1** with Peptide

To investigate further transformation possibilities, adenosine SulfamNuc **7d** was transformed into the corresponding 5′-phosphate under standard Yoshikawa phosphorylation conditions (Scheme 4).^22^ Treatment of **7d** with POCl_3_ in (MeO)_3_PO under cooling resulted in monophosphate **11** in good yield and purity.

**Scheme 4:**
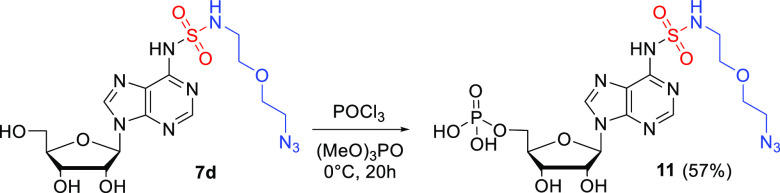
Phosphorylation of SulfamNuc **7d**

Finally, we introduced sulfamide-functionalized adenosine into short RNA oligonucleotide using standard phosphoramidite-based solid phase synthesis (SPS). The synthesis of the proper phosphoramidite building block required for SPS started from 2′*-O*-Me adenosine (2′-*O*-MeA; Scheme 5). This excluded the necessity of selective 2′-OH protection and simplified the synthesis. The synthesis of proper SulfamNuc **14** was accomplished in 3 steps with 72% yield. Introduction of DMT at the 5′ position and phosphoramidite at the 3′ position was achieved using standard nucleoside chemistry and led to **16** in 36% yield (26% overall yield for 5 steps). **16** was used in manual SPS (see SI for details) of trinucleotide **17** composed of uridine, SulfamNuc 2′*-O*-Me adenosine **14**, and guanosine. Upon deprotection and cleavage from the support, the trinucleotide **17** was purified using standard chromatographic techniques in 57% yield.

**Scheme 5:**
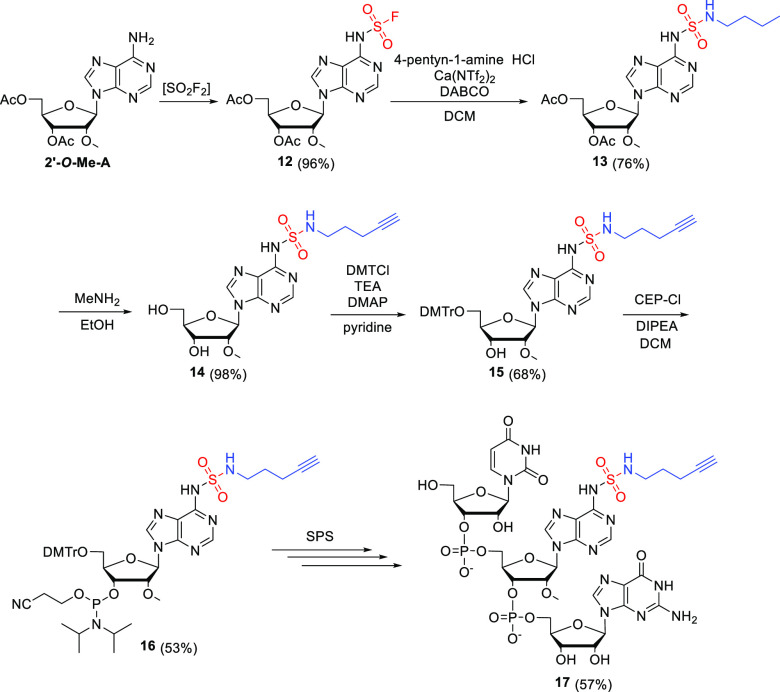
Synthesis of Trinucleotide **17** Containing Sulfamide-Functionalized 2′-*O*-Me Adenosine as an Internal Nucleotide

In summary, by applying ex situ generation of SO_2_F_2_, we were able to prepare adenosine, guanosine, and cytidine derivatives bearing −NHSO_2_F motif as “SuFEx-able” nucleosides (SuFNuc) in high yields. Such a reaction between primary amine and SO_2_F_2_ is very rare and is in contrast to previous observations.^1,12^ The −NHSO_2_F functional group was then used to functionalize these nucleosides with diverse amines leading to previously unknown sulfamide-functionalized nucleosides (SulfamNuc) in good yields. All these transformations are operationally simple, are high yielding, and can be easily upscaled to the gram scale, and obtained compounds are stable upon storage. Further prospects of SulfamNuc chemistry were demonstrated by conjugation with peptide and incorporation into nucleotides including oligonucleotides. These findings potentially open several new avenues in nucleoside and nucleotide chemistry, including more robust preparation of highly functionalized structures, bioconjugates, and nucleotide/nucleoside libraries. Further exploration of SuFNuc- and SulfamNuc-type compounds is under investigation in our group and will be presented in due course.

## Supporting Information Available

Supporting tables and figures, detailed synthetic procedures, and compound characterization (PDF)
