# Supplementary material for: Introducing SuFNucs: Sulfamoyl-Fluoride-Functionalized Nucleosides That Undergo Sulfur Fluoride Exchange Reaction
Source: Org Lett. 2022 Jun 30;24(27):4977–81. doi: 10.1021/acs.orglett.2c02034 (PMC9295159; doi:10.1021/acs.orglett.2c02034)

`

Supporting information
for

Introducing SuFNucs - sulfamoyl fluoride functionalised nucleosides that undergo SuFEx reaction

Mikołaj Chrominski*,[1], Kamil Ziemkiewicz[1], Joanna Kowalska[2], Jacek Jemielity*,[1]

[1]Centre of New Technologies University of Warsaw, Banacha 2c, 02-097 Warsaw, Poland

[2]Division of Biophysics, Institute of Experimental Physics, Faculty of Physics, University of Warsaw, Pasteura 5, 02-093 Warsaw, Poland

Corresponding Authors
Jacek Jemielity: j.jemielity@cent.uw.edu.pl
Mikołaj Chromiński: m.chrominski@cent.uw.edu.pl

Table of content

Supporting figures 2

Synthetic procedures 7

- General information 7
- Starting materials 9
- General procedure 1 gram scale synthesis SuFNucs **1**, **2** and **3.** 10
- General procedure 2 SuFEx reaction of SuFNucs **1**-**3** with amines 13
- General procedure 3 deprotection of SulfamNucs **4**-**6** 26
- The synthesis of conjugate **10** 30
- The synthesis of nucleotide **11** 33
- The synthesis of **16** 34
- The synthesis of oligonucleotide **17** 38

Copies of NMR and HRMS spectra 40

Supporting figures

Table S1. The synthesis of sulfamoyl functionalised adenosine via ex situ generated SO_2F_2 - optimisation studies. Conditions: The reaction was performed in a two-chamber H shape reactor (COware gas reactor, Sigma-Aldrich, total volume 20 ml, cat.no. STW1-1EA) equipped with single use silicone/PTFE septa (Sigma-Aldrich, cat.no. STW3) according to the report by Borggraeve et. al. (Org.Lett.2017, 19, 5244–5247). The paper contains highly detailed description (including video clips) of the usage of such an experimental setup.Both chambers were equipped with the stirring bar and next the chamber A was charged with KF (232 mg, 4.0 mmol) and SDI (297 mg, 1.5 mmol) and the chamber B with **Ac_3A** (196 mg, 0.5 mmol). To the chamber B containing **Ac_3A** solvent was added followed by addition of DBU (300 ul, 2 mmol) and the stirring was turned on. The reactor was tightly sealed with septa and screw caps. When the clear solution was formed, the formation of SO_2F_2 was triggered by careful addition of TFA (1.5ml) to the chamber A containing KF/SDI mixture upon vigorous stirring. After overnight reaction the chamber B was carefully opened and left upon stirring for approx. 10 min under the fumehood to evacuate excess SO_2F_2. The reaction mixture was then transferred to a separatory funnel, diluted with DCM (20 ml) and washed with 0.1 M HCl (2 x 20 ml) and brine (30 ml). The organic phase was dried over Na_2SO_2, filtered off and concentrated *in vacuo*. To the resulting oily residue Et_2O was added (approx. 15 ml) and concentrated under reduced pressure followed by drying under high vacuum (the sticky solid turned into foam).

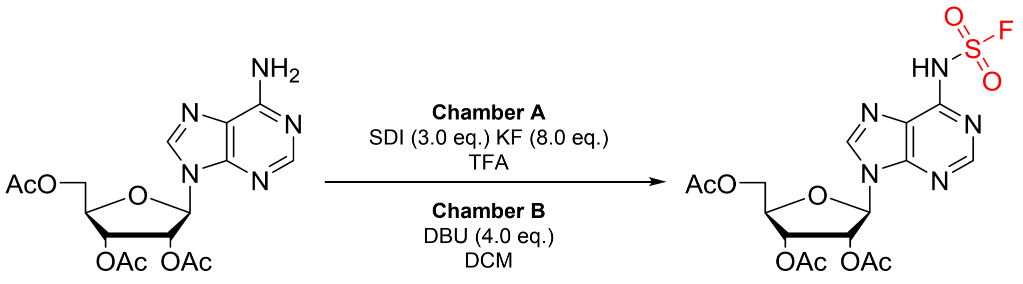

Ac_3A rt, 20h **1**

Entry	Solvent	Isolated yield of **1**
1	DCM	98%
2	DCM[a]	85%[c]
3	DCM[b]	0%
4	MeCN	98%
5	AcOEt	96%
6	Acetone	72%[c]
7	DMF	90%
8	DMSO	92%
9	THF	98%

[a]Reaction performed starting from 1 mmol of **Ac_3A** with the amount of other reagents unchanged; [b]TEA instead of DBU was used; [c]Conversion not complete, yield after column chromatography (silica gel, 5% MeOH in DCM)

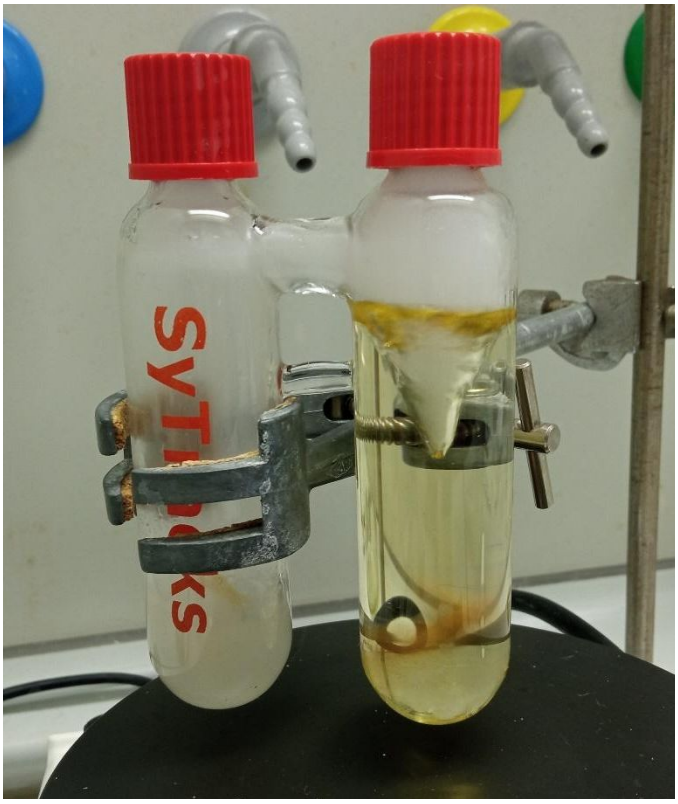

Figure S2. The synthesis of SuFNUcs via ex situ generated SO_2F_2 -the reaction performance a few seconds after addition of TFA to the chamber A (on the left). SO_2F_2 is formed and diffuses into chamber B (on the right) which is manifested as white fume that is absorbed into reaction mixture.

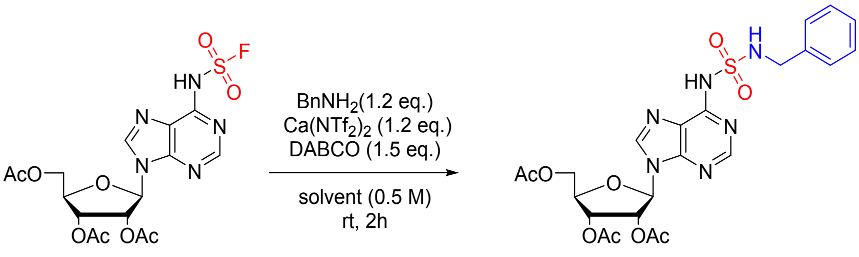

1, (24 mg, 0.05 mmol), $Ca(NTf_2)_2$ (36 mg, 0.06 mmol) and DABCO (9 mg, 0.08 mmol) were dissolved in indicated solvent (0.1 ml). To this mixture benzyl amine (6.7 ul, 0.06 mmol) was added and the reaction mixture was stirred at room temperature until TLC analysis (5% MeOH in DCM) indicated consumption of the starting material (approx. 2h). The reaction mixture was diluted with DCM, washed twice with 0.2 M HCl and washed once with brine. The organic phase was dried over Na_2SO_4, filtered and concentrated under reduced pressure. The product was purified using column chromatography (SiO_2, 0 to 5% MeOH in DCM). Combined fractions containing pure product were concentrated under reduced pressure followed by drying under high vacuum.

Reaction	Solvent	Isolated yield
A	MeCN	45%
B	DCM	50%
C	DMF	28%[a,b]
D	DMSO	12%[a,b]
E	MeOH	17%[a,b]

[a]Unidentified byproducts formed; [b]Conversion not full

Figure S3. The reaction between **1** and benzyl amine – optimisation studies.

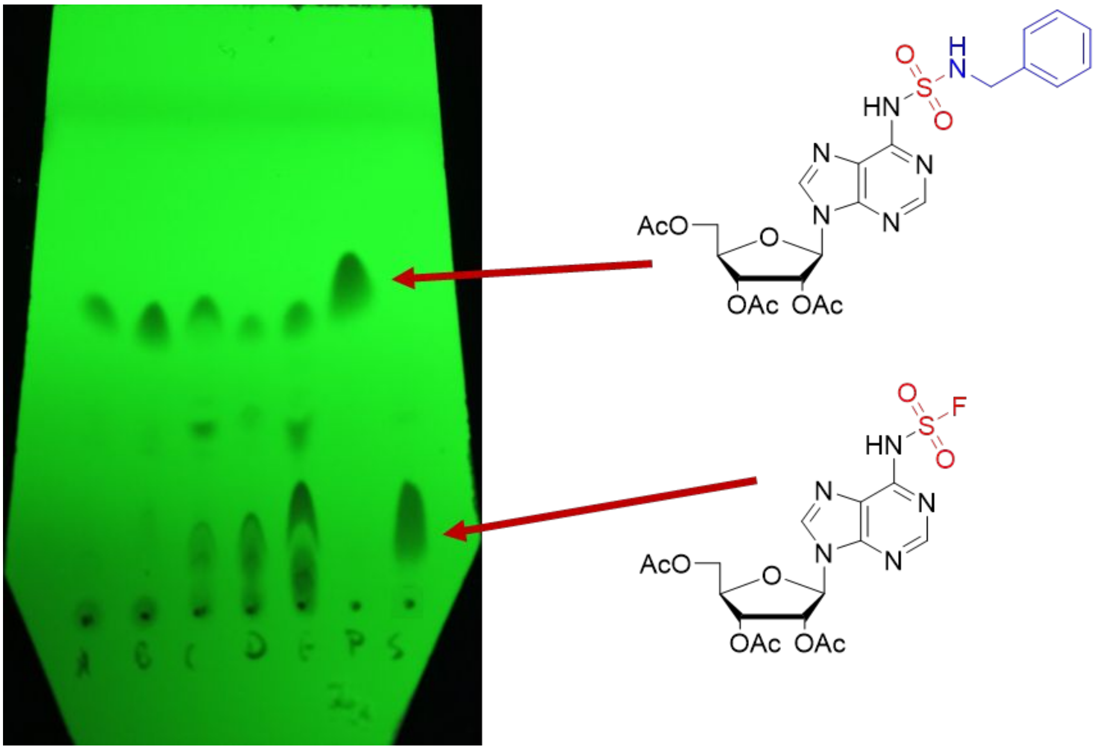

Figure S4. TLC analysis of the reaction mixtures performed in 5% MeOH in DCM and visualised under UV (254 nm) irradiation. Traces A-E correspond to the solvents indicated in the table on Figure S3, traces P and S correspond to the SulfamNuct **4f** and SuFNuc **1** respectively. Samples were prepared by diluting 0.5 µl of the reaction mixture with DCM (200 µl), washing it twice with 0.5 M HCl (approx. 200 µl) and drying over anhydrous Na_2SO_4.

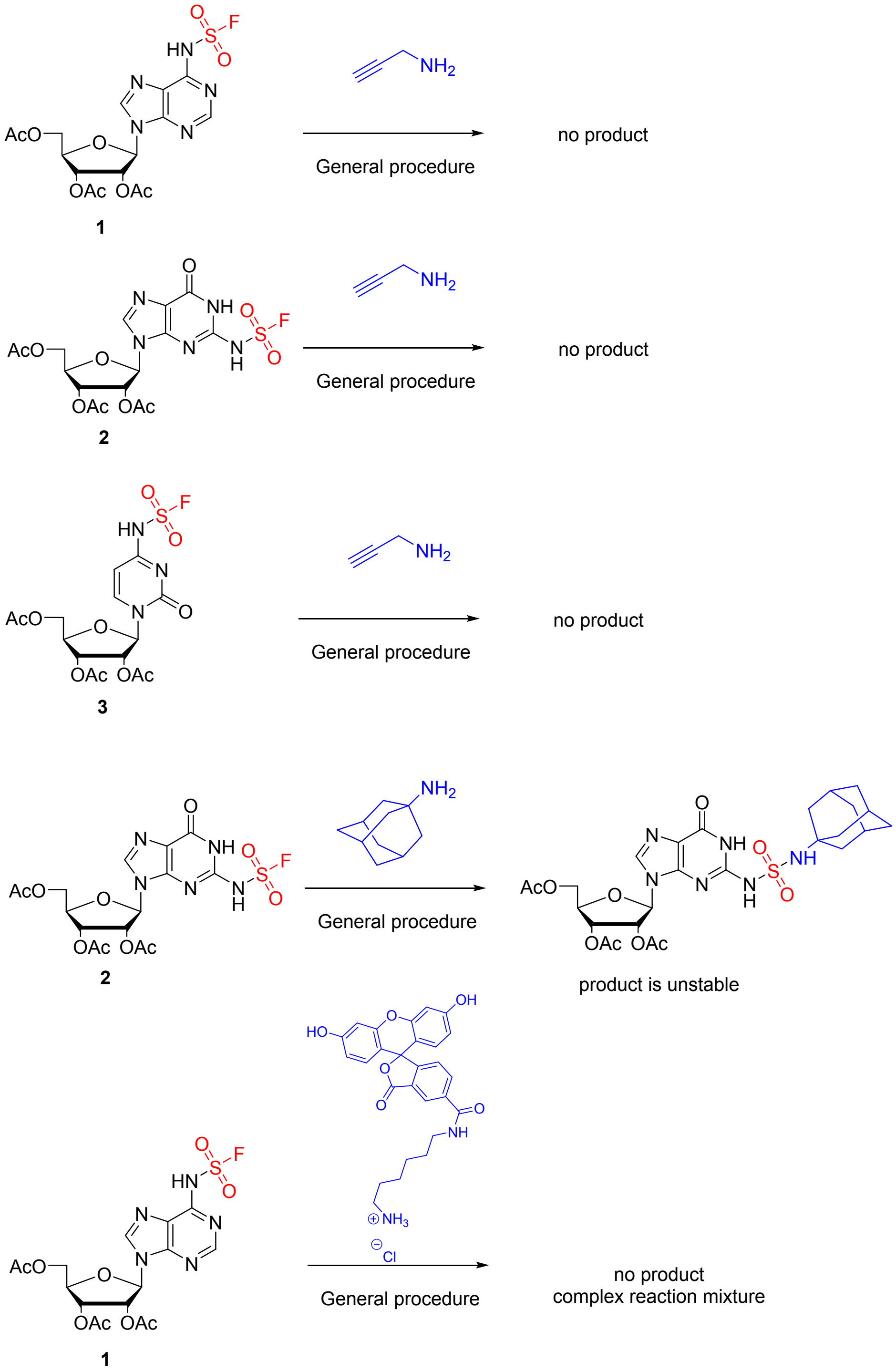

Note: Although during presented research reactions with propargyl amine were unsuccessful, it was recently reported that under similar SuFEx conditions (calcium/DABCO system) it reacts with alkenyl fluorosulfonates leading to alkenyl sulfamates (Michaudel et al, Org. Lett. 2021, 23, 13, 5271–5276)

Figure S5. Unsuccessful reaction between SuFNucs **1-3** and amines.

Synthetic procedures

General information

All commercial reagents were used as received without additional purification except for $POCl_3$, which was distilled prior reaction.

Guanosine, adenosine and 2’-*O*-Me adenosine were purchased from Carbosyntyh, cytosine was purchased from Merck, SDI, KF and TFA were purchased from Fluorochem, Calcium(II) bis(trifluoromethanesulfonimide) and DABCO were purchased from TCI.

Peptide used for the synthesis of conjugate **10** was ordered form Merck.

Sources of reagents for solid phase synthesis are indicated in the procedure.

Analytical and preparative chromatography

Thin Layer Chromatography (TLC) analysis was carried out on pre-coated Silica Gel 60Å on aluminum foil with fluorescence indicator (Merck) and visualised under UV lamp (254 nm).

Preparative chromatography was performed using Silica Gel 60 (0.043-0.063 mm, 230-400 mesh) purchased form Merck.

Ion-Exchange Chromatography

Synthesized nucleotides and oligonucleotides were purified by ion-exchange chromatography on a DEAE Sephadex A-25 (HCO^{3-} form) column. After loading the column with the reaction mixture and washing it with water, the products were eluted using different linear gradients of triethylammonium bicarbonate (TEAB) in deionized water. Fractions containing the desired product were collected together after reversed-phase (RP) HPLC and spectrophotometric (at 260 nm) analysis. Evaporation under reduced pressure with repeated additions of 96% and then 99.8% ethanol resulted in isolation of nucleotides as triethylammonium salts.

Analytical and preparative HPLC

Analytical HPLC was performed on Agilent Tech. Series 1200 using Gemini 3 µm NX-C18 LC column 110 Å (150×4.6 mm, flow rate 1 mL min^{-1}) and UV-detection at 254 nm.

Analytical HPLC programs

Program A

Buffer A: 0.05% TFA in water

Buffer B: 0.05% TFA in acetonitrile

Time [min]	[%] buffer B
0	0
15	50

Program B

Buffer A: 0.05 M ammonium acetate, pH = 5.9

Buffer B: methanol/buffer A, 50/50 v/v

Time [min]	[%] buffer B
0	0
7.5	100
15	100

Semi-preparative HPLC was performed on the same apparatus equipped with RP HPLC using a Gemini® NX-C18 LC 5 µm column 110 Å (150 x 10 mm, flow rate 5.0 mL/min).

Spectroscopic analysis of the synthesized compounds

The structure and purity of compounds were confirmed high resolution mass spectrometry using electrospray ionization (HRMS ESI) and ion trap analyser and NMR spectroscopy.

Mass spectra were recorded on Thermo Scientific LTQ OrbitrapVelos .

NMR spectra were recorded at 25 °C with a BRUKER AVANCE III HD spectrometer at 500 MHz (^{1}H NMR), 126 MHz (^{13}C NMR) 202 MHz (^{31}P NMR) and 471 MHz (^{19}F NMR).

The ^{1}H NMR and ^{13}C NMR chemical shifts were reported in ppm with residual solvent peak as internal standard.

The ^{31}P NMR chemical shifts were reported in ppm and referenced to 20% phosphoric acid in D_2O as an external standard.

The ^{19}FNMR chemical shifts were reported in ppm and referenced to $CFCl_3$ (for spectrarecorded in $CDCl_3$ and DMSO-d6 , 0.65 and −0.24 ppm, respectively) as an external standard.

NMR signals asignments for compounds **1-3**, **16** and **17** were performed according to 2D correlation spectra.

Some of the obtained products contained residual solvents (usually diethyl ether, dichloromethane or triethylamine). The presence of these solvents results from the workup and purification procedures. These residual solvents could not be removed even upon overnight drying under high vacuum. Their presence is indicated on the attached copies of NMR spectra and their content is taken into account and excluded in the reported final yields.

Starting materials

Ac$_3$A was prepared according to *Bioorg. Med. Chem Lett.*, 24(12), 2703–2706.

Ac$_3$G was prepared according to *JACS*, 127(51) 18133–18142.

Ac$_3$C was prepared in the sequence presented below (adapted from *J. Org. Chem.* 2005, 70, 18, 7455–7458).

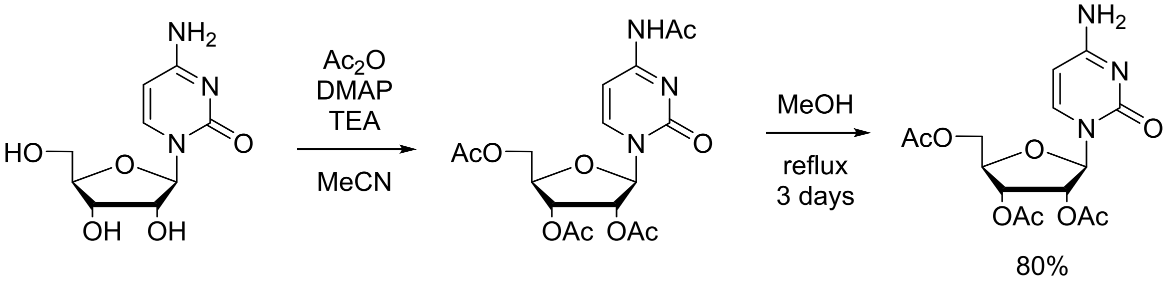

Briefly: **Ac$_4$C** was refluxed in MeOH for 3 days until TLC analysis (2% MeOH in DCM) indicated nearly full consumption of the starting material and traces of compounds with free OH groups. The solvent was evaporated to dryness and the product was isolated using column chromatography (0 to 2% MeOH in DCM). **Ac$_3$C** was obtained as a white solid in 80% yield.

^{1}H NMR (500 MHz, Chloroform-*d*) δ 7.45 (d, *J* = 7.5 Hz, 1H), 6.00 (d, *J* = 4.3 Hz, 1H), 5.89 (d, *J* = 7.5 Hz, 1H), 5.41 (dd, *J* = 5.7, 4.3 Hz, 1H), 5.36 (m 1H), 4.40 – 4.28 (m, 3H), 2.13 (s, 3H), 2.09 (s, 3H), 2.08 (s, 3H).

^{13}C NMR (126 MHz, Chloroform-*d*) δ 170.5, 169.9, 169.8, 165.9, 155.6, 141.2, 95.7, 89.4, 79.4, 73.6, 70.1, 63.2, 21.0, 20.7, 20.7.

LR-MS (ESI): m/z calcd. for M+H$^+$ [$C_{15}H_{20}N_3O_8$]$^+$ 370.1, found: 370.0, m/z calcd. for 2M+H$^+$ [$C_{30}H_{39}N_6O_{16}$]$^+$ 739,2, found: 739.1

2'-*O*-MeAc$_2$A was prepared according to *ChemBioChem*, **2020**, vol. 21, 1-2, 265 - 271.

General procedure 1 gram scale synthesis SuFNucs **1**, **2** and **3**.

The reactions were performed in a two-chamber H shape reactor (COware gas reactor, Sigma-Aldrich, total volume 100 ml, cat.no STW5-1EA) equipped with single use silicone/PTFE septa.

WARRNING: *Upon addition of TFA to the mixture of KF and SDI an instant reaction with the release of gases occurs. This causes a rapid increase of the pressure in the reactor which may cause sudden disconnection of the syringe and the needle or push the plunger out from the syringe barrel and TFA splash. The addition of TFA should be possibly quick and one should take highest possible safety measures during this procedure performance.*

Both chambers were equipped with the stirring bar[a] and next the chamber A was charged with KF (1.39 g, 24.0 mmol) and SDI (1.98 g, 9.0 mmol) and the chamber B with **Ac_3A**, **Ac3G** or **Ac3C** (3.0 mmol). To the chamber B DCM (30 ml) was added followed by addition of DBU (1.8 ml, 12 mmol) and the stirring was turned on. The reactor was tightly sealed with septa and screw caps. When the clear solution was formed, the formation of SO_2F_2 was triggered by careful addition of TFA (9.0 ml) to the chamber A containing KF/SDI mixture upon vigorous stirring.[b] After overnight reaction, chamber B with the reaction mixture was carefully opened[c] and left upon stirring for approx. 10 min under the fumehood to evacuate excess SO_2F_2. The reaction mixture was then transferred to the separatory funnel, diluted with DCM (to 100 ml) and washed with 0.5 M HCl (2 x 50 ml) and brine (50 ml). The organic phase was dried over Na_2SO_2, filtered off and concentrated *in vacuo*. To the resulting oily residue Et_2O was added (approx. 50 ml) and concentrated under reduced pressure followed by drying under high vacuum (the sticky solid turned into foam).[d]

a) Because of the height/width ratio of COware reactor, the stirring bar in chamber B should assure vigorous stirring with vortex formation (20 mm cylindrical stirring bar is recommended). One should adjust the stirring rate to form steady and deep vortex (approx. 800 rpm is recommended) and avoid the reaction mixture splashing into the chamber A (see Figure S2).
b) At this point a rapid and highly exothermic reaction starts in chamber A. SO_2F_2 is formed and white fumes can be observed inside the reactor (see Figure S2).
c) Caution: when opening the reactor a bubbling of the reaction mixture can occur.
d) The yields of the following calcium/DABCO mediated SuFEx procedures depend significantly on the substrate purity. From authors observation: if the nucleosides **1**, **2** or **3** obtained according to this general procedure contain substantial amounts of residual DBU the conversions of SuFEx reactions with amines are not full and yields of sulfamides are much lower. The content of DBU can be verified by ^1H NMR spectra analysis. If necessary, excess DBU can be removed by dissolving nucleoside in DCM and consecutive washing with 0.5 M HCl. From authors experience, column chromatography does not provide satisfying removal of residual DBU.

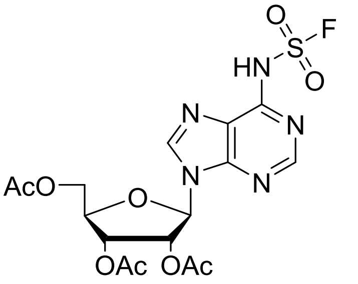

(2R,3R,4R,5R)-2-(acetoxymethyl)-5-(6-((fluorosulfonyl)amino)-9H-purin-9-yl)tetrahydrofuran-3,4-diyl diacetate (1):

Obtained starting from **Ac$_3$A** (1.18 g, 3 mmol), beige solid, yield: 1.39 g (98%).

^{1}H NMR (500 MHz, Chloroform-*d*) δ 8.62 (s, 1H, C2-H), 8.37 (s, 1H, C8-H), 6.24 (d, *J* = 5.5 Hz, 1H, C1'-H), 5.85 (t, *J* = 5.5 Hz, 1H, C2'-H), 5.55 (dd, *J* = 5.6, 4.3 Hz, 1H, C3'-H), 4.50 (m, 1H, C4'-H), 4.41 (d, *J* = 3.8 Hz, 2H, C5'-H), 2.17 (s, 3H, $^{acetyl}CH_3$), 2.15 (s, 3H, $^{acetyl}CH_3$), 2.09 (s, 3H, $^{acetyl}CH_3$).

^{13}C NMR (126 MHz, Chloroform-*d*) δ 170.5 (^{acetyl}C=O), 169.8 (^{acetyl}C=O), 169.5 (^{acetyl}C=O), 151.3 (C6), 150.4 (C4), 144.8 (C2), 142.3 (C8), 123.9 (C5), 86.9 (C1'), 81.0 (C4'), 73.4 (C2'), 70.7 (C3'), 63.1 (C5'), 20.9($^{acetyl}CH_3$), 20.7($^{acetyl}CH_3$), 20.5 ($^{acetyl}CH_3$).

^{19}F NMR (471 MHz, Chloroform-*d*) δ 53.9.

HR-MS (ESI): m/z calcd. for M-H$^+$ [$C_{16}H_{17}FN_5O_9S$]$^-$ 474,0736, found: 474.0735.

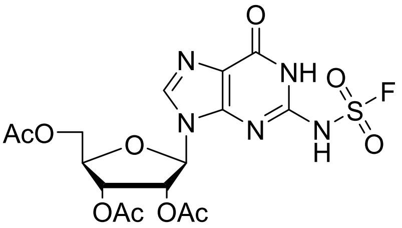

(2R,3R,4R,5R)-2-(acetoxymethyl)-5-(2-((fluorosulfonyl)amino)-6-oxo-1,6-dihydro-9H-purin-9-yl)tetrahydrofuran-3,4-diyl diacetate (2):

Obtained starting from **Ac$_3$G** (1.23 g, 3 mmol), white solid, yield 1.40 g (95%)

^{1}H NMR (500 MHz, DMSO-d_6) δ 11.00 (s, 1H, N1-H), 7.90 (s, 1H, C8-H), 6.01 (d, *J* = 4.8 Hz, 1H, C1'-H), 5.86 (dd, *J* = 6.2, 4.8 Hz, 1H, C2'-H), 5.61 (dd, *J* = 6.3, 4.9 Hz, 1H, C3'-H), 4.39 (m, 1H, 1 x C5'H), 4.27 (m, 2H, C4'-H and 1 x C5'-H), 2.10 (s, 3H, $^{acetyl}CH_3$), 2.05 (s, 3H, $^{acetyl}CH_3$), 2.01 (s, 3H, $^{acetyl}CH_3$).

^{13}C NMR (126 MHz, DMSO-d_6) δ 170.1 (^{acetyl}C=O), 169.3 (^{acetyl}C=O), 169.2 (^{acetyl}C=O), 157.7 (C6), 153.9 (d, *J* = 2.5 Hz, C2), 150.0 (C4), 137.1 (C8), 119.0 (C5), 86.3 (C1'), 79.6 (C4'), 72.5 (C2'), 70.5 (C3'), 63.6 (C5'), 20.6 ($^{acetyl}CH_3$), 20.4 ($^{acetyl}CH_3$), 20.3 ($^{acetyl}CH_3$),.

^{19}F NMR (471 MHz, DMSO-d_6) δ 50.8.

HR-MS (ESI): m/z calcd. for M-H$^+$ [$C_{16}H_{17}FN_5O_{10}S$]$^-$ 490.0686, found:490.0685.

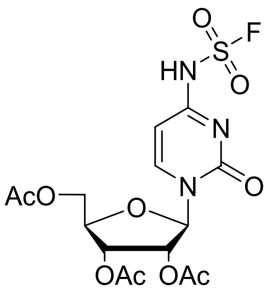

(2R,3R,4R,5R)-2-(acetoxymethyl)-5-(4-((fluorosulfonyl)amino)-2-oxopyrimidin-1(2H)-yl)tetrahydrofuran-3,4-diyl diacetate (3):

Obtained starting from **Ac_3C** (1.11 g, 3 mmol), beige solid, yield 1.01 g (75%)

^{1}H NMR (500 MHz, Chloroform-*d*) δ 7.74 (d, *J* = 8.0 Hz, 1H, C6-H), 6.45 (d, *J* = 8.0 Hz, 1H, C5-H), 5.99 (d, *J* = 5.0 Hz, 1H, C1'-H), 5.37 (m, 1H, C2'-H), 5.31 (dd, *J* = 5.9, 4.8 Hz, 1H, C3'-H), 4.45 – 4.39 (m, 2H, C4'-H and 1 x C5'-H), 4.34 (dd, *J* = 13.7, 3.9 Hz, 1H, 1 x C5'-H), 2.15 (s, 3H, $^{acetyl}CH_3$), 2.13 (s, 3H, $^{acetyl}CH_3$), 2.12 (s, 3H, $^{acetyl}CH_3$).

^{13}C NMR (126 MHz, Chloroform-*d*) δ 170.2 (^{acetyl}C=O), 170.0 (^{acetyl}C=O), 169.8 (^{acetyl}C=O), 160.7 (C4), 147.2 (C2), 142.7 (C6), 99.7 (C5), 88.9 (C1'), 80.7 (C4'), 73.3 (C2'), 70.1 (C3'), 62.9 (C5'), 20.9 ($^{acetyl}CH_3$), 20.6 ($^{acetyl}CH_3$), 20.5 ($^{acetyl}CH_3$).

^{19}F NMR (471 MHz, Chloroform-*d*) δ 52.4.

HR-MS (ESI): m/z calcd. for M-H$^+$ $[C_{15}H_{17}FN_3O_{10}S]^-$ 450.0624, found: 450.0627

General procedure 2 SuFEx reaction of SuFNucs **1-3** with amines

1, **2** or **3** (1 equiv), $Ca(NTf_2)_2$ (1.2 equiv) and DABCO (1.5 equiv)[a] were dissolved in DCM (0.5 M)[b,c]. To this mixture amine or amine salt (1.1 equiv) was added and the reaction mixture was stirred at room temperature until TLC analysis indicated full consumption of the starting material (approx. 1-2h, see Figure S4). The reaction mixture was diluted with DCM, washed twice with 0.5 M HCl and washed once with brine.[d] The organic phase was dried over Na_2SO_4, filtered and concentrated under reduced pressure. The product was purified using column chromatography (SiO_2, 0 to 5% MeOH in DCM).[b] Combined fractions containing pure product were concentrated under reduced pressure followed by drying under high vacuum.[e]

a) If the amine was used in the form of hydrochloride or acetate, 3 to 4 equiv. of DABCO were used.
b) For guanosine derivative **2** the reaction was performed in 3/1 DCM/THF mixture (0.25 M) and the chromatographic purification was performed using 0 to 15% MeOH in DCM as eluent.
c) If the clear solution is not formed a few drops of THF can be added to support dissolution.
d) In some cases during the extraction/washing steps it can take a substantial amount of time for the phases to separate. This can be overcome by transferring the whole mixture into the falcon tube and short centrifugation.
e) If the product did not solidify (oil or film), it was dissolved in a few drops of DCM and then approx. 5 ml of Et_2O was added and resulting solution was concentrated under reduced pressure followed by drying under high vacuum.

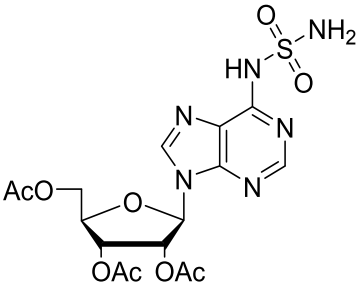

(2R,3R,4R,5R)-2-(acetoxymethyl)-5-(6-(sulfamoylamino)-9H-purin-9-yl)tetrahydrofuran-3,4-diyl diacetate (4a):

Obtained starting from **1** (237 mg, 0.5 mmol), ammonium acetate (54 mg, 0.7 mmol), $Ca(NTf_2)_2$ (360 mg, 0.6 mmol) and DABCO (224 mg, 2.0 mmol). White solid, yield: 70 mg (30%).

^{1}H NMR (500 MHz, Chloroform-*d*) δ 8.64 (s, 1H), 8.25 (s, 1H), 6.27 (bs, 2H), 6.25 (d, *J* = 5.6 Hz, 1H), 5.84 (t, *J* = 5.5 Hz, 1H), 5.59 (dd, *J* = 5.5, 4.3 Hz, 1H), 4.48 (q, *J* = 4.0 Hz, 1H), 4.42 (d, *J* = 3.9 Hz, 2H), 2.17 (s, 3H), 2.15 (s, 3H), 2.09 (s, 3H).

^{13}C NMR (126 MHz, Chloroform-*d*) δ 170.6, 169.8, 169.6, 150.8, 150.3, 149.7, 140.8, 121.4, 86.5, 80.9, 73.5, 70.8, 63.2, 21.0, 20.7, 20.5.

HR-MS (ESI): m/z calcd. for M-H$^+$ $[C_{16}H_{19}N_6O_9S]^-$ 471.0940, found: 471.0940.

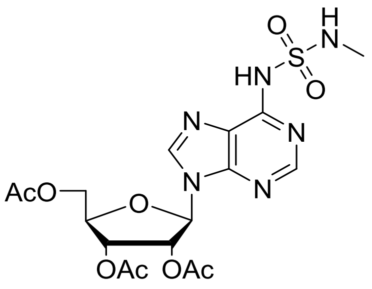

(2R,3R,4R,5R)-2-(acetoxymethyl)-5-(6-((N-methylsulfamoyl)amino)-9H-purin-9-yl)tetrahydrofuran-3,4-diyl diacetate (4b):

Obtained starting from **1** (237 mg, 0.5 mmol), $MeNH_4Cl$ (47 mg, 0.7 mmol), $Ca(NTf_2)_2$ (360 mg, 0.6 mmol) and DABCO (224 mg, 2.0 mmol). White solid, yield: 100 mg (41%).

^{1}H NMR (500 MHz, Chloroform-*d*) δ 8.70 (s, 1H), 8.33 (s, 1H), 6.25 (d, *J* = 5.4 Hz, 1H), 5.91 (t, *J* = 5.5 Hz, 1H), 5.62 (dd, *J* = 5.5, 4.4 Hz, 1H), 4.51 – 4.46 (m, 1H), 4.47 – 4.37 (m, 3H), 2.81 (s, 3H), 2.16 (s, 3H), 2.15 (s, 3H), 2.09 (s, 3H).

^{13}C NMR (126 MHz, Chloroform-*d*) δ 170.5, 169.7, 169.5, 151.8, 150.8, 149.6, 141.4, 86.8, 80.9, 73.3, 70.7, 63.2, 30.1, 21.0, 20.7, 20.5.

HR-MS (ESI): m/z calcd. for M-H$^+$ $[C_{17}H_{21}N_6O_9S]^-$ 485.1096, found: 485.1101.

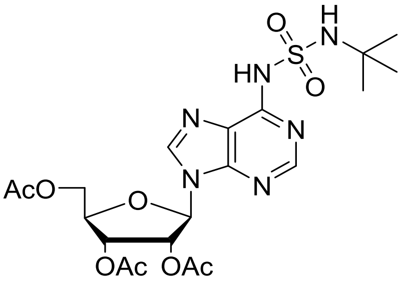

(2R,3R,4R,5R)-2-(acetoxymethyl)-5-(6-((N-(tert-butyl)sulfamoyl)amino)-9H-purin-9-yl)tetrahydrofuran-3,4-diyl diacetate (4c):

Obtained starting from **1** (119 mg, 0.25 mmol), $tBuNH_2$ (47 mg, 0.30 mmol), $Ca(NTf_2)_2$ (180 mg, 0.30 mmol) and DABCO (42 mg, 0.38 mmol). White solid, yield: 73 mg (55%).

^{1}H NMR (500 MHz, Chloroform-*d*) δ 8.77 (s, 1H), 8.28 (s, 1H), 6.25 (d, *J* = 5.4 Hz, 1H), 5.90 (t, *J* = 5.5 Hz, 1H), 5.63 (dd, *J* = 5.6, 4.4 Hz, 1H), 4.50 – 4.38 (m, 2H), 2.16 (s, 3H), 2.14 (s, 3H), 2.09 (s, 3H), 1.34 (s, 9H).

^{13}C NMR (126 MHz, Chloroform-*d*) δ 170.5, 169.7, 169.5, 151.7, 150.9, 150.2, 140.8, 120.8, 86.6, 80.8, 73.3, 70.7, 63.2, 55.0, 29.8, 20.9, 20.7, 20.5.

HR-MS (ESI): m/z calcd. for M-H$^+$ $[C_{20}H_{27}N_6O_9S]^-$ 527.1566, found: 527.1568.

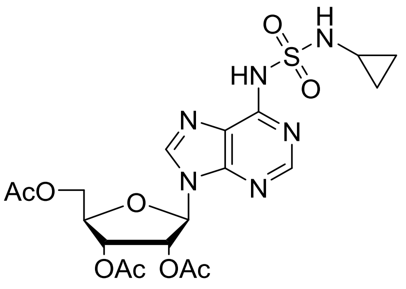

(2R,3R,4R,5R)-2-(acetoxymethyl)-5-(6-((N-cyclopropylsulfamoyl)amino)-9H-purin-9-yl)tetrahydrofuran-3,4-diyl diacetate (4d):

Obtained starting from **1** (237 mg, 0.5 mmol), cyclopropyl amine (42 ul, 0.6 mmol), $Ca(NTf_2)_2$ (360 mg, 0.6 mmol) and DABCO (84 mg, 0.75 mmol). White solid, yield: 113 mg (44%).

^{1}H NMR (500 MHz, Chloroform-*d*) δ 8.76 (s, 1H), 8.32 (s, 1H), 6.25 (d, *J* = 5.5 Hz, 1H), 5.92 (t, *J* = 5.5 Hz, 1H), 5.63 (dd, *J* = 5.6, 4.4 Hz, 1H), 4.50 – 4.43 (m, 1H), 4.44 – 4.36 (m, 2H), 2.44 (tt, *J* = 6.8, 3.5 Hz, 1H), 2.16 (s, 3H), 2.15 (s, 3H), 2.08 (s, 3H), 0.86 – 0.76 (m, 1H), 0.70 – 0.61 (m, 1H).

^{13}C NMR (126 MHz, Chloroform-*d*) δ 170.5, 169.7, 169.5, 151.6, 150.9, 150.2, 141.4, 121.1, 86.6, 80.8, 77.4, 76.9, 70.8, 63.2, 25.2, 20.9, 20.7, 20.5, 6.1, 6.0.

HR-MS (ESI): m/z calcd. for M-H$^+$ $[C_{19}H_{23}N_6O_9S]^-$ 511.1253, found: 511.1253.

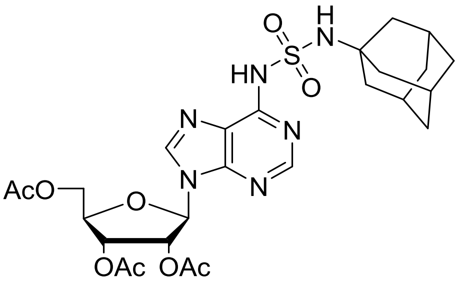

(2R,3R,4R,5R)-2-(acetoxymethyl)-5-(6-((N-((1s,3S)-adamantan-1-yl)sulfamoyl)amino)-9H-purin-9-yl)tetrahydrofuran-3,4-diyl diacetate (4e):

Obtained starting from **1** (237 mg, 0.5 mmol), 1-adamantylamine (90 mg, 0.6 mmol), $Ca(NTf_2)_2$ (360 mg, 0.6 mmol) and DABCO (84 mg, 0.75 mmol). White solid, yield: 139mg (45%).

^{1}H NMR (500 MHz, Chloroform-*d*) δ 8.75 (s, 1H), 8.28 (s, 1H), 6.26 (d, *J* = 5.4 Hz, 1H), 5.89 (t, *J* = 5.5 Hz, 1H), 5.64 (t, *J* = 5.0 Hz, 1H), 4.51 – 4.44 (m, 1H), 4.46 – 4.37 (m, 2H), 2.16 (s, 3H), 2.15 (s, 3H), 2.09 (s, 3H), 2.04 (s, 3H), 1.99 – 1.90 (m, 6H), 1.65 – 1.55 (m, 6H).

^{13}C NMR (126 MHz, Chloroform-*d*) δ 170.5, 169.7, 169.5, 151.6, 150.9, 150.1, 140.8, 120.6, 86.6, 80.8, 73.4, 70.7, 63.2, 55.7, 42.6, 36.0, 29.6, 21.0, 20.7, 20.5.

HR-MS (ESI): m/z calcd. for M-H$^+$ $[C_{26}H_{33}N_6O_9S]^-$ 605.2035, found: 605.2043.

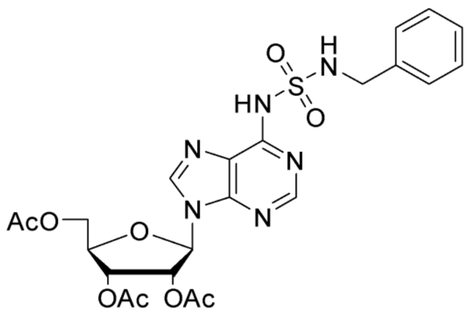

(2R,3R,4R,5R)-2-(acetoxymethyl)-5-(6-((N-benzylsulfamoyl)amino)-9H-purin-9-yl)tetrahydrofuran-3,4-diyl diacetate (4f)

Obtained starting from **1** (119 mg, 0.25 mmol), $BnNH_2$ (66 ul, 0.30 mmol), $Ca(NTf_2)_2$ (180 mg, 0.30 mmol) and DABCO (42 mg, 0.38 mmol). White solid, yield: 70mg (50%).

^{1}H NMR (500 MHz, Chloroform-*d*) δ 8.67 (s, 1H), 8.25 (s, 1H), 7.26 – 7.12 (m, 5H), 6.22 (d, *J* = 5.3 Hz, 1H), 5.88 (t, *J* = 5.5 Hz, 1H), 5.64 – 5.59 (m, 1H), 4.50 – 4.45 (m, 1H), 4.51 – 4.37 (m, 2H), 4.29 (s, 2H), 2.16 (s, 3H), 2.13 (s, 3H), 2.10 (s, 3H).

^{13}C NMR (126 MHz, Chloroform-*d*) δ 170.5, 169.7, 169.5, 151.0, 150.5, 150.0, 140.9, 136.1, 128.6, 128.3, 127.9, 120.8, 86.7, 80.8, 73.4, 70.7, 63.1, 48.2, 20.9, 20.7, 20.5.

HR-MS (ESI): m/z calcd. for M-H^+ $[C_{23}H_{25}N_6O_9S]^-$ 561.1409, found: 561.1407.

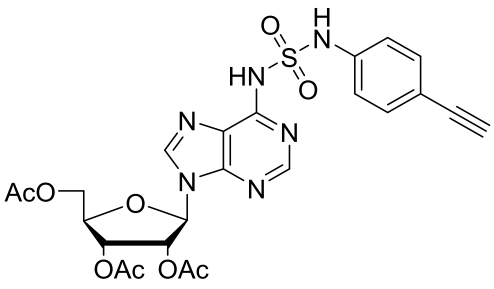

(2R,3R,4R,5R)-2-(acetoxymethyl)-5-(6-((N-(4-ethynylphenyl)sulfamoyl)amino)-9H-purin-9-yl)tetrahydrofuran-3,4-diyl diacetate (4g)

Obtainedstarting from **1** (237 mg, 0.5 mmol), 1-Amino-4-ethynylbenzene (70 mg, 0.6 mmol), $Ca(NTf_2)_2$ (360 mg, 0.6 mmol) and DABCO (84 mg, 0.75 mmol). Beige solid, yield: 217mg (76%).

^{1}H NMR (500 MHz, Chloroform-*d*) δ 9.39 (s, 1H), 8.70 (s, 1H), 8.30 (s, 1H), 7.32 (d, *J* = 8.6 Hz, 2H), 7.09 (d, *J* = 8.7 Hz, 2H), 6.26 (d, *J* = 5.6 Hz, 1H), 5.80 (t, *J* = 5.6 Hz, 1H), 5.58 (dd, *J* = 5.6, 4.3 Hz, 1H), 4.53 – 4.47 (m, 1H), 4.45 – 4.41 (m, 2H), 2.98 (s, 1H), 2.17 (s, 3H), 2.17 (s, 3H), 2.10 (s, 3H).

^{13}C NMR (126 MHz, Chloroform-*d*) δ 170.4, 169.7, 169.5, 151.1, 150.3, 148.3, 140.4, 138.5, 133.4, 127.6, 121.7, 120.6, 120.3, 118.5, 86.6, 83.2, 81.0, 73.6, 70.7, 63.2, 21.0, 20.7, 20.5.

HR-MS (ESI): m/z calcd. for M-H^+ $[C_{24}H_{23}N_6O_9S]^-$ 571.1253, found: 571.1255.

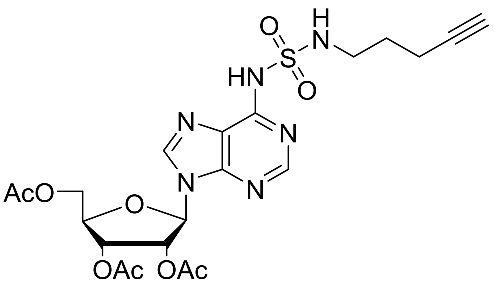

(2R,3R,4R,5R)-2-(acetoxymethyl)-5-(6-((N-(pent-4-yn-1-yl)sulfamoyl)amino)-9H-purin-9-yl)tetrahydrofuran-3,4-diyl diacetate (4h):

Obtainedstarting from **1** (950 mg, 2.0 mmol), 4-pentyn-1-amine (250 ul, 2.4 mmol), $Ca(NTf_2)_2$ (1440 mg, 2.4 mmol) and DABCO (336 mg, 3.0 mmol). White solid, yield: 742 mg (69%).

^{1}H NMR (500 MHz, Chloroform-*d*) δ 8.74 (s, 1H), 8.32 (s, 1H), 6.24 (d, *J* = 5.5 Hz, 1H), 5.91 (t, *J* = 5.5 Hz, 1H), 5.63 (t, *J* = 5.0 Hz, 1H), 4.50 – 4.44 (m, 1H), 4.44 – 4.36 (m, 2H), 3.22 (t, *J* = 6.8 Hz, 2H), 2.29 (td, *J* = 7.0, 2.7 Hz, 2H), 2.16 (s, 3H), 2.14 (s, 3H), 2.08 (s, 3H), 1.91 (t, *J* = 2.7 Hz, 1H), 1.85 – 1.76 (m, 2H).

^{13}C NMR (126 MHz, Chloroform-*d*) δ 170.5, 169.7, 169.5, 151.5, 150.8, 150.1, 141.3, 120.9, 86.7, 82.9, 80.8, 73.3, 70.7, 69.5, 63.2, 42.7, 28.1, 20.9, 20.7, 20.5, 15.9.

HR-MS (ESI): m/z calcd. for $M\text{-}H^+$ $[C_{21}H_{25}N_6O_9S]^-$ 537.1409, found: 537.1416.

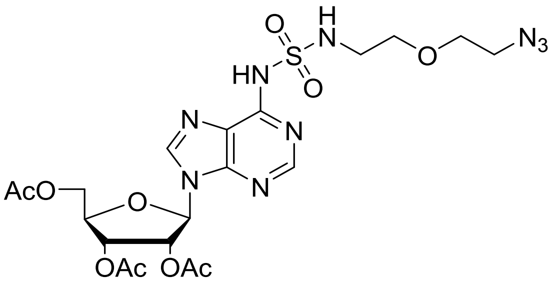

(2R,3R,4R,5R)-2-(acetoxymethyl)-5-(6-((N-(2-(2-azidoethoxy)ethyl)sulfamoyl)amino)-9H-purin-9-yl)tetrahydrofuran-3,4-diyl diacetate (4i):

Obtained starting from **1** (950 mg, 2.0 mmol), 2-(2-azidoethoxy)ethan-1-amine (312 mg, 2.4 mmol), $Ca(NTf_2)_2$ (1440 mg, 2.4 mmol) and DABCO (336 mg, 3.0 mmol). White solid, yield: 819 mg (70%).

^{1}H NMR (500 MHz, Chloroform-*d*) δ 8.72 (s, 1H), 8.34 (s, 1H), 6.25 (d, *J* = 5.6 Hz, 1H), 5.89 (t, *J* = 5.6 Hz, 1H), 5.61 (dd, *J* = 5.6, 4.3 Hz, 1H), 4.49 – 4.44 (m, 1H), 4.45 – 4.37 (m, 2H), 3.63 (t, *J* = 5.1 Hz, 2H), 3.53 (dd, *J* = 5.4, 4.4 Hz, 2H), 3.32 (t, *J* = 5.0 Hz, 2H), 3.25 (dd, *J* = 5.5, 4.3 Hz, 2H), 2.16 (s, 3H), 2.15 (s, 3H), 2.08 (s, 3H).

^{13}C NMR (126 MHz, Chloroform-*d*) δ 170.5, 169.7, 169.5, 151.1, 150.7, 150.1, 141.2, 120.8, 86.6, 80.9, 73.3, 70.8, 70.0, 69.4, 63.2, 50.8, 43.9, 20.9, 20.7, 20.5.

HR-MS (ESI): m/z calcd. for $M\text{-}H^+$ $[C_{20}H_{26}N_9O_{10}S]^-$ 584.1529, found: 584.1537.

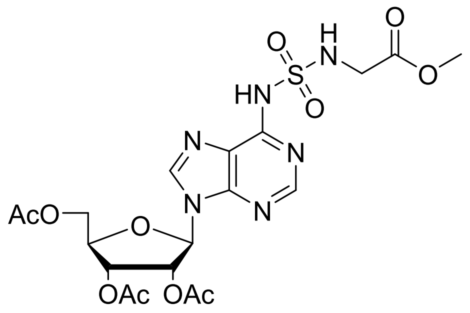

(2R,3R,4R,5R)-2-(acetoxymethyl)-5-(6-((N-(2-methoxy-2-oxoethyl)sulfamoyl)amino)-9H-purin-9-yl)tetrahydrofuran-3,4-diyl diacetate (4j):

Obtained starting from **1** (237 mg, 0.5 mmol), glycine methyl ester hydrochloride (75 mg, 0.6 mmol), $Ca(NTf_2)_2$ (360 mg, 0.6 mmol) and DABCO (168 mg, 1.5 mmol). White solid, yield: 127 mg (46%).

^{1}H NMR (500 MHz, Chloroform-*d*) δ 8.69 (s, 1H), 8.30 (s, 1H), 6.25 (d, *J* = 5.5 Hz, 1H), 5.85 (t, *J* = 5.5 Hz, 1H), 5.59 (dd, *J* = 5.5, 4.4 Hz, 1H), 4.50 – 4.44 (m, 1H), 4.46 – 4.36 (m, 2H), 3.99 (s, 2H), 3.64 (s, 3H), 2.16 (s, 3H), 2.15 (s, 3H), 2.09 (s, 3H).

^{13}C NMR (126 MHz, Chloroform-*d*) δ 170.5, 169.7, 169.6, 169.5, 150.7, 150.3, 149.5, 140.7, 121.5, 86.6, 80.9, 73.4, 70.7, 63.1, 52.6, 45.3, 20.9, 20.7, 20.5.

HR-MS (ESI): m/z calcd. for $M-H^+$ $[C_{19}H_{23}N_6O_{11}S]^-$ 543.1151, found: 543.1165.

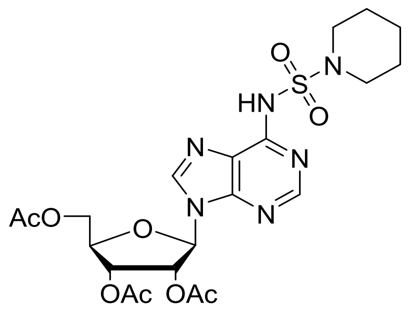

(2R,3R,4R,5R)-2-(acetoxymethyl)-5-(6-(piperidine-1-sulfonamido)-9H-purin-9-yl)tetrahydrofuran-3,4-diyl diacetate (4k):

Obtained starting from **1** (237 mg, 0.5 mmol), piperidine (60 ul, 0.6 mmol), $Ca(NTf_2)_2$ (360 mg, 0.6 mmol) and DABCO (84 mg, 0.75 mmol). White solid, yield: 190 mg (70%).

^{1}H NMR (500 MHz, Chloroform-*d*) δ 8.38 (bs, 1H), 8.23 (bs, 1H), 6.19 (d, *J* = 5.2 Hz, 1H), 5.90 (t, *J* = 5.3 Hz, 1H), 5.60 (t, *J* = 5.1 Hz, 1H), 4.55 – 4.31 (m, 3H), 3.38 (bs, 4H), 2.15 (s, 3H), 2.13 (s, 3H), 2.09 (s, 3H), 1.72 – 1.61 (m, 4H), 1.58 – 1.47 (m, 2H).

^{13}C NMR (126 MHz, Chloroform-*d*) δ 170.5, 169.7, 169.5, 149.1, 140.7, 86.9, 80.7, 73.3, 70.6, 63.1, 47.7, 25.4, 23.7, 20.9, 20.7, 20.5.

HR-MS (ESI): m/z calcd. for $M-H^+$ $[C_{21}H_{27}N_6O_9S]^-$ 539.1566, found: 539.1570.

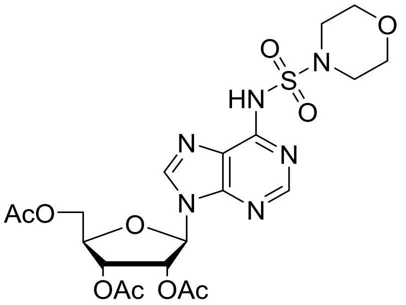

(2R,3R,4R,5R)-2-(acetoxymethyl)-5-(6-(morpholine-4-sulfonamido)-9H-purin-9-yl)tetrahydrofuran-3,4-diyl diacetate (4l)

Obtained starting from **1** (237 mg, 0.5 mmol), morpholine (53 ul , 0.6 mmol), $Ca(NTf_2)_2$ (360 mg, 0.6 mmol) and DABCO (84 mg, 0.75 mmol). White solid, yield: 173 mg (64%).

^{1}H NMR (500 MHz, Chloroform-*d*) δ 8.28 (bs, 1H), 8.12 (bs, 1H), 6.16 (d, *J* = 5.2 Hz, 1H), 5.89 (t, *J* = 5.3 Hz, 1H), 5.59 (t, *J* = 5.1 Hz, 1H), 4.50 – 4.34 (m, 3H), 3.86 – 3.68 (m, 4H), 3.37 (bs, 4H), 2.15 (s, 3H), 2.13 (s, 3H), 2.09 (s, 2H).

^{13}C NMR (126 MHz, Chloroform-*d*) δ 170.4, 169.7, 169.5, 149.0, 140.7, 86.8, 80.6, 73.4, 70.6, 66.3, 63.1, 46.8, 20.9, 20.7, 20.5.

HR-MS (ESI): m/z calcd. for M-H$^+$ $[C_{20}H_{25}N_6O_9S]^-$ 541.1358, found: 541.1263.

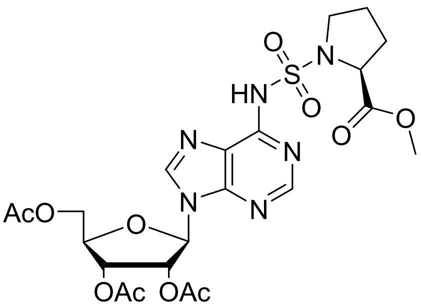

(2R,3R,4R,5R)-2-(acetoxymethyl)-5-(6-(((S)-2-(methoxycarbonyl)pyrrolidine)-1-sulfonamido)-9H-purin-9-yl)tetrahydrofuran-3,4-diyl diacetate (4m):

Obtained starting from **1** (237 mg, 0.5 mmol), proline methyl ester hydrochloride (100 mg, 0.6 mmol), $Ca(NTf_2)_2$ (360 mg, 0.6 mmol) and DABCO (168 mg, 1.5 mmol). White solid, yield: 126 mg (43%).

^{1}H NMR (500 MHz, Chloroform-*d*) δ 8.46 (s, 1H), 8.38 (s, 1H), 6.22 (d, *J* = 5.1 Hz, 1H), 5.93 (t, *J* = 5.4 Hz, 1H), 5.62 (t, *J* = 5.1 Hz, 1H), 4.50 – 4.32 (m, 3H), 3.79 (s, 3H), 3.59 – 3.50 (m, 1H), 2.36 – 2.24 (m, 2H), 2.15 (s, 3H), 2.12 (s, 3H), 2.08 (s, 3H), 2.07 – 2.01 (m, 2H), 2.01 – 1.92 (m, 2H).

^{13}C NMR (126 MHz, Chloroform-*d*) δ 170.5, 169.7, 169.5, 149.3, 141.3, 87.0, 80.6, 73.2, 70.6, 63.1, 61.6, 53.0, 49.1, 30.9, 25.2, 20.9, 20.7, 20.5.

HR-MS (ESI): m/z calcd. for M-H$^+$ $[C_{22}H_{27}N_6O_{11}S]^-$ 583.1464, found: 583.1470.

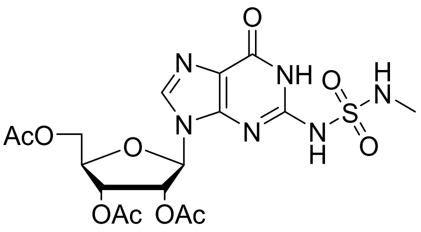

(2R,3R,4R,5R)-2-(acetoxymethyl)-5-(2-((N-methylsulfamoyl)amino)-6-oxo-1,6-dihydro-9H-purin-9-yl)tetrahydrofuran-3,4-diyl diacetate (5a):

Obtained starting from **2** (245 mg, 0.5 mmol), $MeNH_4Cl$ (47 mg, 0.7 mmol), $Ca(NTf_2)_2$ (360 mg, 0.6 mmol) and DABCO (224 mg, 2.0 mmol). White solid, yield: 117 mg (46%).

^{1}H NMR (500 MHz, DMSO-d_6) δ 10.61 (bs, 1H), 8.17 (s, 1H), 7.46 (bs, 1H), 6.27 (d, J = 5.1 Hz, 1H), 5.72 (dd, J = 6.4, 5.1 Hz, 1H), 5.50 – 5.46 (m, 1H), 4.41 – 4.31 (m, 2H), 4.26 (dd, J = 11.5, 5.9 Hz, 1H), 2.61 (s, 3H), 2.12 (s, 3H), 2.04 (s, 3H), 2.03 (s, 3H).

^{13}C NMR (126 MHz, DMSO-d_6) δ 170.1, 169.6, 169.4, 137.6, 85.1, 79.3, 72.7, 69.9, 63.2, 28.9, 20.5, 20.3, 20.2.

HR-MS (ESI): m/z calcd. for M-H$^+$ $[C_{17}H_{21}N_6O_{10}S]^-$ 501.1045, found: 501.1039.

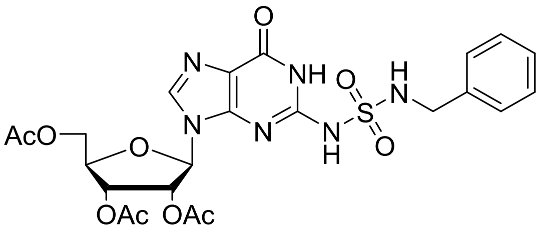

(2R,3R,4R,5R)-2-(acetoxymethyl)-5-(2-((N-benzylsulfamoyl)amino)-6-oxo-1,6-dihydro-9H-purin-9-yl)tetrahydrofuran-3,4-diyl diacetate (5b):

Obtained starting from **2** (245 mg, 0.5 mmol), benzyl amine (66 ul, 0.6 mmol), $Ca(NTf_2)_2$ (360 mg, 0.6 mmol) and DABCO (84 mg, 0.75 mmol). White solid, yield: 127 mg (43%).

^{1}H NMR (500 MHz, DMSO-d_6) δ 7.98 (s, 1H), 7.31 – 7.26 (m, 2H), 7.25 – 7.20 (m, 2H), 7.19 – 7.15 (m, 1H), 6.07 (d, J = 5.7 Hz, 1H), 5.77 (t, J = 6.0 Hz, 1H), 5.47 (dd, J = 6.2, 4.7 Hz, 1H), 4.34 (dd, J = 11.5, 3.5 Hz, 1H), 4.30 – 4.26 (m, 1H), 4.23 (dd, J = 11.5, 6.0 Hz, 1H), 4.11 – 3.99 (m, 2H), 2.07 (s, 3H), 2.03 (s, 3H), 1.96 (s, 3H).

^{13}C NMR (126 MHz, DMSO-d_6) δ 170.1, 169.6, 169.3, 157.1, 150.3, 138.5, 135.8, 134.0, 128.0, 127.6, 126.8, 120.8, 117.9, 84.5, 79.3, 72.3, 70.2, 63.2, 46.7, 20.5, 20.3, 20.1.

HR-MS (ESI): m/z calcd. for M-H$^+$ $[C_{23}H_{25}N_6O_{10}S]^-$ 577.1358, found: 577.1357.

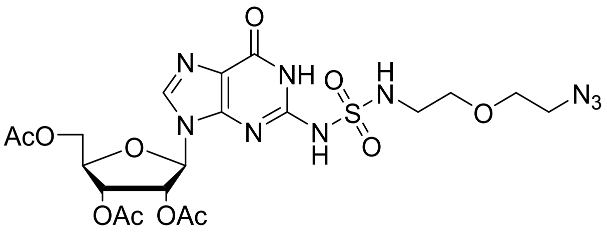

(2R,3R,4R,5R)-2-(acetoxymethyl)-5-(2-((N-(2-(2-azidoethoxy)ethyl)sulfamoyl)amino)-6-oxo-1,6-dihydro-9H-purin-9-yl)tetrahydrofuran-3,4-diyl diacetate (5c):

Obtained starting from **2** (245 mg, 0.5 mmol), 2-(2-azidoethoxy)ethan-1-amine (85 ul, 0.6 mmol), $Ca(NTf_2)_2$ (360 mg, 0.6 mmol) and DABCO (84 mg, 0.75 mmol). White solid, yield: 72 mg (25%)

^{1}H NMR (500 MHz, DMSO-d_6) δ 10.37 (s, 1H), 7.87 (s, 1H), 6.03 (d, J = 5.5 Hz, 1H), 5.80 (t, J = 5.9 Hz, 1H), 5.49 (dd, J = 6.3, 4.9 Hz, 1H), 4.36 (dd, J = 11.6, 3.7 Hz, 1H), 4.33 – 4.29 (m, 1H), 4.24 (dd, J = 11.5, 6.0 Hz, 1H), 3.52 – 3.45 (m, 4H), 3.29 – 3.25 (m, 2H), 3.01 – 2.90 (m, 2H), 2.12 (s, 3H), 2.05 (s, 3H), 2.03 (s, 3H).

^{13}C NMR (126 MHz, DMSO-d_6) δ 170.1, 169.6, 169.4, 157.7, 150.9, 135.3, 117.3, 84.8, 79.3, 72.4, 70.2, 69.1, 69.0, 63.3, 49.8, 42.8, 20.5, 20.3, 20.2.

HR-MS (ESI): m/z calcd. for M-H$^+$ $[C_{20}H_{26}N_9O_{11}S]^-$ 600.1478, found: 600.1474.

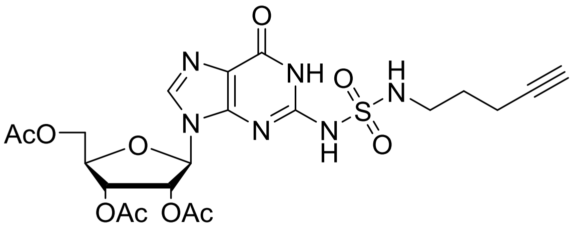

(2R,3R,4R,5R)-2-(acetoxymethyl)-5-(6-oxo-2-((N-(pent-4-yn-1-yl)sulfamoyl)amino)-1,6-dihydro-9H-purin-9-yl)tetrahydrofuran-3,4-diyl diacetate (5d):

Obtained starting from **2** (245 mg, 0.5 mmol), 4-pentyn-1-amine (67 ul, 0.6 mmol), $Ca(NTf_2)_2$ (360 mg, 0.6 mmol) and DABCO (84 mg, 0.75 mmol). White solid, yield: 74 mg (33%).

^{1}H NMR (500 MHz, DMSO-d_6) δ 10.37 (s, 1H), 7.89 (s, 1H), 6.05 (d, J = 5.7 Hz, 1H), 5.80 (t, J = 6.0 Hz, 1H), 5.48 (dd, J = 6.2, 4.8 Hz, 1H), 4.35 (dd, J = 11.5, 3.7 Hz, 1H), 4.33 – 4.30 (m, 1H), 4.23 (dd, J = 11.5, 5.8 Hz, 1H), 2.91 – 2.79 (m, 2H), 2.68 (t, J = 2.6 Hz, 1H), 2.16 (td, J = 7.2, 2.6 Hz, 2H), 2.12 (s, 3H), 2.05 (s, 3H), 2.04 (s, 3H), 1.65 – 1.55 (m, 2H).

^{13}C NMR (126 MHz, DMSO-d_6) δ 170.1, 169.6, 169.4, 157.6, 151.0, 135.4, 117.3, 84.5, 84.1, 79.2, 72.2, 71.2, 70.2, 63.3, 42.1, 28.2, 20.5, 20.4, 20.2, 15.4.

HR-MS (ESI): m/z calcd. for M-H$^+$ $[C_{21}H_{25}N_6O_{10}S]^-$ 553.1358, found: 553.1350.

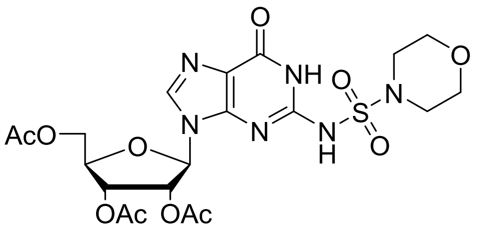

(2R,3R,4R,5R)-2-(acetoxymethyl)-5-(2-(morpholine-4-sulfonamido)-6-oxo-1,6-dihydro-9H-purin-9-yl)tetrahydrofuran-3,4-diyl diacetate (5e):

Obtained starting from **2** (245 mg, 0.5 mmol), morpholine (60 ul, 0.6 mmol), $Ca(NTf_2)_2$ (360 mg, 0.6 mmol) and DABCO (84 mg, 0.75 mmol). White solid, yield 122 mg (43%).

^{1}H NMR (500 MHz, DMSO-d_6) δ 10.76 (s, 1H), 8.21 (s, 1H), 6.05 (d, J = 6.1 Hz, 1H), 5.74 (t, J = 6.2 Hz, 1H), 5.43 (dd, J = 6.2, 4.1 Hz, 1H), 4.40 – 4.34 (m, 2H), 4.28 (dd, J = 13.0, 7.3 Hz, 1H), 3.68 – 3.58 (m, 4H), 3.33 – 3.25 (m, 4H), 2.10 (s, 3H), 2.04 (s, 3H), 2.04 (s, 3H).

^{13}C NMR (126 MHz, DMSO-d_6) δ 170.1, 169.4, 169.3, 155.8, 148.5, 147.1, 137.4, 84.7, 79.7, 72.4, 70.1, 65.7, 63.1, 46.4, 40.0, 20.5, 20.3, 20.1.

HR-MS (ESI): m/z calcd. for M-H$^+$ $[C_{20}H_{25}N_6O_{11}S]^-$ 577.1307, found: 577.1297.

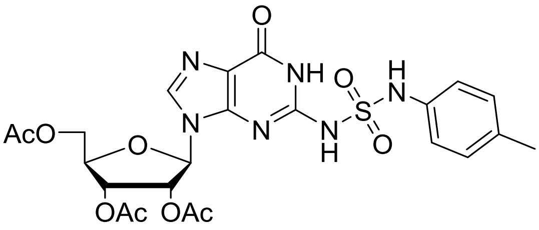

(2R,3R,4R,5R)-2-(acetoxymethyl)-5-(6-oxo-2-((N-(p-tolyl)sulfamoyl)amino)-1,6-dihydro-9H-purin-9-yl)tetrahydrofuran-3,4-diyl diacetate (5f):

Obtainedstarting from **2** (245 mg, 0.5 mmol), p-toluidine (65 mg, 0.6 mmol), $Ca(NTf_2)_2$ (360 mg, 0.6 mmol) and DABCO (84mg, 0.75 mmol). White solid, yield: 92 mg (32%).

^{1}H NMR (500 MHz, DMSO-d_6) δ 10.54 (bs, 1H), 10.18 (bs, 1H), 8.26 (s, 1H), 7.08 (s, 4H), 6.44 (d, J = 5.5 Hz, 1H), 5.79 – 5.72 (m, 1H), 5.56 – 5.45 (m, 1H), 4.43 – 4.35 (m, 2H), 4.30 – 4.22 (m, 1H), 2.21 (s, 3H), 2.15 (s, 3H), 2.04 (s, 3H), 2.04 (s, 3H).

^{13}C NMR (126 MHz, DMSO-d_6) δ 170.1, 169.7, 169.3, 137.6, 134.4, 133.6, 129.5, 120.4, 84.3, 79.3, 72.6, 69.7, 64.9, 63.1, 54.9, 20.5, 20.4, 20.3, 20.2, 15.2.

HR-MS (ESI): m/z calcd. for M-H$^+$ $[C_{23}H_{25}N_6O_{10}S]^-$ 577.1358, found: 577.1351.

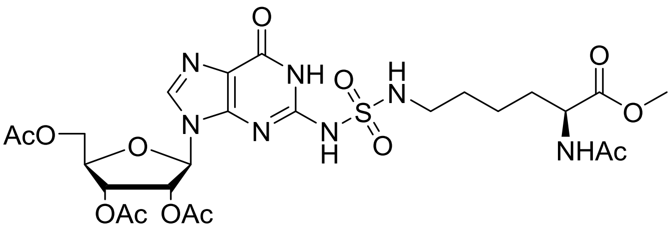

(2R,3R,4R,5R)-2-(2-((N-((S)-5-acetamido-6-methoxy-6-oxohexyl)sulfamoyl)amino)-6-oxo-1,6-dihydro-9H-purin-9-yl)-5-(acetoxymethyl)tetrahydrofuran-3,4-diyl diacetate (5g):

Obtained starting from **2** (245 mg, 0.5 mmol), Nα-acetyl-L-lysine methyl ester hydrochloride (143 mg, 0.6 mmol), $Ca(NTf_2)_2$ (360 mg, 0.6 mmol) and DABCO (168 mg, 1.5 mmol). White solid, yield: 124 mg (39%).

^{1}H NMR (500 MHz, DMSO-d_6) δ 10.49 (bs, 1H), 8.17 (d, J = 7.4 Hz, 1H), 8.00 (s, 1H), 6.12 (d, J = 5.3 Hz, 1H), 5.79 (dd, J = 6.3, 5.3 Hz, 1H), 5.49 (dd, J = 6.2, 5.2 Hz, 1H), 4.39 – 4.29 (m, 2H), 4.23 (dd, J = 11.5, 5.9 Hz, 1H), 4.14 (ddd, J = 9.0, 7.4, 5.2 Hz, 1H), 3.58 (s, 3H), 2.90 – 2.78 (m, 2H), 2.12 (s, 3H), 2.04 (s, 3H), 2.03 (s, 3H), 1.82 (s, 3H), 1.66 – 1.56 (m, 1H), 1.56 – 1.48 (m, 1H), 1.47 – 1.39 (m, 2H), 1.35 – 1.25 (m, 2H).

^{13}C NMR (126 MHz, DMSO-d_6) δ 172.8, 170.1, 169.6, 169.4, 169.4, 157.0, 150.1, 136.4, 120.8, 118.2, 84.9, 79.2, 72.4, 70.1, 63.2, 51.9, 51.7, 42.7, 30.6, 28.5, 22.8, 22.2, 20.5, 20.3, 20.2.

HR-MS (ESI): m/z calcd. for $M-H^+$ $[C_{25}H_{34}N_7O_{13}S]^-$ 672.1941, found: 672.1936.

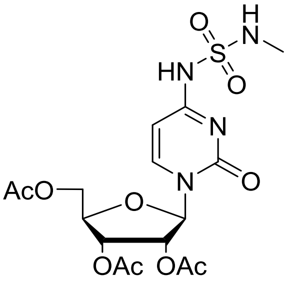

(2R,3R,4R,5R)-2-(acetoxymethyl)-5-(4-((N-methylsulfamoyl)amino)-2-oxopyrimidin-1(2H)-yl)tetrahydrofuran-3,4-diyl diacetate (6a):

Obtained starting from **3** (113 mg, 0.25 mmol), $MeNH_3Cl$ (33 mg, 0.5 mmol), $Ca(NTf_2)_2$ (180 mg, 0.30 mmol) and DABCO (112 mg, 1.0 mmol). White solid, yield: 36 mg (31%)

^{1}H NMR (500 MHz, Chloroform-d) δ 7.43 (d, J = 8.1 Hz, 1H), 5.99 (d, J = 5.2 Hz, 1H), 5.34 (t, J = 5.5 Hz, 1H), 5.33 – 5.27 (m, 1H), 4.42 – 4.28 (m, 3H), 2.76 (s, 3H), 2.14 (s, 3H), 2.12 (s, 3H), 2.10 (s, 3H).

^{13}C NMR (126 MHz, Chloroform-d) δ 170.3, 169.8, 169.8, 139.1, 88.0, 80.3, 73.0, 70.2, 63.1, 30.0, 20.9, 20.6, 20.5.

HR-MS (ESI): m/z calcd. for $M-H^+$ $[C_{16}H_{21}N_4O_{10}S]^-$ 461.0984, found: 461.0990.

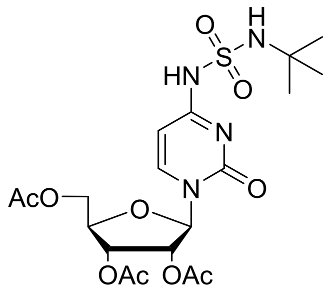

(2R,3R,4R,5R)-2-(acetoxymethyl)-5-(4-((N-(tert-butyl)sulfamoyl)amino)-2-oxopyrimidin-1(2H)-yl)tetrahydrofuran-3,4-diyl diacetate (6b):

Obtained starting from **3** (113 mg, 0.25 mmol), $tBuNH_2$ (35 ul, 0.30 mmol), $Ca(NTf_2)_2$ (180 mg, 0.30 mmol) and DABCO (42 mg, 0.38 mmol). White solid, yield: 54 mg (42%).

^{1}H NMR (500 MHz, Chloroform-*d*) δ 7.36 (s, 1H), 6.00 (d, *J* = 4.6 Hz, 1H), 5.77 (s, 1H), 5.36 – 5.28 (m, 2H), 4.41 – 4.29 (m, 3H), 2.14 (s, 3H), 2.12 (s, 3H), 2.10 (s, 3H), 1.35 (s, 9H).

^{13}C NMR (126 MHz, Chloroform-*d*) δ 170.2, 169.7, 138.4, 103.6, 87.7, 80.2, 77.4, 77.2, 76.9, 72.9, 70.2, 63.1, 30.1, 20.9, 20.6, 20.6.

HR-MS (ESI): m/z calcd. for M-H$^+$ $[C_{19}H_{27}N_4O_{10}S]^-$ 503.1453, found: 503.1460.

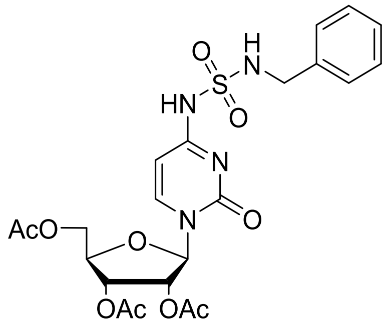

(2R,3R,4R,5R)-2-(acetoxymethyl)-5-(4-((N-benzylsulfamoyl)amino)-2-oxopyrimidin-1(2H)-yl)tetrahydrofuran-3,4-diyl diacetate (6c):

Obtained starting from **3** (113 mg, 0.25 mmol), benzyl amine (35 ul, 0.3 mmol), $Ca(NTf_2)_2$ (180 mg, 0.30 mmol) and DABCO (42 mg, 0.38 mmol). White solid, yield: 71 mg (52%).

^{1}H NMR (500 MHz, Chloroform-*d*) δ 7.38 (bs, 1H), 7.36 – 7.26 (m, 4H), 7.27 – 7.21 (m, 1H), 5.95 (d, *J* = 5.0 Hz, 1H), 5.38 – 5.23 (m, 2H), 4.40 – 4.29 (m, 3H), 4.27 (bs, 2H), 2.14 (s, 3H), 2.12 (s, 3H), 2.09 (s, 3H).

^{13}C NMR (126 MHz, Chloroform-*d*) δ 170.2, 169.7, 128.7, 128.4, 128.0, 127.9, 88.0, 80.2, 73.0, 70.1, 63.0, 48.0, 20.9, 20.6, 20.5.

HR-MS (ESI): m/z calcd. for M-H$^+$ $[C_{22}H_{25}N_4O_{10}S]^-$ 537.1297, found: 537.1302.

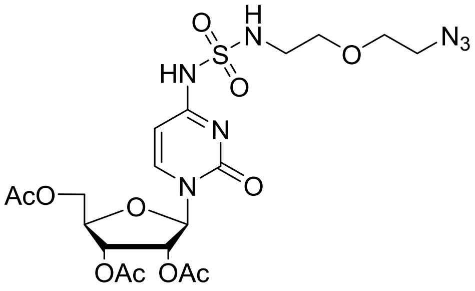

(2R,3R,4R,5R)-2-(acetoxymethyl)-5-(4-((N-(2-(2-azidoethoxy)ethyl)sulfamoyl)amino)-2-oxopyrimidin-1(2H)-yl)tetrahydrofuran-3,4-diyl diacetate (6d):

Obtained starting from **3** (113 mg, 0.25 mmol), 2-(2-azidoethoxy)ethan-1-amine (45 ul, 0.30 mmol), $Ca(NTf_2)_2$ (180 mg, 0.30 mmol) and DABCO (42 mg, 0.38 mmol). White solid, yield: 70 mg (50%).

^{1}H NMR (500 MHz, Chloroform-*d*) δ 7.40 (d, *J* = 8.1 Hz, 1H), 6.00 (d, *J* = 4.9 Hz, 1H), 5.37 – 5.26 (m, 2H), 4.41 – 4.30 (m, 3H), 3.69 – 3.63 (m, 4H), 3.41 – 3.36 (m, 2H), 3.35 – 3.30 (m, 2H), 2.14 (s, 3H), 2.12 (s, 3H), 2.10 (s, 3H).

^{13}C NMR (126 MHz, Chloroform-*d*) δ 170.2, 169.8, 169.7, 138.9, 87.8, 80.3, 72.9, 70.2, 70.2, 69.5, 63.1, 50.8, 43.7, 20.9, 20.6, 20.5.

HR-MS (ESI): m/z calcd. for M-H$^+$ $[C_{19}H_{26}N_7O_{11}S]^-$ 560.1416, found: 560.1421.

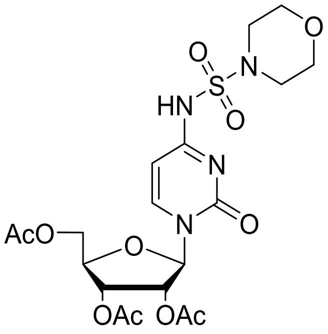

(2R,3R,4R,5R)-2-(acetoxymethyl)-5-(4-(morpholine-4-sulfonamido)-2-oxopyrimidin-1(2H)-yl)tetrahydrofuran-3,4-diyl diacetate (6e):

Obtained starting from **3** (113 mg, 0.25 mmol), morpholine (35 ul, 0.3 mmol), $Ca(NTf_2)_2$ (180 mg, 0.30 mmol) and DABCO (42mg, 0.38 mmol). White solid, yield: 56 mg (43%).

^{1}H NMR (500 MHz, Chloroform-*d*) δ 7.40 (d, *J* = 8.0 Hz, 1H), 5.96 (d, *J* = 5.0 Hz, 1H), 5.81 (bs, 1H), 5.38 – 5.25 (m, 2H), 4.42 – 4.27 (m, 3H), 3.82 – 3.73 (m, 4H), 3.23 – 3.11 (m, 4H), 2.13 (s, 3H), 2.11 (s, 3H), 2.09 (s, 3H).

^{13}C NMR (126 MHz, Chloroform-*d*) δ 170.2, 169.7, 169.7, 155.2, 147.3, 139.0, 103.1, 88.0, 80.2, 72.9, 70.1, 65.9, 63.0, 46.6, 20.9, 20.6, 20.5.

HR-MS (ESI): m/z calcd. for M-H$^+$ $[C_{19}H_{25}N_4O_{11}S]^-$ 517.1246, found: 517.1251.

General procedure 3 deprotection of SulfamNucs **4-6**

This reaction was performed under argon atmosphere. To a sample of acetylated nucleoside a 33% methylamine in ethanol was added (0.1 ml of the solution per each 10 mg of the nucleoside). The reaction mixture was stirred under argon atmosphere until TLC analysis indicated full consumption of starting material (2-4h). The solution was then concentrated under reduced pressure. The oily residue was dissolved in minimal amount of MeOH and the resulting solution was added dropwise to the cold Et_2O (approx. 1 ml per each 10 mg of the starting material) upon stirring. The suspension was centrifuged, washed with ether and dried firstly under well ventilated fumehood and then under high vacuum.

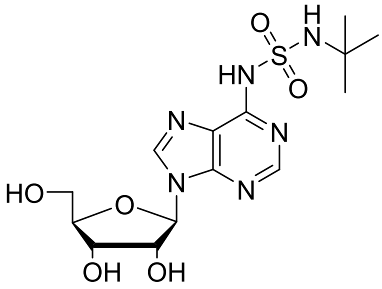

***N*-9-[(2*R*,3*R*,4*S*,5*R*)-3,4-dihydroxy-5-(hydroxymethyl)tetrahydrofuran-2-yl]-9*H*-purin-6-yl-*N*'-methyl sulfuric diamide (7a):**

Obtained starting from **4c** (53 mg, 0.1 mmol). White solid, 38 mg (95%).

Note: this compound was precipitated with MeCN

^{1}H NMR (500 MHz, DMSO-d_6) δ 8.38 (s, 1H), 8.16 (s, 1H), 5.88 (d, *J* = 6.0 Hz, 1H), 5.47 (bs, 2H), 5.17 (d, *J* = 4.5 Hz, 1H), 4.69 – 4.47 (m, 1H), 4.18 – 4.11 (m, 1H), 3.99 – 3.93 (m, 1H), 3.70 – 3.63 (m, 1H), 3.59 – 3.52 (m, 1H), 1.10 (bs, 9H).

^{13}C NMR (126 MHz, DMSO-d_6) δ 88.0, 85.9, 73.5, 70.6, 61.6, 52.0, 29.7.

HR-MS (ESI): m/z calcd. for M-H$^+$ $[C_{14}H_{21}N_6O_6S]^-$ 401.1249, found: 401.1251.

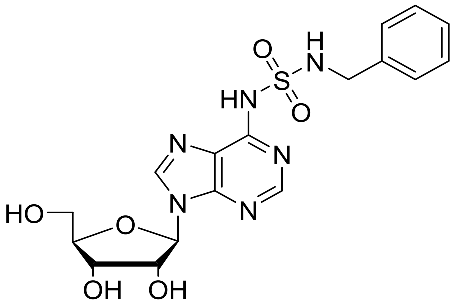

***N*-benzyl-*N*'-9-[(2*R*,3*R*,4*S*,5*R*)-3,4-dihydroxy-5-(hydroxymethyl)tetrahydrofuran-2-yl]-9*H*-purin-6-yl sulfuric diamide (7b):**

Obtained starting from **4f** (56 mg, 0.1 mmol). White solid, 43 mg (98%).

Note: this compound was precipitated with MeCN

^{1}H NMR (500 MHz, DMSO-d_6) δ 8.32 (s, 1H), 8.15 (s, 1H), 7.43 – 7.00 (m, 5H), 5.87 (d, *J* = 6.0 Hz, 1H), 5.46 (bs, 2H), 5.17 (bs, 1H), 4.60 (bs, 1H), 4.14 (bs, 1H), 3.99 – 3.93 (m, 1H), 3.88 (bs, 2H), 3.71 – 3.63 (m, 1H), 3.60 – 3.51 (m, 1H).

^{13}C NMR (126 MHz, DMSO-d_6) δ 139.4, 138.8, 128.2, 127.7, 126.8, 88.0, 85.9, 73.5, 70.6, 61.6, 47.0.

HR-MS (ESI): m/z calcd. for M-H$^+$ $[C_{17}H_{19}N_6O_6S]^-$ 435.1092, found: 435.1095.

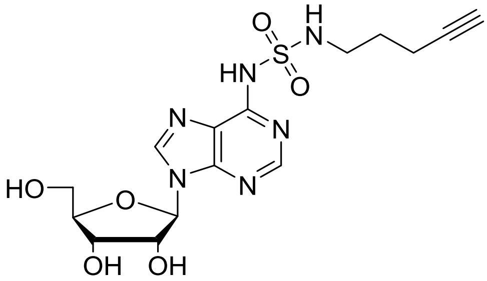

***N*-9-[(2*R*,3*R*,4*S*,5*R*)-3,4-dihydroxy-5-(hydroxymethyl)tetrahydrofuran-2-yl]-9*H*-purin-6-yl-*N*'-pent-4-yn-1-ylsulfuric diamide (7c):**

Obtained starting from **4h** (538 mg, 1.0 mmol). White solid, 383 mg (93%).

^{1}H NMR (500 MHz, DMSO-d_6) δ 8.41 (s, 1H), 8.27 (s, 1H), 5.90 (d, *J* = 6.0 Hz, 1H), 5.48 (d, *J* = 6.1 Hz, 1H), 5.38 (bs, 1H), 5.19 (d, *J* = 4.7 Hz, 1H), 4.62 – 4.55 (m, 1H), 4.17 – 4.11 (m, 1H), 4.00 – 3.92 (m, 1H), 3.71 – 3.63 (m, 1H), 3.61 – 3.52 (m, 1H), 2.87 (t, *J* = 7.0 Hz, 2H), 2.69 (t, *J* = 2.6 Hz, 1H), 2.15 (td, *J* = 7.1, 2.7 Hz, 2H), 1.66 – 1.52 (m, 2H).

^{13}C NMR (126 MHz, DMSO-d_6) δ 150.1, 140.2, 87.8, 85.8, 83.9, 73.6, 71.3, 70.5, 61.5, 42.1, 28.0, 15.3.

HR-MS (ESI): m/z calcd. for M-H$^+$ $[C_{15}H_{19}N_6O_6S]^-$ 511.1092, found: 411.1095.

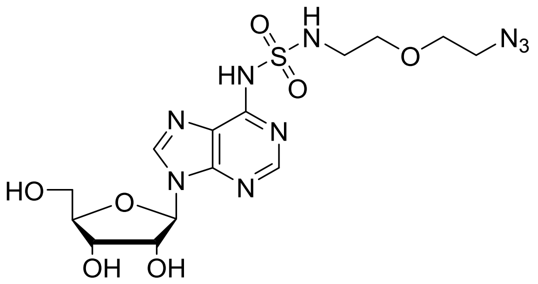

***N*-9-[(2*R*,3*R*,4*S*,5*R*)-3,4-dihydroxy-5-(hydroxymethyl)tetrahydrofuran-2-yl]-9*H*-purin-6-yl-*N*'-(2-(2-azidoethoxy)ethyl sulfuric diamide (7d):**

Obtained starting from **4i** (585 mg, 1.0 mmol). White solid, 418 mg (91%).

^{1}H NMR (500 MHz, DMSO-d_6) δ 8.20 (s, 1H), 8.10 (s, 1H), 5.84 (d, *J* = 6.2 Hz, 1H), 5.61 (bs, 1H), 5.41 (bs, 1H), 5.14 (bs, 1H), 4.68 – 4.53 (m, 1H), 4.13 (dd, *J* = 5.0, 2.9 Hz, 1H), 4.00 – 3.91 (m, 1H), 3.67 (dd, *J* = 12.1, 3.4 Hz, 1H), 3.54 (d, *J* = 12.9 Hz, 1H), 3.46 (t, *J* = 5.6 Hz, 2H), 3.43 (t, *J* = 5.0 Hz, 2H), 3.30 (t, *J* = 4.9 Hz, 2H), 2.87 (t, *J* = 5.6 Hz, 2H).
^{13}C NMR (126 MHz, DMSO-d_6) δ 151.2, 148.6, 139.0, 88.1, 85.9, 73.4, 70.8, 69.4, 68.9, 64.9, 50.0, 48.6, 42.9, 24.6.

HR-MS (ESI): m/z calcd. for M-H$^+$ [$C_{14}H_{20}N_9O_7S$]$^-$ 458.1212, found: 458.1217.

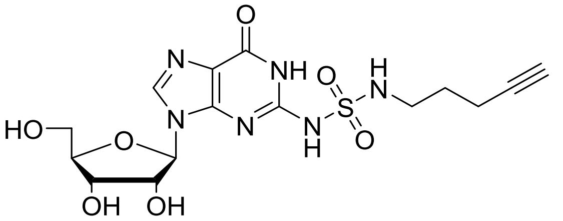

***N*-9-[(2*R*,3*R*,4*S*,5*R*)-3,4-dihydroxy-5-(hydroxymethyl)tetrahydrofuran-2-yl]-6-oxo-6,9-dihydro-1*H*-purin-2-yl-*N*'-pent-4-yn-1-ylsulfuric diamide (8a):**

Obtained starting from **5d** (55 mg, 0.1 mmol). White solid, 41 mg (95%).

^{1}H NMR (500 MHz, DMSO-d_6) δ 10.20 (bs, 1H), 7.82 (s, 1H), 5.75 (t, *J* = 6.6 Hz, 1H), 5.69 (d, *J* = 6.0 Hz, 1H), 5.36 (d, *J* = 6.5 Hz, 1H), 5.16 (d, *J* = 5.1 Hz, 1H), 5.06 (t, *J* = 5.5 Hz, 1H), 4.40 – 4.33 (m, 1H), 4.10 – 4.03 (m, 1H), 3.90 – 3.85 (m, 1H), 3.63 – 3.57 (m, 1H), 3.55 – 3.48 (m, 2H), 2.79 – 2.74 (m, 2H), 2.68 (t, *J* = 2.6 Hz, 1H), 2.14 (td, *J* = 7.2, 2.7 Hz, 2H), 1.64 – 1.53 (m, 2H).

^{13}C NMR (126 MHz, DMSO-d_6) δ 158.0, 154.7, 151.5, 134.8, 116.6, 86.6, 85.3, 84.2, 74.0, 71.1, 70.4, 61.5, 42.3, 28.2, 15.5.

HR-MS (ESI): m/z calcd. for M-H$^+$ [$C_{15}H_{19}N_6O_7S$]$^-$ 427.1041, found: 427.1044.

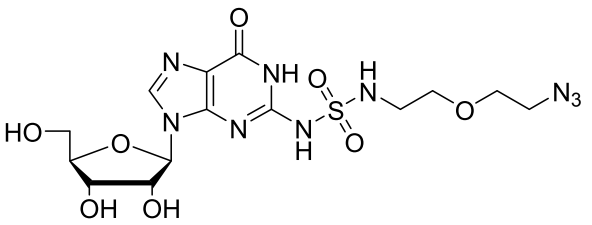

N-9-[(2R,3R,4S,5R)-3,4-dihydroxy-5-(hydroxymethyl)tetrahydrofuran-2-yl]-6-oxo-6,9-dihydro-1H-purin-2-yl-N'-(2-(2-azidoethoxy)ethylsulfuric diamide (8b):

Obtained starting from **5c** (60 mg, 0.1 mmol). White solid, 42 mg (90%).

^{1}H NMR (500 MHz, DMSO-d_6) δ 10.20 (bs, 1H), 7.83 (s, 1H), 5.80 (t, J = 6.5 Hz, 1H), 5.70 (d, J = 5.8 Hz, 1H), 5.35 (d, J = 6.3 Hz, 1H), 5.15 (d, J = 5.0 Hz, 1H), 5.04 (t, J = 5.5 Hz, 1H), 4.40 – 4.33 (m, 1H), 4.11 – 4.06 (m, 1H), 3.91 – 3.85 (m, 1H), 3.65 – 3.57 (m, 1H), 3.56 – 3.45 (m, 5H), 3.32 – 3.29 (m, 2H), 2.91 – 2.83 (m, 2H).

^{13}C NMR (126 MHz, DMSO-d_6) δ 158.0, 154.8, 151.4, 134.8, 116.7, 86.6, 85.2, 74.2, 70.4, 69.3, 69.0, 61.5, 49.8, 42.9.

HR-MS (ESI): m/z calcd. for M-H$^+$ $[C_{14}H_{20}N_9O_8S]^-$ 474.1161, found: 474.1161.

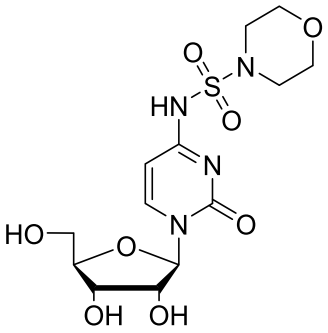

N-1-[(2R,3R,4S,5R)-3,4-dihydroxy-5-(hydroxymethyl)tetrahydrofuran-2-yl]-2-oxo-1,2-dihydropyrimidin-4-yl-piperidine-1-sulfonamide (9):

Obtained starting from **6e** (52 mg, 0.1 mmol). White solid, 38 mg (97%).

^{1}H NMR (500 MHz, DMSO-d_6) δ 7.83 (d, J = 7.8 Hz, 1H), 6.10 (d, J = 7.8 Hz, 1H), 5.75 (d, J = 4.5 Hz, 1H), 4.02 – 3.96 (m, 1H), 3.97 – 3.91 (m, 1H), 3.85 – 3.79 (m, 1H), 3.65 (dd, J = 12.1, 3.2 Hz, 1H), 3.62 – 3.58 (m, 4H), 3.54 (dd, J = 12.1, 3.5 Hz, 1H), 2.98 – 2.92 (m, 4H).

^{13}C NMR (126 MHz, DMSO-d_6) δ 140.2, 98.5, 88.9, 84.3, 73.9, 69.5, 65.5, 60.6, 48.6, 46.5.

HR-MS (ESI): m/z calcd. for M-H$^+$ $[C_{13}H_{19}N_4O_8S]^-$ 391.0929, found: 391.0933.

The synthesis of conjugate **10**

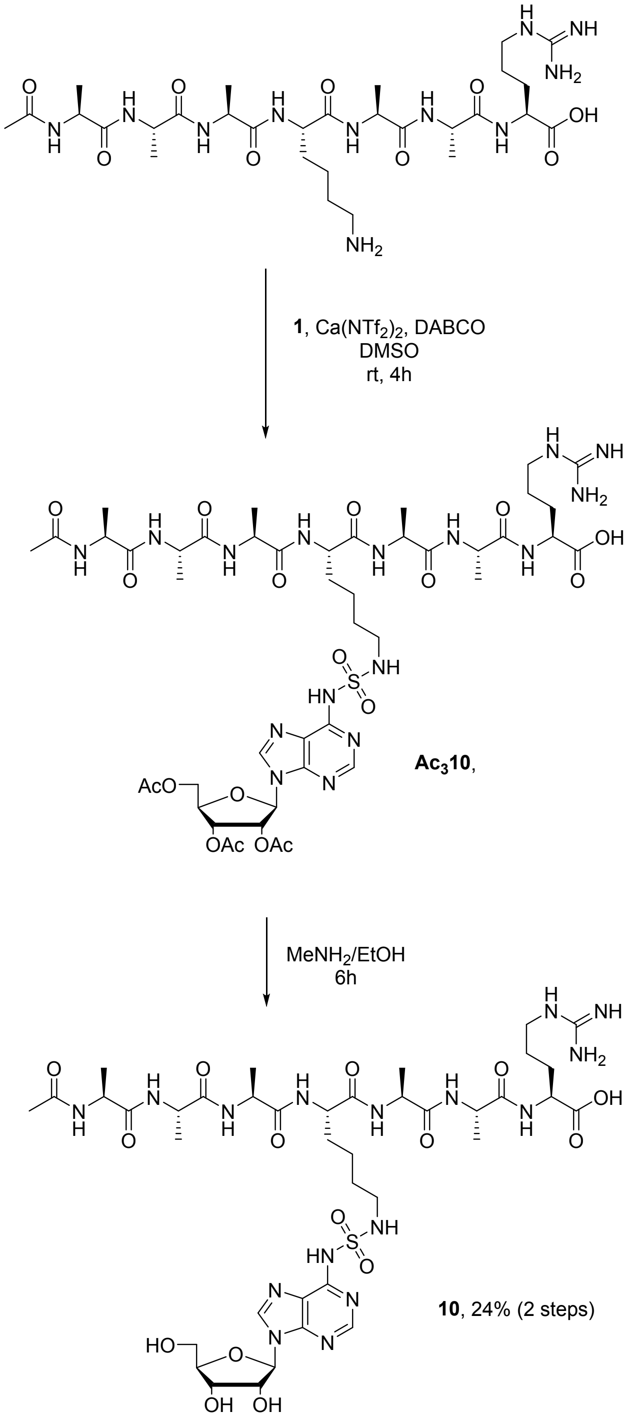

1 (1.5 mg, 4.2 µmol), $Ca(NTf_2)_2$ (5.0 mg, 8.4 µmol) and DABCO (2.3 mg, 20.5 µmol) were dissolved in DMSO (100 µl) followed by addition of the solution of peptide (TFA salt, 1.0 mg, 1.2 µmol in 100 µl DMSO). The resulting mixture was vigorously shaken at room temperature until HPLC analysis indicated no further progress of the reaction (approx. 4h). The reaction mixture was added to Et_2O (5 ml), the resulting suspension centrifuged, washed twice with Et_2O (2 ml) and dried. The resulting solid was dissolved in EDTA solution (3.0 mg, 10 µmol in 500 µl H_2O). The obtained solution was centrifuged,

loaded on semi preparative HPLC column and eluted with linear gradient of MeCN (+ 0.05% TFA) in water (+0.05% TFA). Fractions containing product were collected and freeze dried.

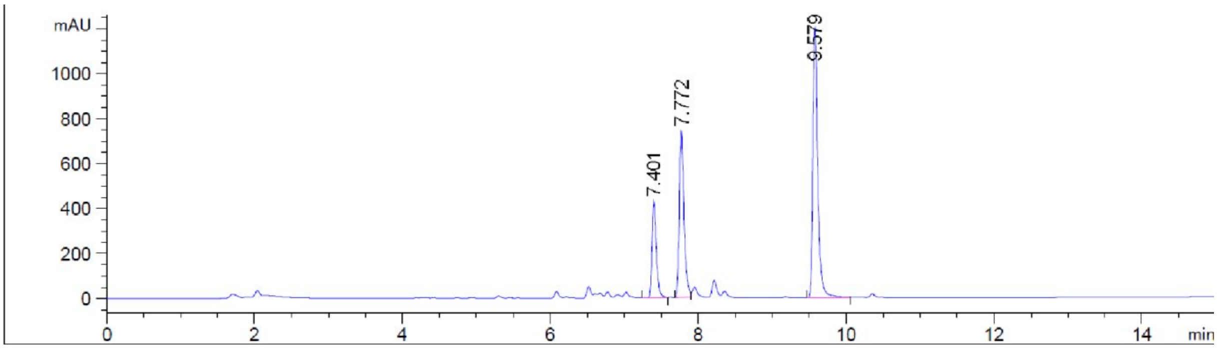

HPLC analysis of the reaction mixture (program A). Signal at t = 7.40 min corresponds to **Ac$_3$10**, signal at t = 9.58 min corresponds to **1**, signal at t = 7.77 corresponds to unidentified **1** derivative [possibly -NSO$_2$-F group was hydrolysed to NSO$_3^-$ (LRMS m/z = 473.1), but this compound was not isolated and fully characterised] .

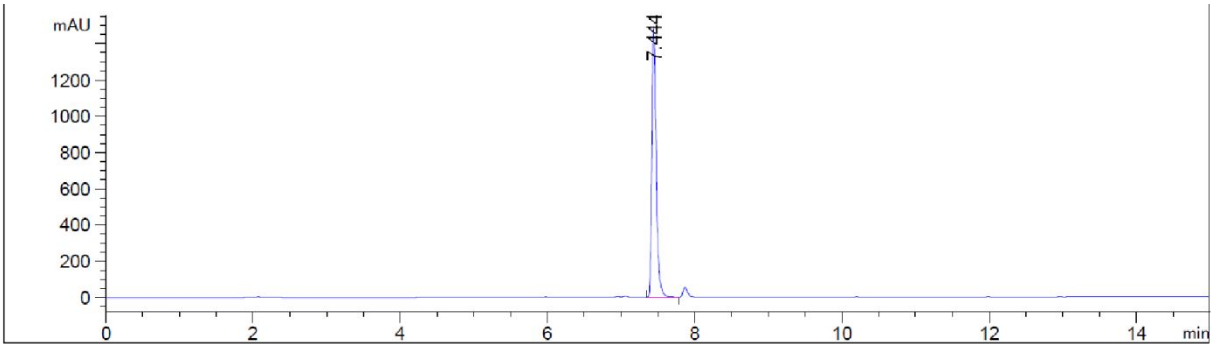

HPLC analysis of the purified **Ac$_3$10**.

HR-MS (ESI): m/z calcd. for M+H$^+$ [$C_{45}H_{71}N_{16}O_{18}S$]$^+$ 1155.4847, found: 1155.4830.

The resulting conjugate was dissolved in 33% methylamine in ethanol (100 µl) under argon atmosphere and was vigorously shaken at room temperature for 6 hours. Then the reaction mixture was concentrated under reduced pressure. The resulting oil was dissolved in 0.05% TFA in water (150 µl), loaded on semi preparative HPLC column and eluted with linear gradients of MeCN (+ 0.05% TFA) in water (+0.05% TFA). Fractions containing product were collected and freeze dried. After repeated freeze drying product **10** was obtained as white foam, 0.3 mg, 24% (two steps).

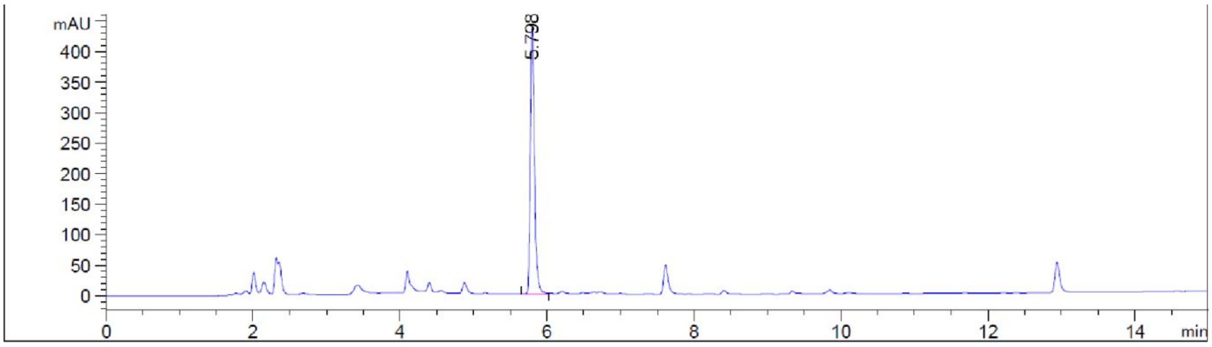

HPLC analysis of the reaction mixture (program A). Signal at t 5.79 min corresponds to **10**.

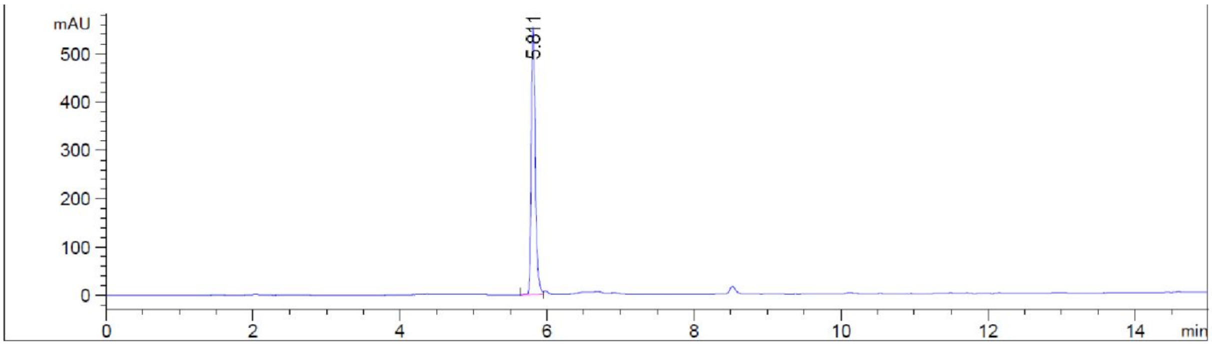

HPLC analysis of the purified conjugate **10**.

HR-MS (ESI): m/z calcd. for M+H⁺ $[C_{39}H_{65}N_{16}O_{15}S]^+$ 1029.4531, found: 1029.1509.

<u>Note:</u> The reaction conditions and workup/purification procedures were not optimised.

The synthesis of nucleotide **11**

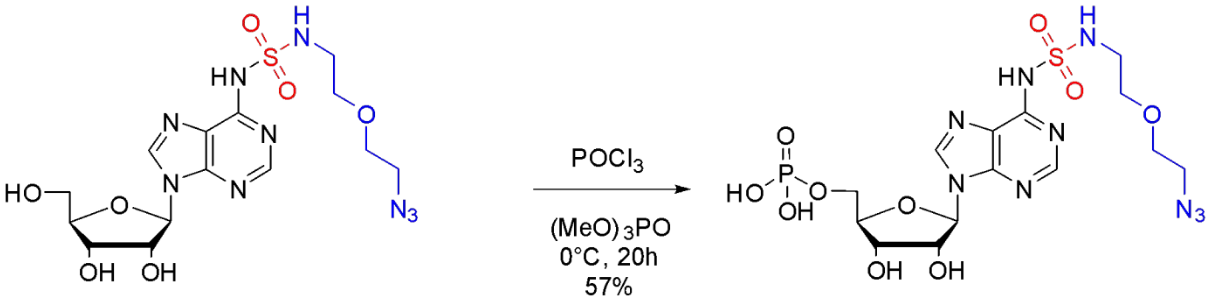

7d (115 mg, 0.25 mmol) was dissolved in anhydrous $(MeO)_3PO$ (2.5 ml) under gentle flow of argon. The resulting solution was cooled below 0°C (ice/brine bath) followed by dropwise addition of freshly distilled $POCl_3$ (70 µl, 0.75 mmol). The reaction mixture was stirred below 0°C for approx. 20 h after which RP HPLC analysis indicated full consumption of the starting material. The reaction mixture was poured into cold, deionised water (approx. 20 ml) and neutralised with 10% $NaHCO_3$. The resulting mixture was loaded on DEAE Sephadex A-25 column (HCO^{3-} form), the column was washed thoroughly with water and then eluted using TEAB in deionized water (0 to 0.7 M linear gradient). The fractions containing product (UV and RP HPLC analysis) were combined, concentrated *in vacuo*, co-evaporated with 96% EtOH (approx. 50 ml), co-evaporated with MeCN (approx. 50 ml). The residue was dissolved in MQ water and freeze-dried. The product was purified using semi-preparative HPLC with linear gradient of MeCN (+ 0.05% TFA) in water (+0.05% TFA). The fractions containing pure product were concentrated *in vacuo*, dissolved in MQ water (approx. 10 ml) and freeze-dried. After repeated freeze drying **11** was obtained as white solid (77 mg, 57%).

((2R,3S,4R,5R)-5-(6-((N-(2-(2-azidoethoxy)ethyl)sulfamoyl)amino)-9H-purin-9-yl)-3,4-dihydroxytetrahydrofuran-2-yl)methyl dihydrogen phosphate (11):

^{1}H NMR (500 MHz, Deuterium Oxide) δ 8.60 (s, 1H), 8.44 (s, 1H), 6.20 (d, *J* = 5.7 Hz, 1H), 4.77 (m 1H, overlapped with water), 4.51 (dd, *J* = 5.1, 3.7 Hz, 1H), 4.42 – 4.37 (m, 1H), 4.20 – 4.03 (m, 2H), 3.66 – 3.61 (m, 2H), 3.52 – 3.46 (m, 2H), 3.32 – 3.24 (m, 4H).

^{13}C NMR (126 MHz, Deuterium Oxide) δ 153.3, 151.9, 150.4, 143.7, 124.8, 90.1, 86.9 (d, *J* = 8.7 Hz) 77.2, 73.1, 71.6, 71.3, 67.0 (d, *J* = 5.0 Hz), 52.8, 45.5.

^{31}P NMR (203 MHz, Deuterium Oxide) δ 0.5.

HR-MS (ESI): m/z calcd. for $M-H^+$ $[C_{14}H_{21}N_9O_{10}PS]^-$ 538.0875, found: 538.0875.

The synthesis of **16**

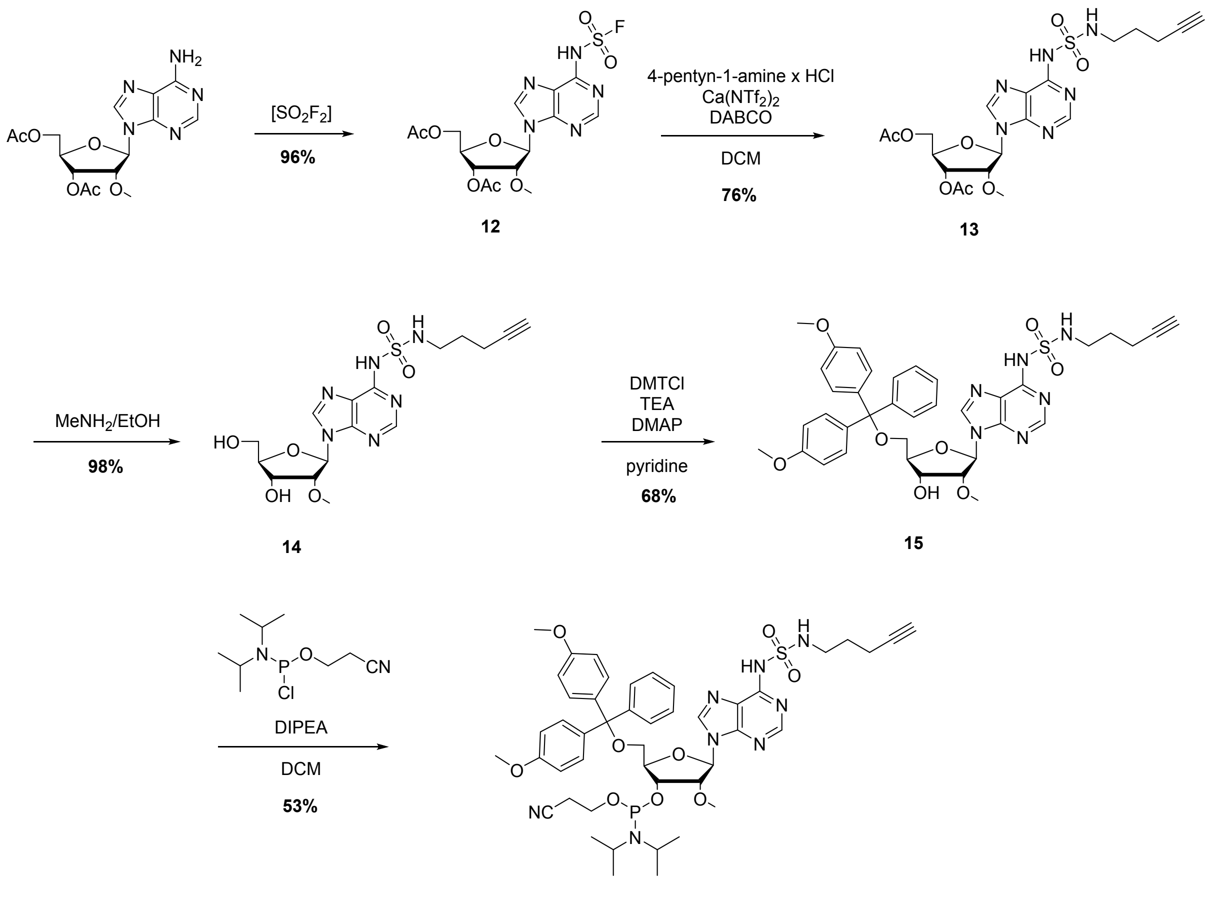

((2R,3R,4R,5R)-3-acetoxy-5-(6-((fluorosulfonyl)amino)-9H-purin-9-yl)-4-methoxytetrahydrofuran-2-yl)methyl acetate (12):

Obtained according to General Procedure 1 from **2'OMe Ac₂A** (1.10 g, 3.0 mmol).

White solid, yield: 1.29 g (96%).

^{1}H NMR (500 MHz, Chloroform-d) δ 8.67 (s, 1H), 8.44 (s, 1H), 6.16 (d, J = 5.3 Hz, 1H), 5.35 (t, J = 4.7 Hz, 1H), 4.53 (t, J = 5.3 Hz, 1H), 4.51 – 4.45 (m, 1H), 4.43 – 4.36 (m, 2H), 3.43 (s, 3H), 2.19 (s, 3H), 2.15 (s, 3H).

^{13}C NMR (126 MHz, Chloroform-*d*) δ 170.6, 170.2, 151.4, 150.4, 145.3, 142.4, 123.8, 87.4, 82.0, 80.9, 70.6, 63.1, 59.5, 21.0, 20.8.

^{19}F NMR (471 MHz, Chloroform-*d*) δ 53.6.

HR-MS (ESI): m/z calcd. for M-H$^+$ $[C_{15}H_{17}FN_5O_8S]^-$ 446.0787, found: 446.0799.

((2R,3R,4R,5R)-3-acetoxy-4-methoxy-5-(6-((N-(pent-4-yn-1-yl)sulfamoyl)amino)-9H-purin-9-yl)tetrahydrofuran-2-yl)methyl acetate (13):

Obtained according to General Procedure 2 from **12** (1.12 g, 2.5 mmol) and 4-pentyn-1-amine hydrochloride (358 mg, 3.0 mmol).

White foam, yield: 969 mg (76%).

^{1}H NMR (500 MHz, Chloroform-*d*) δ 8.75 (s, 1H), 8.32 (s, 1H), 6.13 (d, *J* = 5.1 Hz, 1H), 5.36 (t, *J* = 4.8 Hz, 1H), 4.64 (t, *J* = 5.1 Hz, 1H), 4.49 – 4.35 (m, 3H), 3.43 (s, 3H), 3.22 (t, *J* = 6.8 Hz, 2H), 2.29 (td, *J* = 6.9, 2.7 Hz, 2H), 2.18 (s, 3H), 2.15 (s, 3H), 1.90 (t, *J* = 2.6 Hz, 1H), 1.84 – 1.77 (m, 2H).

^{13}C NMR (126 MHz, Chloroform-*d*) δ 170.6, 170.2, 151.1, 150.6, 150.3, 141.3, 121.1, 87.6, 82.9, 81.6, 80.6, 70.6, 69.4, 63.0, 59.5, 42.7, 28.1, 21.0, 20.8, 15.9.

HR-MS (ESI): m/z calcd. for M-H$^+$ $[C_{20}H_{25}N_6O_8S]^-$ 509.1460, found: 509.1462.

***N*-9-[(2*R*,3*R*,4*S*,5*R*)-3-hydroxy-4-methoxy--5-(hydroxymethyl)tetrahydrofuran-2-yl]-9*H*-purin-6-yl-*N*'-pent-4-yn-1-ylsulfuric diamide (14):**

Obtained according to General Procedure 3 from **13** (765 mg, 1.5 mmol).

White solid, yield: 630 mg (98%).

^{1}H NMR (500 MHz, DMSO-d_6) δ 8.32 (s, 1H), 8.18 (s, 1H), 5.99 (d, *J* = 6.1 Hz, 1H), 5.55 (bs, 1H), 5.26 (bs, 1H), 4.40 – 4.34 (m, 1H), 4.36 – 4.31 (m, 1H), 4.01 – 3.95 (m, 1H),3.30 (s, 3H), 3.67 (dd, *J* = 12.1, 3.6 Hz, 1H), 3.56 (dd, *J* = 12.0, 3.7 Hz, 1H), 2.82 (tt, *J* = 6.8, 3.4 Hz, 2H), 2.68 (t, *J* = 2.6 Hz, 1H),, 2.14 (td, *J* = 7.1, 2.7 Hz, 2H), 1.68 – 1.45 (m, 2H).

^{13}C NMR (126 MHz, DMSO-d_6) δ 151.2, 148.7, 139.2, 123.2, 86.5, 85.9, 84.0, 82.5, 71.2, 68.8, 64.9, 57.5, 42.2, 28.2, 24.6, 15.4.

HR-MS (ESI): m/z calcd. for M-H$^+$ $[C_{16}H_{21}N_6O_6S]^-$ 425.1249, found: 425.1253.

***N*-9-[(2*R*,3*R*,4*S*,5*R*)-3-hydroxy-4-methoxy-5-((bis(4-methoxyphenyl)(phenyl)methoxy)methyl)tetrahydrofuran-2-yl]-9*H*-purin-6-yl-*N*'-pent-4-yn-1-ylsulfuric diamide (15)**:

<u>**Note**</u>: *prior the reaction **14** and 4,4'-Dimethoxytrityl chloride were dried overnight under high vacuum in the desiccator over P_2O_5. The reaction was performed in strictly anhydrous conditions using Schlenk techniques. From authors experience, derogation from this regime (for example by using septum/balloon setups) resulted in drastic drop in selectivity and yield of the reaction.*

14 (426 mg, 1.0 mmol) was dissolved in anhydrous pyridine (5 ml) followed by addition of anhydrous trimethylamine (720 µl, 10.0 mmol) and DMAP (12 mg, 0.1 mmol). To the resulting solution 4,4'-dimethoxytrityl chloride (608 mg, 1.8 mmol) was added. The reaction mixture was stirred until TLC analysis (3% MeOH in DCM) indicated full consumption of the starting material (approx. 4h), cooled down to 0°C and quenched by dropwise addition of 5% $NaHCO_3$ solution (approx. 5 ml). The reaction mixture was transferred to the separatory funnel, diluted with 50 ml of water and extracted wit AcOEt (3x 50 ml). The combined organic phases were washed with brine (100 ml), dried over Na_2SO_4, filtered and concentrated under reduced pressure. The resulting oil was co-evaporated twice with EtOH and

the oily residue was subjected to the high vacuum until it turned into brittle foam. The product was purified using column chromatography (SiO_2, 0 to 3% MeOH in DCM + 0.5% triethylamine). Combined fractions containing pure product were concentrated under reduced pressure followed by evaporation with Et_2O (approx. 10 ml) and drying under high vacuum.

White foam, 500 mg (68%).

^{1}H NMR (500 MHz, DMSO-d_6) δ 8.16 (s, 1H), 8.11 (s, 1H), 7.41 – 7.34 (m, 2H), 7.30 – 7.17 (m, 7H), 6.94 – 6.76 (m, 4H), 6.36 (bs, 1H), 6.02 (d, *J* = 4.2 Hz, 1H), 5.27 (bs, 1H), 4.40 (d, *J* = 3.7 Hz, 2H), 4.04 (q, *J* = 4.6 Hz, 1H), 3.73 (s, 6H), 3.36 (s, 3H), 3.24 (d, *J* = 4.7 Hz, 2H), 2.85 – 2.73 (m, 2H overlapping with TEA), 2.68 (t, *J* = 2.7 Hz, 1H), 2.13 (td, *J* = 7.1, 2.7 Hz, 2H), 1.64 – 1.51 (m, 2H).

^{13}C NMR (126 MHz, DMSO-d_6) δ 158.1, 151.6, 148.9, 144.8, 138.4, 135.6, 135.4, 129.7, 129.7, 127.8, 127.7, 126.6, 123.1, 113.1, 85.6, 85.5, 84.0, 83.3, 82.0, 71.2, 69.0, 63.6, 57.7, 55.0, 42.1, 28.3, 15.4.

HR-MS (ESI): m/z calcd. for M+H$^+$ $[C_{37}H_{41}N_6O_8S]^+$ 729.2701, found: 729.2685.

(2R,3R,4R,5R)-2-((bis(4-methoxyphenyl)(phenyl)methoxy)methyl)-4-methoxy-5-(6-((N-(pent-4-yn-1-yl)sulfamoyl)amino)-9H-purin-9-yl)tetrahydrofuran-3-yl(2-cyanoethyl) diisopropylphosphoramidite (16):

14 (240 mg, 0.33 mmol) was dissolved in anhydrous DCM (5 ml) under argon atmosphere followed by addition of anhydrous N,N-diisopropylethylamine (175 µl, 1.00 mmol). The resulting solution was cooled down to 0°C (ice bath) and 2-cyanoethyl N,N-diisopropylchlorophosphoramidite (118 µl, 0.50 mmol) was added dropwise under gentle flow of argon. The ice bath was removed and the reaction mixture was stirred at room temperature until TLC analysis (3% MeOH in DCM) indicated full conversion (approx. 2h). The solvent was evaporated and the resulting solid was dissolved in ethyl acetate (20 ml) and washed with 5% $NaHCO_3$ solution (2 x 20 ml) and with brine (20 ml). The organic layer was dried over Na_2SO_4, filtered and concentrated under reduced pressure. The product was purified using column chromatography (SiO_2, 0 to 100% ethyl acetate in hexane + 1% triethylamine). Combined fractions containing pure product were concentrated under reduced pressure followed by evaporation with Et_2O (approx. 10 ml) and drying under high vacuum. **16** was obtained as a mixture of diastereoisomers in approx. 4:6 ratio (based on ^{31}P NMR).

White foam, 163 mg (53%).

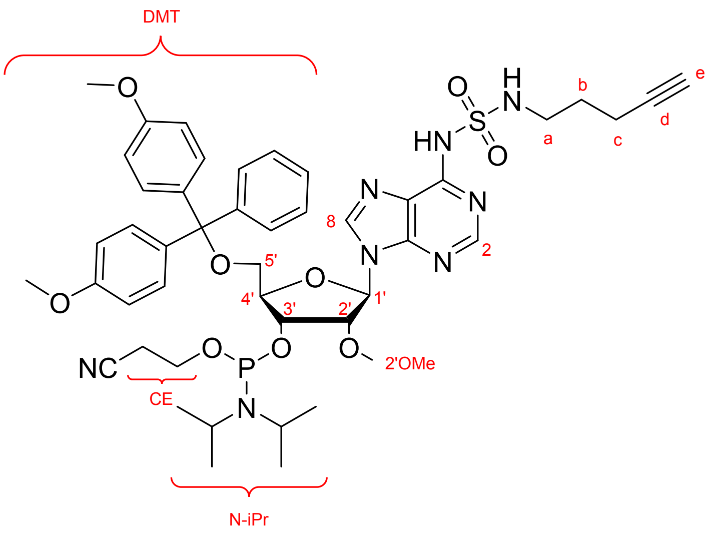

^{1}H NMR (500 MHz, Chloroform-*d*) δ 8.66, 8.65 (2 x s, 1H, C2-H), 8.32, 8.52 (2 x s, 1H, C8-H), 7.48 – 7.38 (m, 2H, DMT), 7.36 – 7.20 (m, 7H, DMT), 6.92 – 6.77 (m, 4H, DMT), 6.21 – 6.10 (m, 1H. C1'-H), 4.71 – 4.63 and 4.63 – 4.55 (2 x m, 1H, C3'-H), 4.51 – 4.44 (m, 0.6H, C2'-H), 4.44 – 4.37 (m, 0.4H C2'-H, 0.6H, C4'-H), 4.37 – 4.33 (m, 0.4H, C4'-H) 3.96 – 3.81 (m, 1H, 1x ^{CE}1-H), 3.81, 3.3.80 (2 x s, 6H, 2x DMT-OMe), 3.73 – 3.53 (m, 4H, 1x ^{CE}1 –H, 2 x $^{N\text{-}iPr}$CH, 1 x C5'-H), 3.52, 3.51 (2 x s. 3H. 2'OMe), 3.44 – 3.35 (m, 1H, 1 x C5'-H), 3.24 – 3.10 (m, 2H, ^{a}CH$_2$), 2.64 (td, *J* = 6.3, 2.4 Hz, 1H, ^{CE}2 –H), 2.39 (t, *J* = 6.3 Hz, 1H, ^{CE}2 –H), 2.26 – 2.17 (m, 2H, ^{c}CH$_2$), 1.85 (t, *J* = 2.6 Hz, 1H, ^{e}C-H), 1.78 – 1.70 (m, 2H, ^{b}CH$_2$), 1.23 – 1.12 (m, 9H, 3 x $^{N\text{-}iPr}$CH$_3$, overlapping with Et$_2$O), 1.07 (d, *J* = 6.7 Hz, 3H, 1 x $^{N\text{-}iPr}$CH$_3$).

^{13}C NMR (126 MHz, Chloroform-d) δ 158.8, 150.6, 144.5, 144.4, 141.2, 141.1, 135.7, 135.6, 135.6, 135.6, 130.3, 130.3, 128.5, 128.4, 128.1, 127.2, 121.6, 117.8, 117.5, 113.3, 87.2, 87.0, 86.9, 83.8, 83.1, 83.1, 83.0, 82.6, 69.3, 69.3, 62.7, 62.2, 43.6, 43.5, 43.4, 43.3, 42.7, 28.2, 28.2, 24.8, 24.7, 24.7, 24.7, 20.5, 20.5, 20.3, 15.9.

^{31}P NMR (203 MHz, Chloroform-*d*) δ 151.1, 150.5.

HR-MS (ESI): m/z calcd. for M+H$^+$ [$C_{46}H_{58}N_8O_9PS$]$^+$ 929.3780, found: 929.3760.

Note 1: Because **16** was obtained as a mixture of diastereoisomers, some of the signals on ^{1}H NMR and ^{13}C NMR spectra are doubled or "split" and spectra are difficult for interpretation. Authors recommend verification of the purity of the compound by recording ^{31}P NMR spectra in wide range to track potential impurities.

Note 2: Because of its limited stability, authors recommend utilisation of **16** for SPS possibly quickly after obtaining. To avoid decomposition **16** should be stored at - 20°C. Nevertheless if stored for longer periods, authors recommend verification of its purity prior SPS (^{31}P NMR).

The synthesis of oligonucleotide **17**

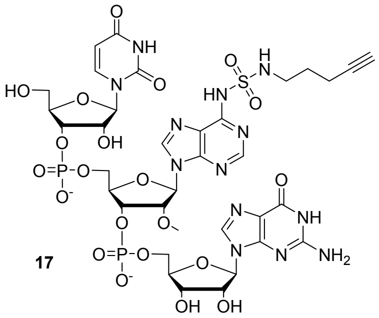

17

Solid-phase synthesis trinucleotide of trinucleotide **17** was performed in a 5 mL syringe equipped with a frit and loaded with 168 mg (52 µmol, 1.0 equiv) of polystyrene support ribo G 300 PrimerSupport™ 5G (308 µmol/g, GE Healthcare). The detritylation step was performed by passing 10 ml of 3% (v/v) trichloroacetic acid in DCM through the column. The solid support was washed with DNA synthesis grade acetonitrile (3x4 ml, <10 ppm of H_2O, Supleco Merck) and dried in a vacuum desiccator. In the coupling step, a solution of 140 mg (151 µmol, 2.9 equiv.) of **16** in 0.50 ml of anhydrous acetonitrile and 0.75 ml of BTT Activator (Sigma Aldrich) were shaken with the support for 30 min. Then the support was washed with acetonitrile (3x4 ml) and the phosphite triester was oxidized by passing 4 ml of 0.05 M iodine in pyridine/water 9:1 (v/v). The solid support was washed with 12 ml of DNA synthesis grade acetonitrile (3x4 ml, <10 ppm of H_2O). The detritylation, coupling and oxidation steps were performed analogously using 113 mg (151 µmol, 2.9 equiv) of 2'-OTBDMS-5'-ODMTr-uridine 3'-CE phosphoramidite (LinkTech). The support was dried in a vacuum desiccator, transferred to a 50 ml polypropylene tube, and the compound was cleaved from the support using 2.5 ml of AMA (1:1 (v/v) mixture of 33% ammonium hydroxide and 40% methylamine in water; 37°C, 3h). The suspension was filtered, washed with water (3x3 ml), evaporated to dryness, redissolved in water, and freeze-dried. The O-TBDMS groups were removed by dissolving the residue in 50 µL of DMSO, followed by the addition of triethylamine (108 µL) and triethylammonium trihydrofluoride (63 µL). The resulting mixture was stirred at 65°C for 3 h. The reaction was quenched by addition of 0.05 M $NaHCO_3$ (7 ml). The product was isolated by ion-exchange chromatography on DEAE Sephadex using a linear gradient of TEAB (0–1.2M). Collected fractions containing the product were evaporated to dryness, redissolved in water, and freeze-dried. The final product was additionally purified by semi-preparative RP HPLC with linear gradient of MeCN in 0.05 M ammonium acetate buffer (pH 5.9). After repeated freeze-drying of the collected fractions, the trinucleotide was isolated as ammonium salt.

White solid (33 mg, 57%).

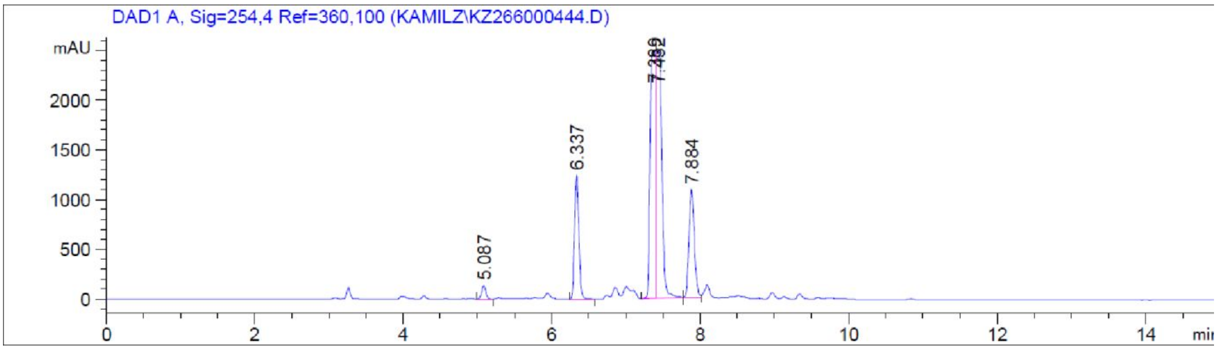

HPLC of reaction mixture upon cleavage of TBDMS groups (program B). Peak at t = 7.28 min. corresponds to **17**.

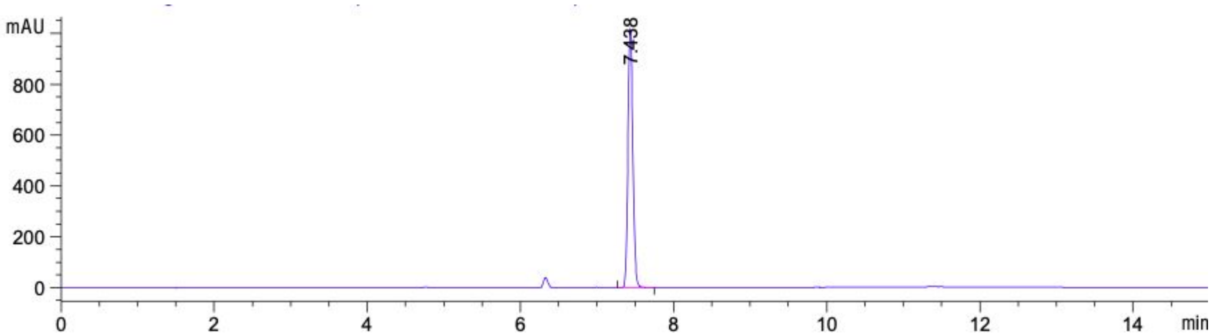

HPLC analysis of the purified trinucleotide **17**.

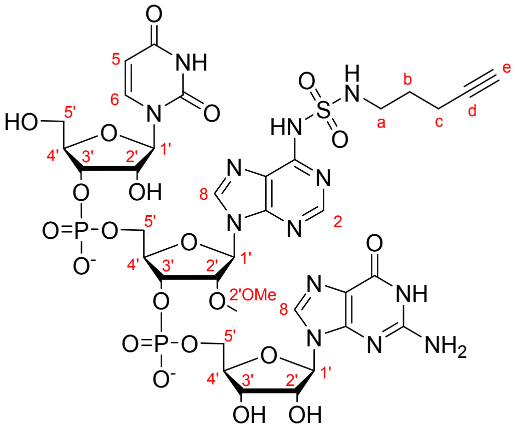

^{1}H NMR (500 MHz, Deuterium Oxide) δ 8.48 (s, 1H, ^{A}C2-H), 8.38 (s, 1H,^{G}C8-H), 8.32 (s, 1H, ^{A}C8-H), 7.74 (d, *J* = 8.1 Hz, 1H, ^{U}C6-H), 6.11 (d, *J* = 5.6 Hz, 1H, ^{A}C1'-H), 5.90 (d, *J* = 4.8 Hz, 1H, ^{U}C1'-H), 5.79 – 5.73 (m, 2H. ^{G}C1'-H and ^{U}C5-H), 5.00 – 4.92 (m, 1H, ^{A}C3'-H), 4.74 – 4.70 (m, 1H, ^{U}C2'-H), 4.53 – 4.43 (m, 4H, ^{A}C2'-H, ^{A}C4'-H, ^{G}C3'-H and ^{U}C3'-H), 4.33 – 4.27 (m, 2H, ^{G}C2'-H and ^{G}C4'-H), 4.23 – 4.14 (m, 2H, ^{G}C5'-H), 4.13 – 4.09 (m, 3H, ^{U}C4'-H, ^{A}C5'-H), 3.67 (d, *J* = 3.4 Hz, 2H, ^{U}C5-H), 3.40 (s, 3H, ^{A}2'OMe), 3.10 (t, *J* = 6.6 Hz, 2H, $^{a}CH_2$), 2.11 (td, *J* = 6.9, 2.7 Hz, 2H, $^{c}CH_2$), 2.03 (t, *J* = 2.7 Hz, 1H, ^{e}CH), 1.69 – 1.59 (m, 2H, $^{b}CH_2$).

^{31}P NMR (203 MHz, Deuterium Oxide) δ -0.9 (2P).
HR-MS (ESI): m/z calcd. for M-H^{+} $[C_{35}H_{44}N_{13}O_{21}P_2S]^{-}$ 1076.1976, found: 1076.1988.

Copies of NMR and HRMS spectra

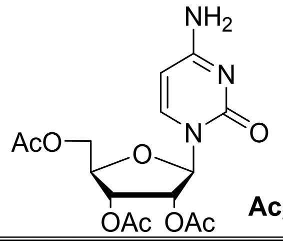

Ac$_3$C

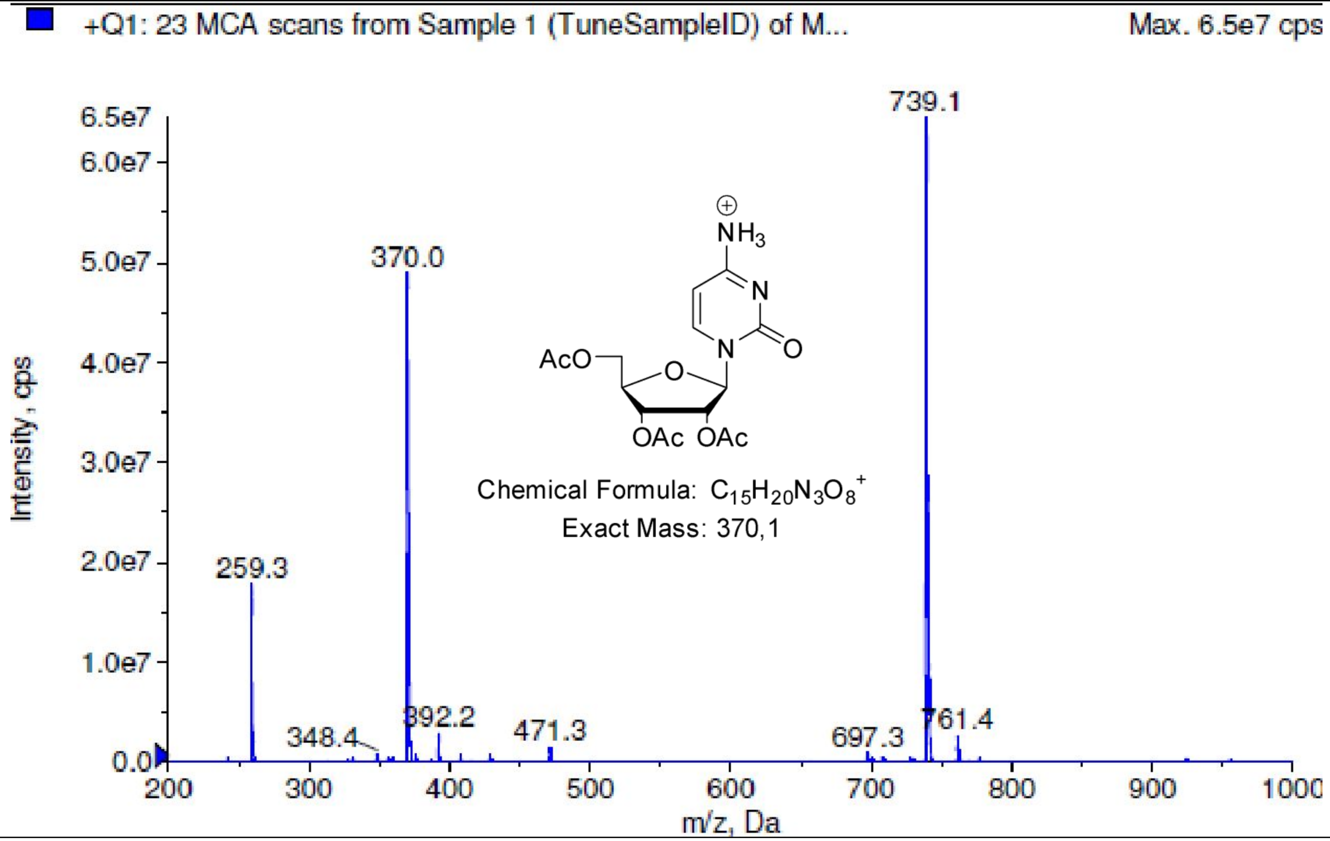

^1H NMR spectrum (500 MHz) of **Ac_3C**

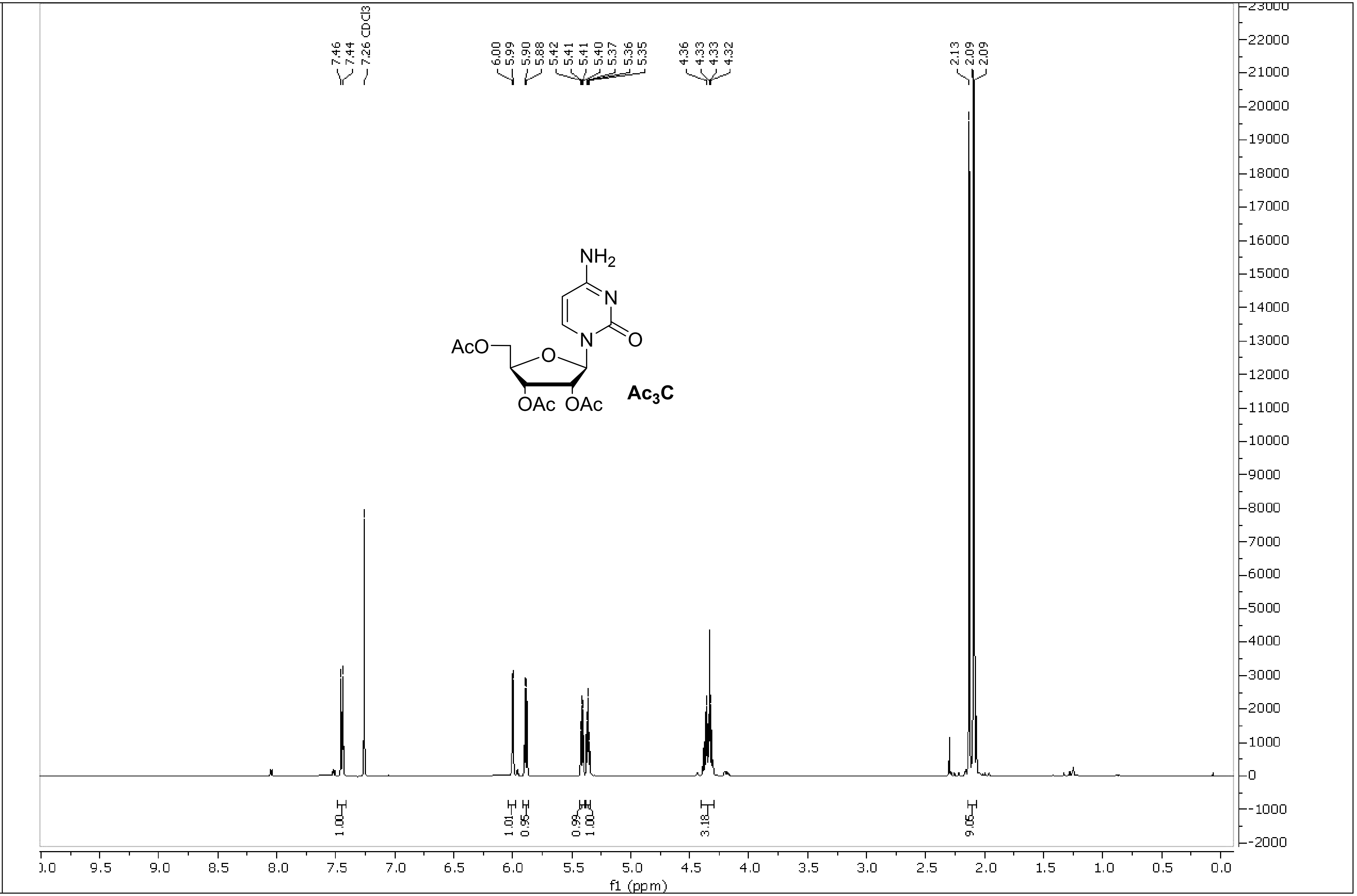

^{13}C NMR spectrum (126 MHz) of **Ac$_3$C**

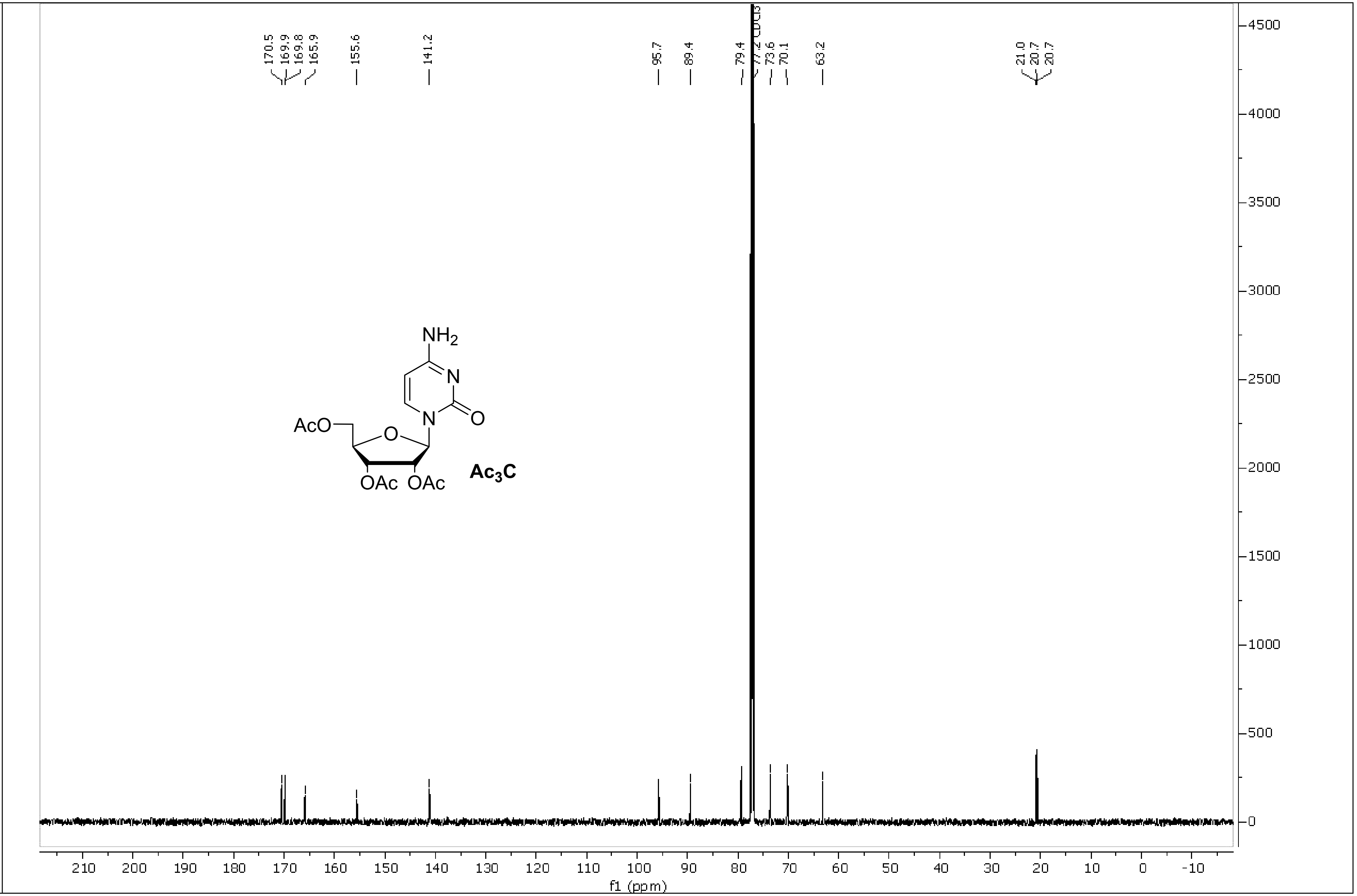

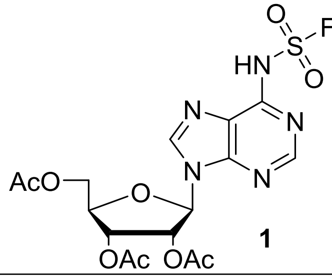

1

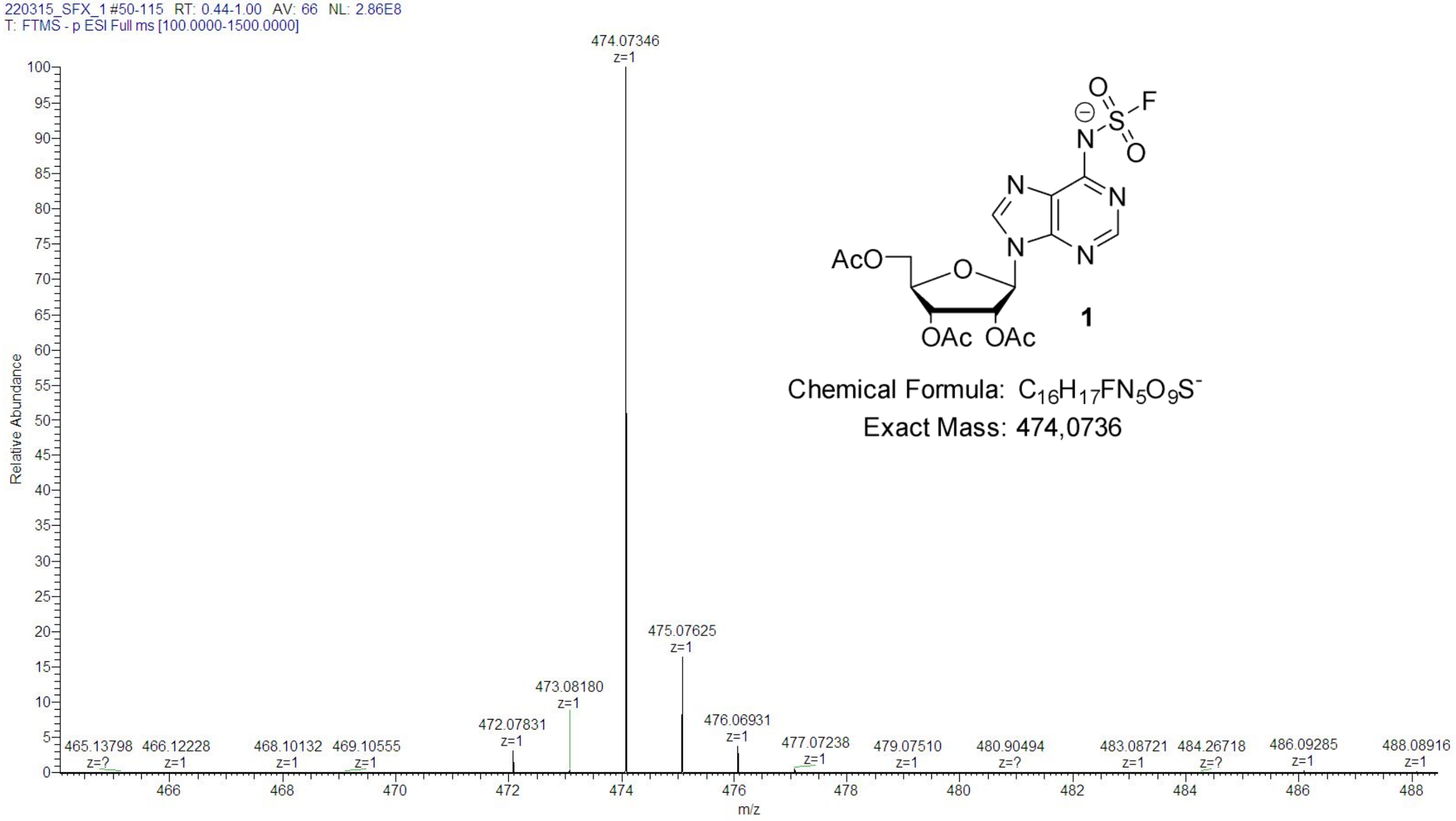

220315_SFX_1 #50-115 RT: 0.44-1.00 AV: 66 NL: 2.86E8
T: FTMS - p ESI Full ms [100.0000-1500.0000]
474.07346 z=1
475.07625 z=1
473.08180 z=1
472.07831 z=1
476.06931 z=1
477.07238 z=1
465.13798 z=?
466.12228 z=1
468.10132 z=1
469.10555 z=1
479.07510 z=1
480.90494 z=?
483.08721 z=1
484.26718 z=?
486.09285 z=1
488.08916 z=1
Relative Abundance
m/z
1
Chemical Formula: $C_{16}H_{17}FN_5O_9S^-$
Exact Mass: 474,0736

^{1}H NMR spectrum (500 MHz) of **1**

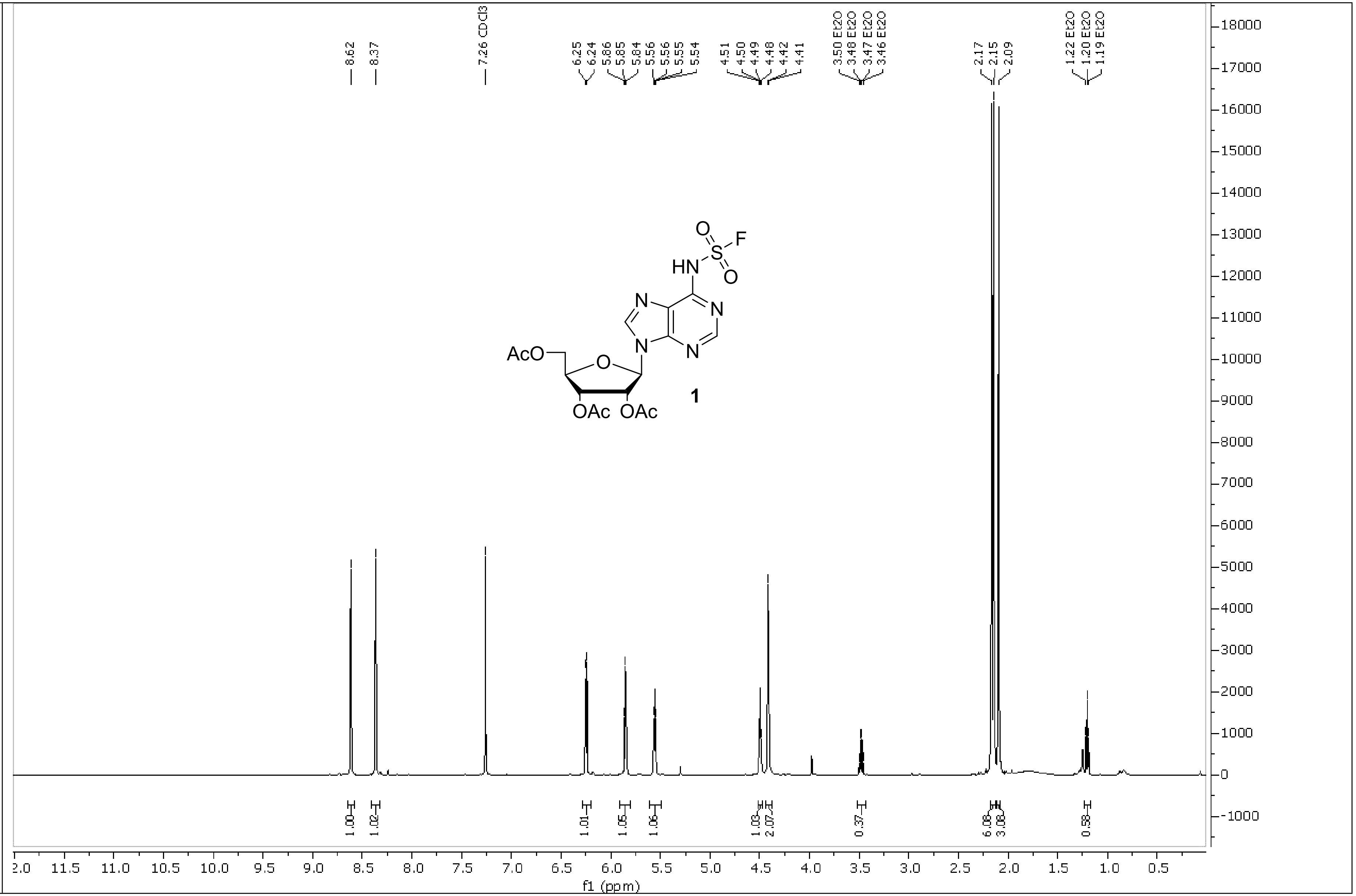

^{13}C NMR spectrum (126 MHz) of **1**

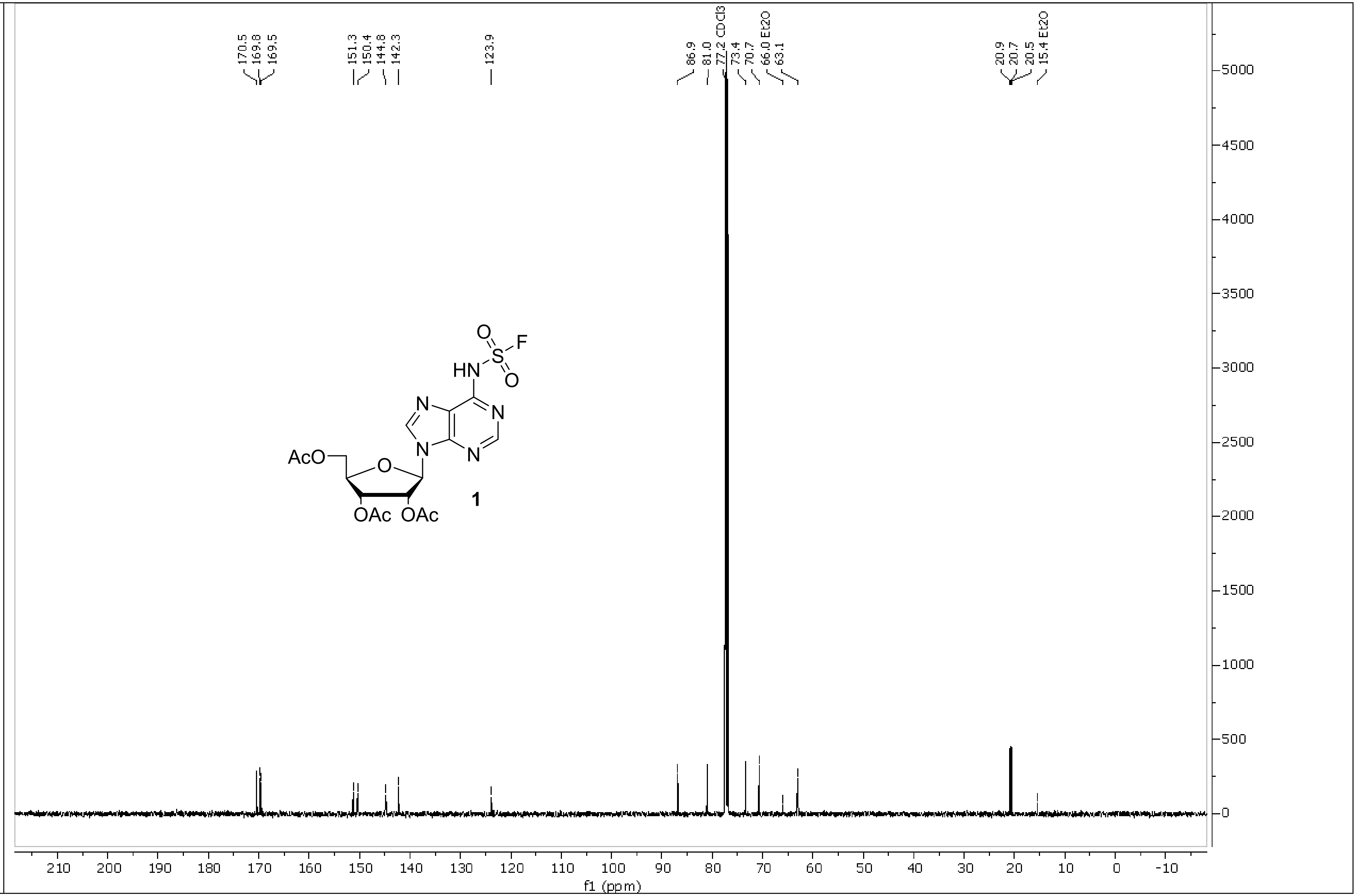

^{19}F NMR spectrum (471 MHz) of **1**

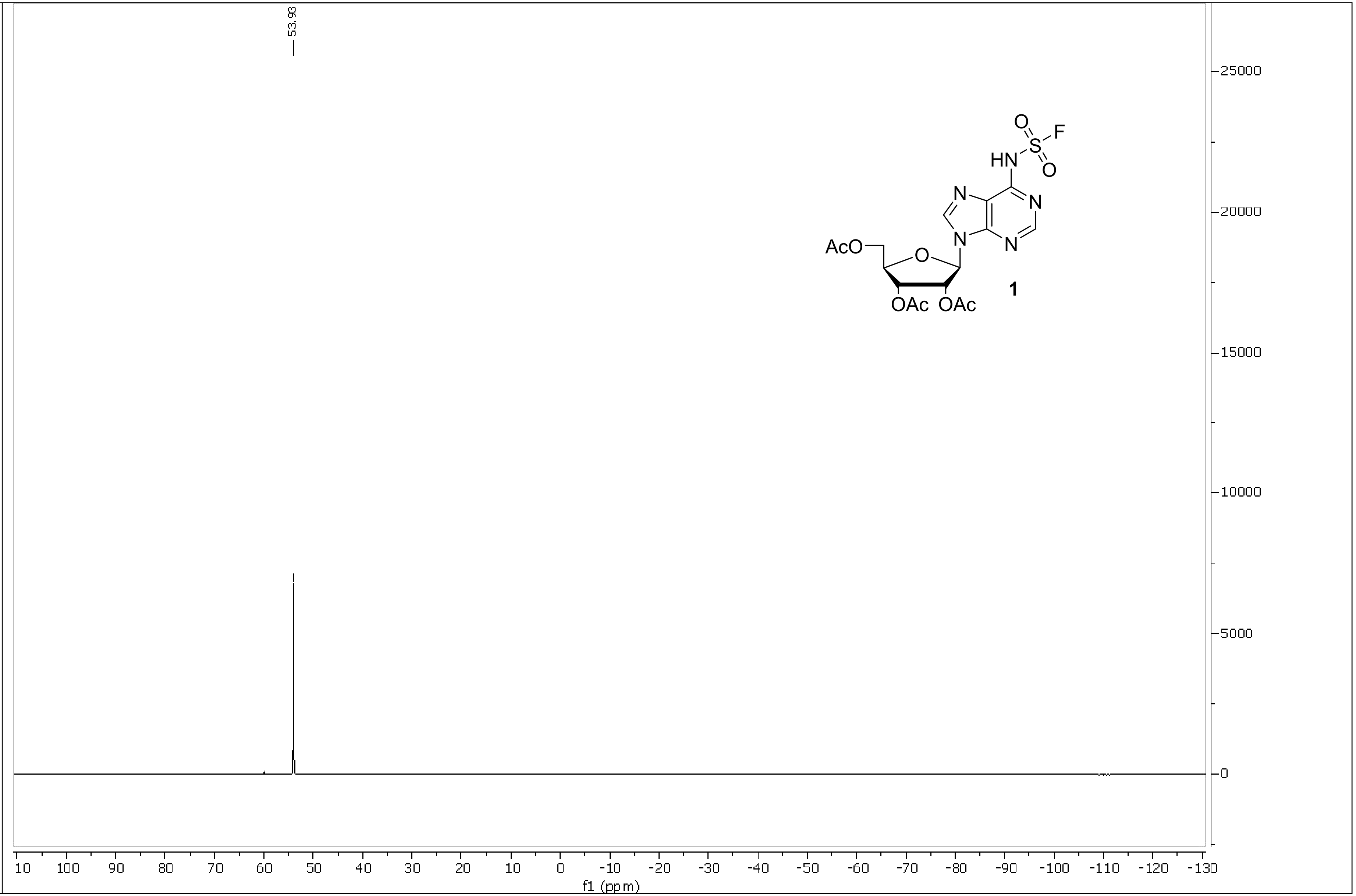

COSY NMR spectrum of **1**

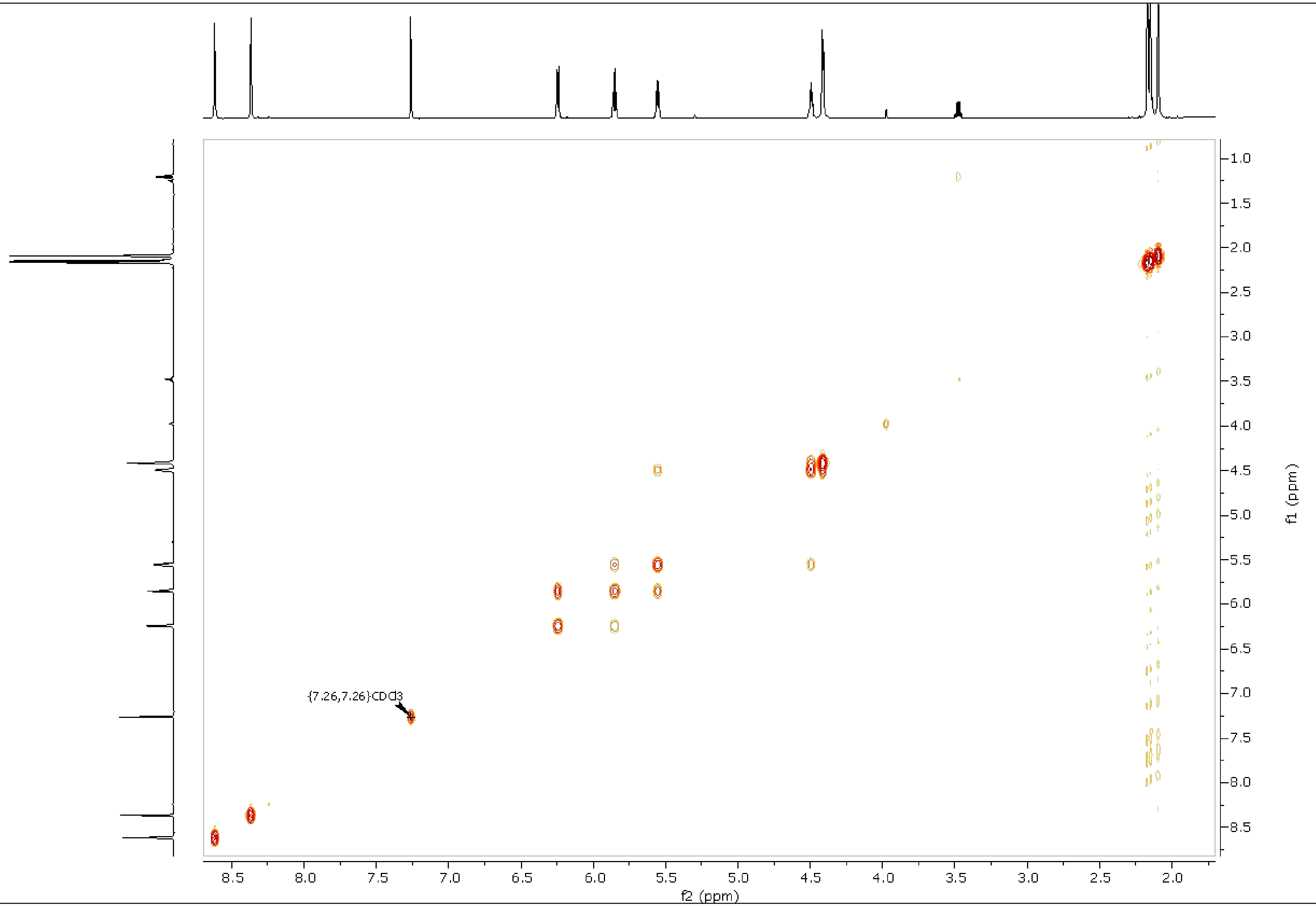

^{1}H – ^{13}C HSQC NMR spectrum of **1**

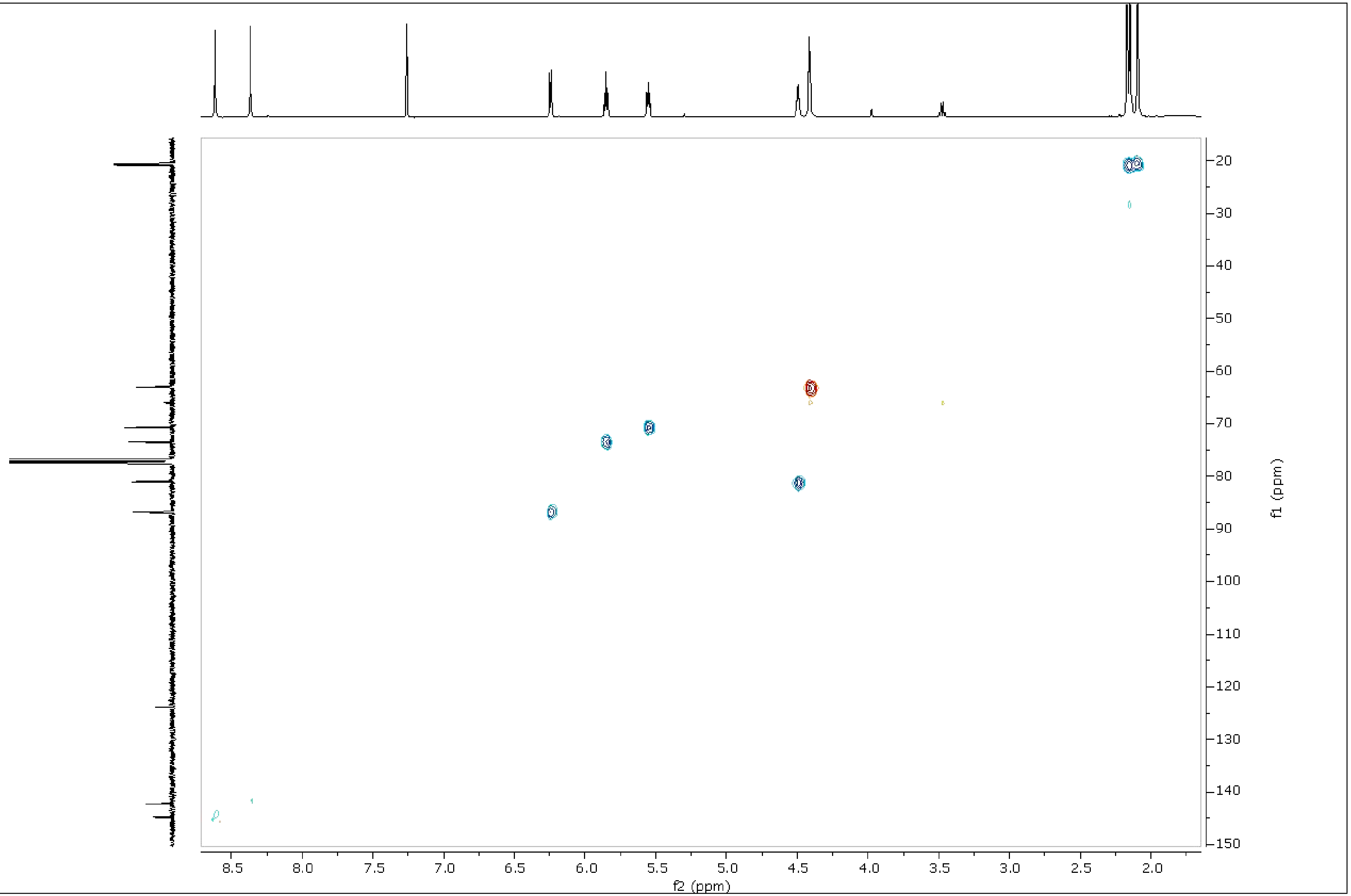

^{1}H – ^{13}C HMBC NMR spectrum of **1**

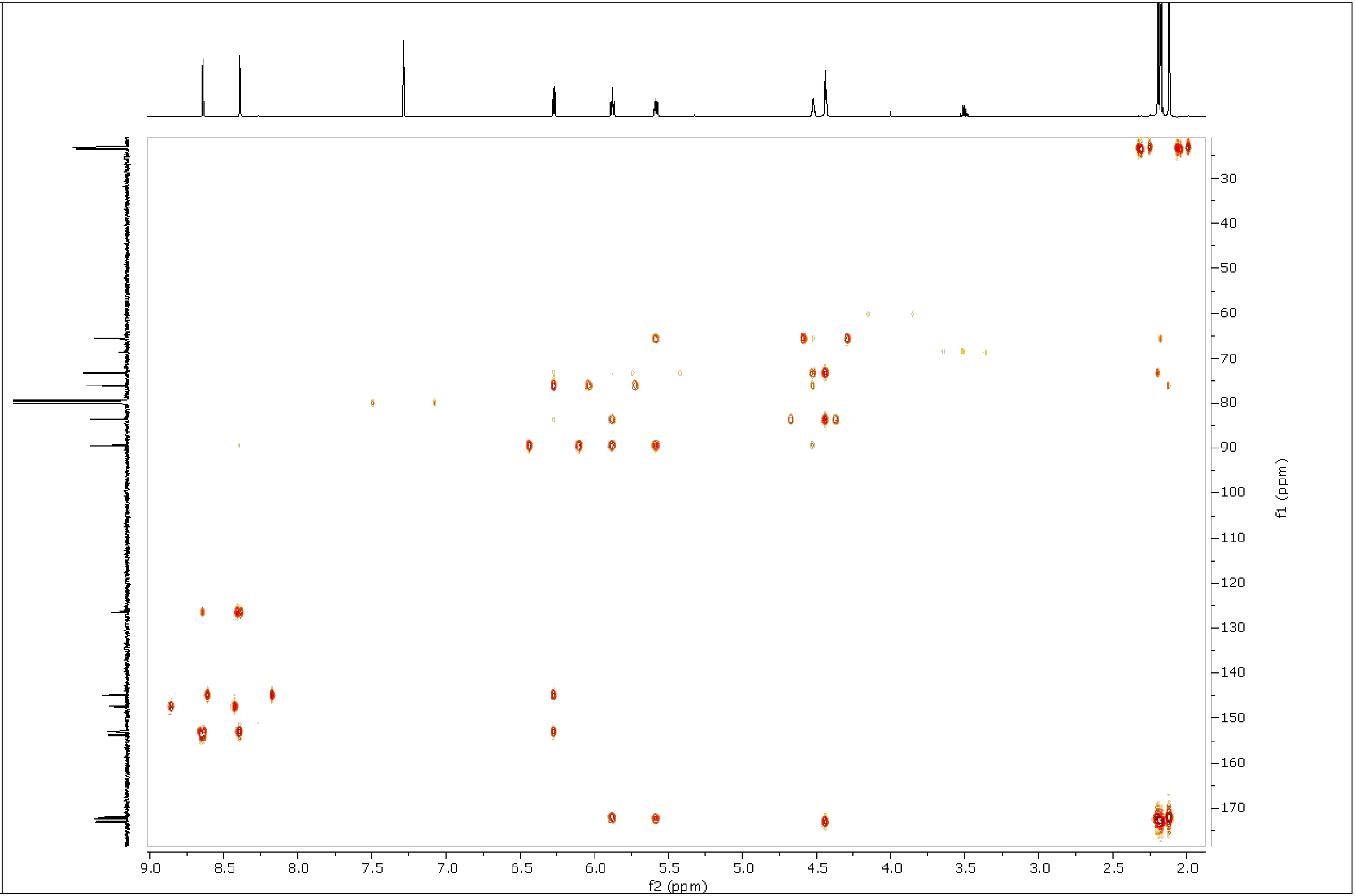

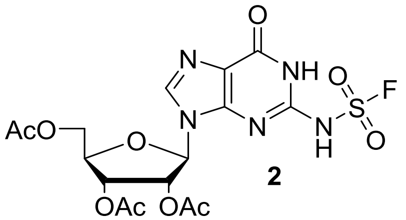

AcO
O
N
N
NH
O
N
H
S
O
F
O
OAc OAc

2

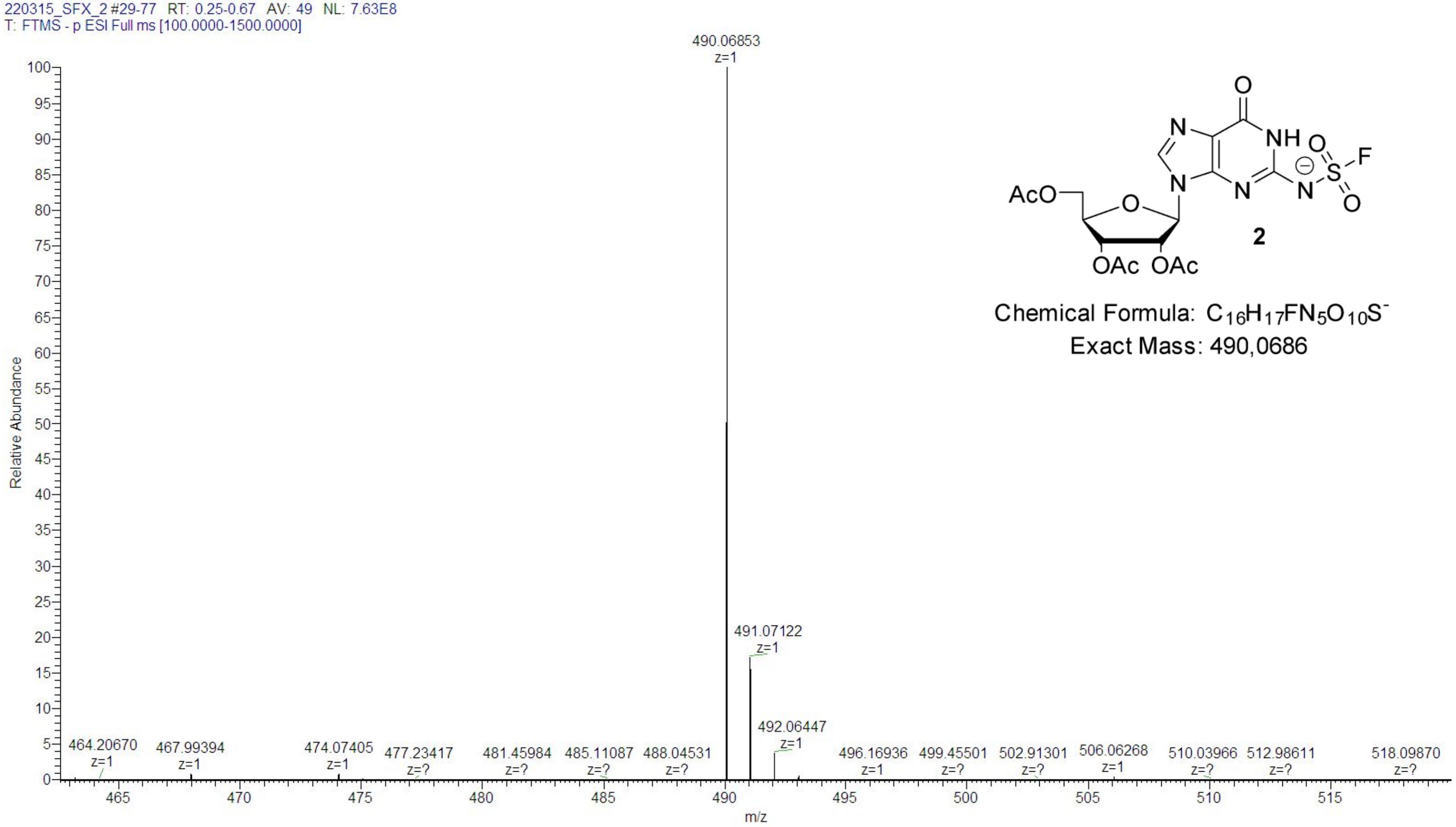

220315_SFX_2 #29-77 RT: 0.25-0.67 AV: 49 NL: 7.63E8
T: FTMS - p ESI Full ms [100.0000-1500.0000]
490.06853
z=1
491.07122
z=1
492.06447
z=1
464.20670
467.99394
474.07405
477.23417
481.45984
485.11087
488.04531
496.16936
499.45501
502.91301
506.06268
510.03966
512.98611
518.09870
Relative Abundance
m/z
2
Chemical Formula: C16H17FN5O10S-
Exact Mass: 490,0686

^{1}H NMR spectrum (500 MHz) of **2**

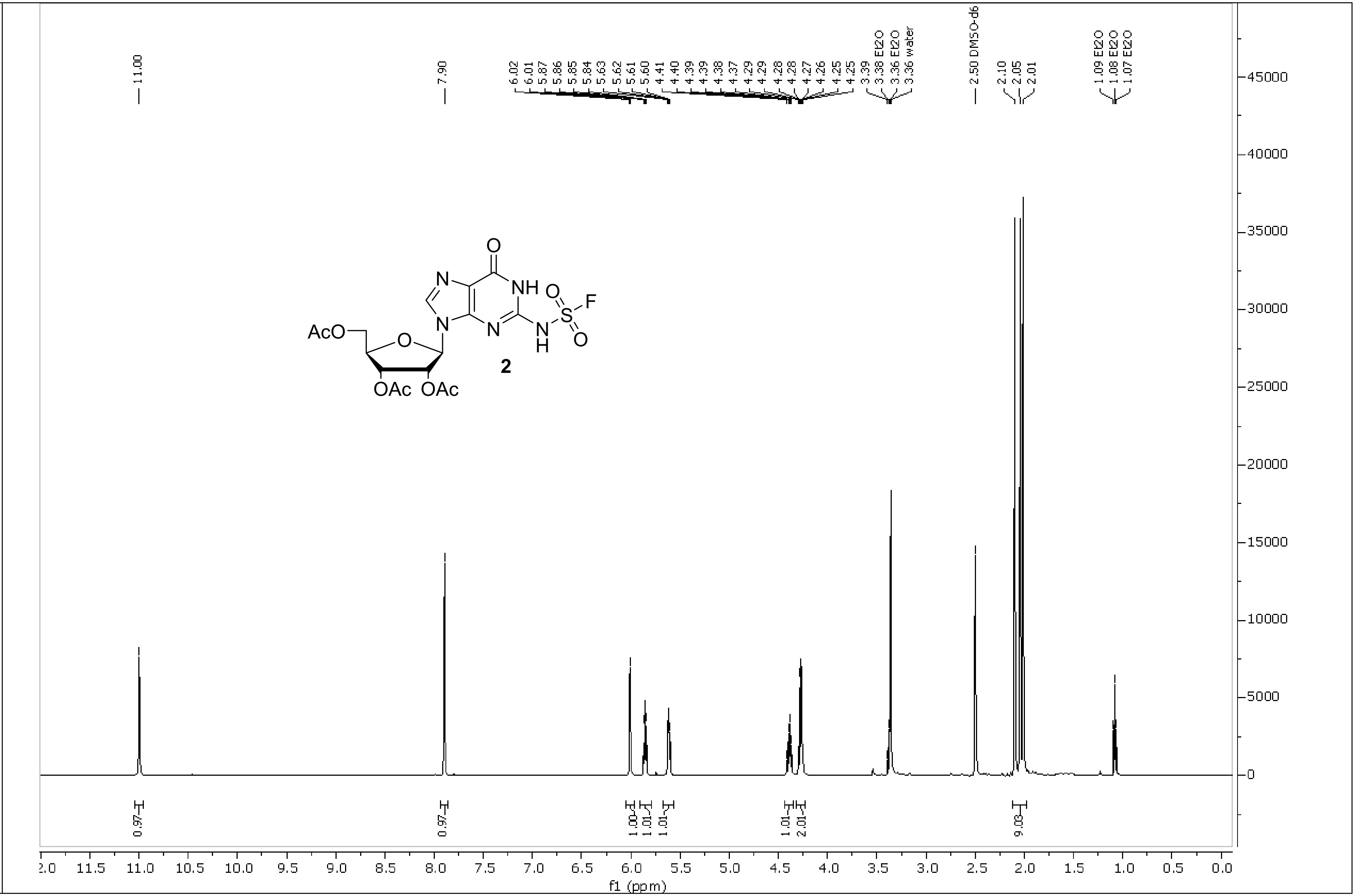

^{13}C NMR spectrum (126 MHz) of **2**

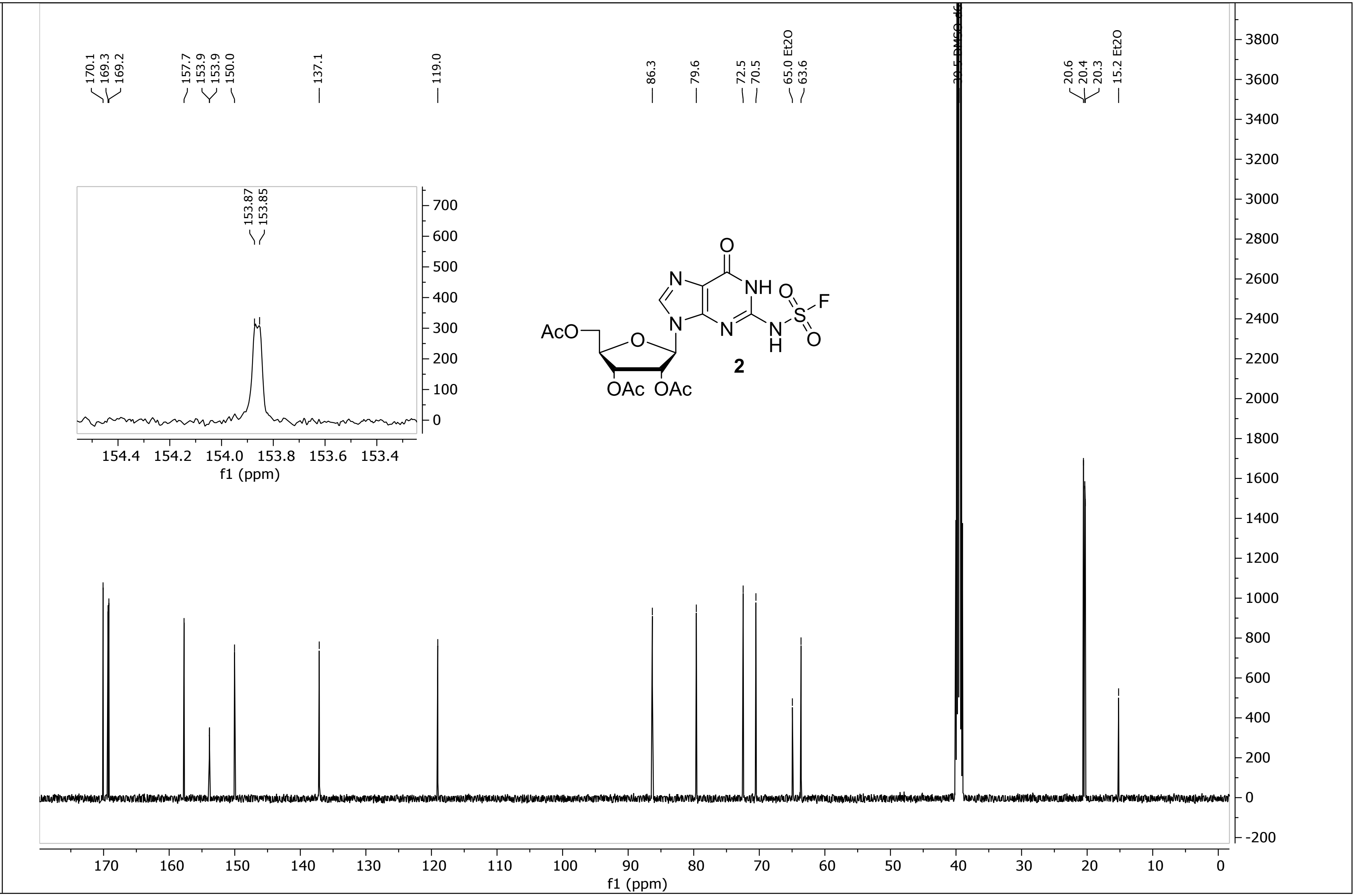

^{19}F NMR spectrum (471 MHz) of **2**

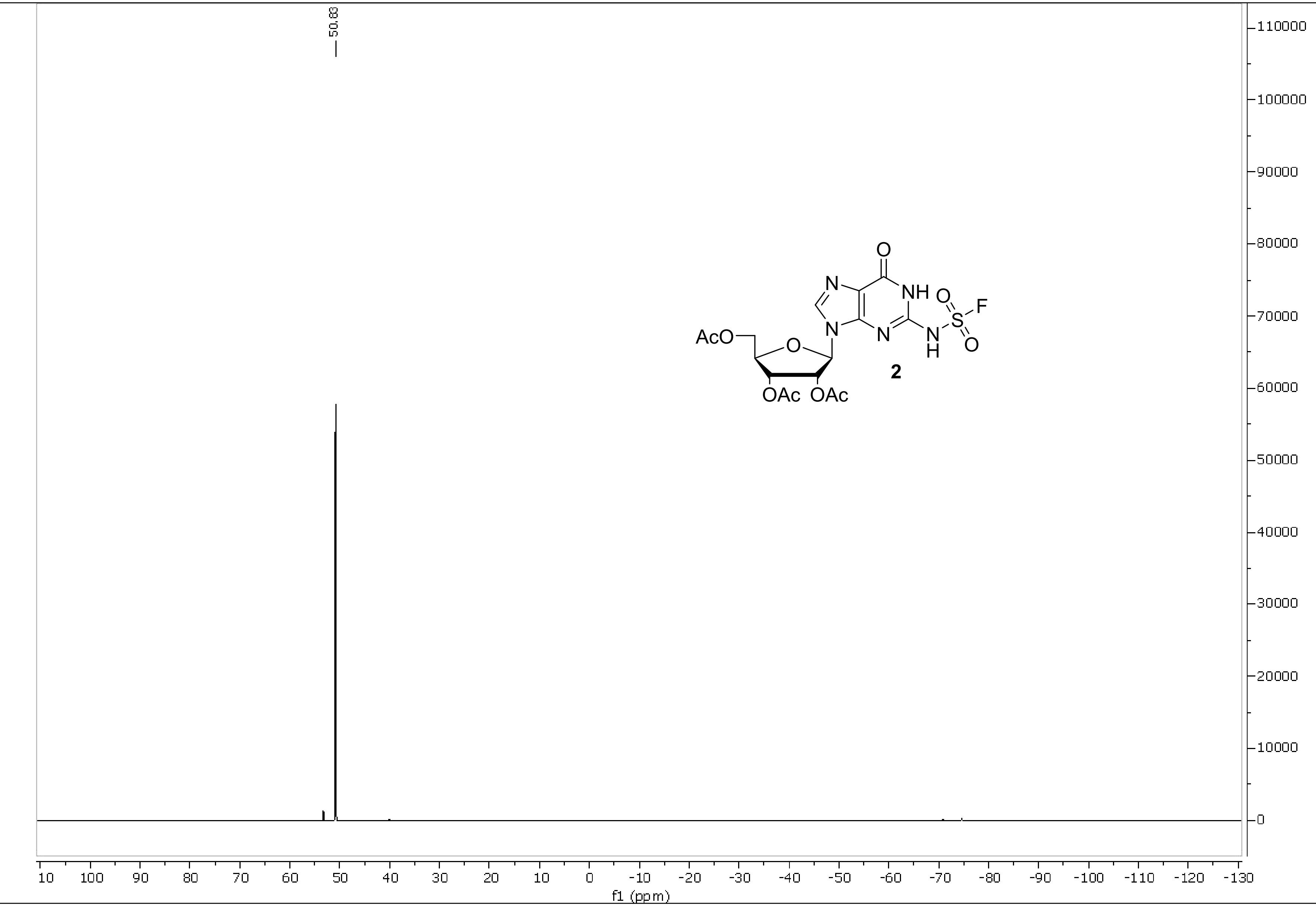

COSY NMR spectrum of **2**

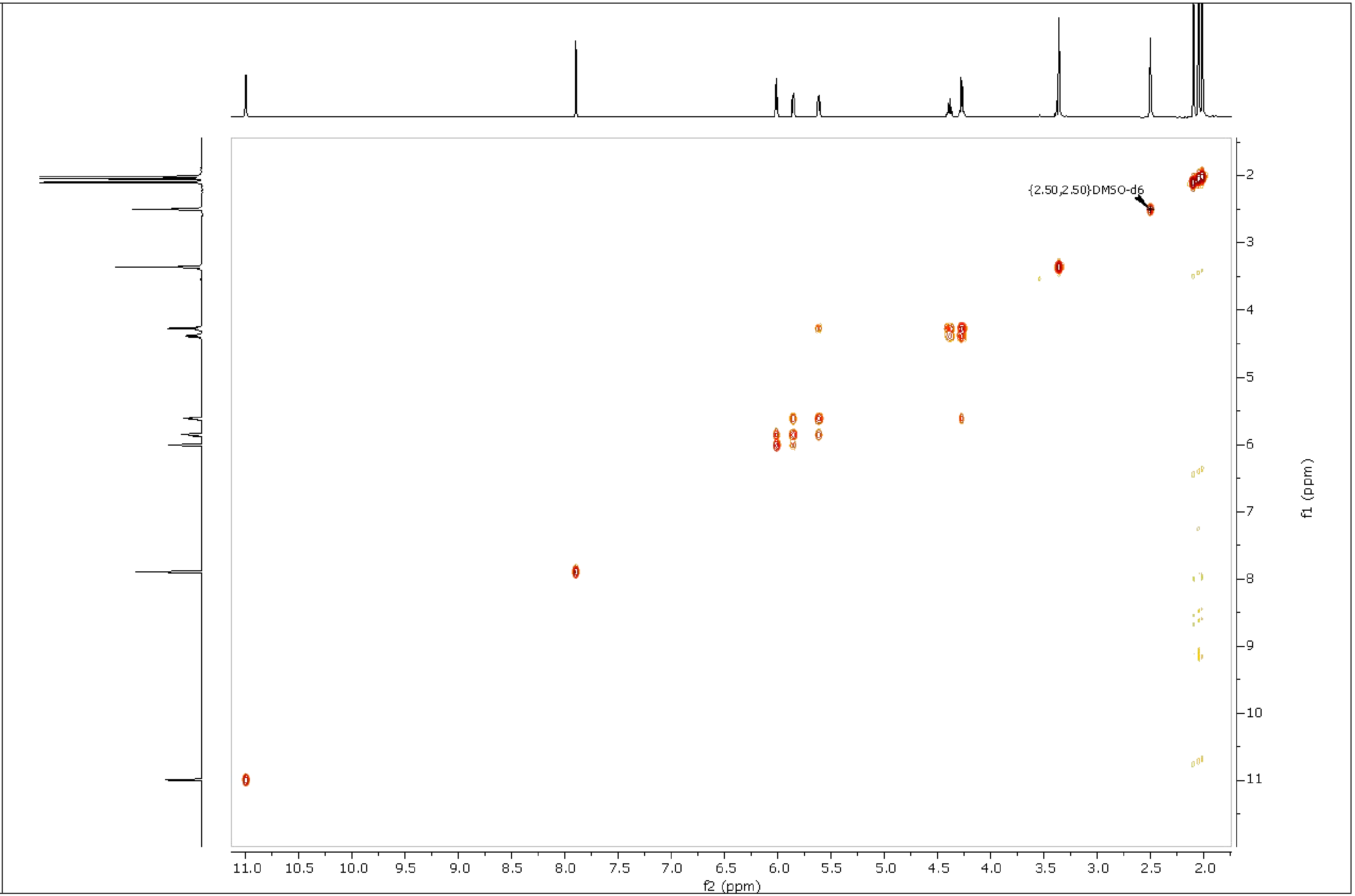

^{1}H – ^{13}C HSQC NMR spectrum of **2**

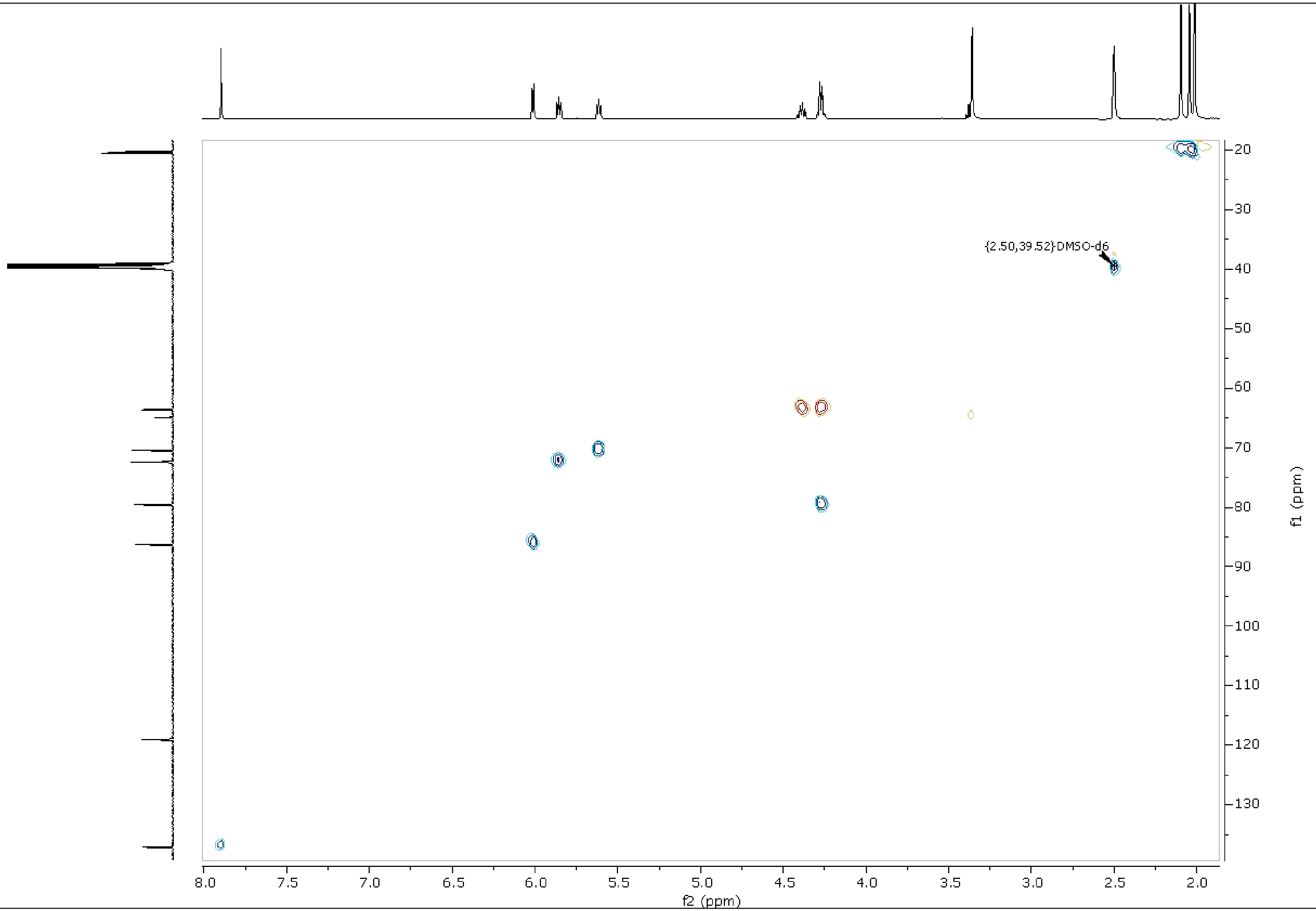

^{1}H – ^{13}C HMBC NMR spectrum of **2**

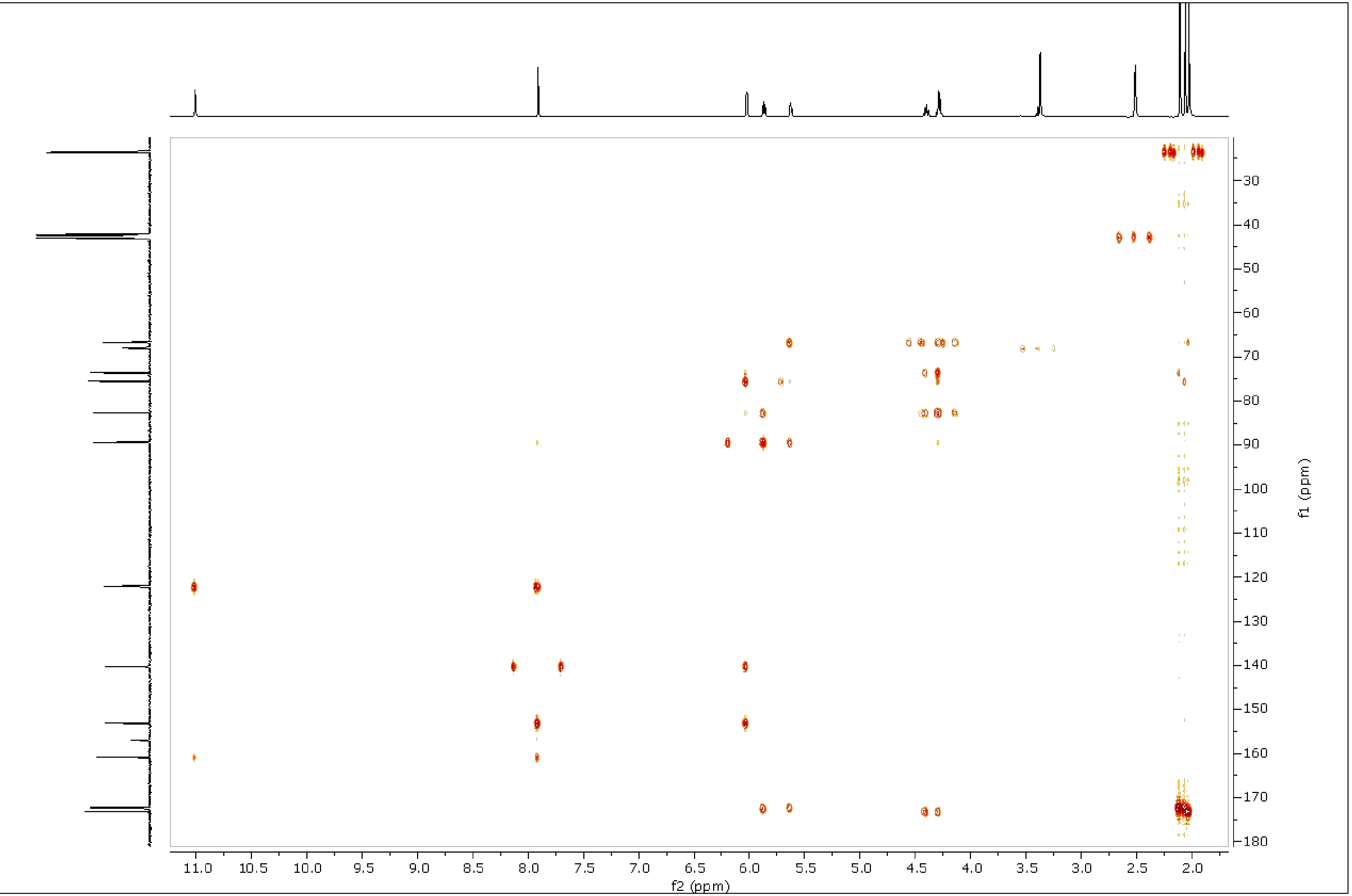

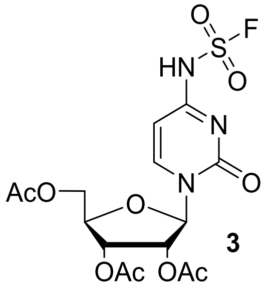

3

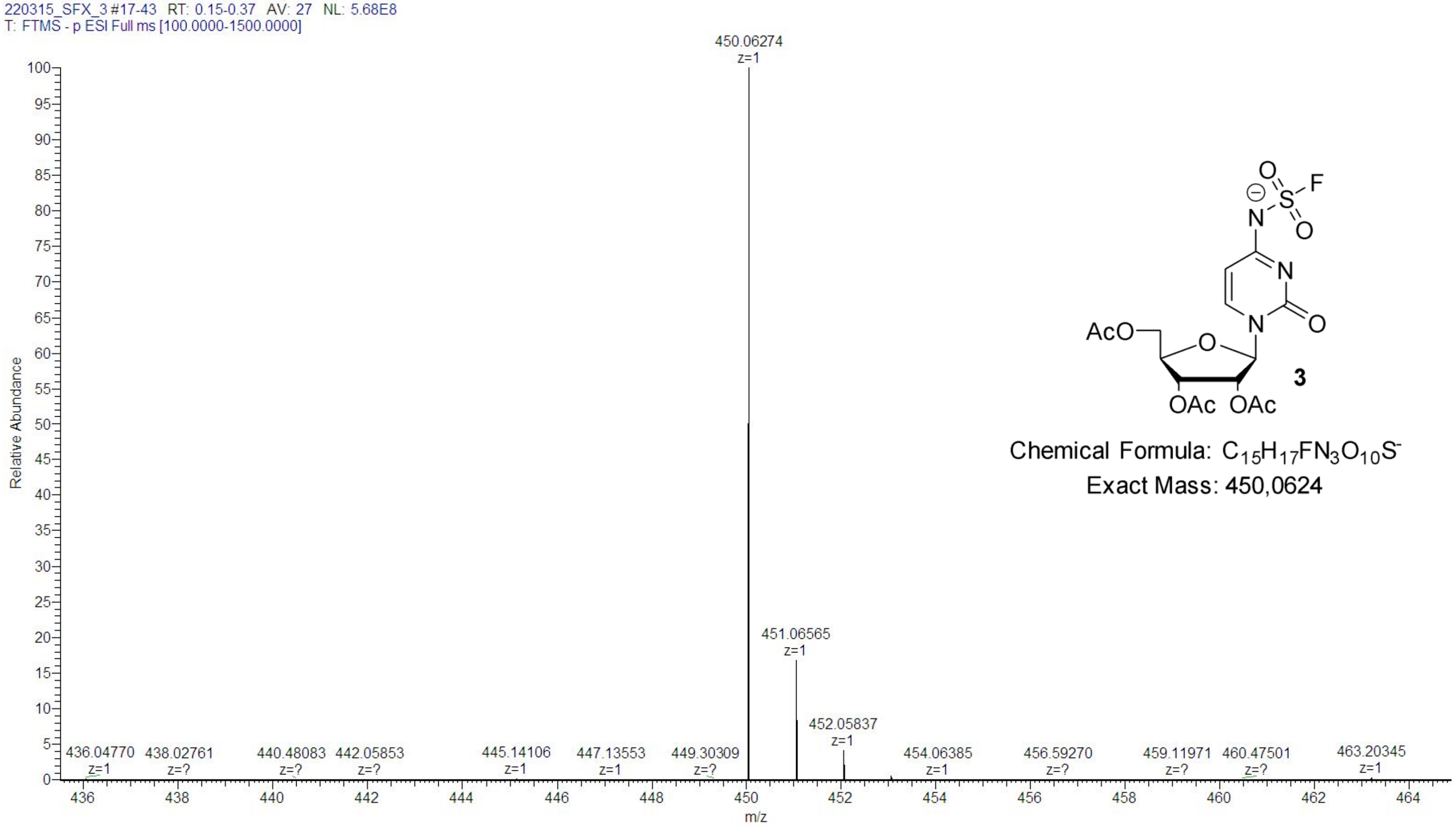

220315_SFX_3 #17-43 RT: 0.15-0.37 AV: 27 NL: 5.68E8
T: FTMS - p ESI Full ms [100.0000-1500.0000]
450.06274 z=1
451.06565 z=1
452.05837 z=1
436.04770 z=1
438.02761 z=?
440.48083 z=?
442.05853 z=?
445.14106 z=1
447.13553 z=1
449.30309 z=?
454.06385 z=1
456.59270 z=?
459.11971 z=?
460.47501 z=?
463.20345 z=1
Relative Abundance
m/z
3
Chemical Formula: $C_{15}H_{17}FN_3O_{10}S^-$
Exact Mass: 450,0624

^1H NMR spectrum (500 MHz) of **3**

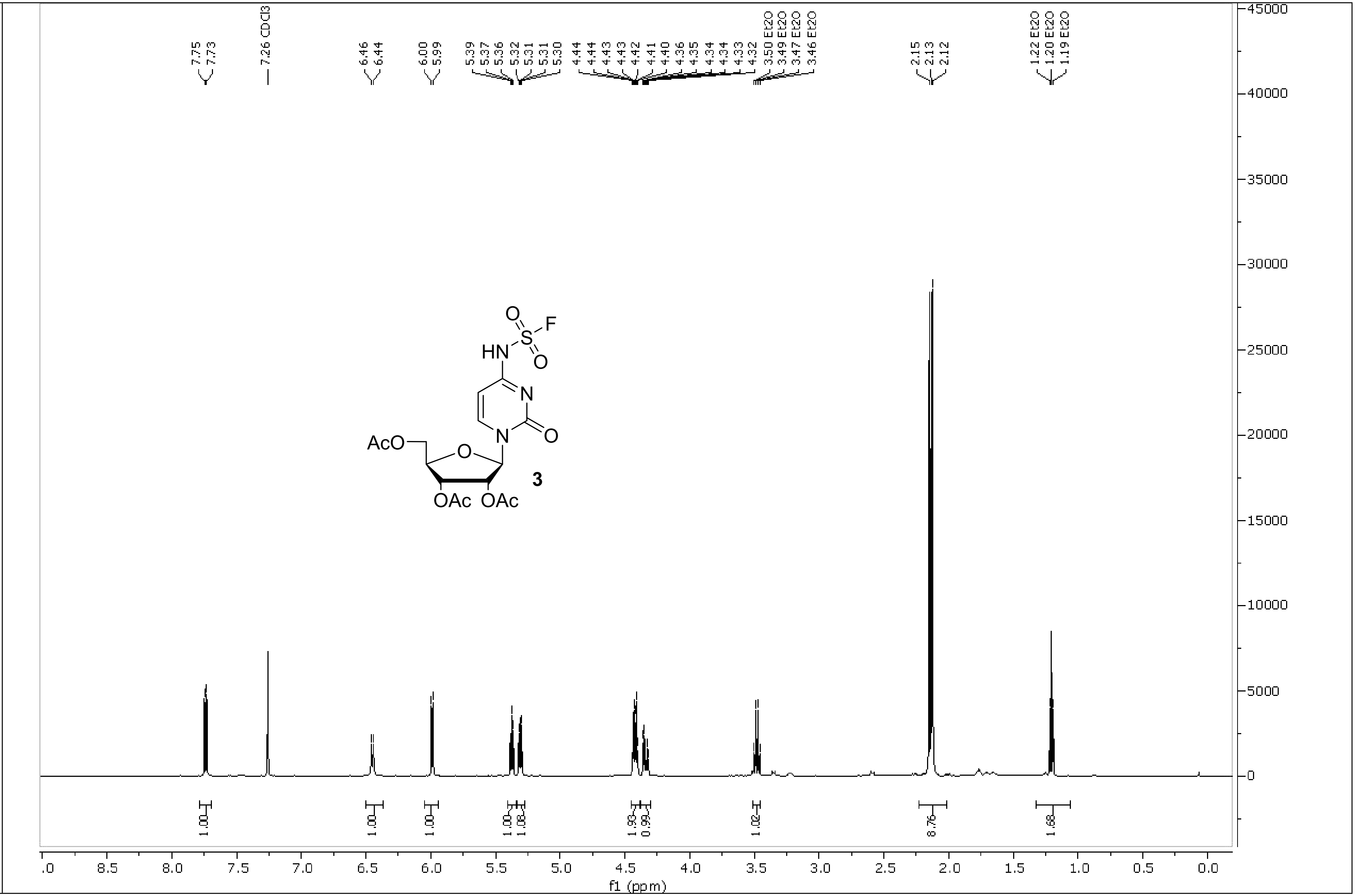

^{13}C NMR spectrum (126 MHz) of **3**

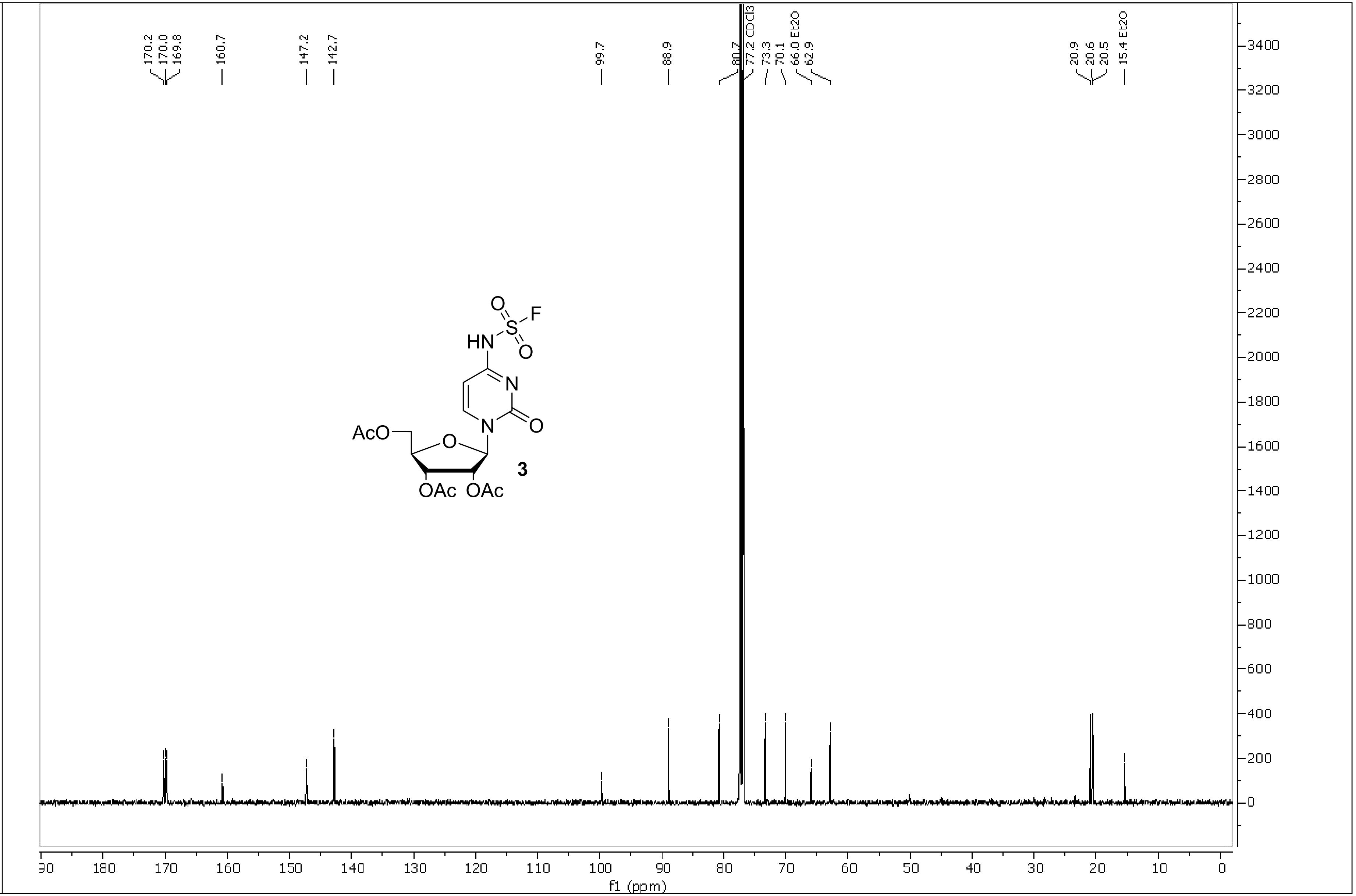

^{19}F NMR spectrum (471 MHz) of **3**

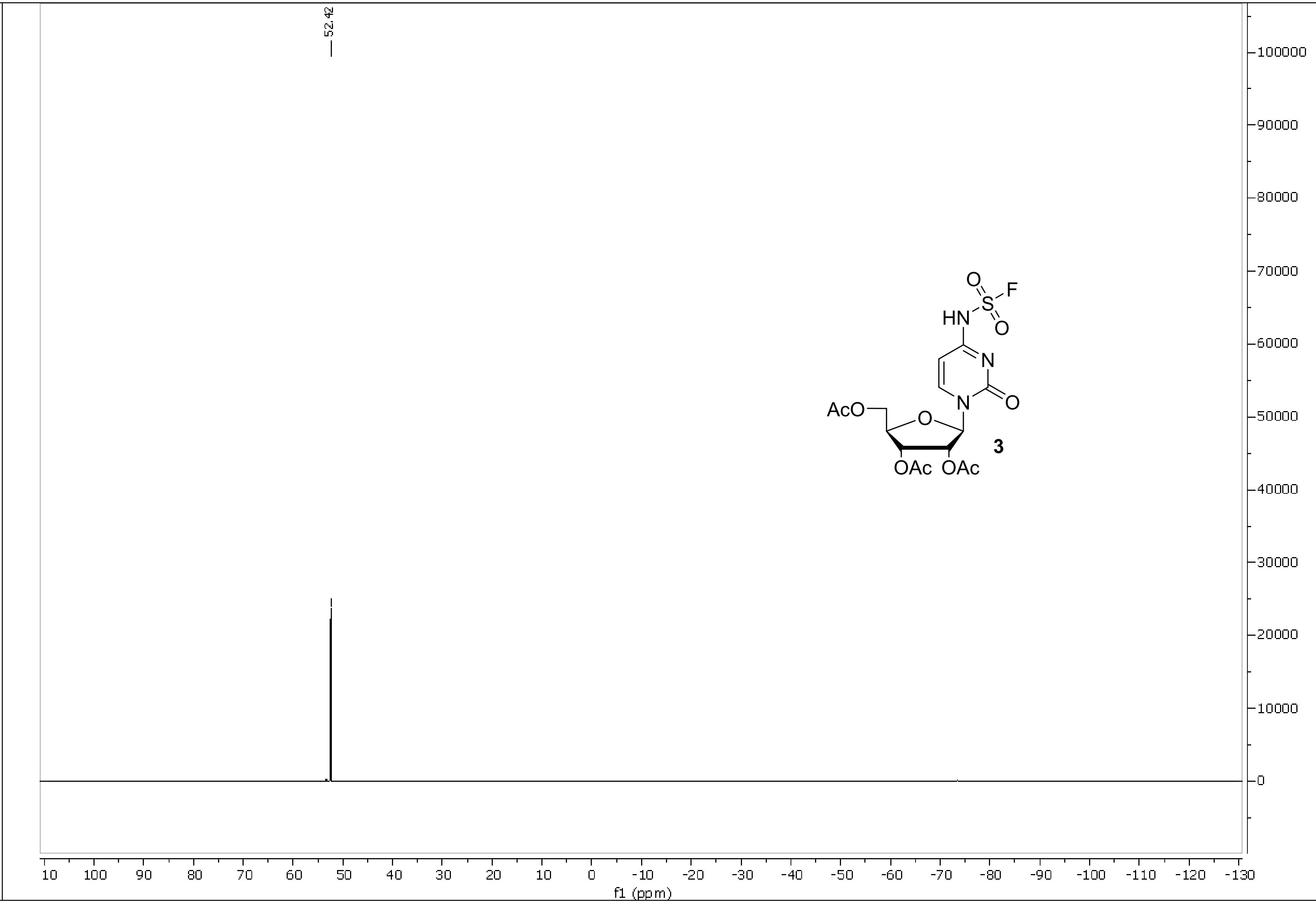

COSY NMR spectrum of **3**

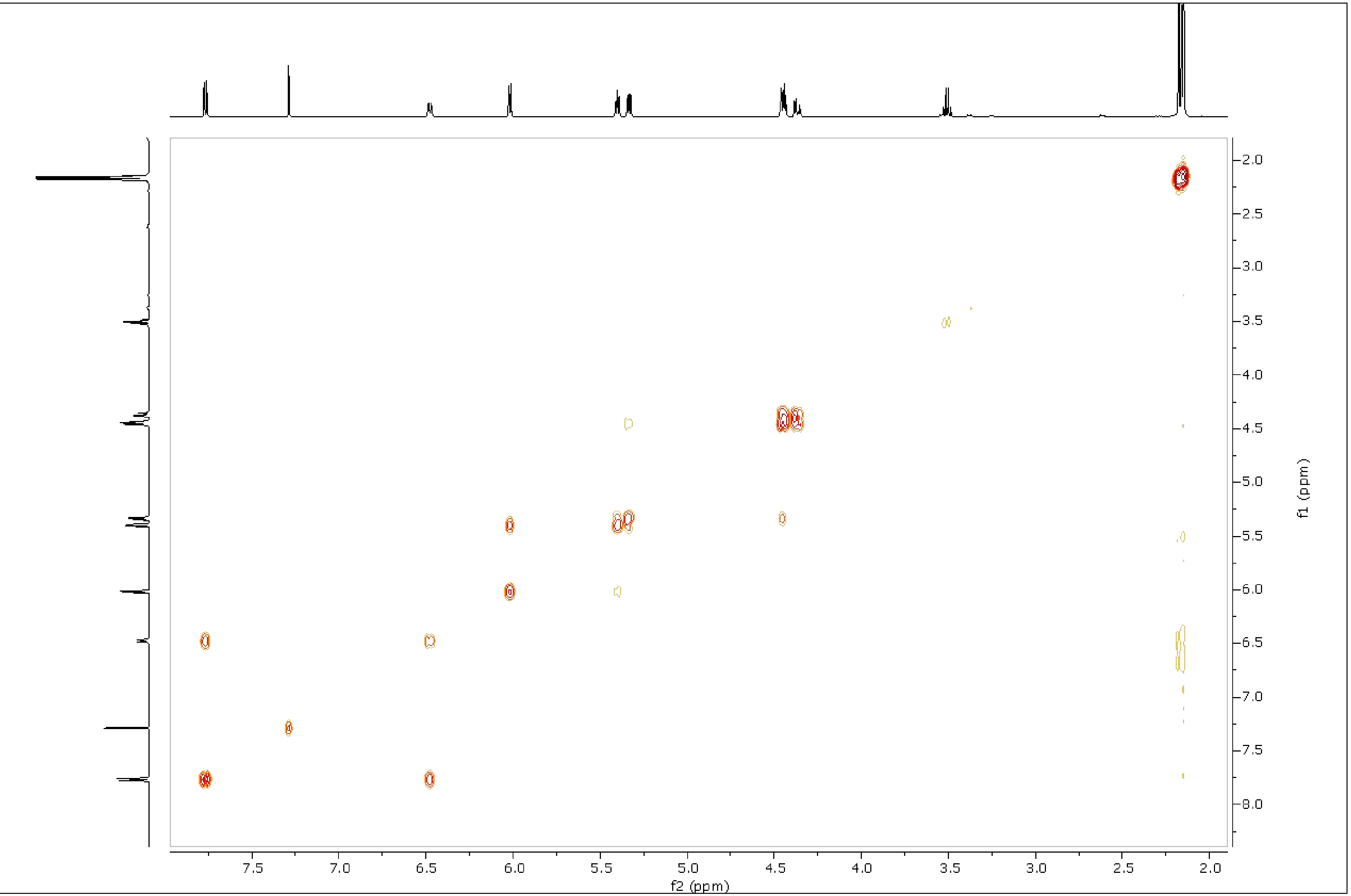

^{1}H – ^{13}C HSQC NMR spectrum of **3**

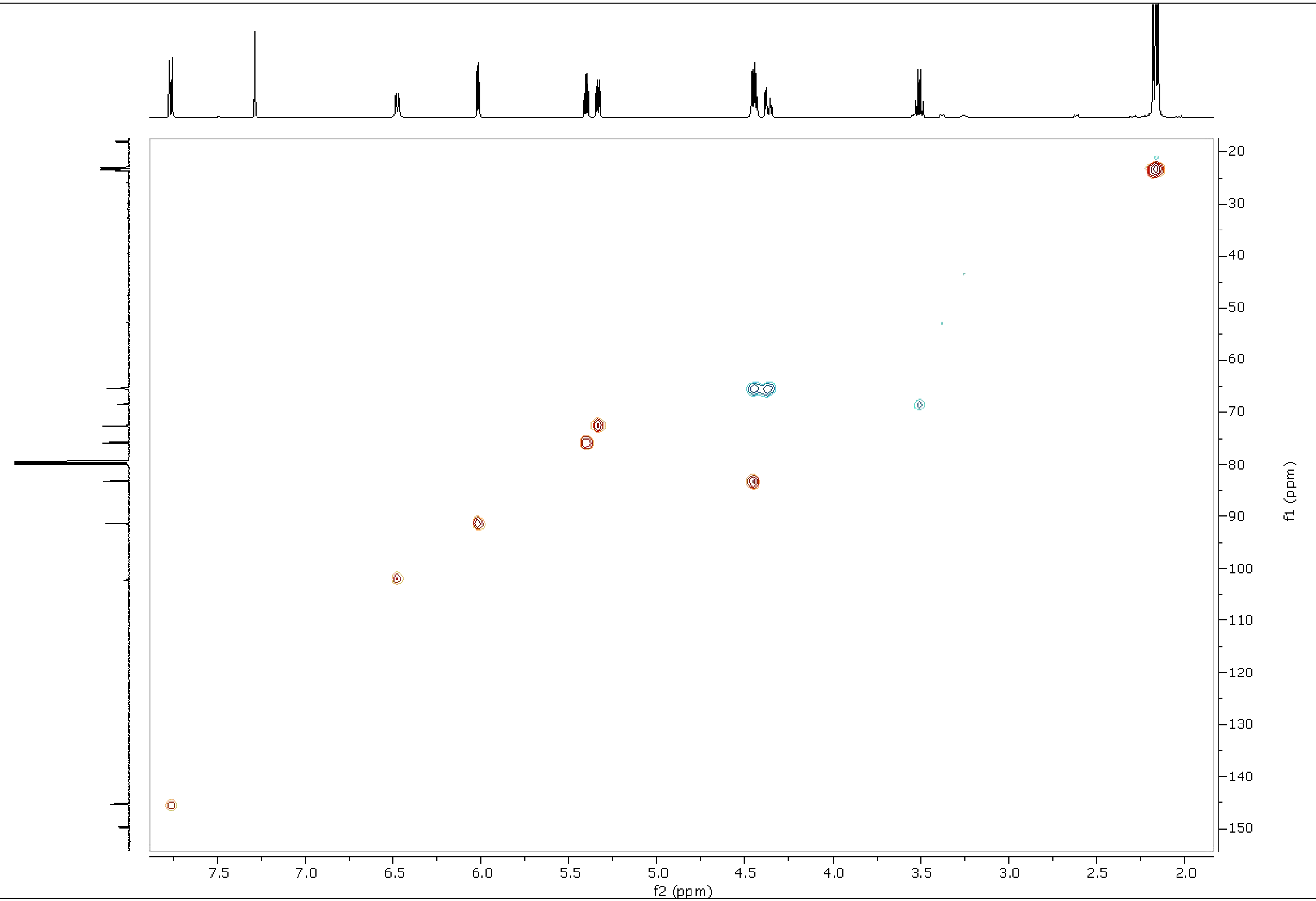

^{1}H – ^{13}C HMBC NMR spectrum of **3**

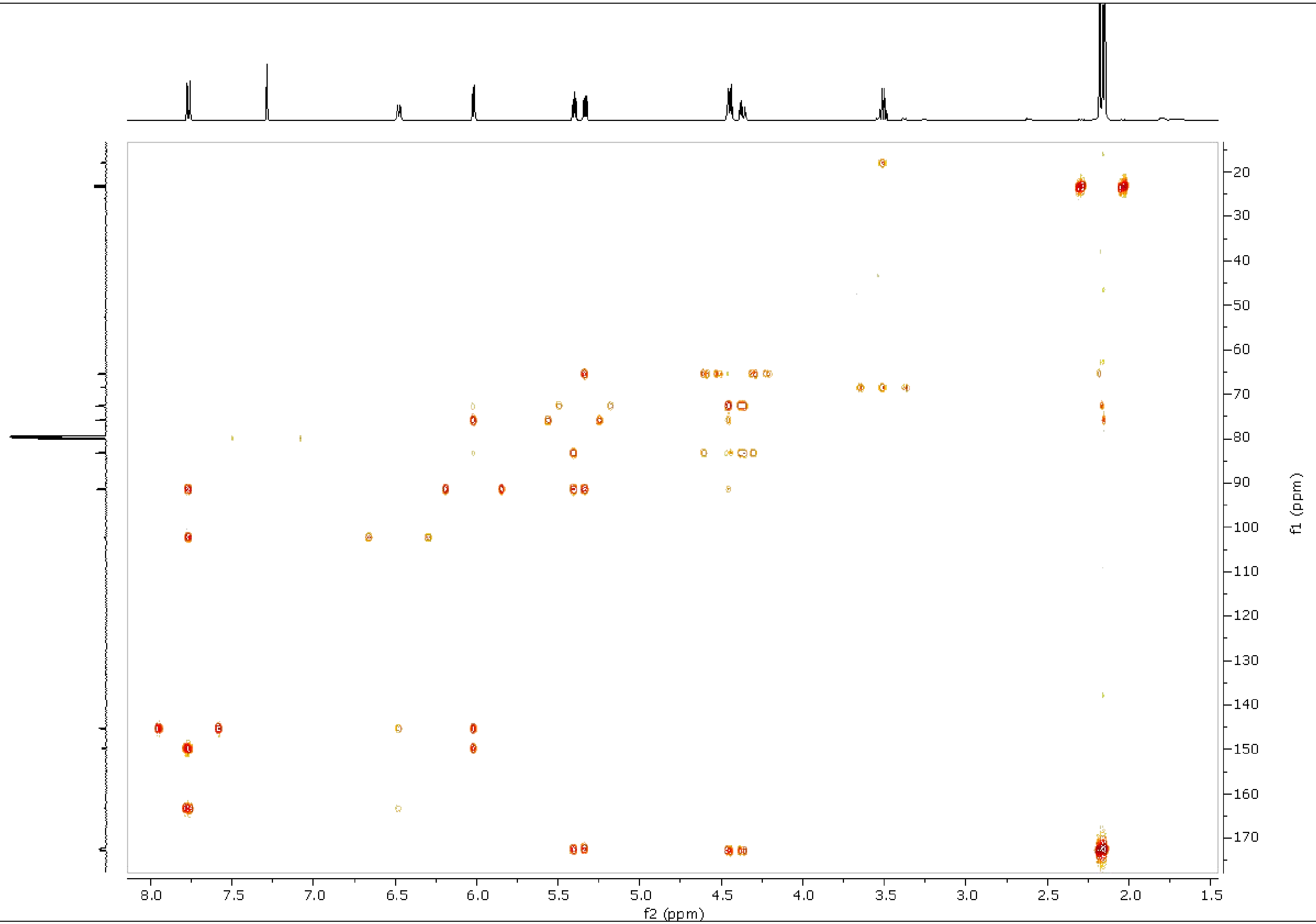

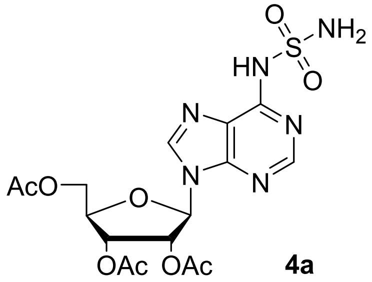

4a

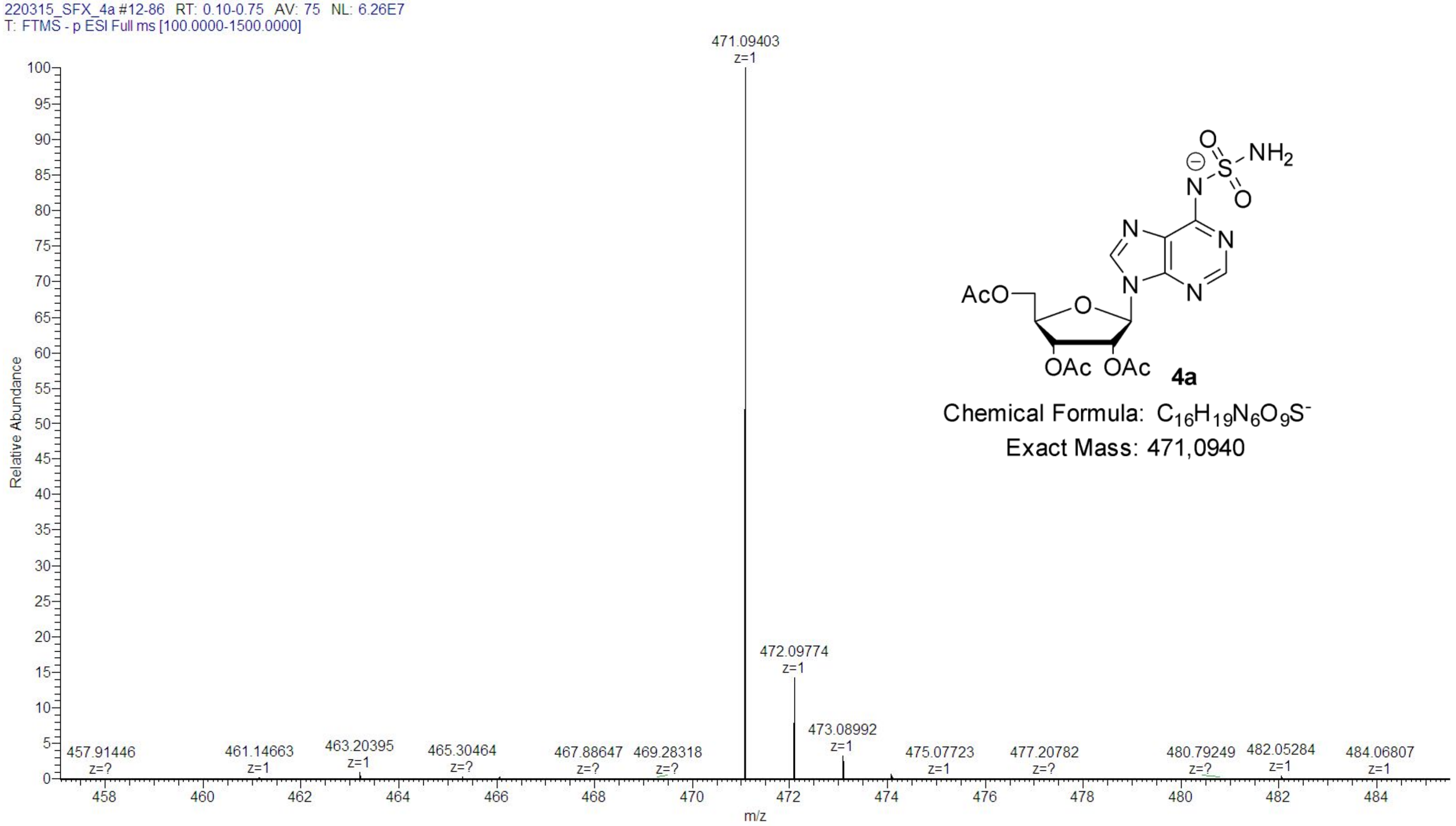

220315_SFX_4a #12-86 RT: 0.10-0.75 AV: 75 NL: 6.26E7
T: FTMS - p ESI Full ms [100.0000-1500.0000]
471.09403 z=1
472.09774 z=1
473.08992 z=1
457.91446 z=?
461.14663 z=1
463.20395 z=1
465.30464 z=?
467.88647 z=?
469.28318 z=?
475.07723 z=1
477.20782 z=?
480.79249 z=?
482.05284 z=1
484.06807 z=1
Relative Abundance
m/z
4a
Chemical Formula: $C_{16}H_{19}N_6O_9S^-$
Exact Mass: 471,0940

^{1}H NMR spectrum (500 MHz) of **4a**

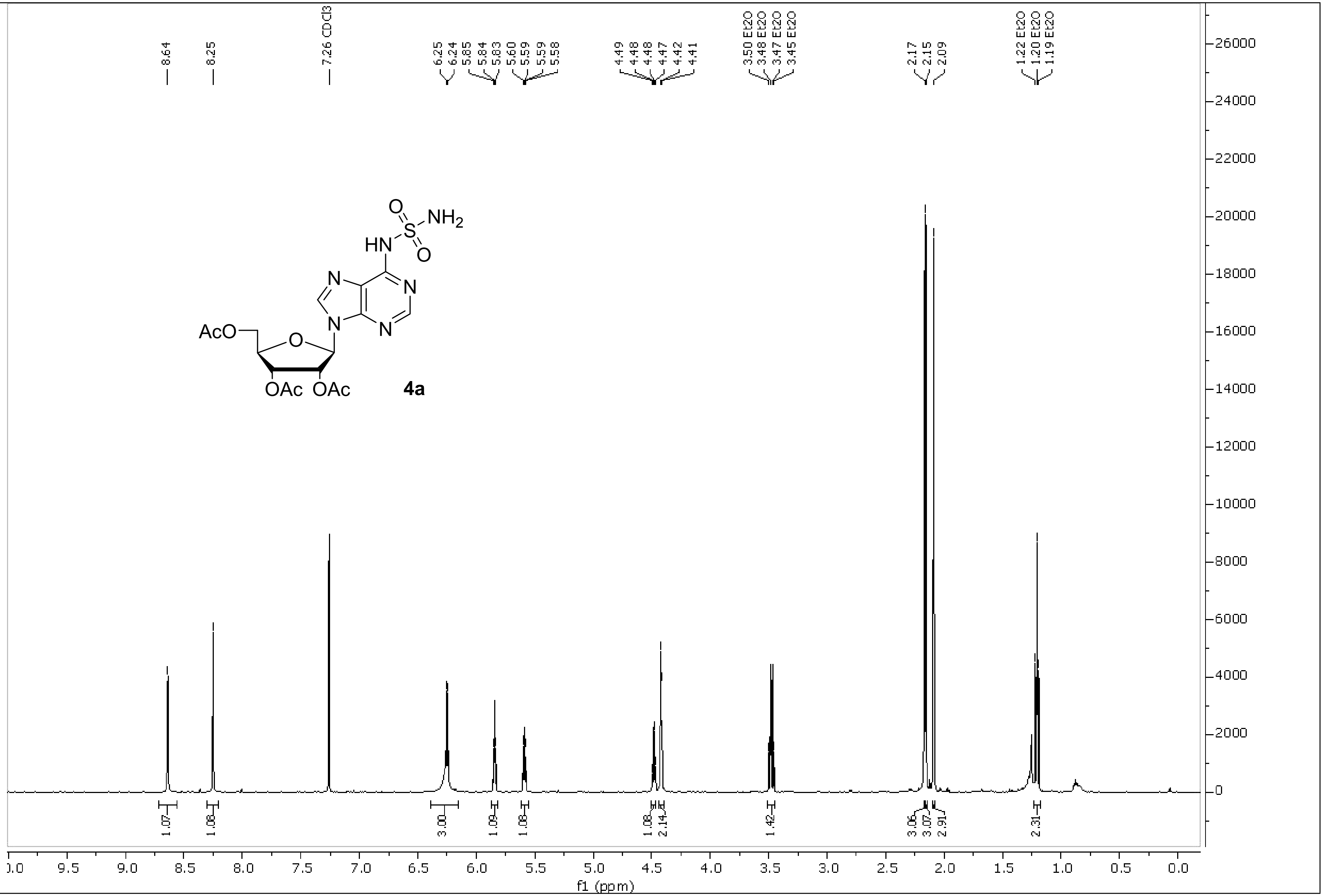

^{13}C NMR spectrum (126 MHz) of **4a**

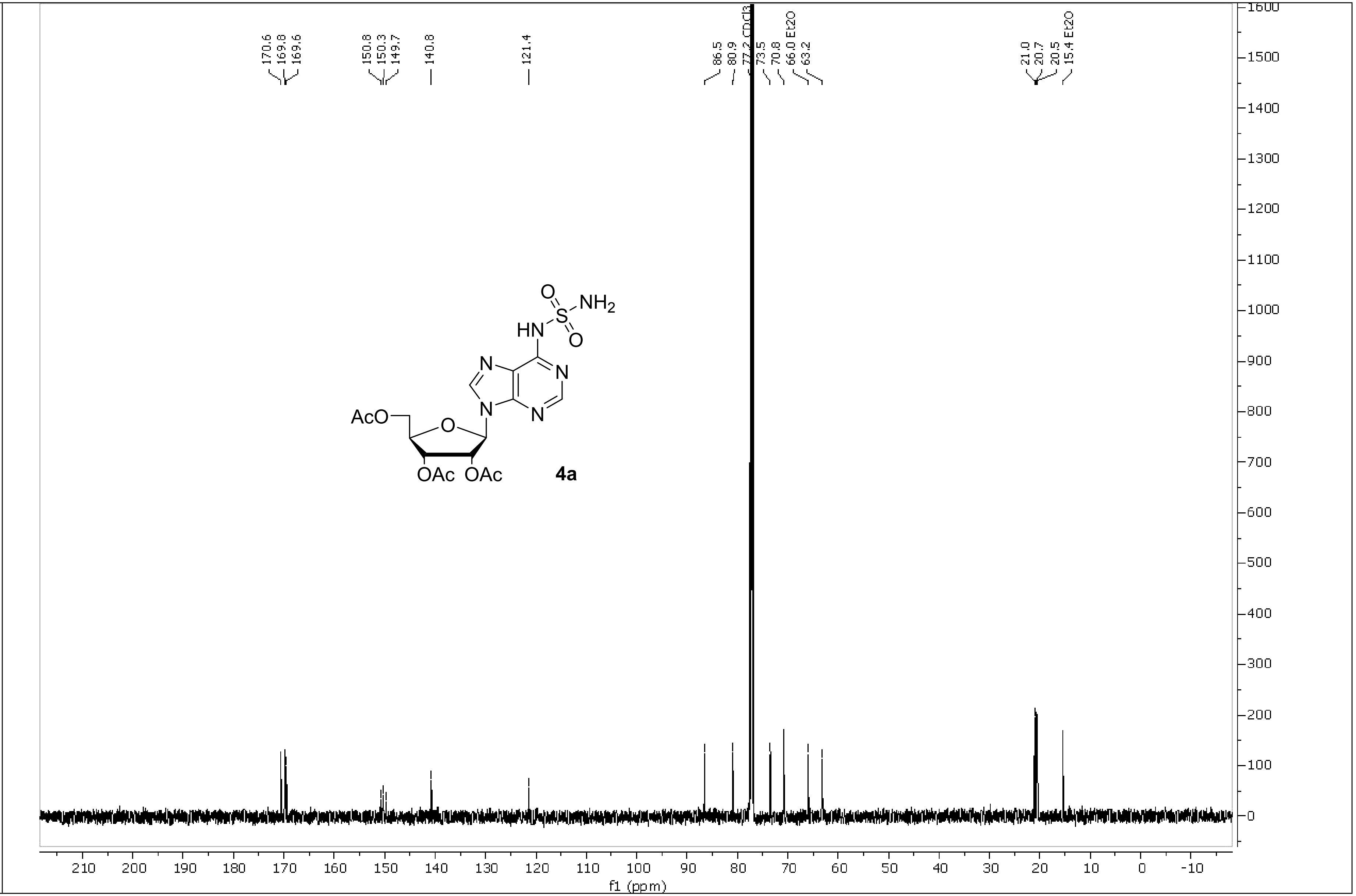

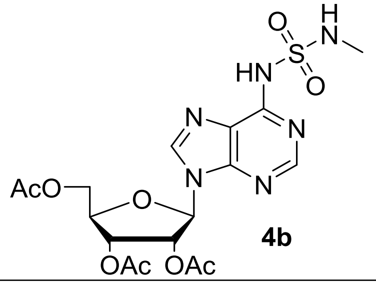

AcO
O
N
N
N
N
HN
S
O
O
N
H
OAc
OAc

4b

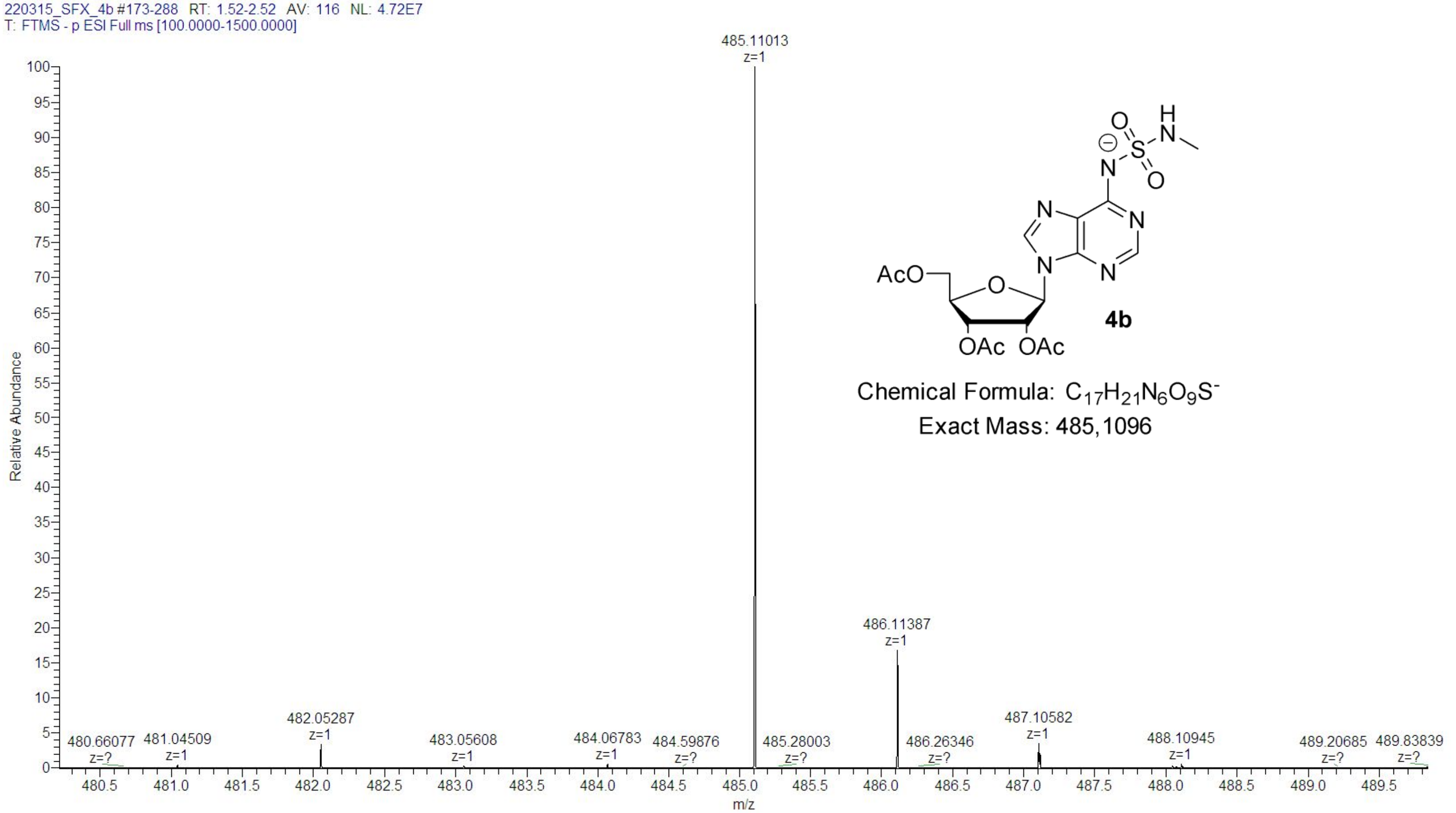

220315_SFX_4b #173-288 RT: 1.52-2.52 AV: 116 NL: 4.72E7
T: FTMS - p ESI Full ms [100.0000-1500.0000]
485.11013
z=1
486.11387
z=1
487.10582
z=1
482.05287
z=1
480.66077
z=?
481.04509
z=1
483.05608
z=1
484.06783
z=1
484.59876
z=?
485.28003
z=?
486.26346
z=?
488.10945
z=1
489.20685
z=?
489.83839
z=?
Relative Abundance
m/z
4b
Chemical Formula: $C_{17}H_{21}N_6O_9S^-$
Exact Mass: 485,1096

^{1}H NMR spectrum (500 MHz) of **4b**

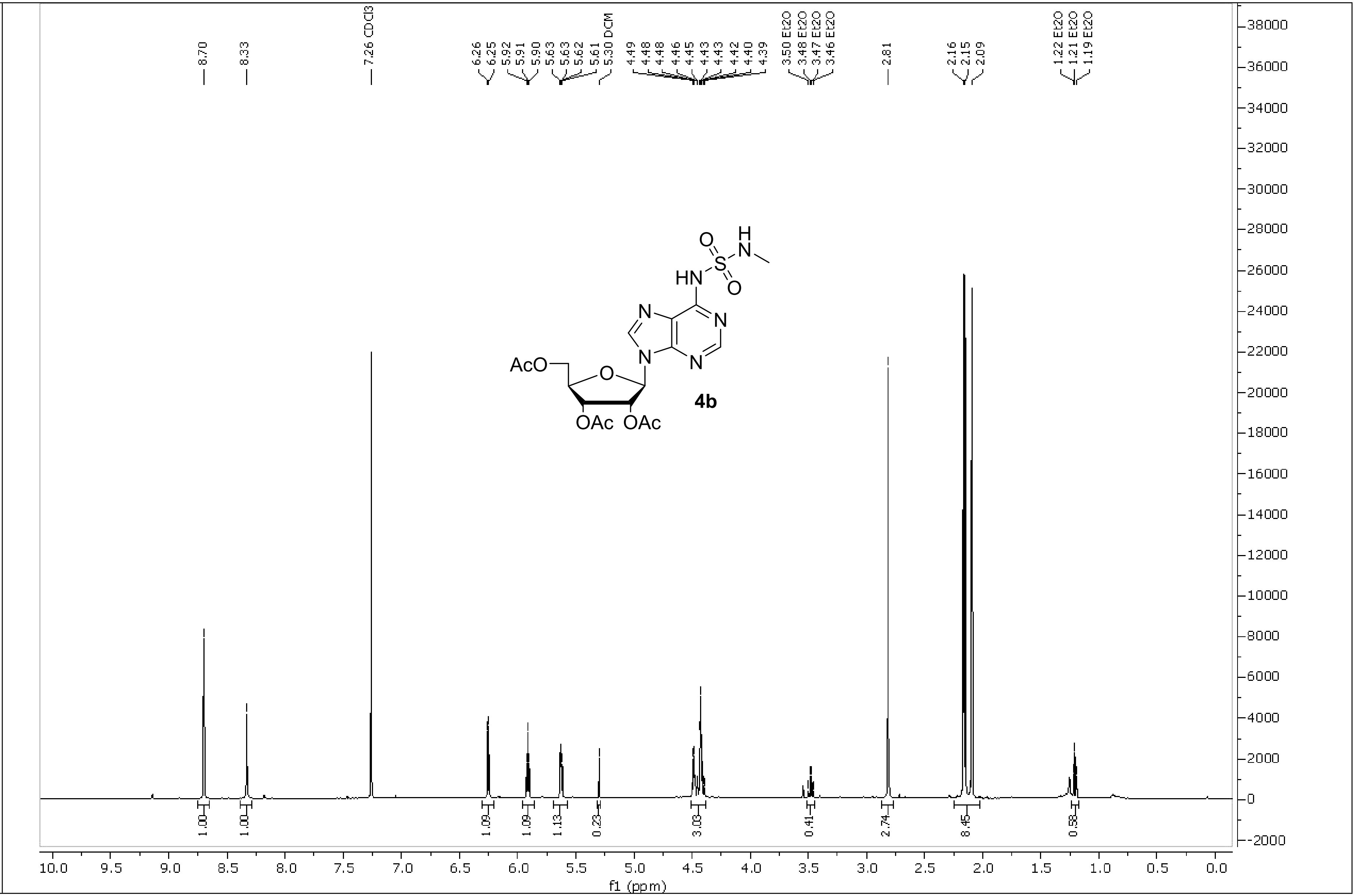

^{13}C NMR spectrum (126 MHz) of **4b**

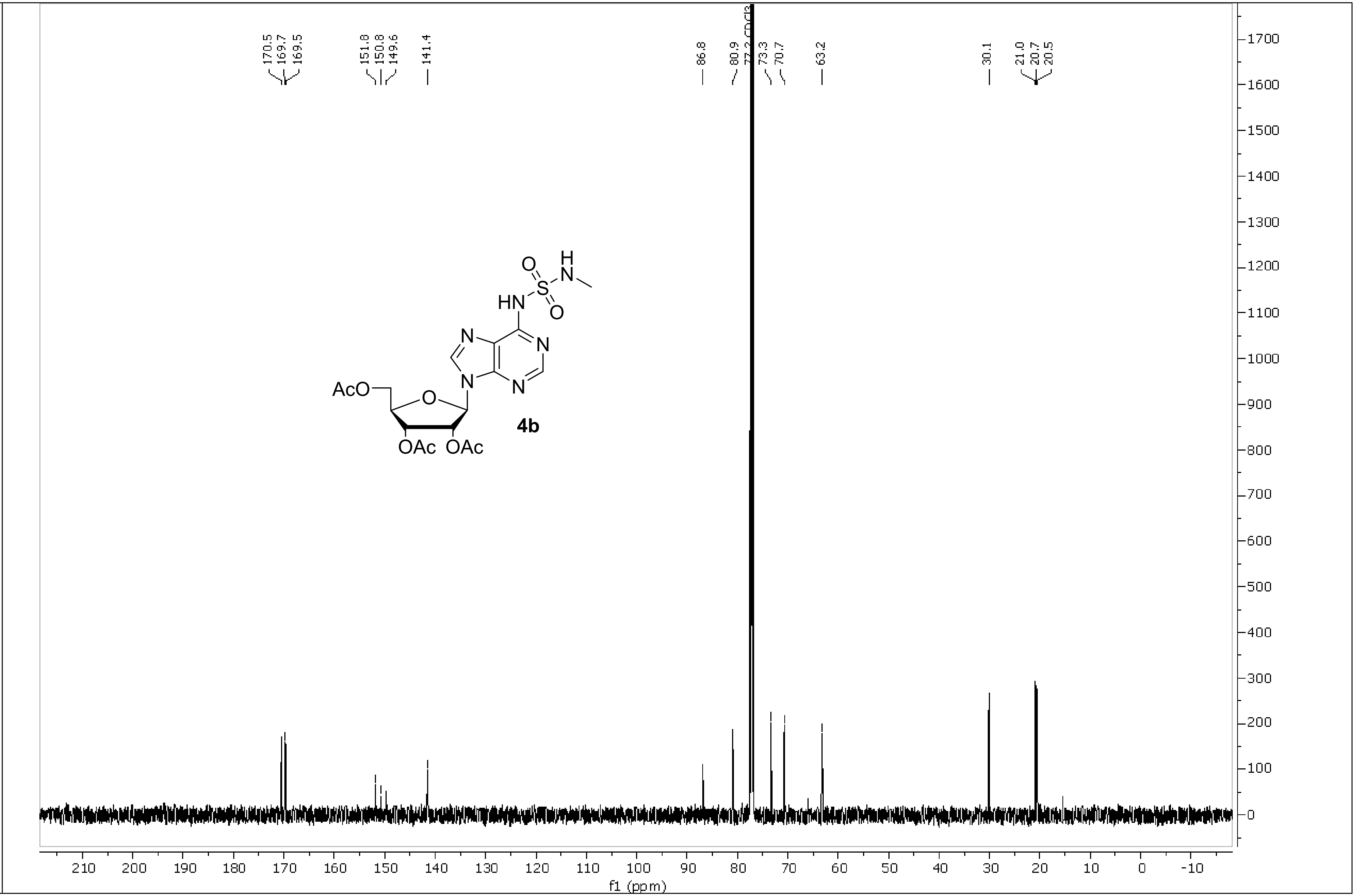

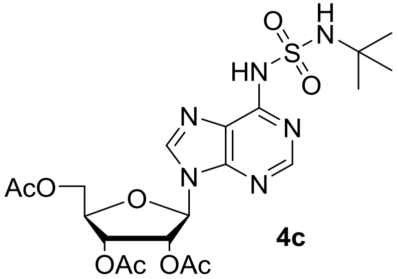

4c

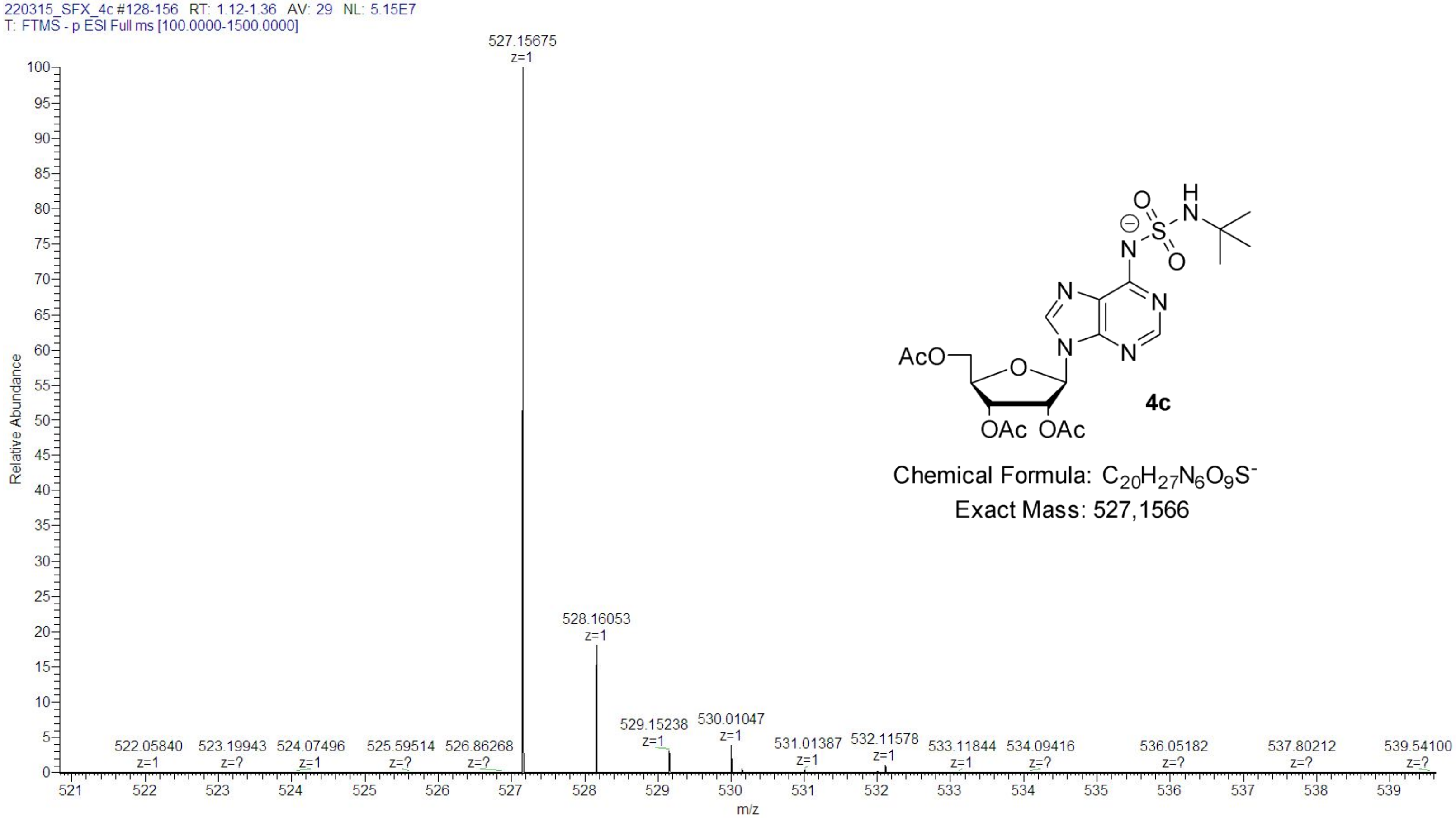

220315_SFX_4c #128-156 RT: 1.12-1.36 AV: 29 NL: 5.15E7
T: FTMS - p ESI Full ms [100.0000-1500.0000]
Relative Abundance
m/z
527.15675 z=1
528.16053 z=1
529.15238 z=1
530.01047 z=1
522.05840 z=1
523.19943 z=?
524.07496 z=1
525.59514 z=?
526.86268 z=?
531.01387 z=1
532.11578 z=1
533.11844 z=1
534.09416 z=?
536.05182 z=?
537.80212 z=?
539.54100 z=?
4c
Chemical Formula: $C_{20}H_{27}N_6O_9S^-$
Exact Mass: 527,1566

^{1}H NMR spectrum (500 MHz) of **4c**

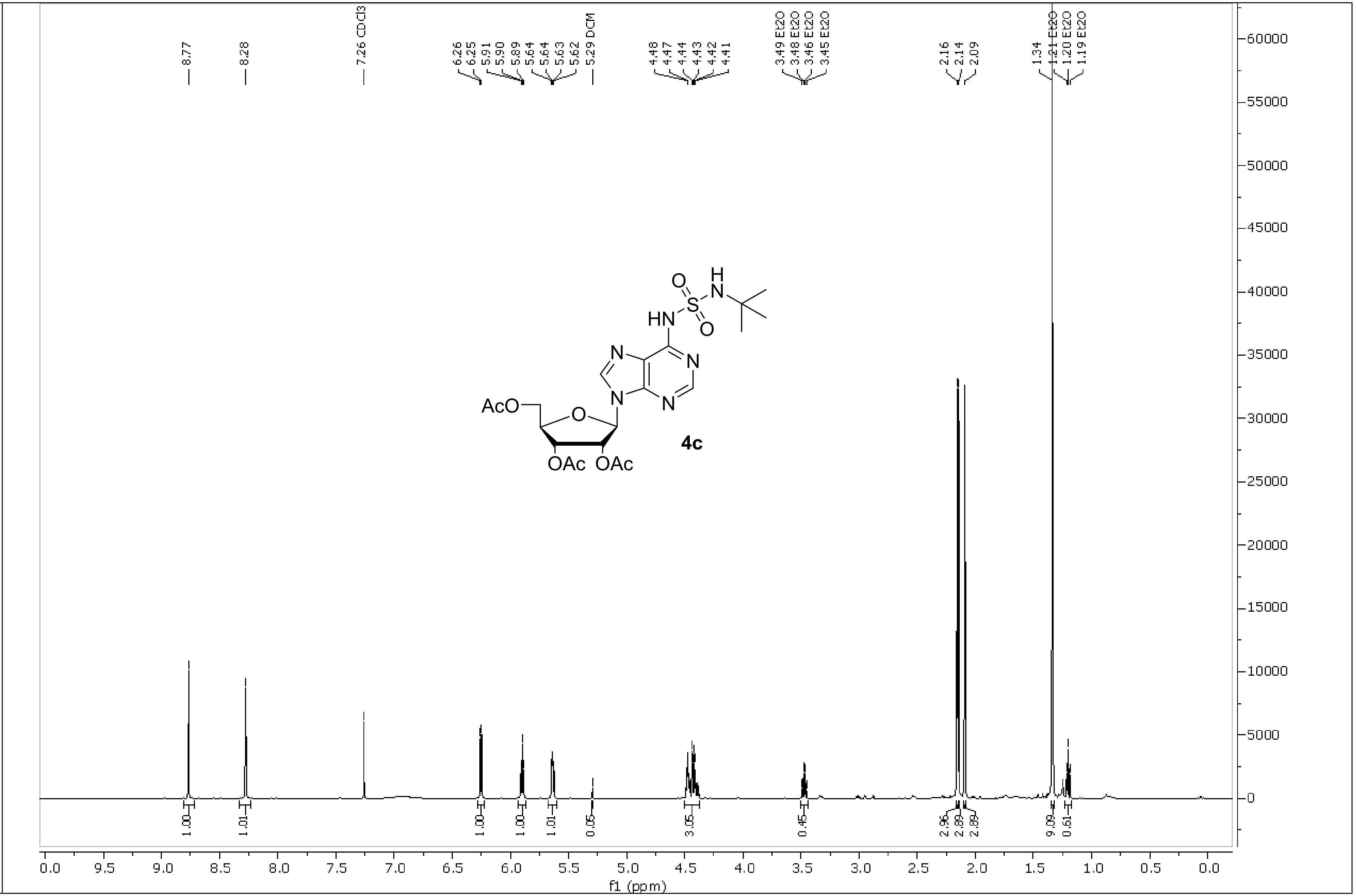

^{13}C NMR spectrum (126 MHz) of **4c**

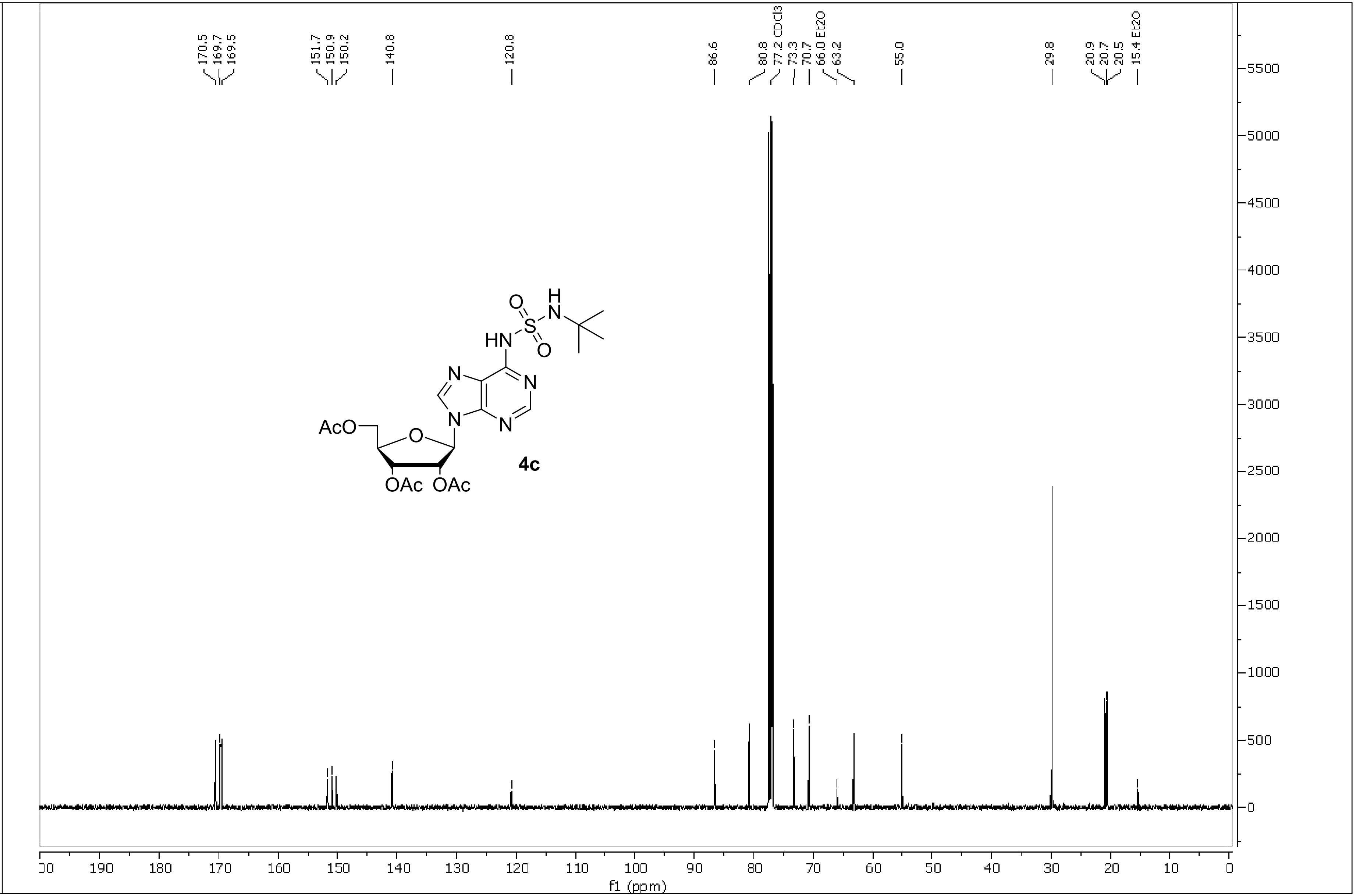

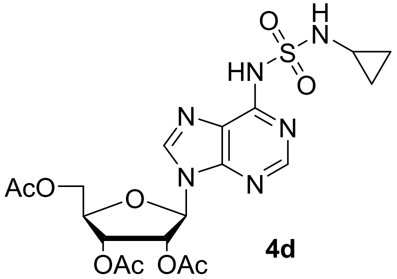

O
H
N
HN
S
O
N
N
N
N
AcO
O
OAc OAc

4d

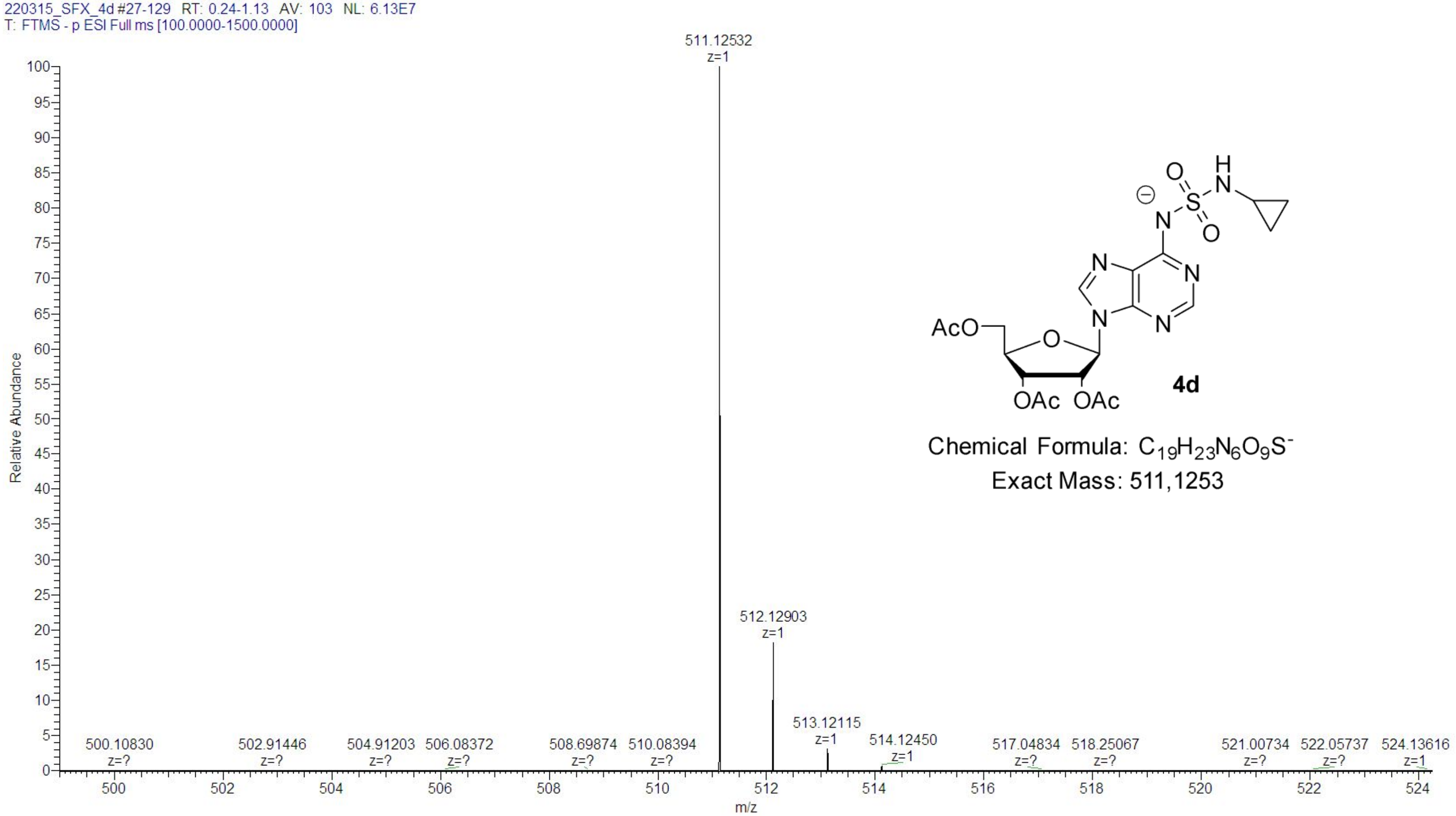

220315_SFX_4d #27-129 RT: 0.24-1.13 AV: 103 NL: 6.13E7
T: FTMS - p ESI Full ms [100.0000-1500.0000]
511.12532
z=1
512.12903
z=1
513.12115
z=1
514.12450
z=1
500.10830
z=?
502.91446
z=?
504.91203
z=?
506.08372
z=?
508.69874
z=?
510.08394
z=?
517.04834
z=?
518.25067
z=?
521.00734
z=?
522.05737
z=?
524.13616
z=1
Relative Abundance
m/z
4d
Chemical Formula: $C_{19}H_{23}N_6O_9S^-$
Exact Mass: 511,1253

^{1}H NMR spectrum (500 MHz) of **4d**

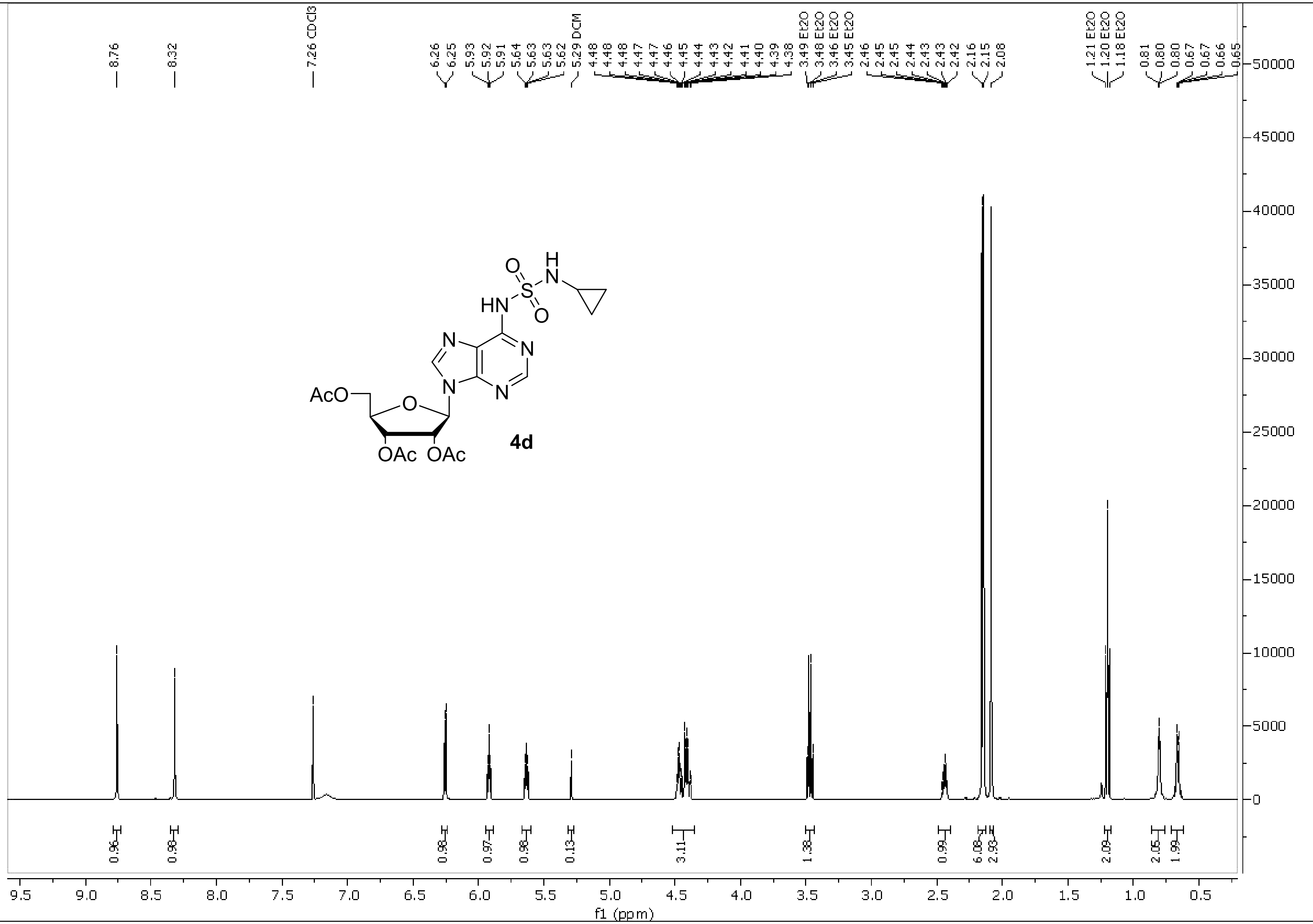

^{13}C NMR spectrum (126 MHz) of **4d**

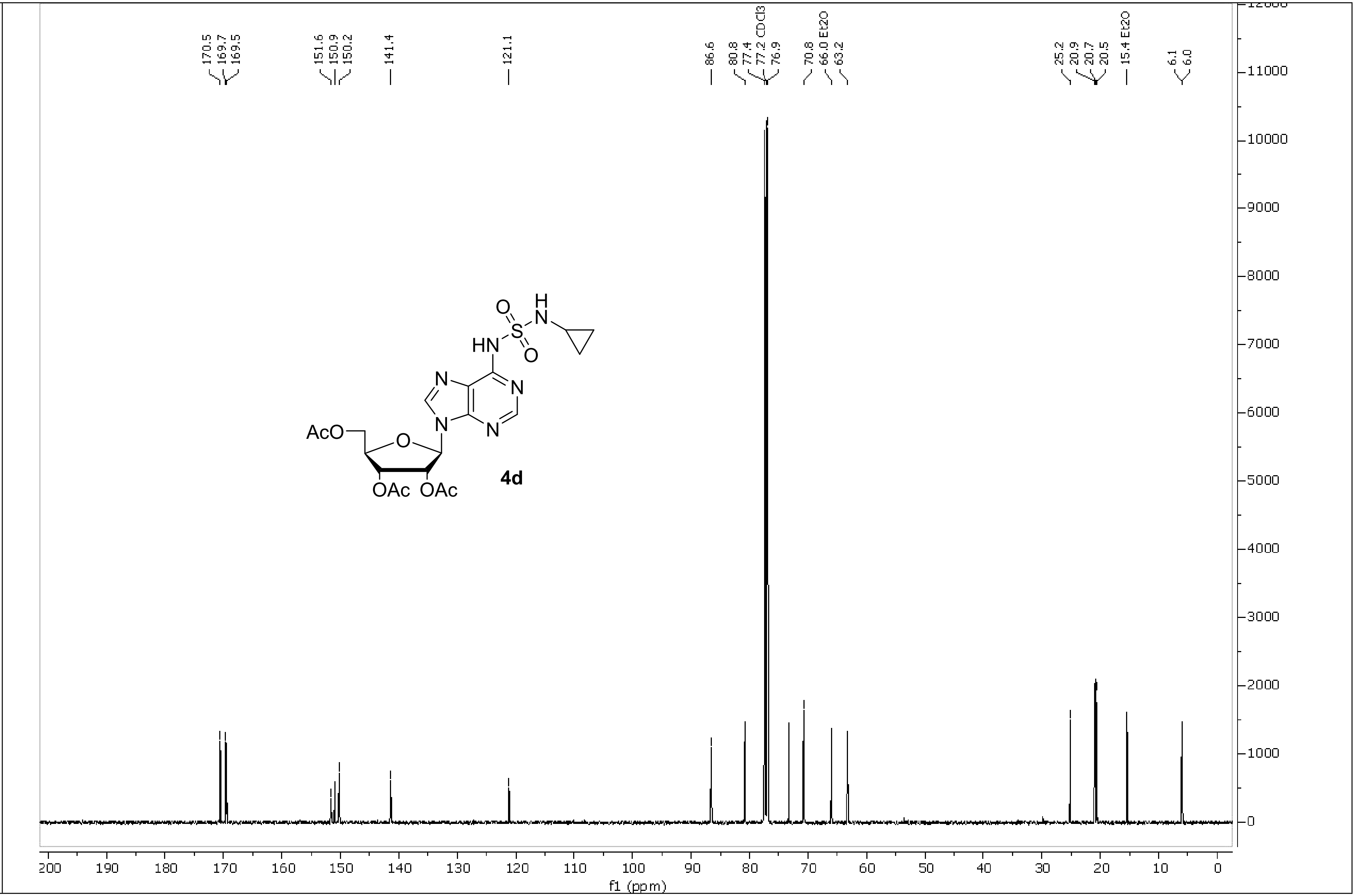

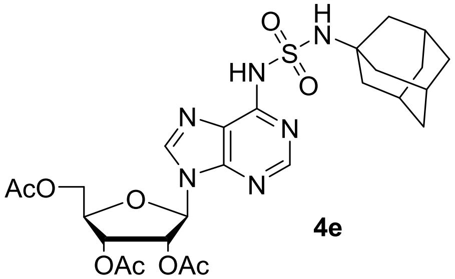

4e

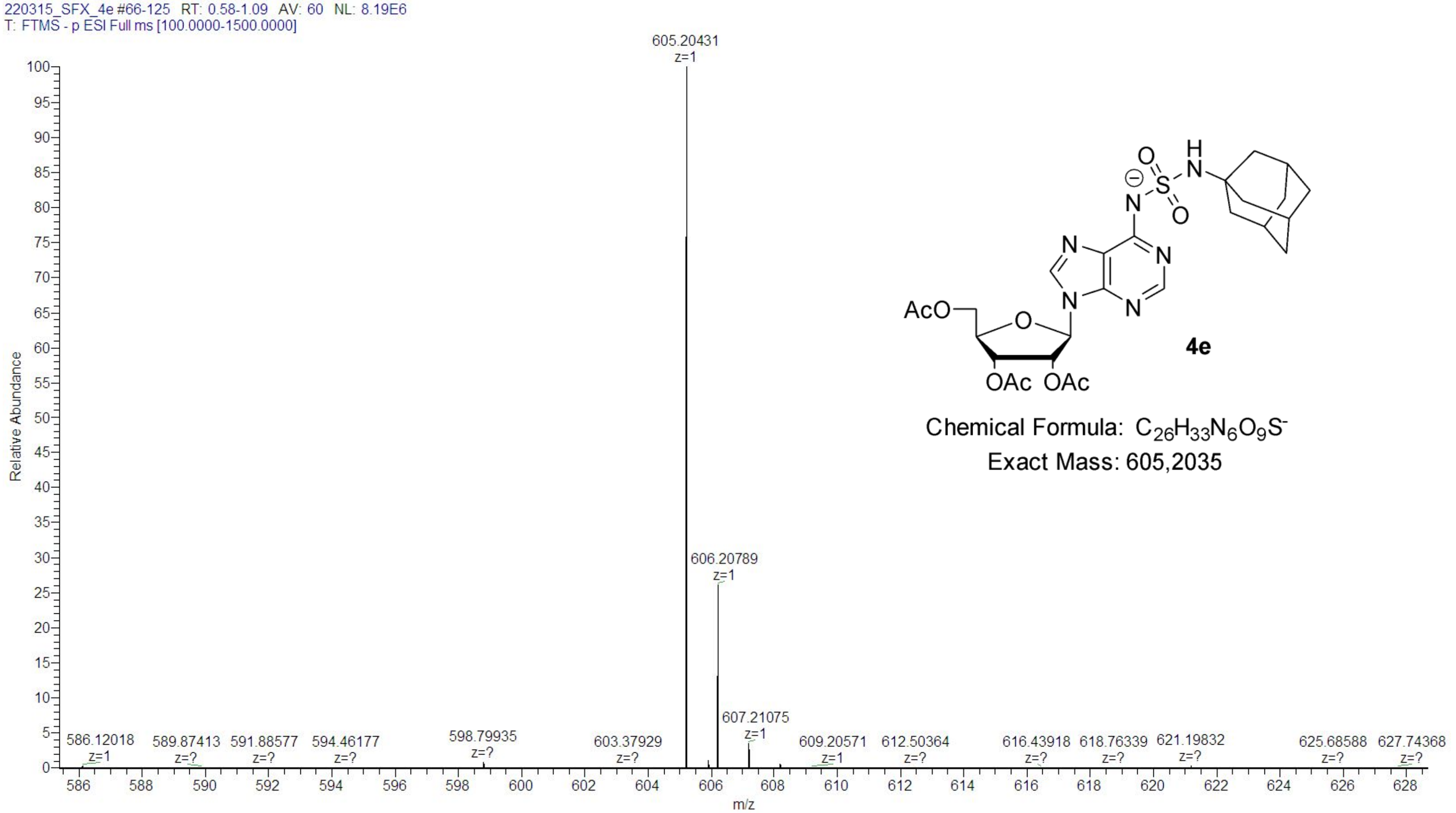

Chemical Formula: $C_{26}H_{33}N_6O_9S^-$
Exact Mass: 605,2035

^{1}H NMR spectrum (500 MHz) of **4e**

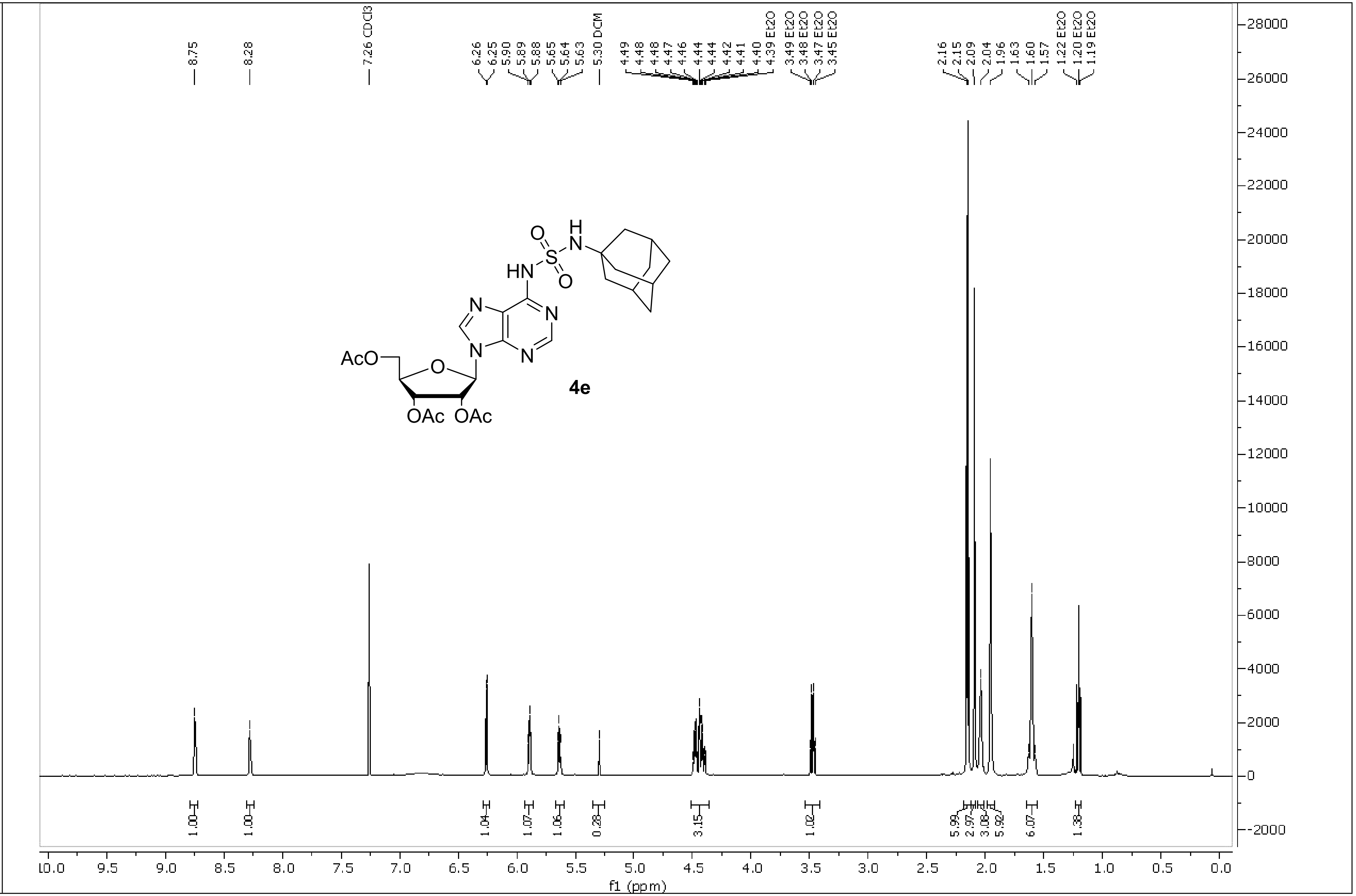

^{13}C NMR spectrum (126 MHz) of **4e**

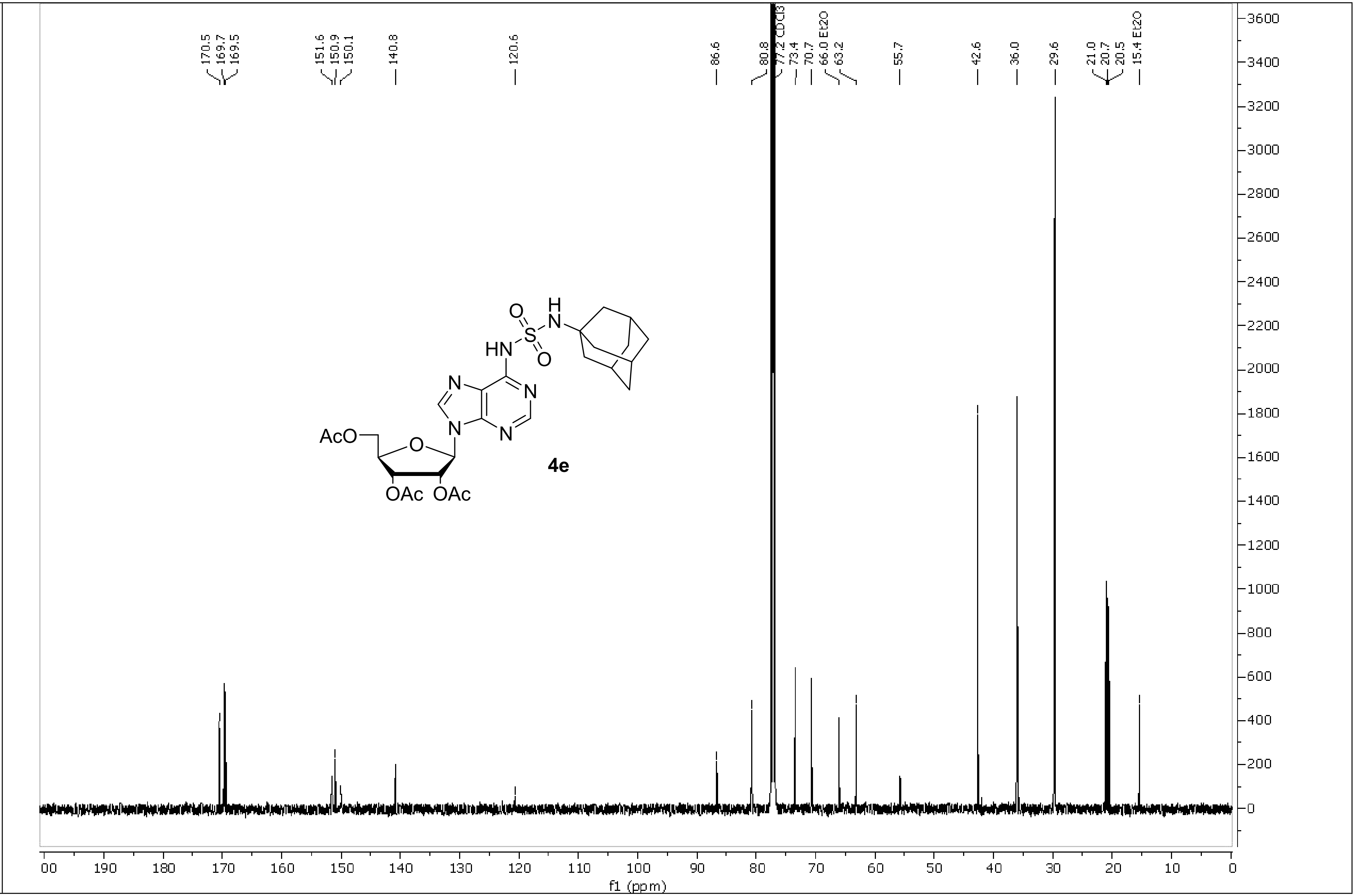

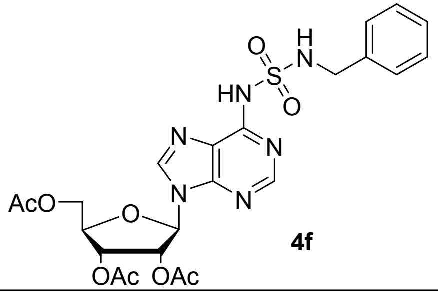

4f

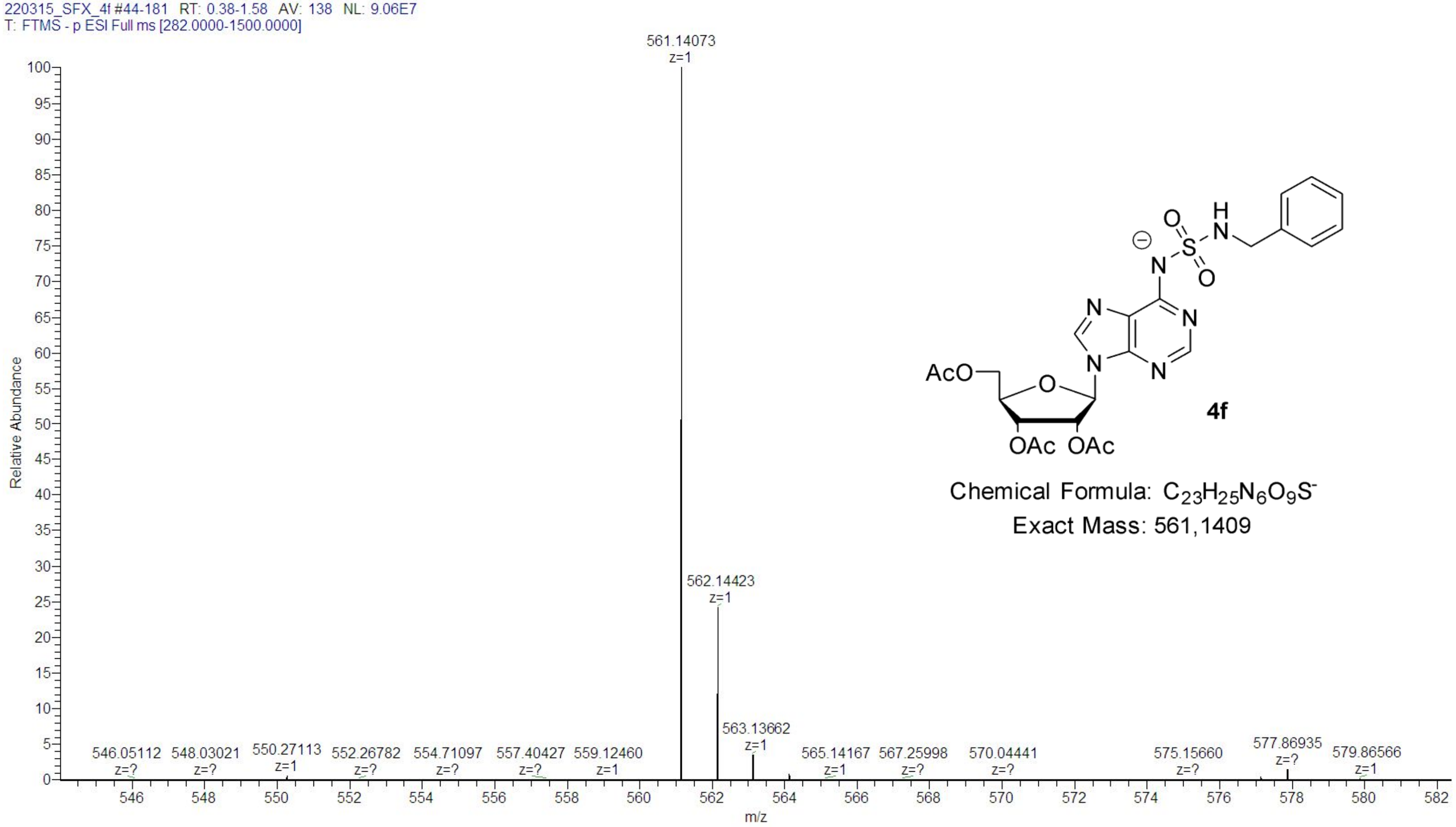
220315_SFX_4f #44-181 RT: 0.38-1.58 AV: 138 NL: 9.06E7
T: FTMS - p ESI Full ms [282.0000-1500.0000]
561.14073 z=1
562.14423 z=1
563.13662 z=1
546.05112 z=?
548.03021 z=?
550.27113 z=1
552.26782 z=?
554.71097 z=?
557.40427 z=?
559.12460 z=1
565.14167 z=1
567.25998 z=?
570.04441 z=?
575.15660 z=?
577.86935 z=?
579.86566 z=1
Relative Abundance
m/z
4f
Chemical Formula: $C_{23}H_{25}N_6O_9S^-$
Exact Mass: 561,1409

^{1}H NMR spectrum (500 MHz) of **4f**

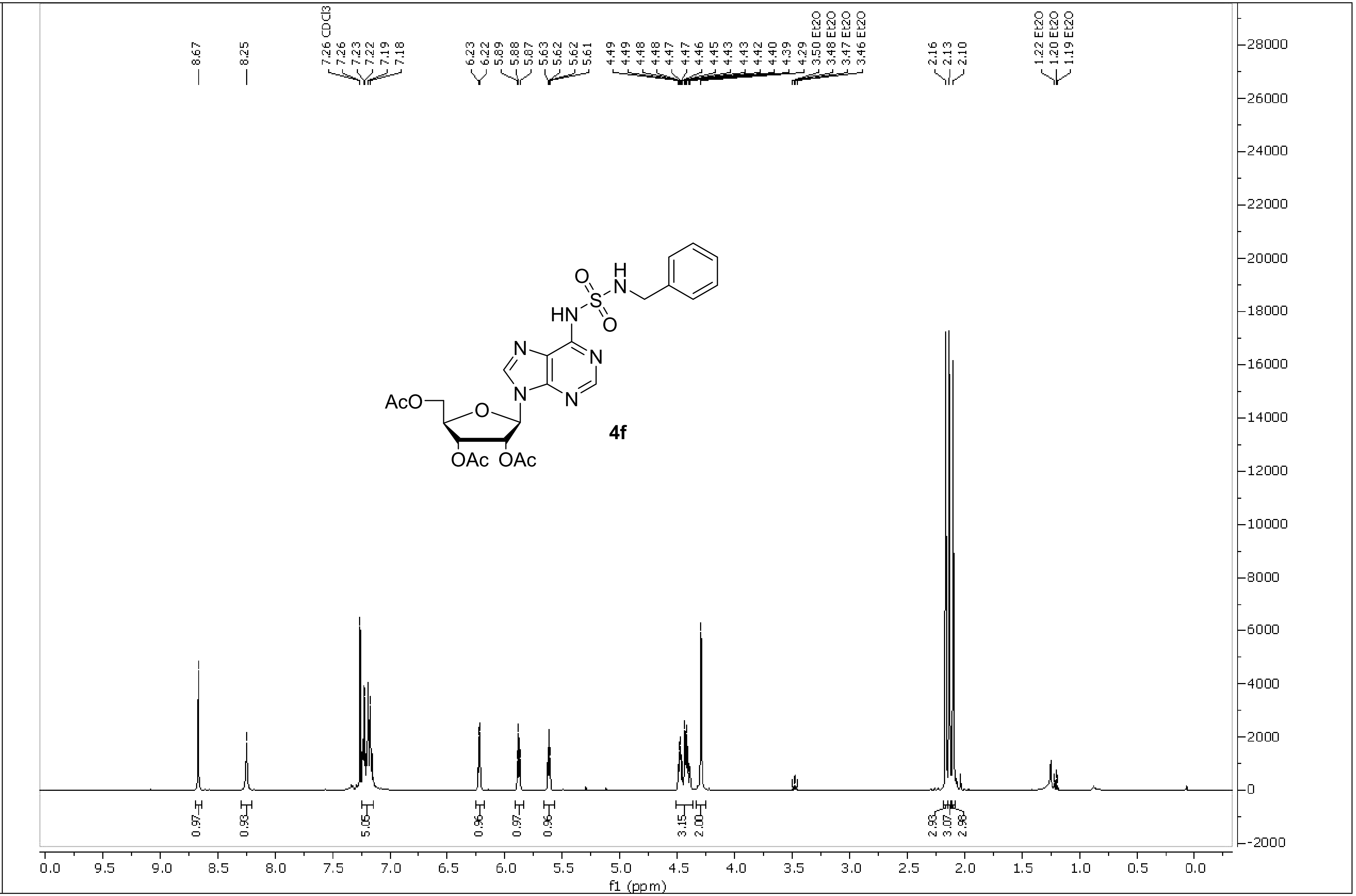

^{13}C NMR spectrum (126 MHz) of **4f**

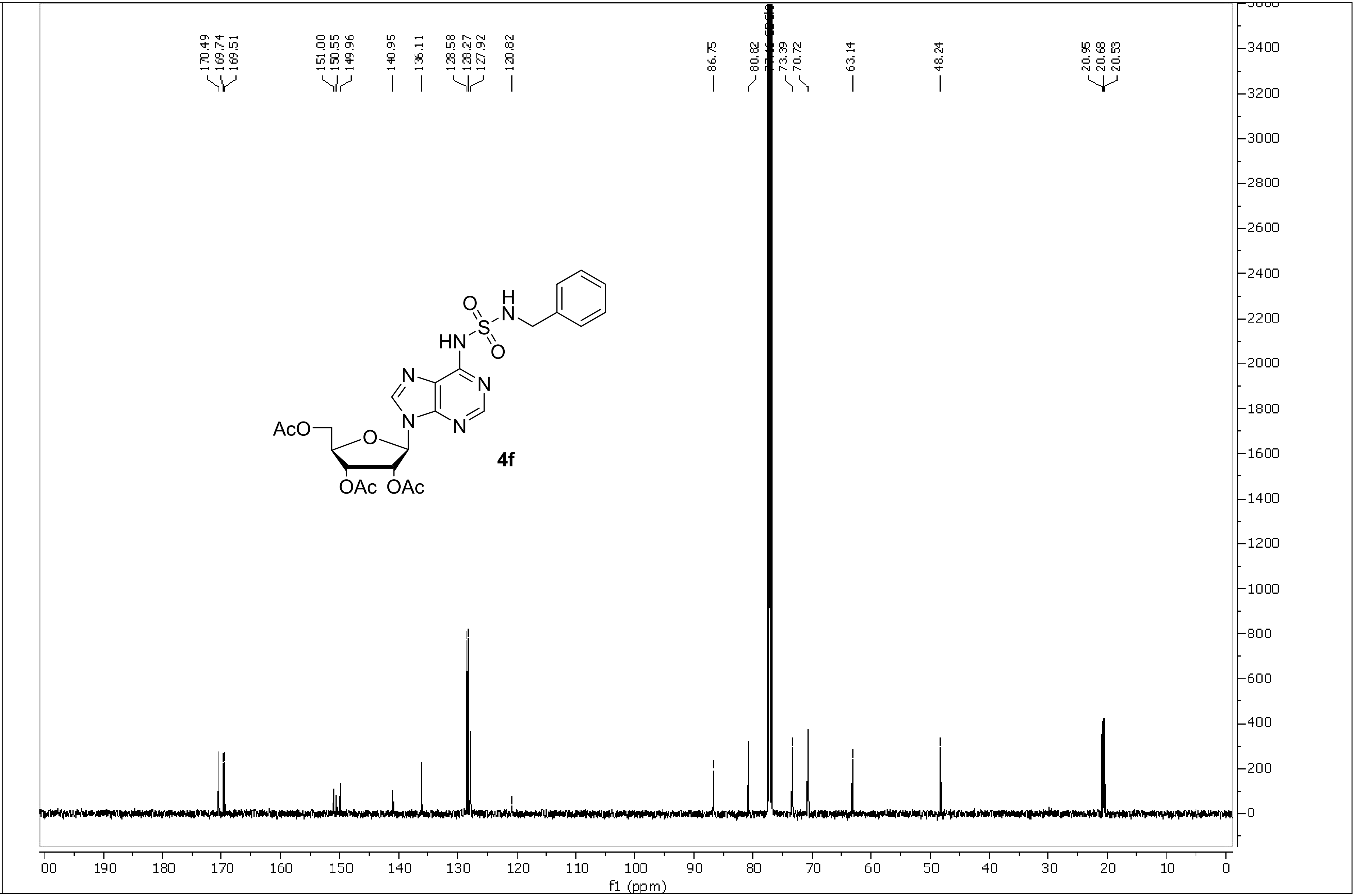

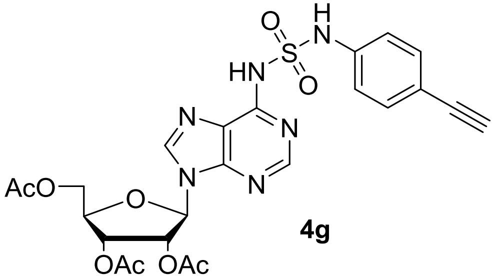

4g

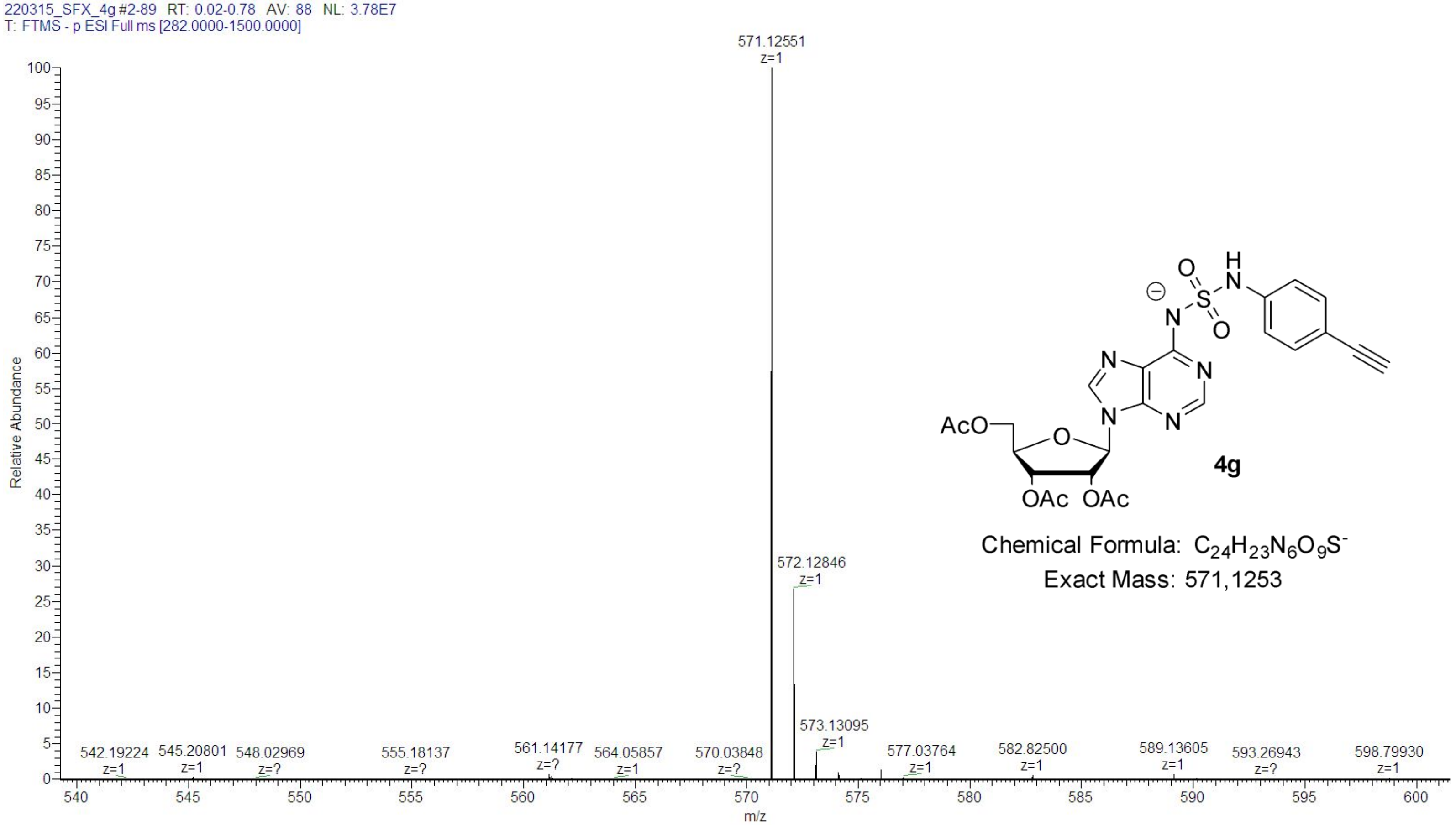

220315_SFX_4g #2-89 RT: 0.02-0.78 AV: 88 NL: 3.78E7
T: FTMS - p ESI Full ms [282.0000-1500.0000]
571.12551 z=1
572.12846 z=1
573.13095 z=1
542.19224 z=1
545.20801 z=1
548.02969 z=?
555.18137 z=?
561.14177 z=?
564.05857 z=1
570.03848 z=?
577.03764 z=1
582.82500 z=1
589.13605 z=1
593.26943 z=?
598.79930 z=1
Relative Abundance
m/z
4g
Chemical Formula: C24H23N6O9S-
Exact Mass: 571,1253

^1H NMR spectrum (500 MHz) of **4g**

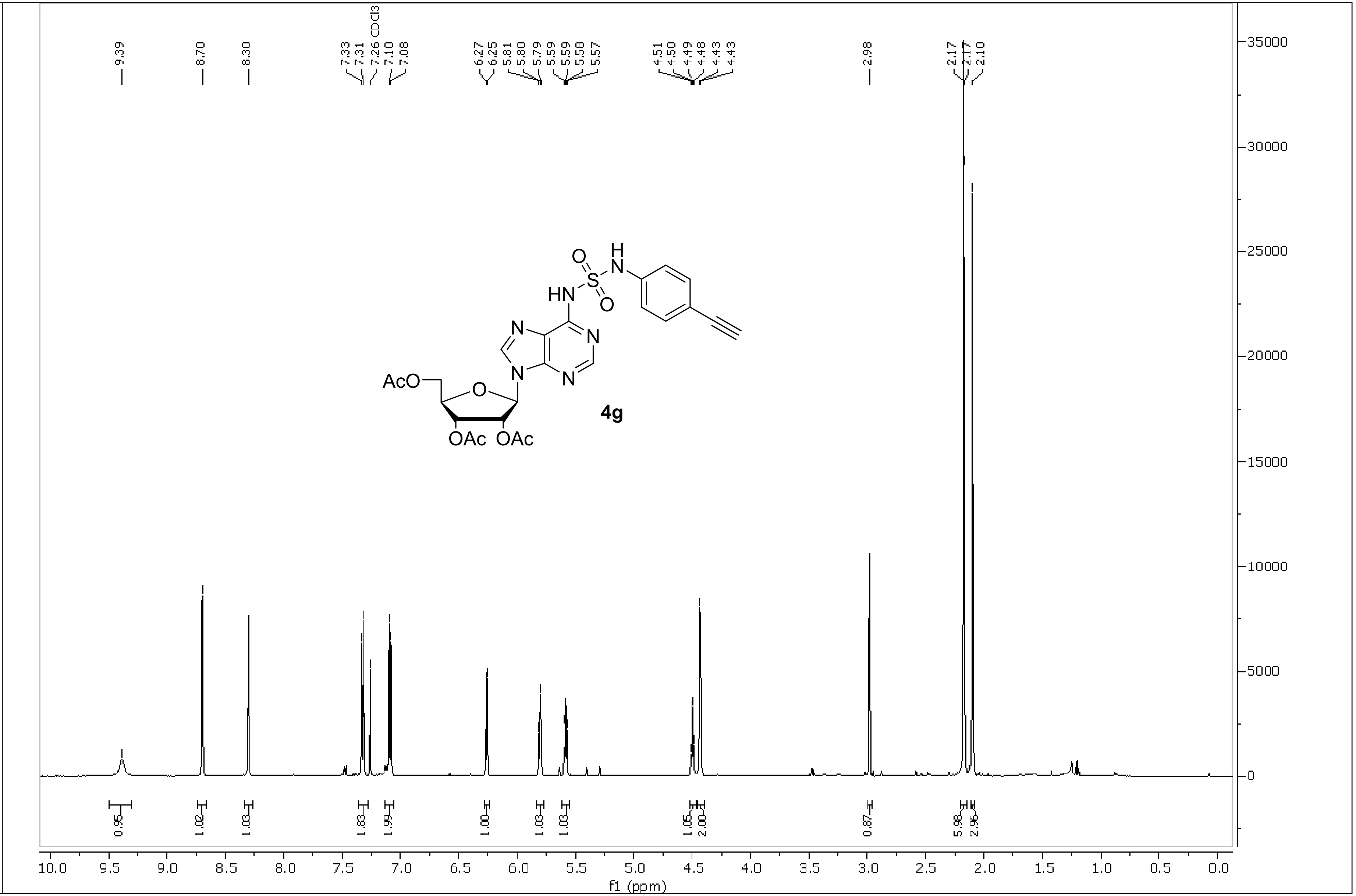

^{13}C NMR spectrum (126 MHz) of **4g**

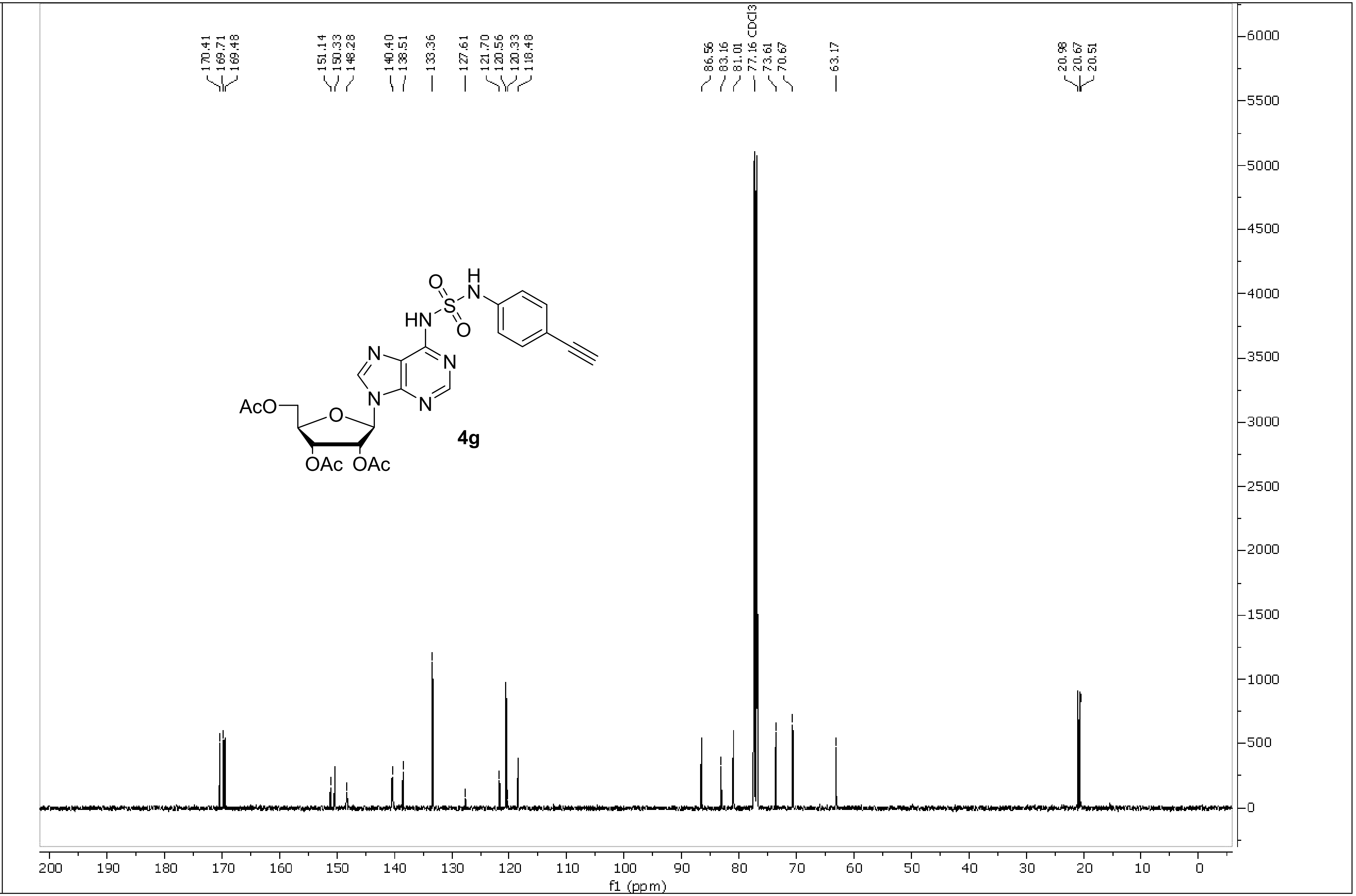

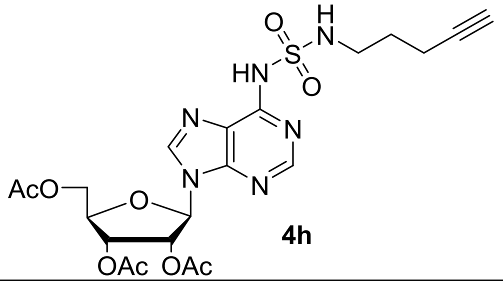

AcO
OAc
OAc

4h

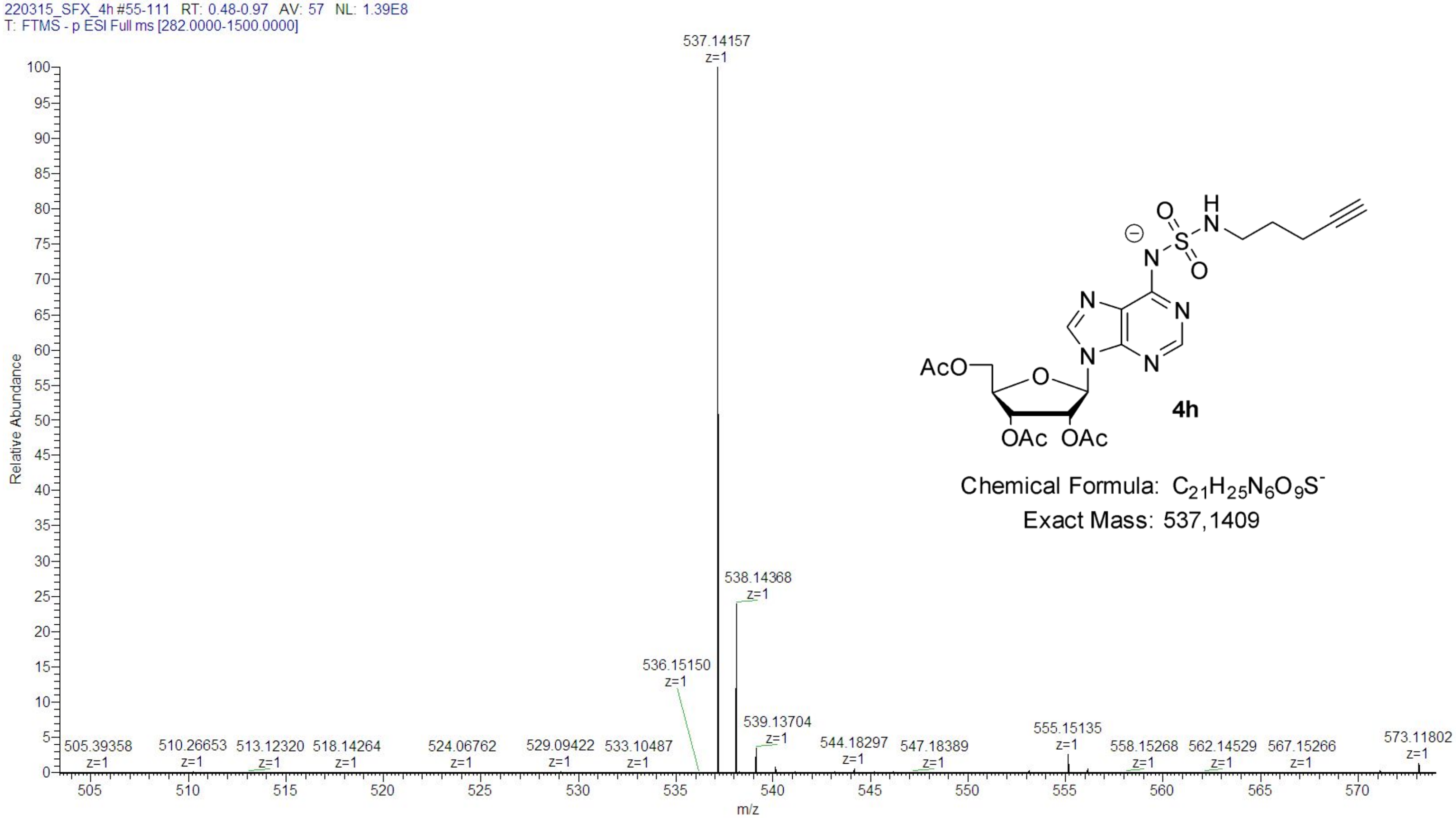

220315_SFX_4h #55-111 RT: 0.48-0.97 AV: 57 NL: 1.39E8
T: FTMS - p ESI Full ms [282.0000-1500.0000]
537.14157 z=1
538.14368 z=1
536.15150 z=1
539.13704 z=1
505.39358 z=1
510.26653 z=1
513.12320 z=1
518.14264 z=1
524.06762 z=1
529.09422 z=1
533.10487 z=1
544.18297 z=1
547.18389 z=1
555.15135 z=1
558.15268 z=1
562.14529 z=1
567.15266 z=1
573.11802 z=1
Relative Abundance
m/z
4h
Chemical Formula: $C_{21}H_{25}N_6O_9S^-$
Exact Mass: 537,1409

^{1}H NMR spectrum (500 MHz) of **4h**

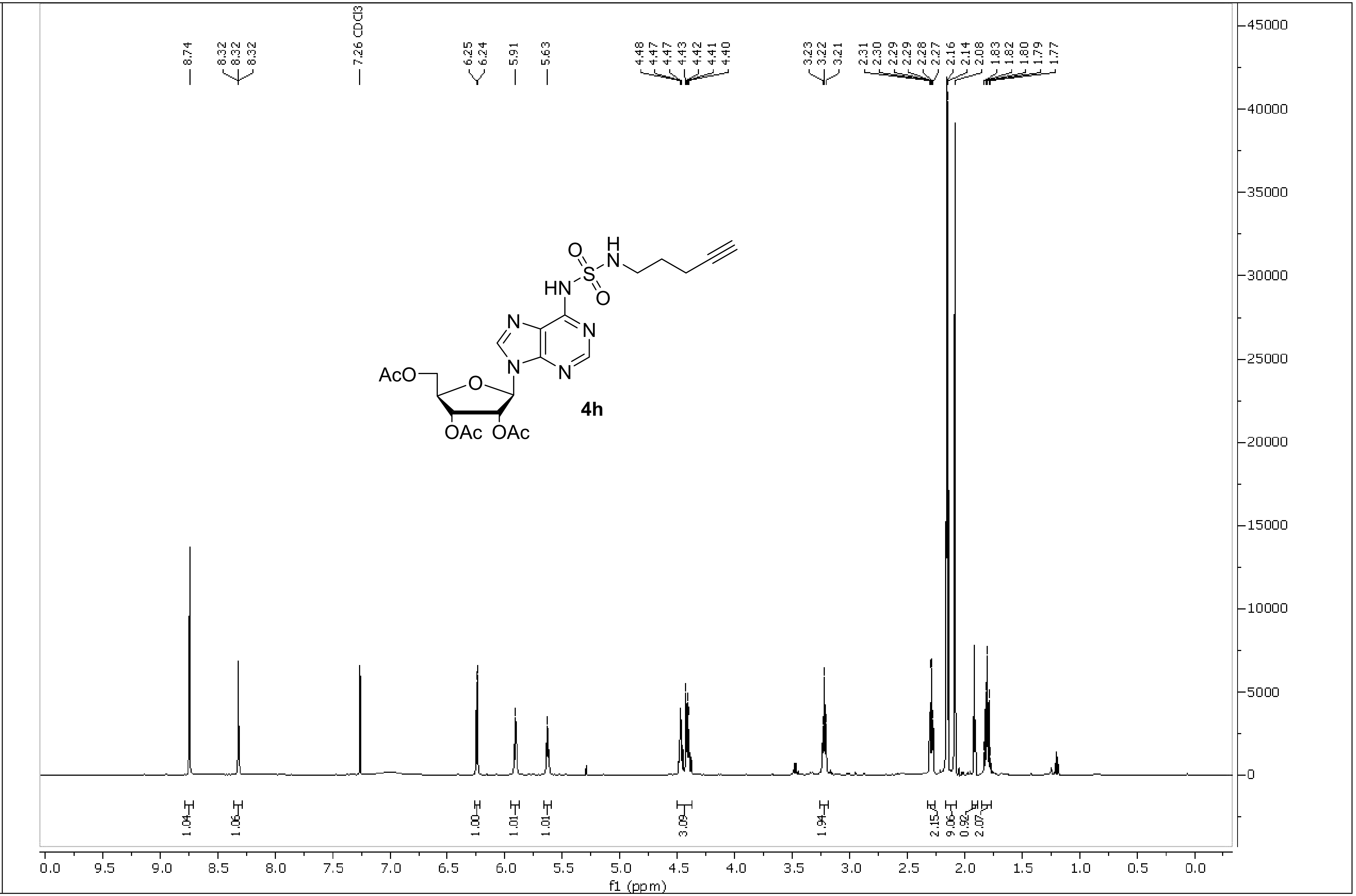

^{13}C NMR spectrum (126 MHz) of **4h**

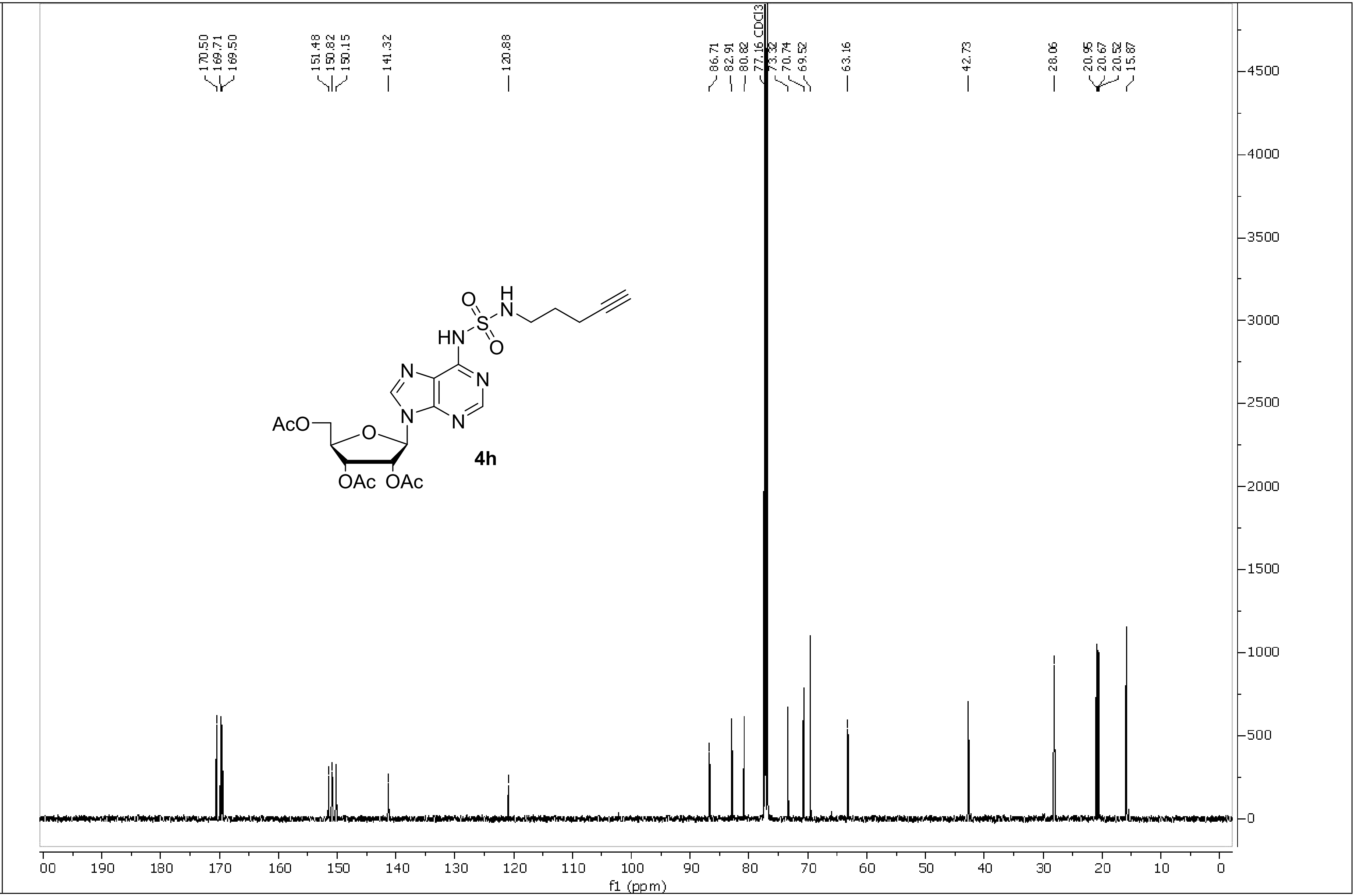

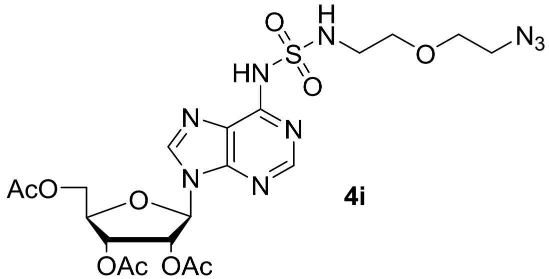

4i

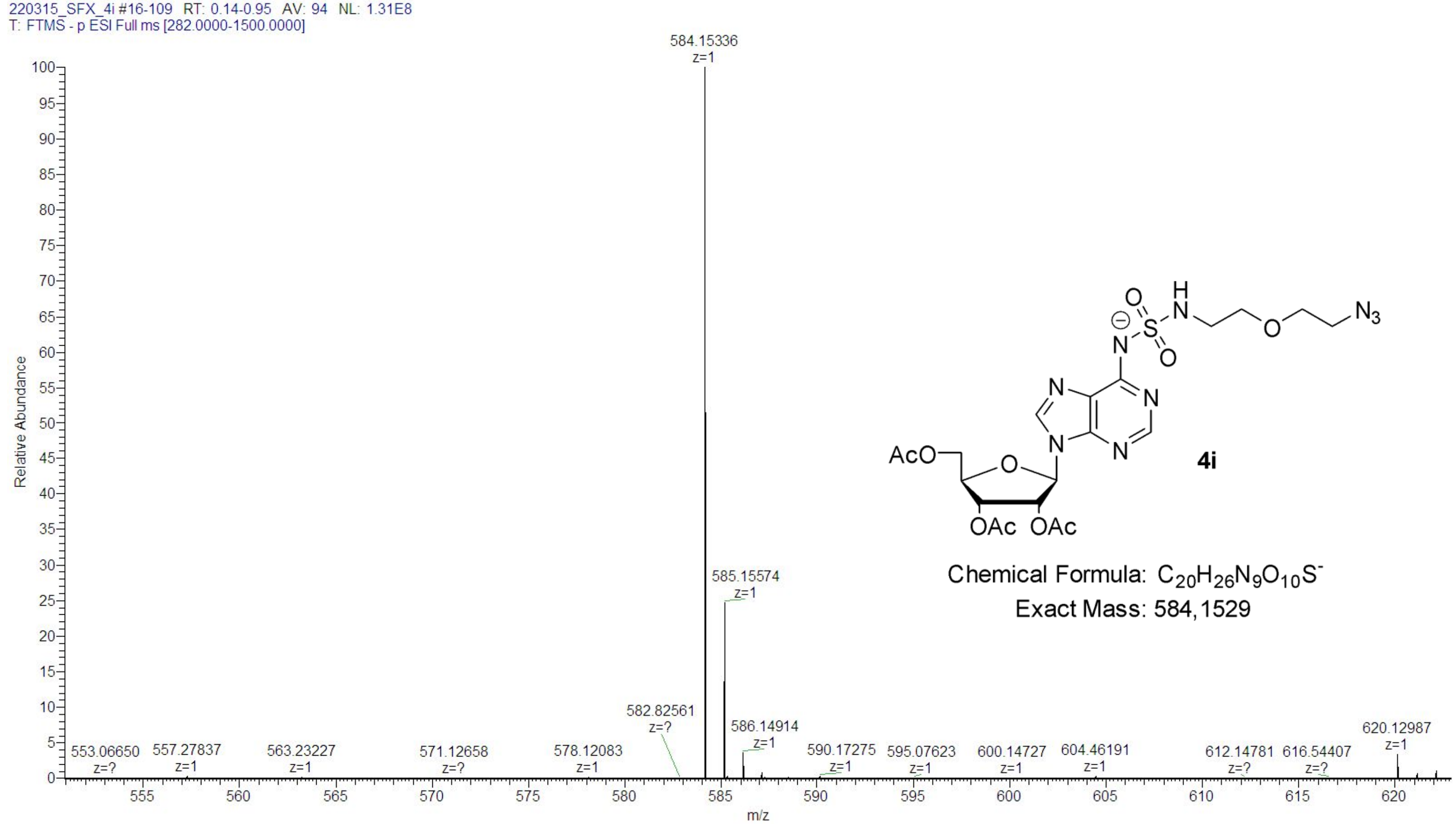

220315_SFX_4i #16-109 RT: 0.14-0.95 AV: 94 NL: 1.31E8
T: FTMS - p ESI Full ms [282.0000-1500.0000]
584.15336 z=1
585.15574 z=1
586.14914 z=1
582.82561 z=?
553.06650 z=?
557.27837 z=1
563.23227 z=1
571.12658 z=?
578.12083 z=1
590.17275 z=1
595.07623 z=1
600.14727 z=1
604.46191 z=1
612.14781 z=?
616.54407 z=?
620.12987 z=1
Relative Abundance
m/z
4i
Chemical Formula: $C_{20}H_{26}N_9O_{10}S^-$
Exact Mass: 584,1529

^1H NMR spectrum (500 MHz) of **4i**

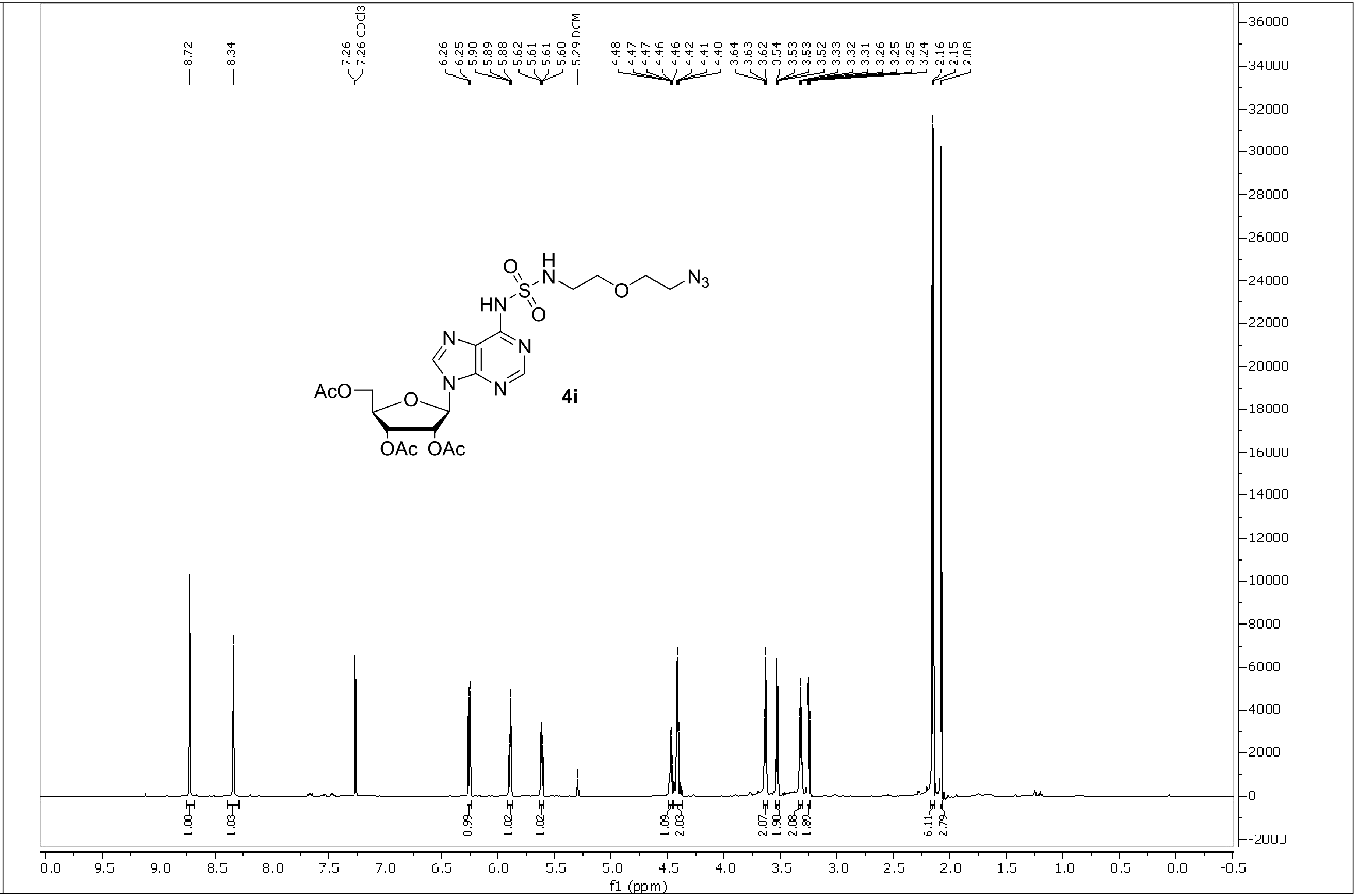

^{13}C NMR spectrum (126 MHz) of **4i**

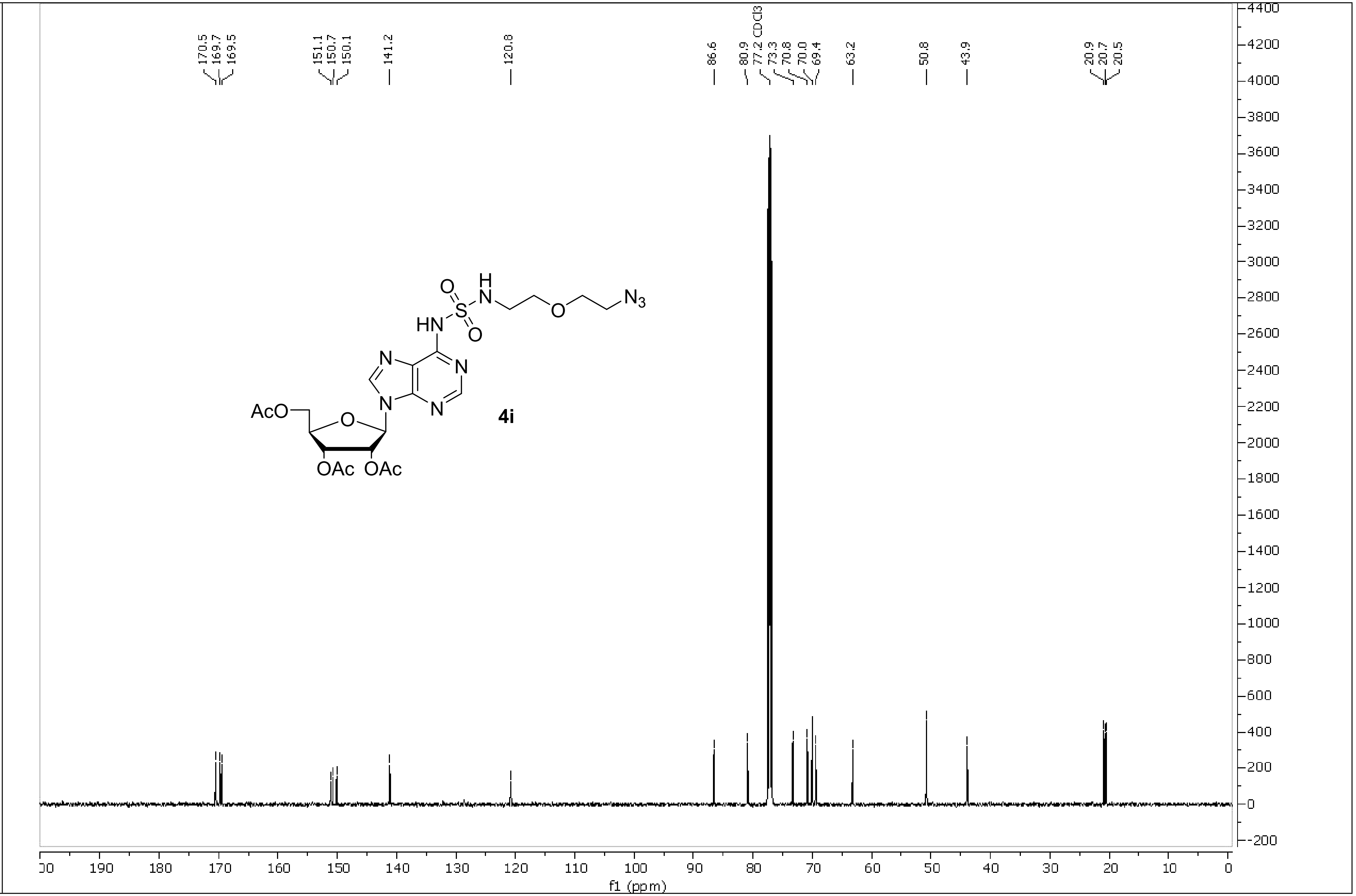

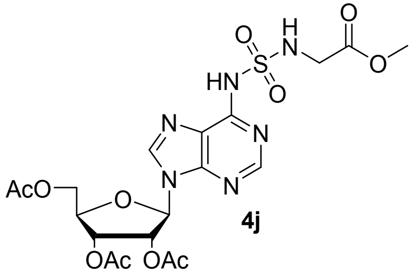

HN
N
N
N
N
O
O
S
N
H
O
O
AcO
O
OAc
OAc
4j

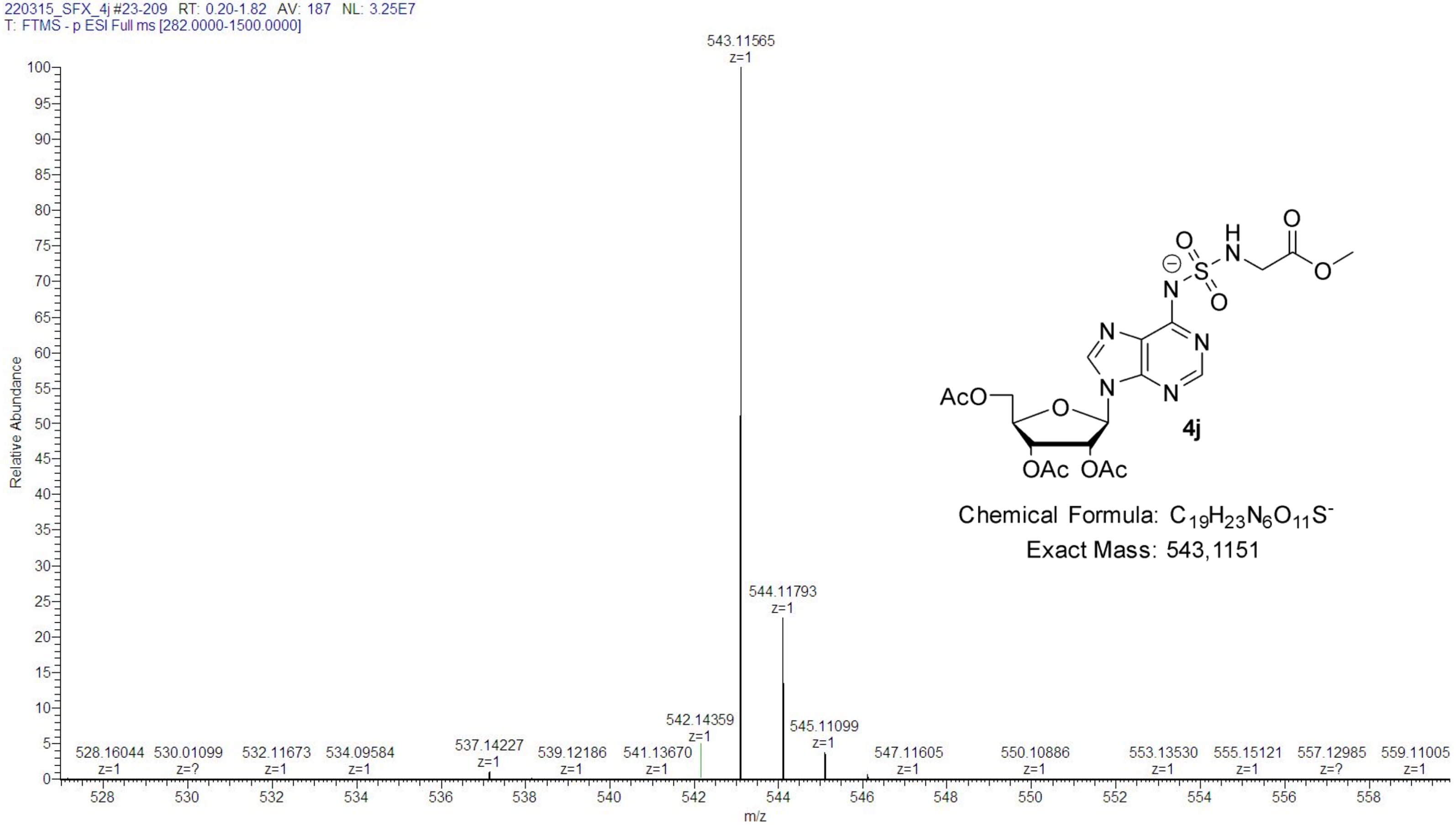

220315_SFX_4j #23-209 RT: 0.20-1.82 AV: 187 NL: 3.25E7
T: FTMS - p ESI Full ms [282.0000-1500.0000]
543.11565
z=1
544.11793
z=1
542.14359
z=1
545.11099
z=1
528.16044
z=1
530.01099
z=?
532.11673
z=1
534.09584
z=1
537.14227
z=1
539.12186
z=1
541.13670
z=1
547.11605
z=1
550.10886
z=1
553.13530
z=1
555.15121
z=1
557.12985
z=?
559.11005
z=1
Relative Abundance
m/z
4j
Chemical Formula: $C_{19}H_{23}N_6O_{11}S^-$
Exact Mass: 543,1151

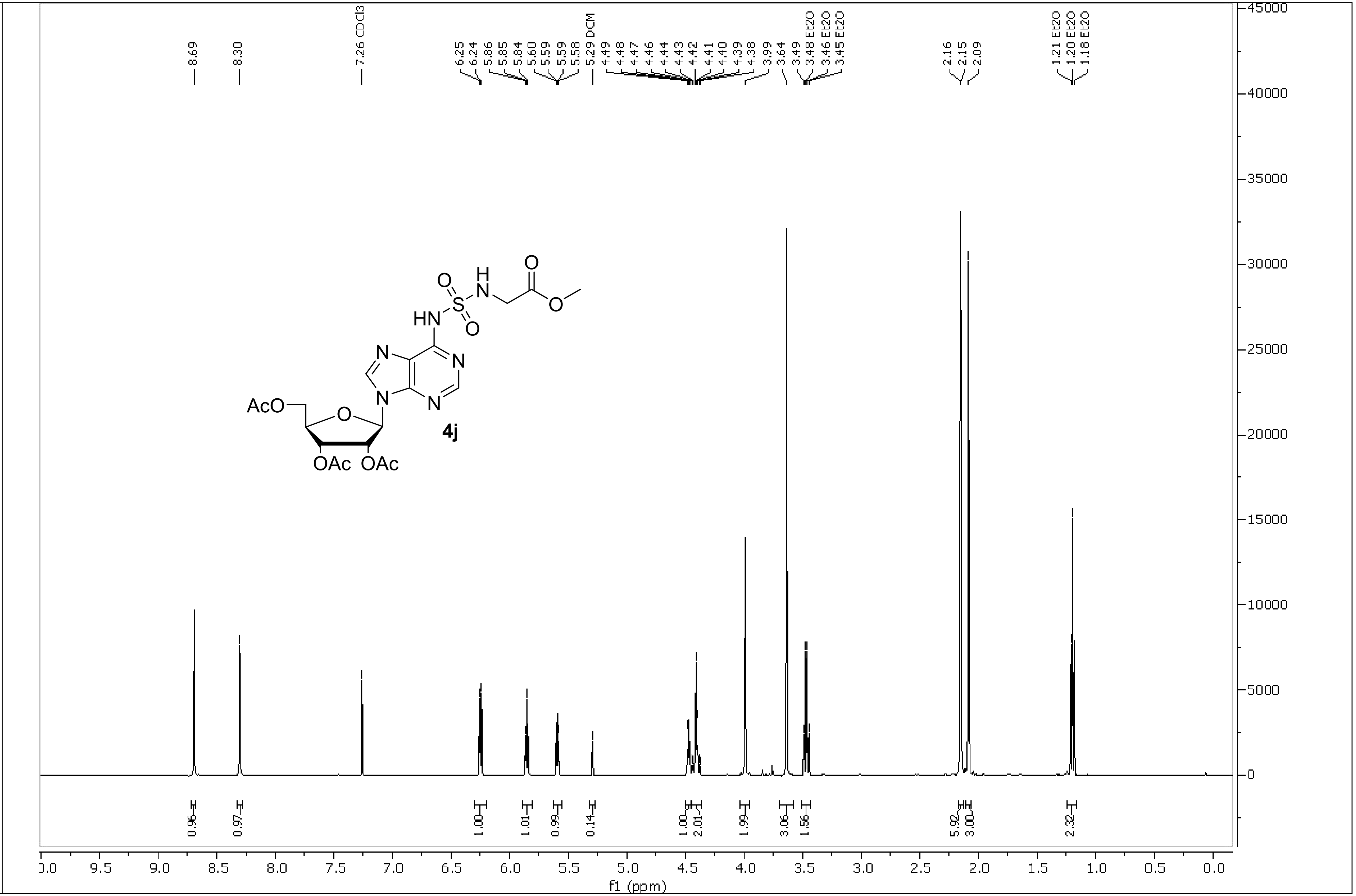

^{1}H NMR spectrum (500 MHz) of **4j**

^{13}C NMR spectrum (126 MHz) of **4j**

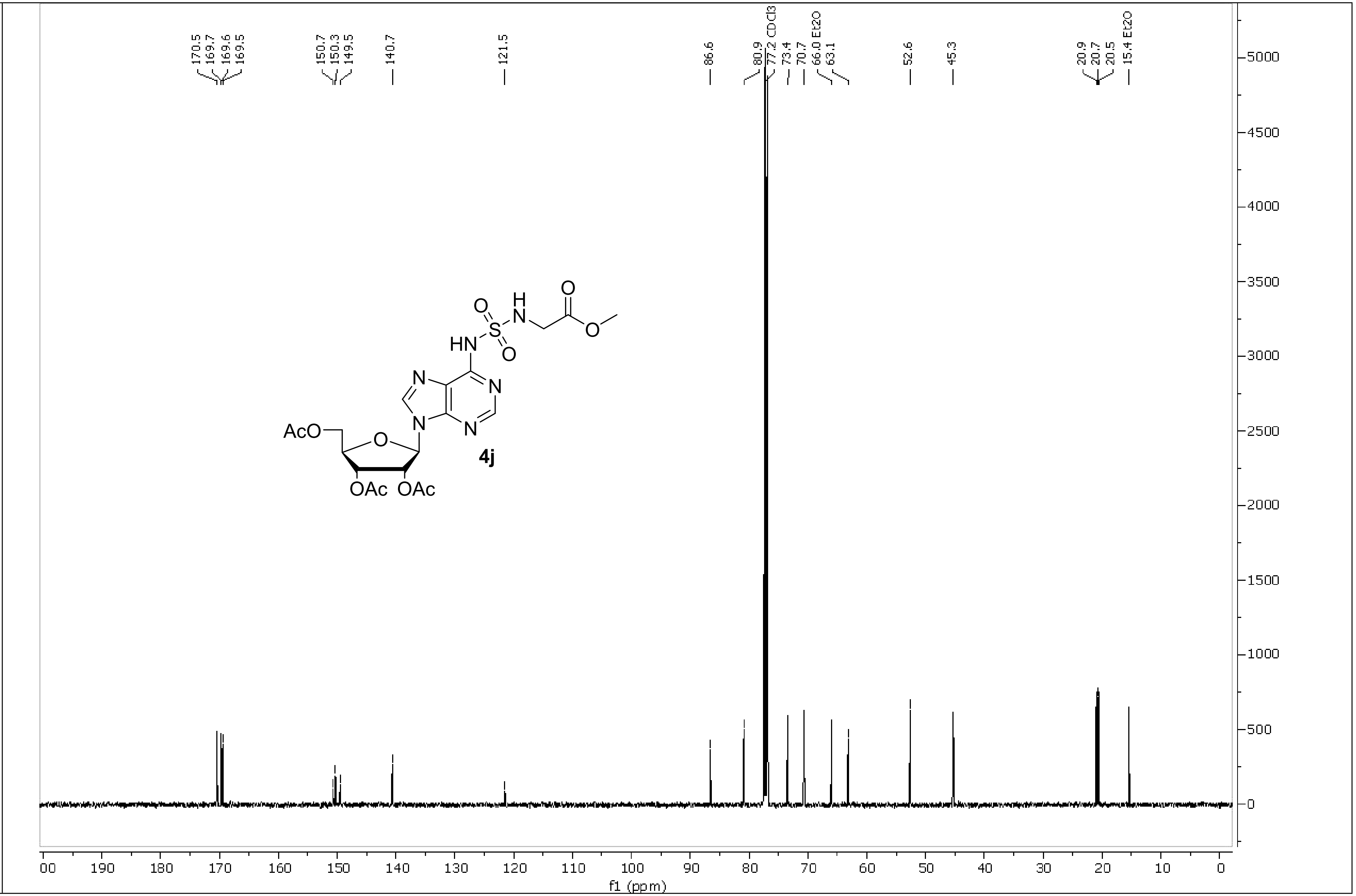

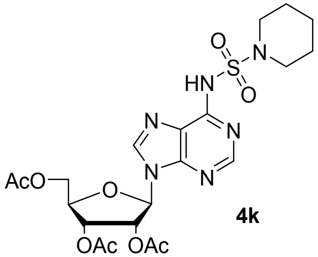

AcO
OAc OAc
HN

4k

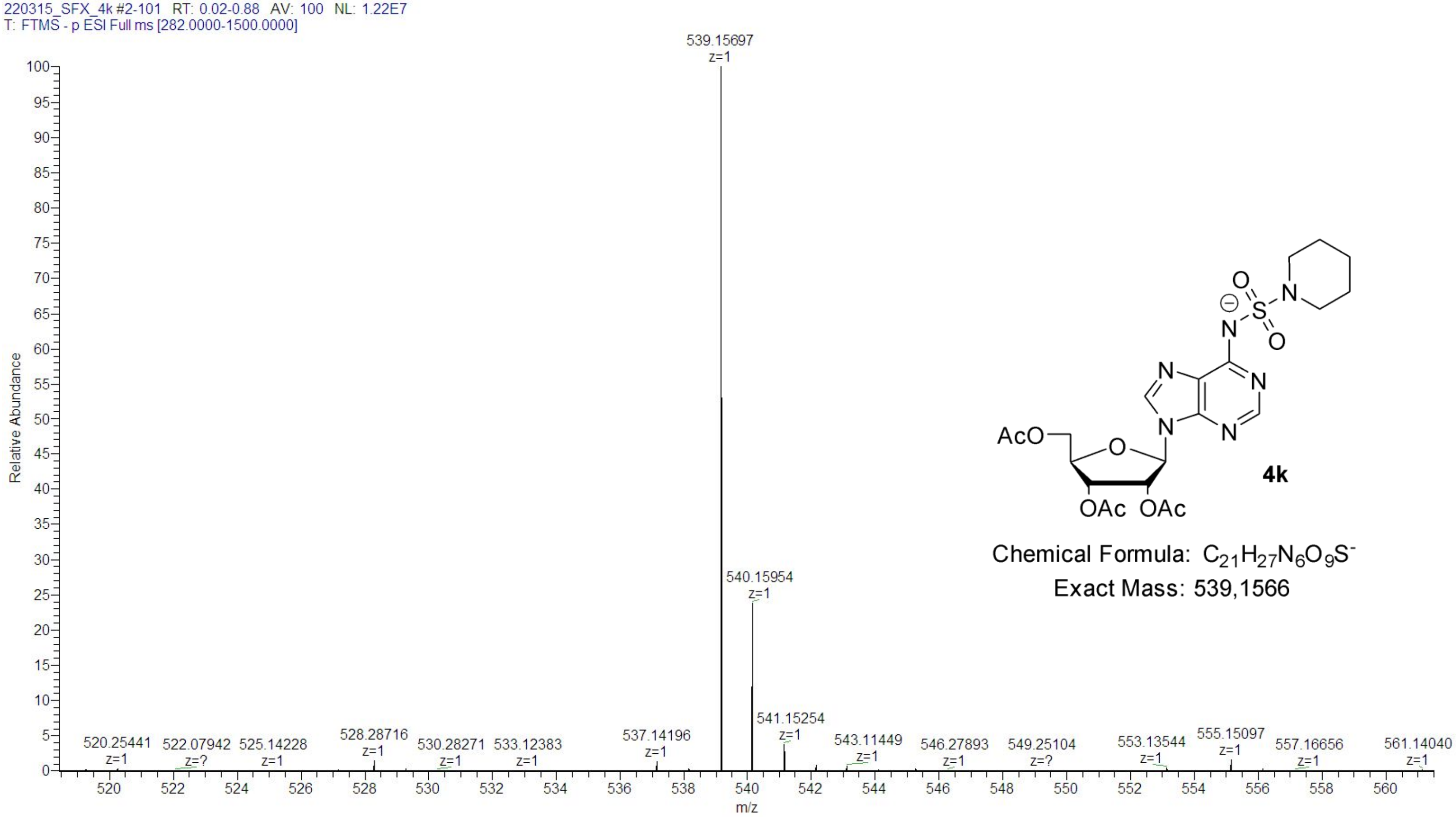

220315_SFX_4k #2-101 RT: 0.02-0.88 AV: 100 NL: 1.22E7
T: FTMS - p ESI Full ms [282.0000-1500.0000]
539.15697
540.15954
541.15254
543.11449
546.27893
549.25104
553.13544
555.15097
557.16656
561.14040
537.14196
533.12383
530.28271
528.28716
525.14228
522.07942
520.25441
Relative Abundance
m/z
4k
Chemical Formula: C21H27N6O9S-
Exact Mass: 539,1566

^{1}H NMR spectrum (500 MHz) of **4k**

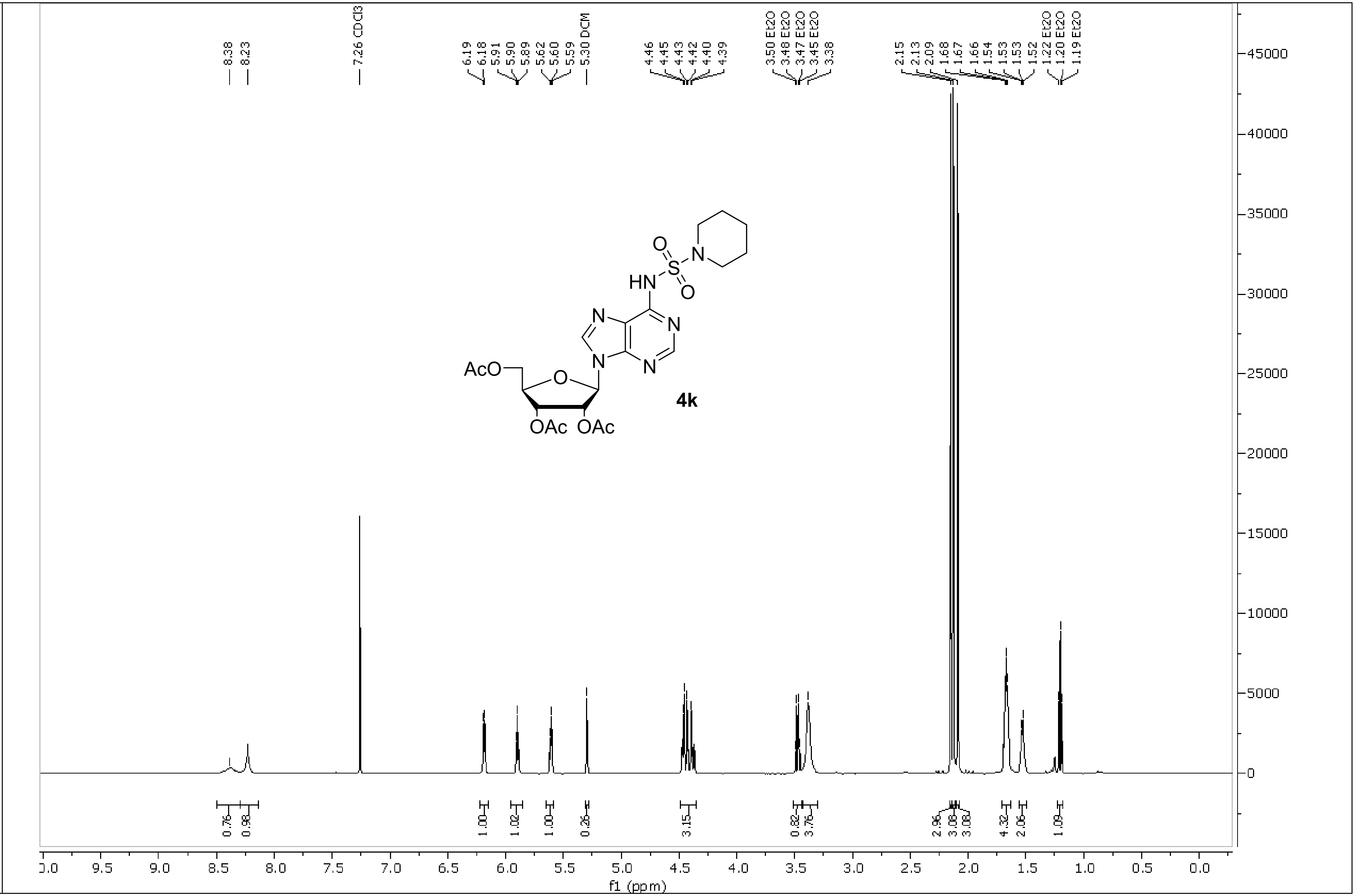

^{13}C NMR spectrum (126 MHz) of **4k**

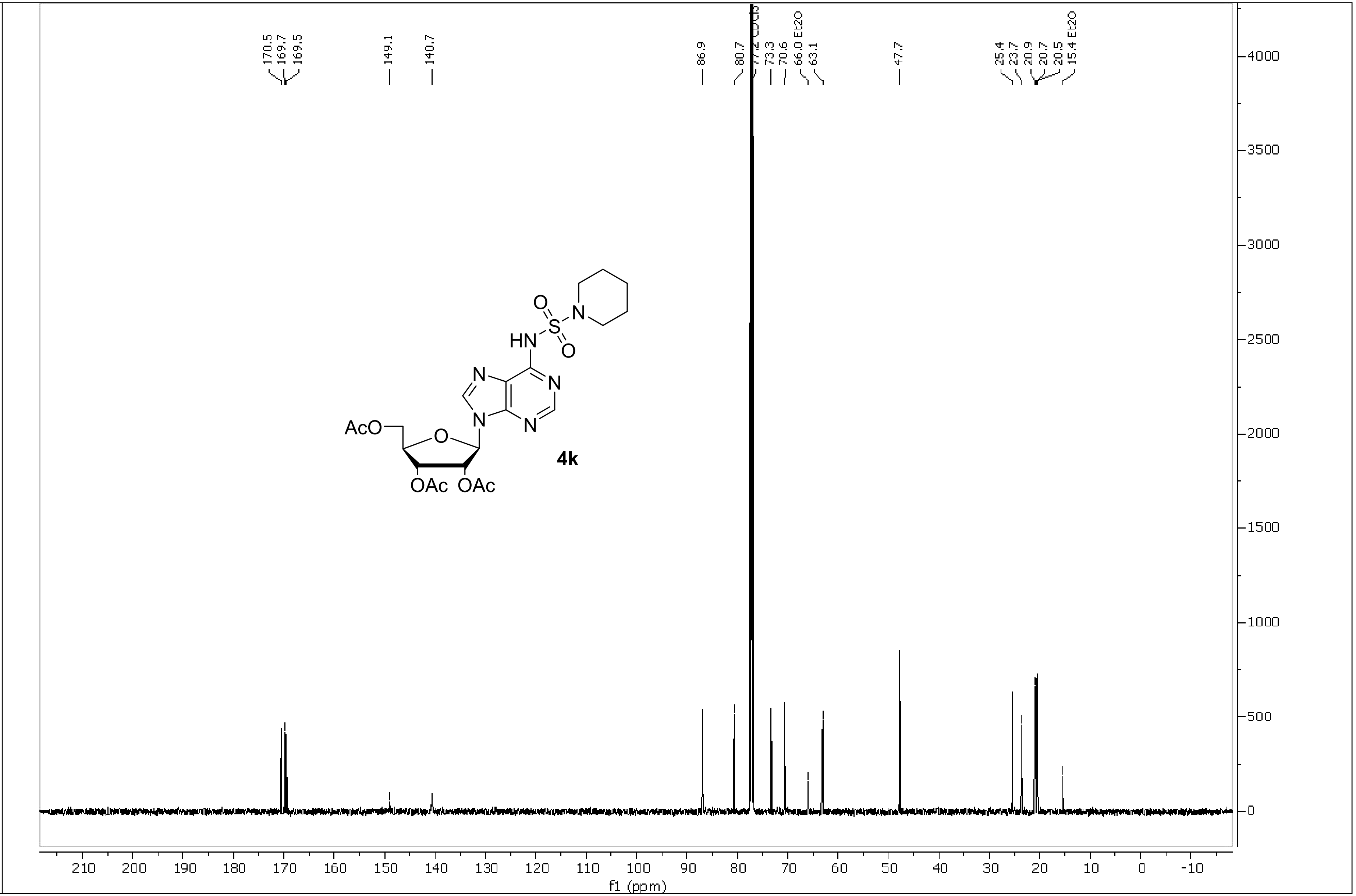

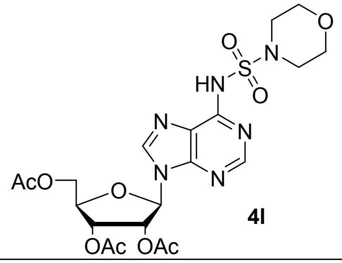

4l

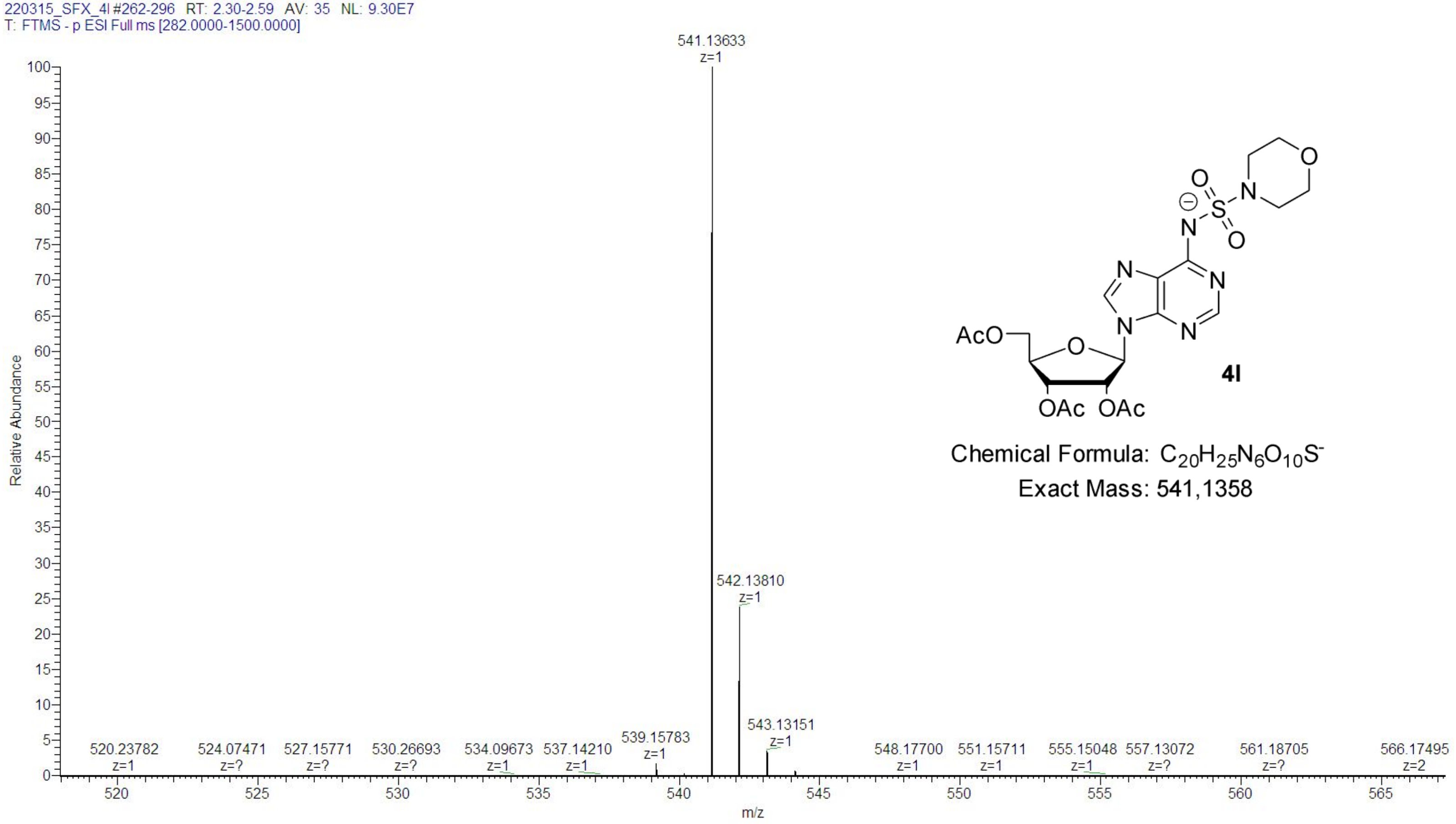

220315_SFX_4l #262-296 RT: 2.30-2.59 AV: 35 NL: 9.30E7
T: FTMS - p ESI Full ms [282.0000-1500.0000]
541.13633 z=1
542.13810 z=1
543.13151 z=1
539.15783 z=1
520.23782 z=1
524.07471 z=?
527.15771 z=?
530.26693 z=?
534.09673 z=1
537.14210 z=1
548.17700 z=1
551.15711 z=1
555.15048 z=1
557.13072 z=?
561.18705 z=?
566.17495 z=2
Relative Abundance
m/z
4l
Chemical Formula: C20H25N6O10S-
Exact Mass: 541,1358

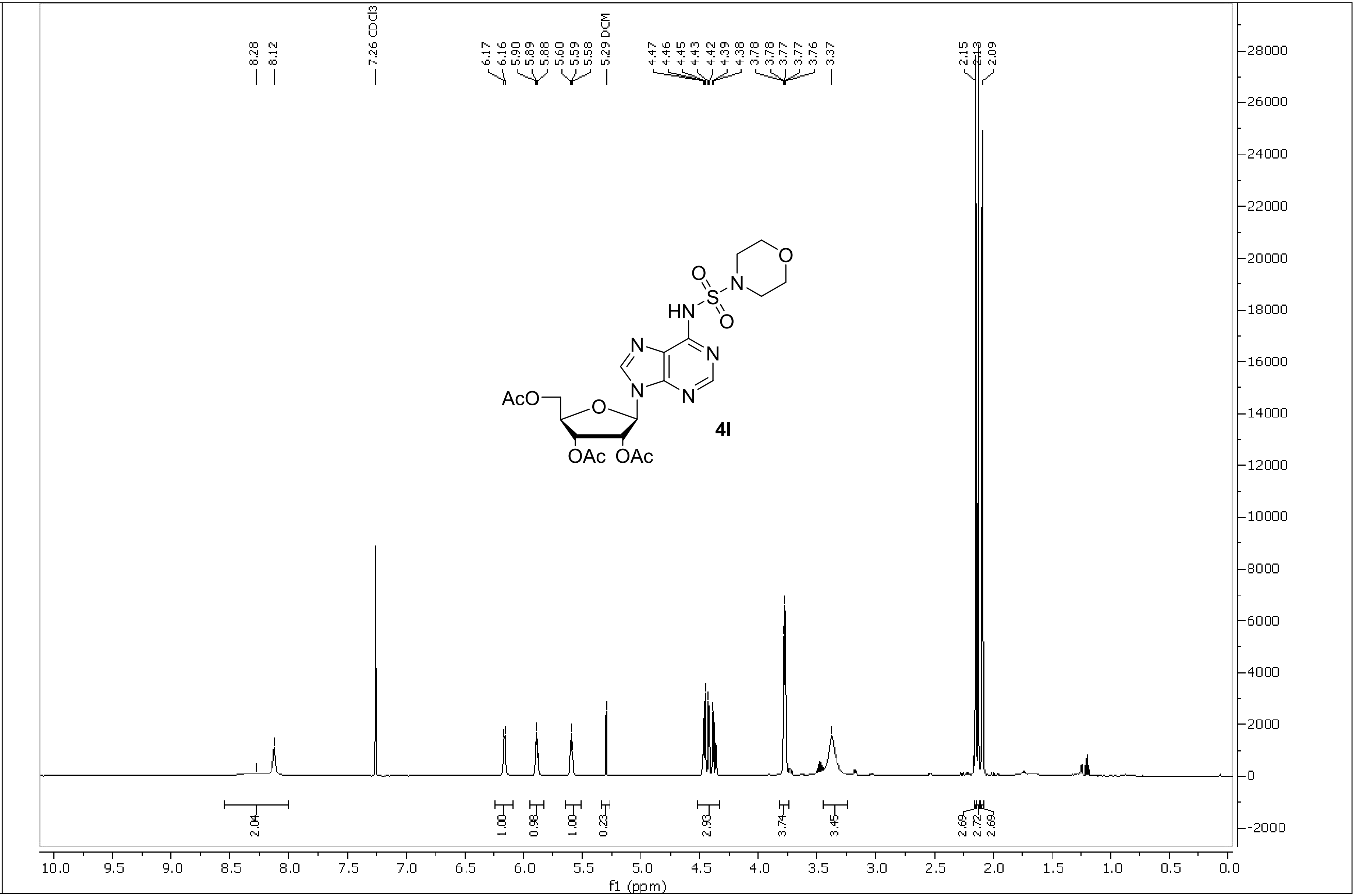

^{1}H NMR spectrum (500 MHz) of **4l**

^{13}C NMR spectrum (126 MHz) of **4l**

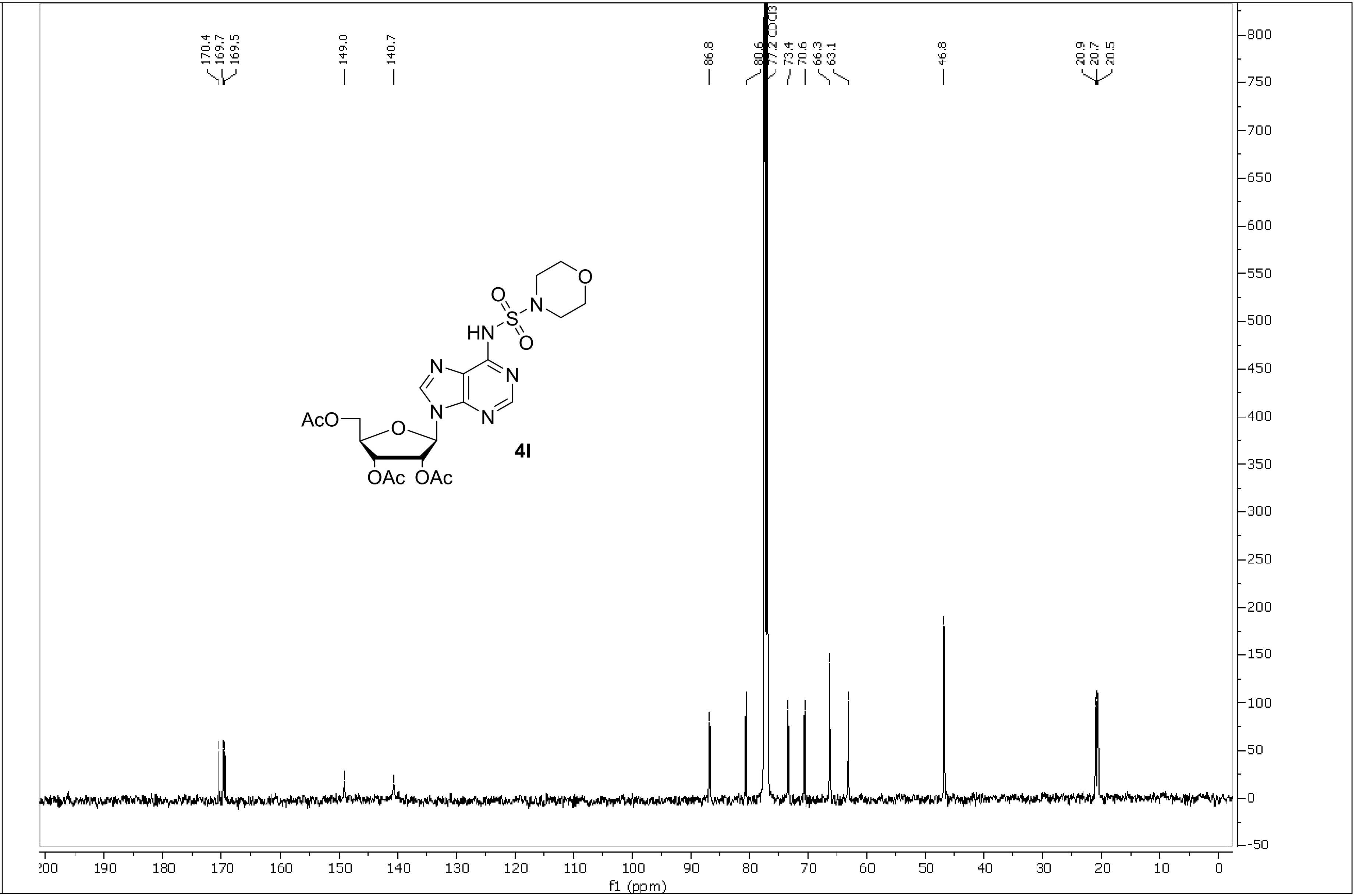

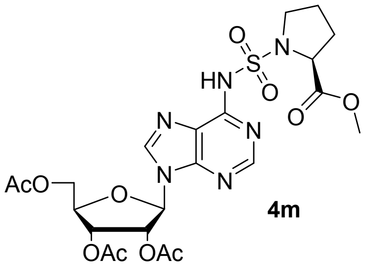

4m

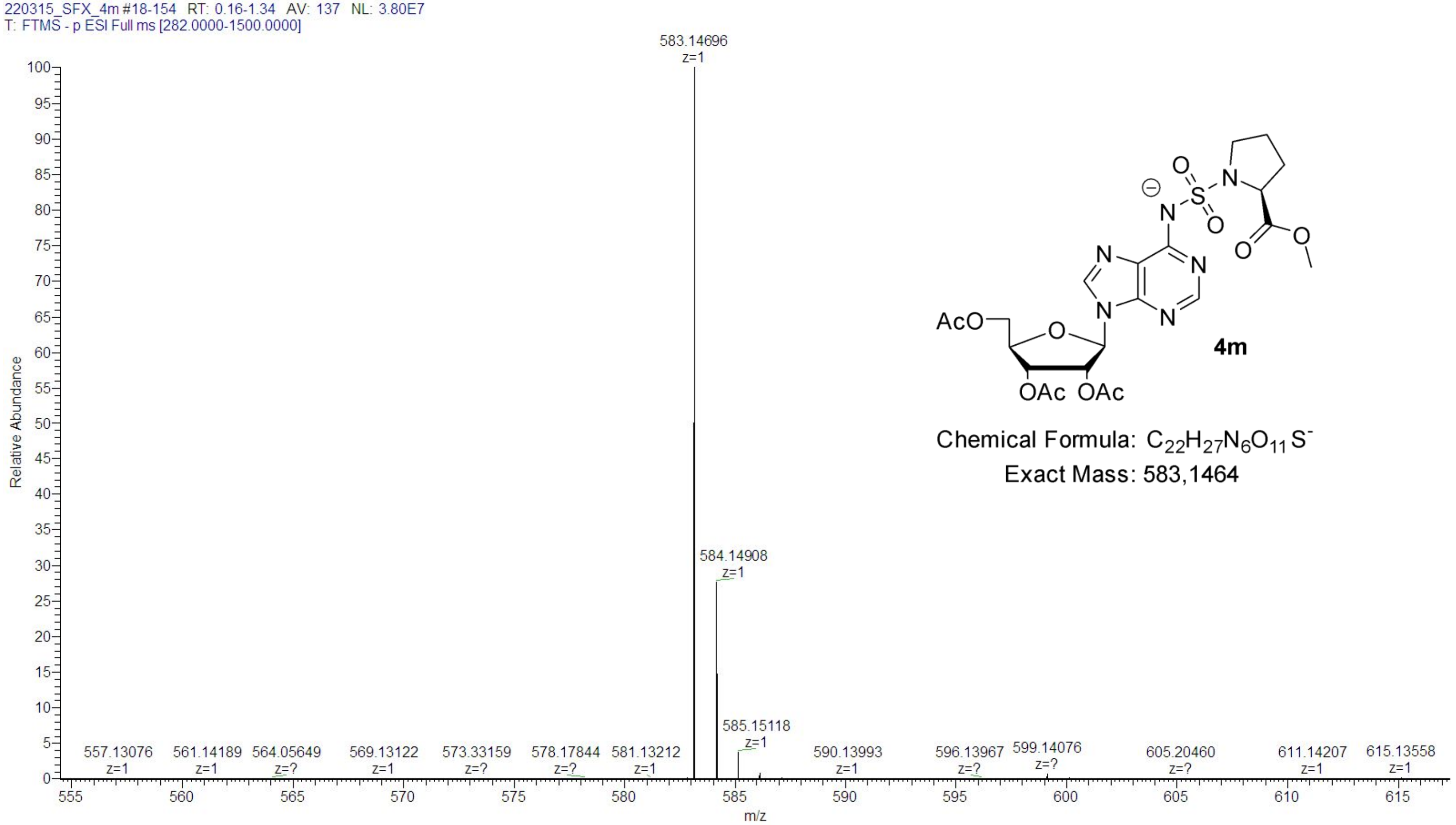

220315_SFX_4m #18-154 RT: 0.16-1.34 AV: 137 NL: 3.80E7
T: FTMS - p ESI Full ms [282.0000-1500.0000]
583.14696 z=1
584.14908 z=1
585.15118 z=1
557.13076 z=1
561.14189 z=1
564.05649 z=?
569.13122 z=1
573.33159 z=?
578.17844 z=?
581.13212 z=1
590.13993 z=1
596.13967 z=?
599.14076 z=?
605.20460 z=?
611.14207 z=1
615.13558 z=1
Relative Abundance
m/z
4m
Chemical Formula: $C_{22}H_{27}N_6O_{11}S^-$
Exact Mass: 583,1464

^{1}H NMR spectrum (500 MHz) of **4m**

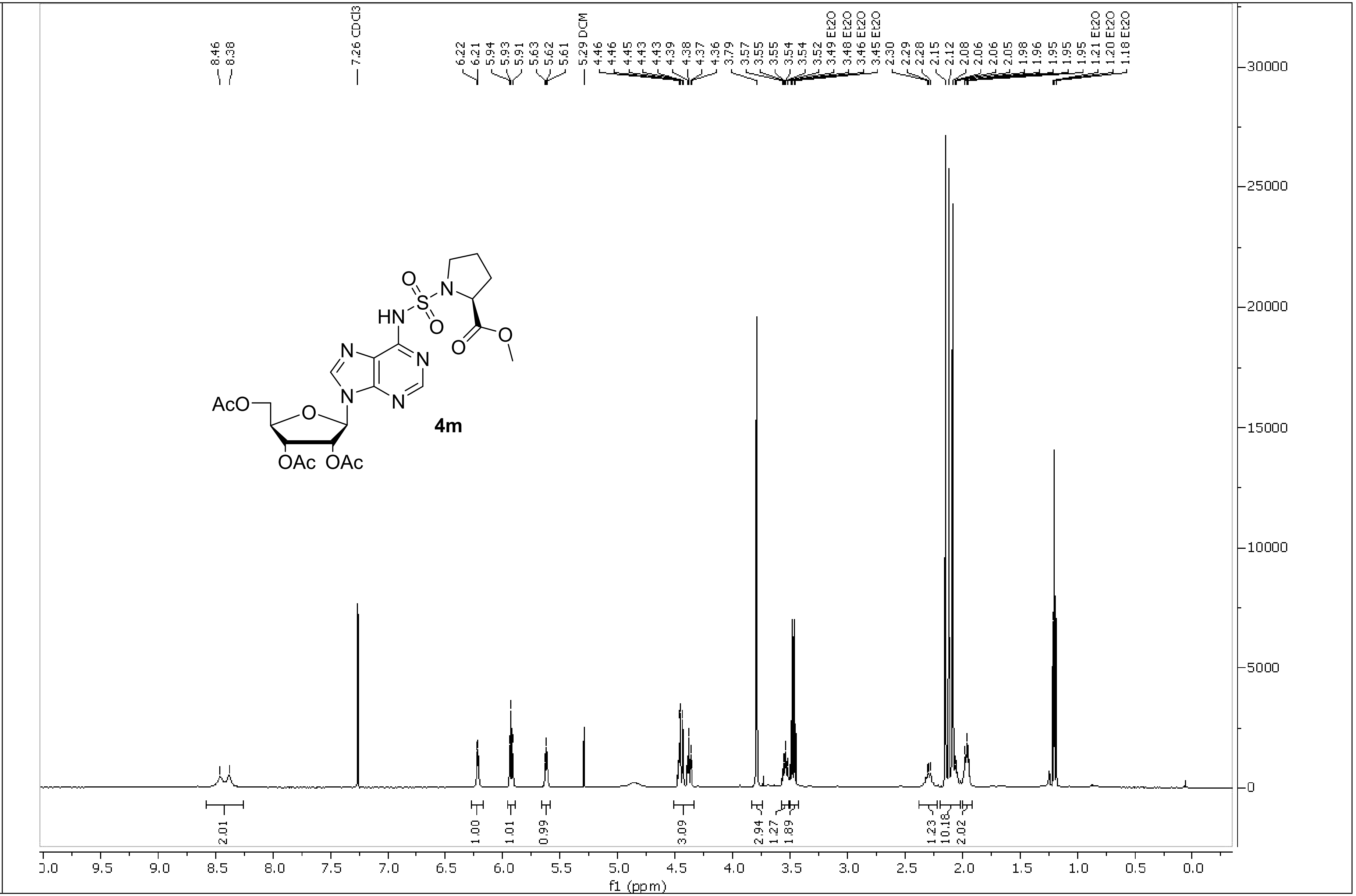

^{13}C NMR spectrum (126 MHz) of **4m**

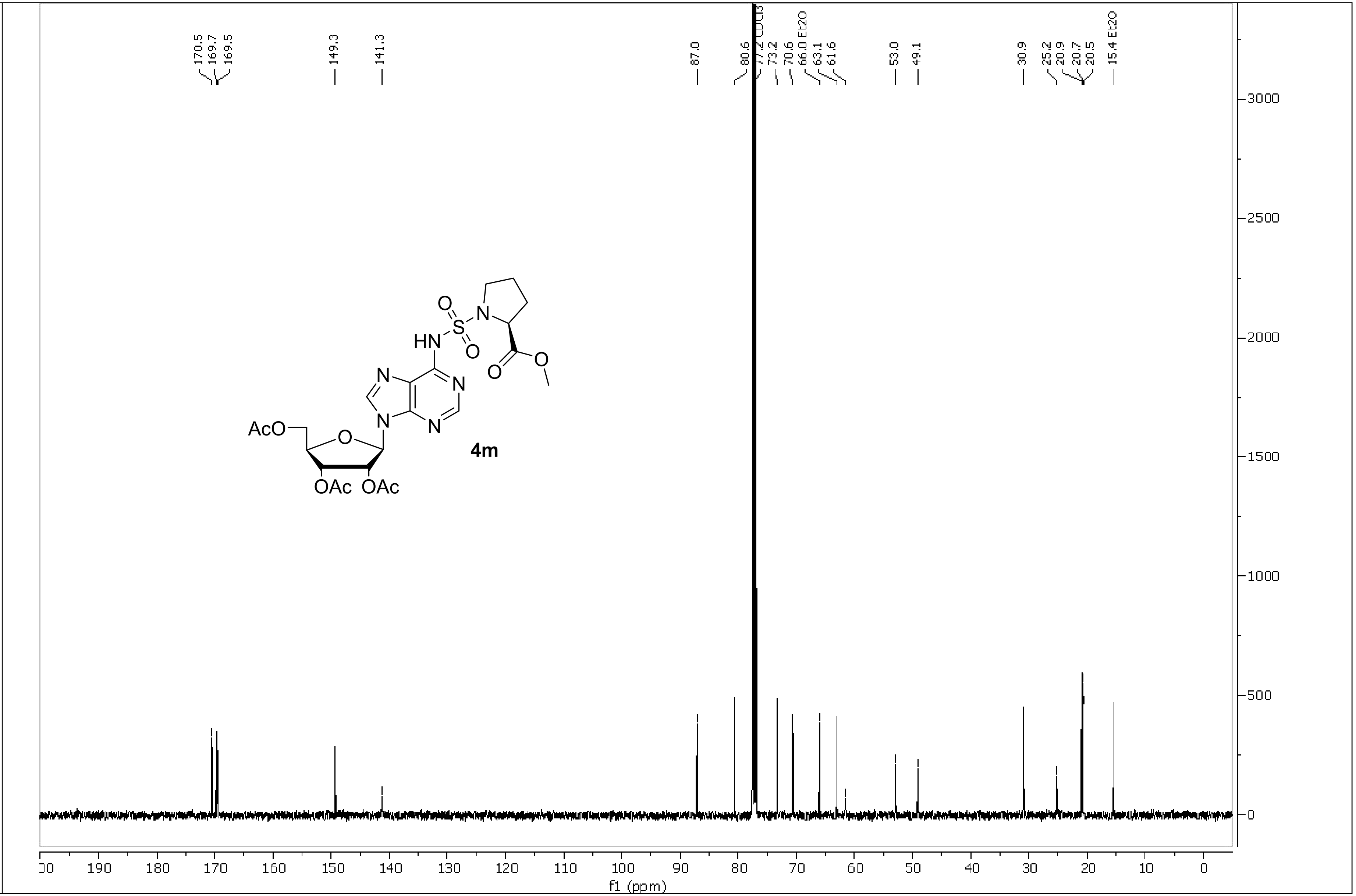

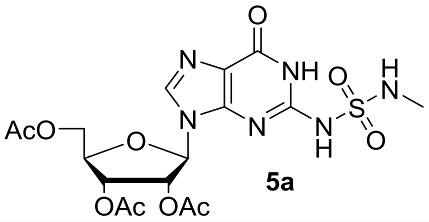

5a

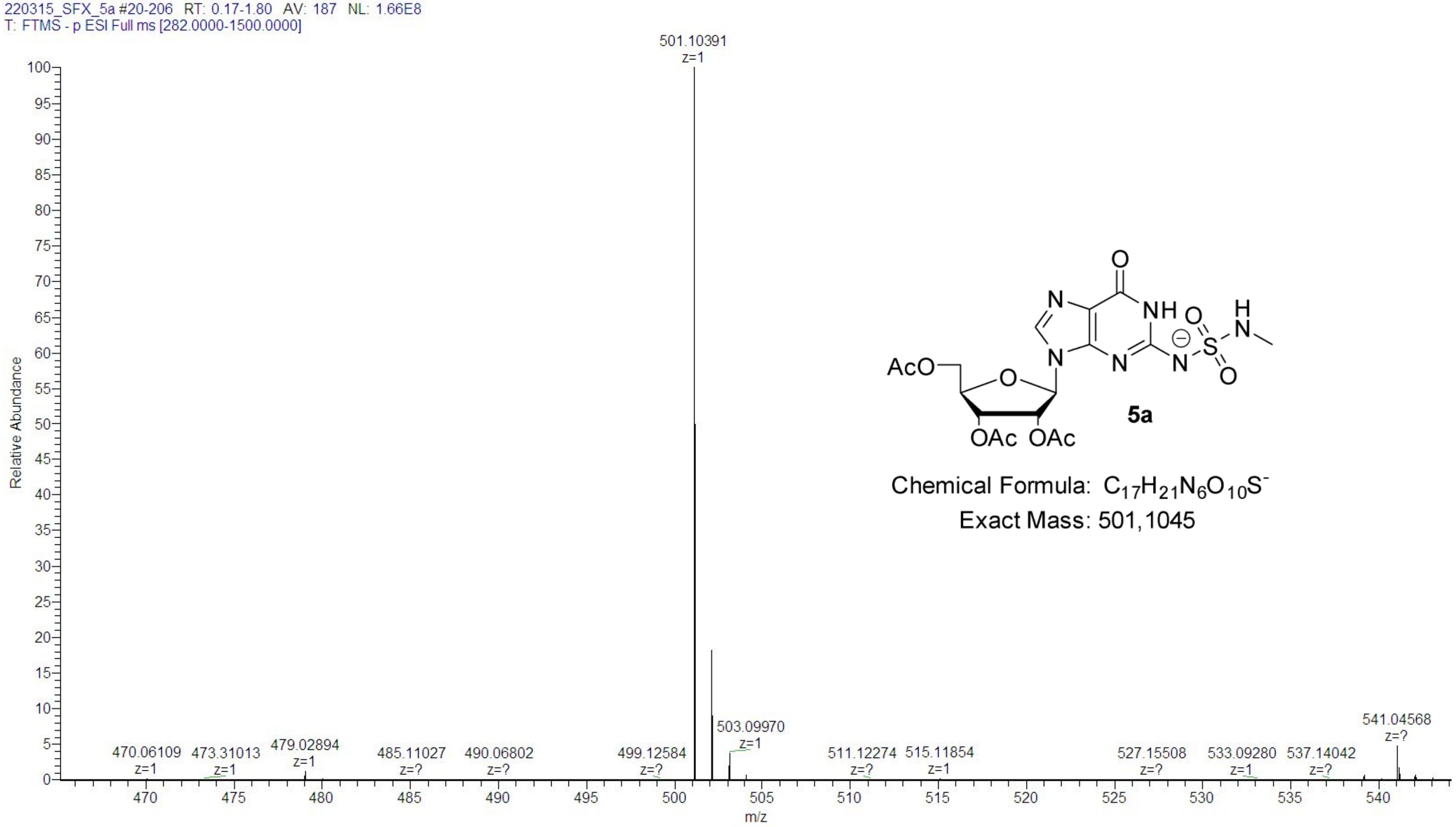

220315_SFX_5a #20-206 RT: 0.17-1.80 AV: 187 NL: 1.66E8
T: FTMS - p ESI Full ms [282.0000-1500.0000]
501.10391
z=1
Relative Abundance
m/z
470.06109 z=1
473.31013 z=1
479.02894 z=1
485.11027 z=?
490.06802 z=?
499.12584 z=?
503.09970 z=1
511.12274 z=?
515.11854 z=1
527.15508 z=?
533.09280 z=1
537.14042 z=?
541.04568 z=?
5a
Chemical Formula: $C_{17}H_{21}N_6O_{10}S^-$
Exact Mass: 501,1045

^{1}H NMR spectrum (500 MHz) of **5a**

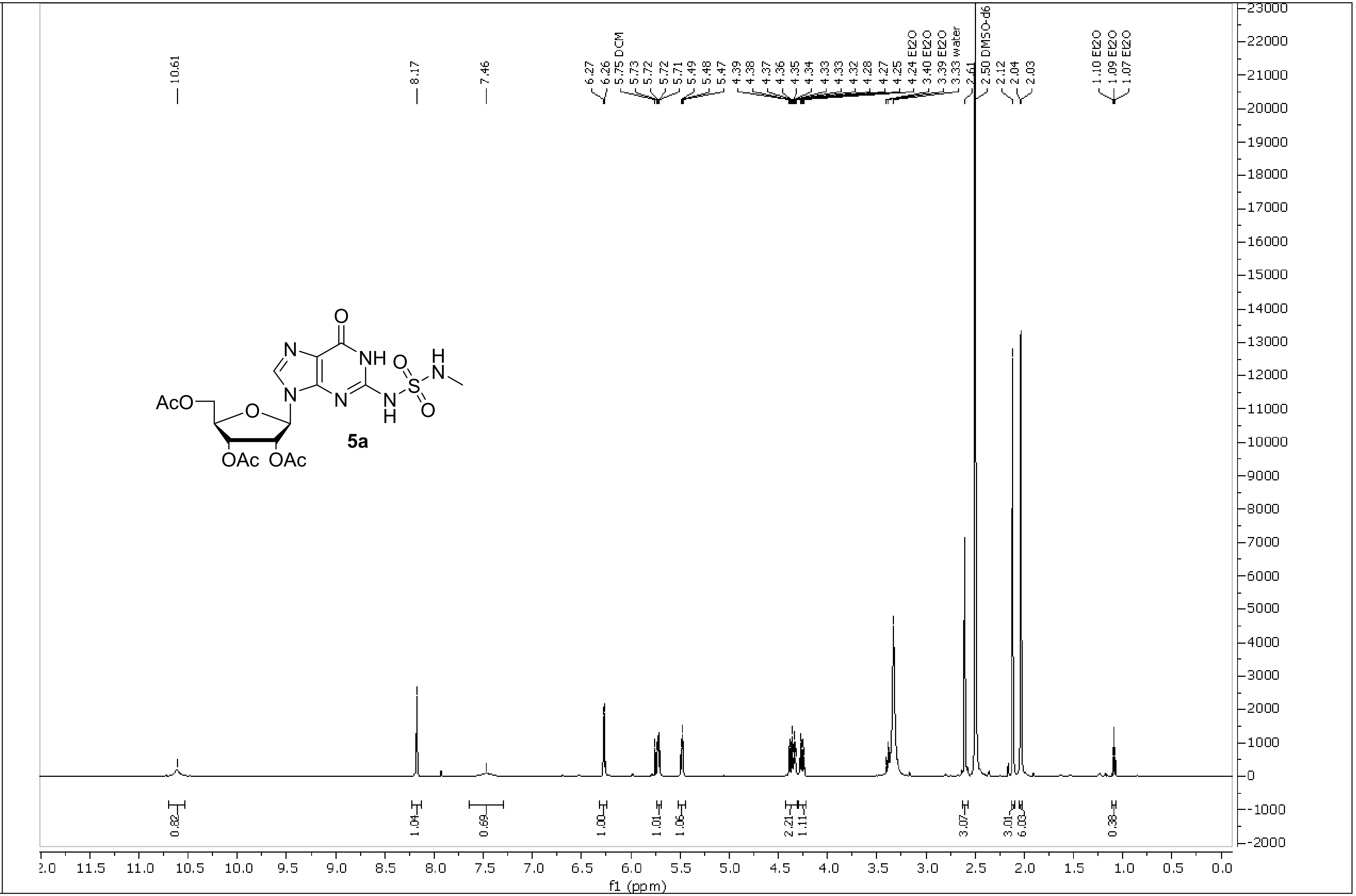

^{13}C NMR spectrum (126 MHz) of **5a**

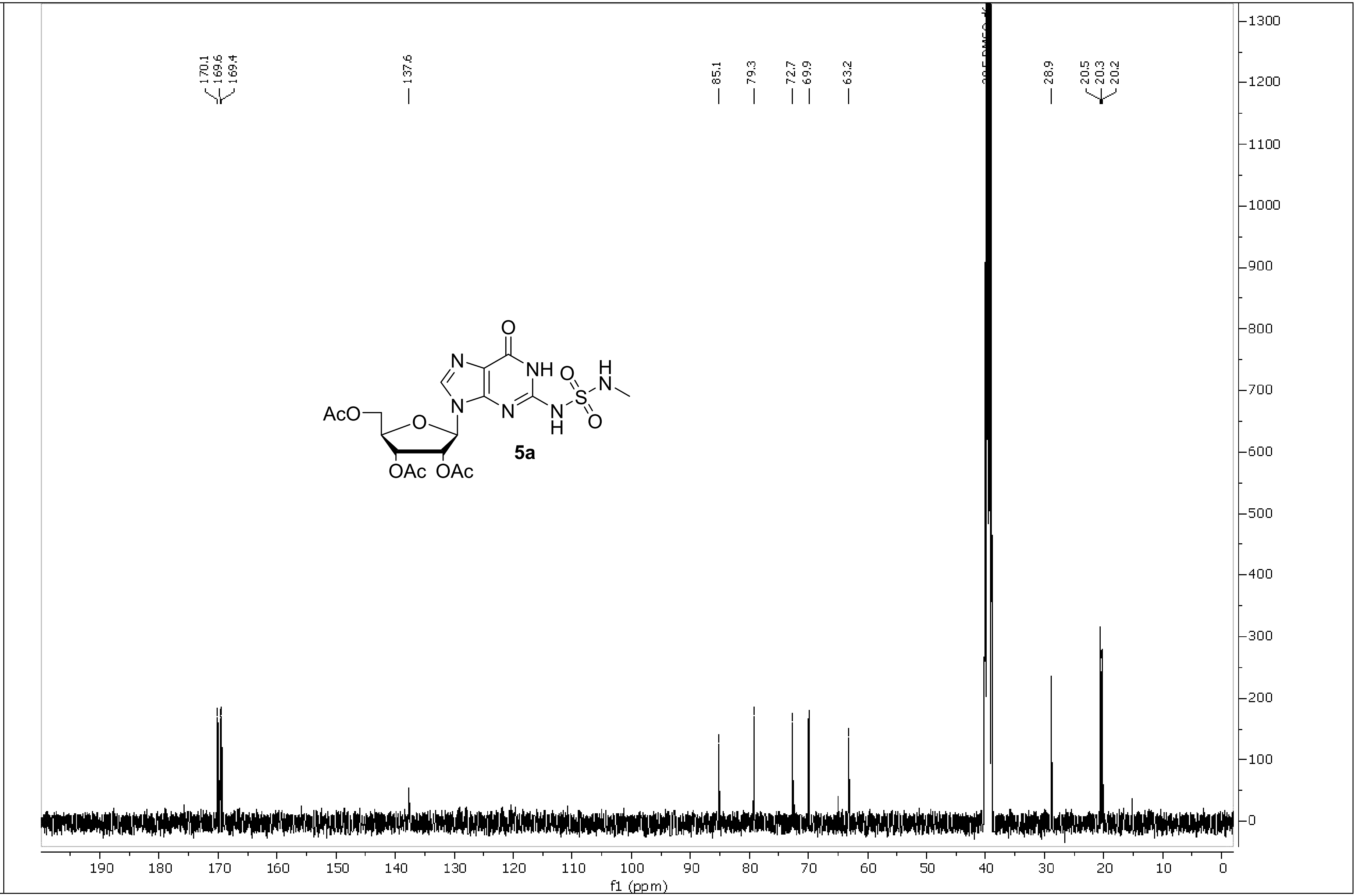

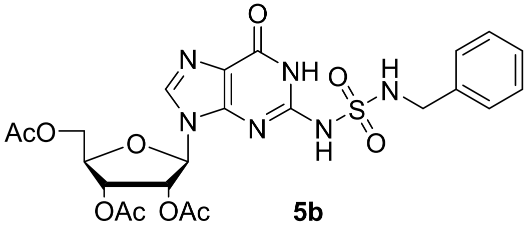

5b

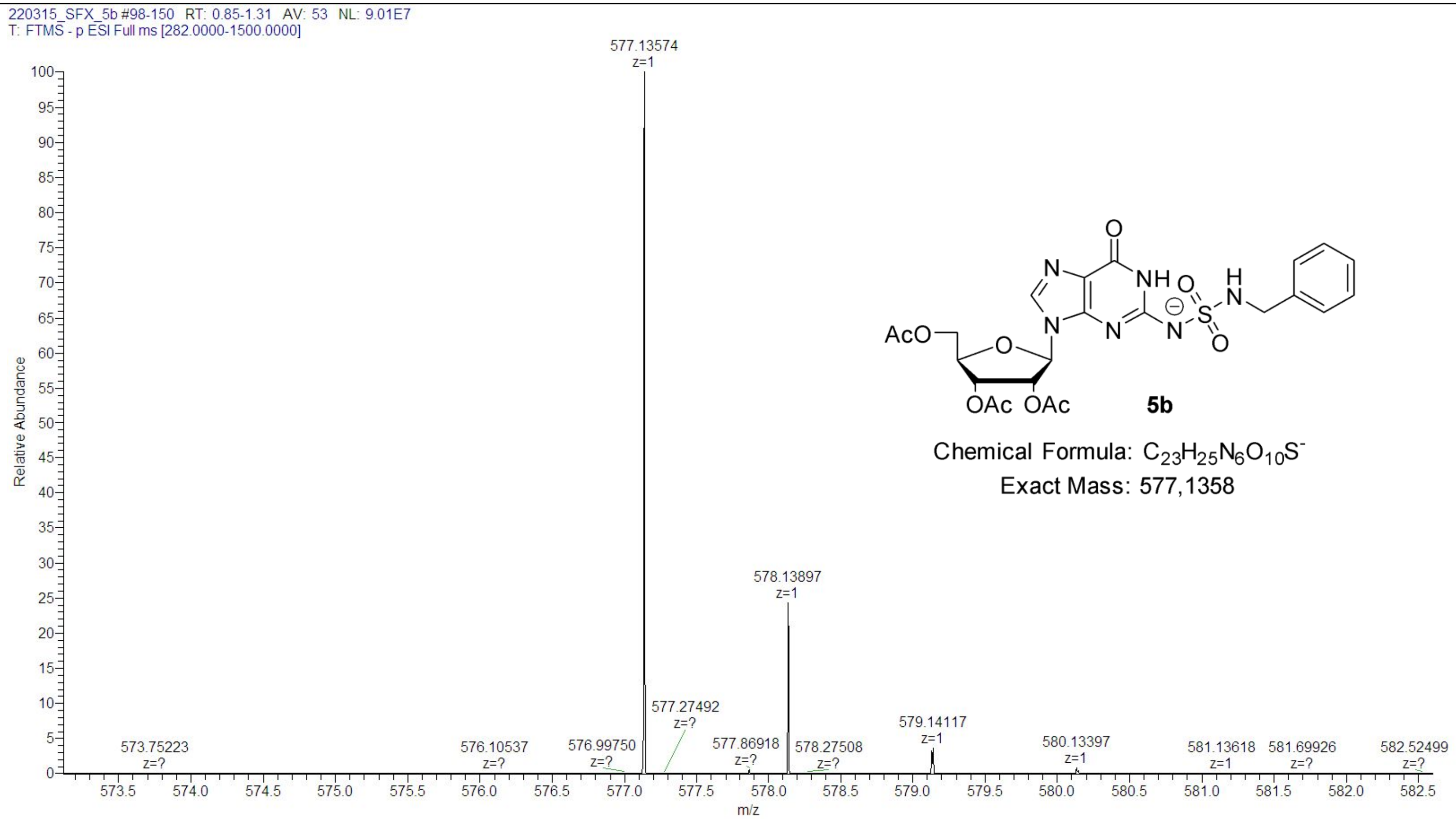
220315_SFX_5b #98-150 RT: 0.85-1.31 AV: 53 NL: 9.01E7
T: FTMS - p ESI Full ms [282.0000-1500.0000]
577.13574
z=1
578.13897
z=1
579.14117
z=1
580.13397
z=1
573.75223
z=?
576.10537
z=?
576.99750
z=?
577.27492
z=?
577.86918
z=?
578.27508
z=?
581.13618
z=1
581.69926
z=?
582.52499
z=?
Relative Abundance
m/z
5b
Chemical Formula: $C_{23}H_{25}N_6O_{10}S^-$
Exact Mass: 577,1358

^{1}H NMR spectrum (500 MHz) of **5b**

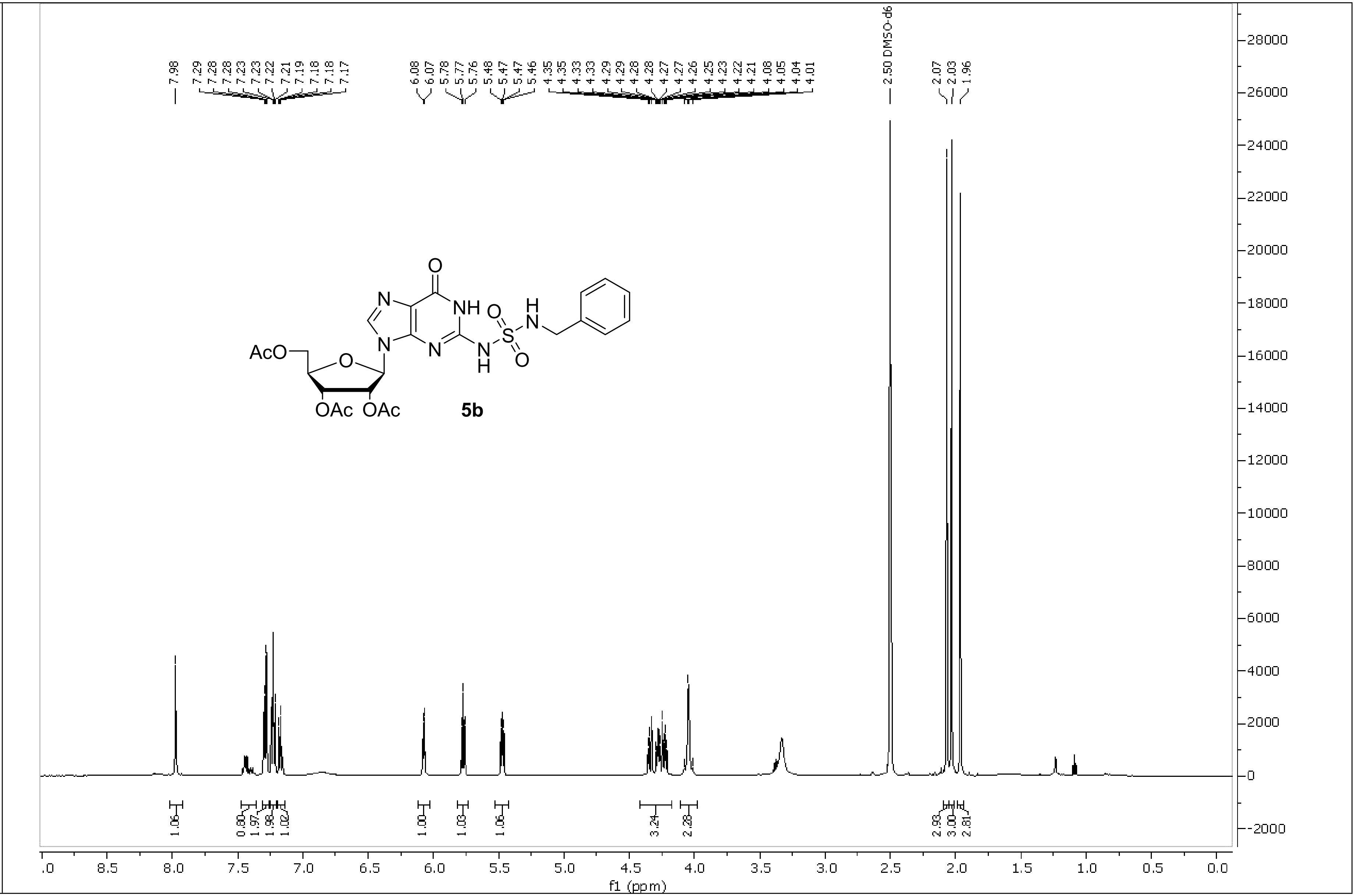

^{13}C NMR spectrum (126 MHz) of **5b**

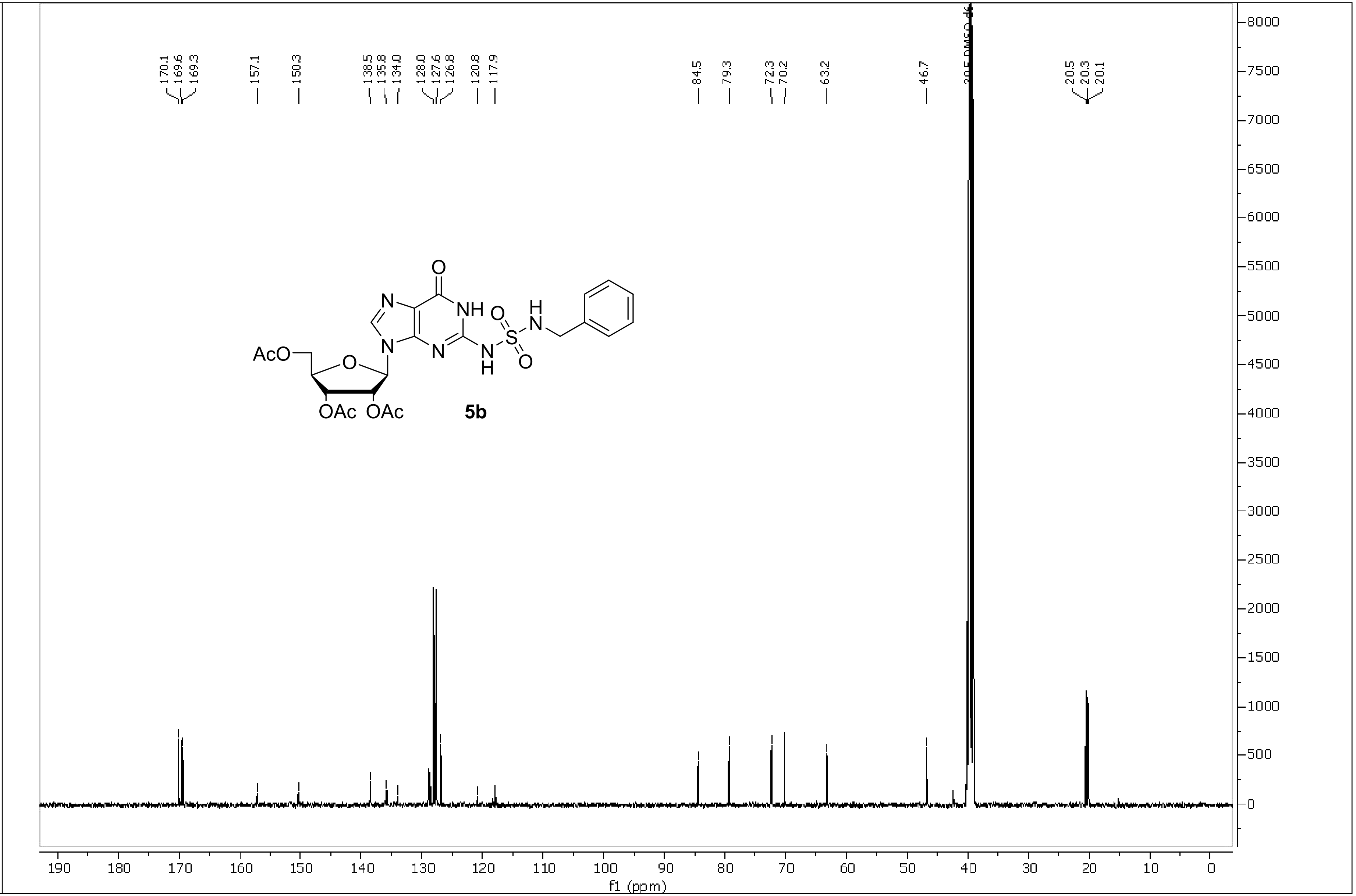

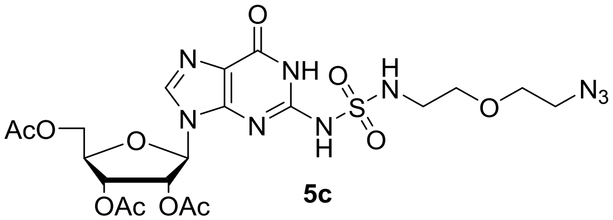

5c

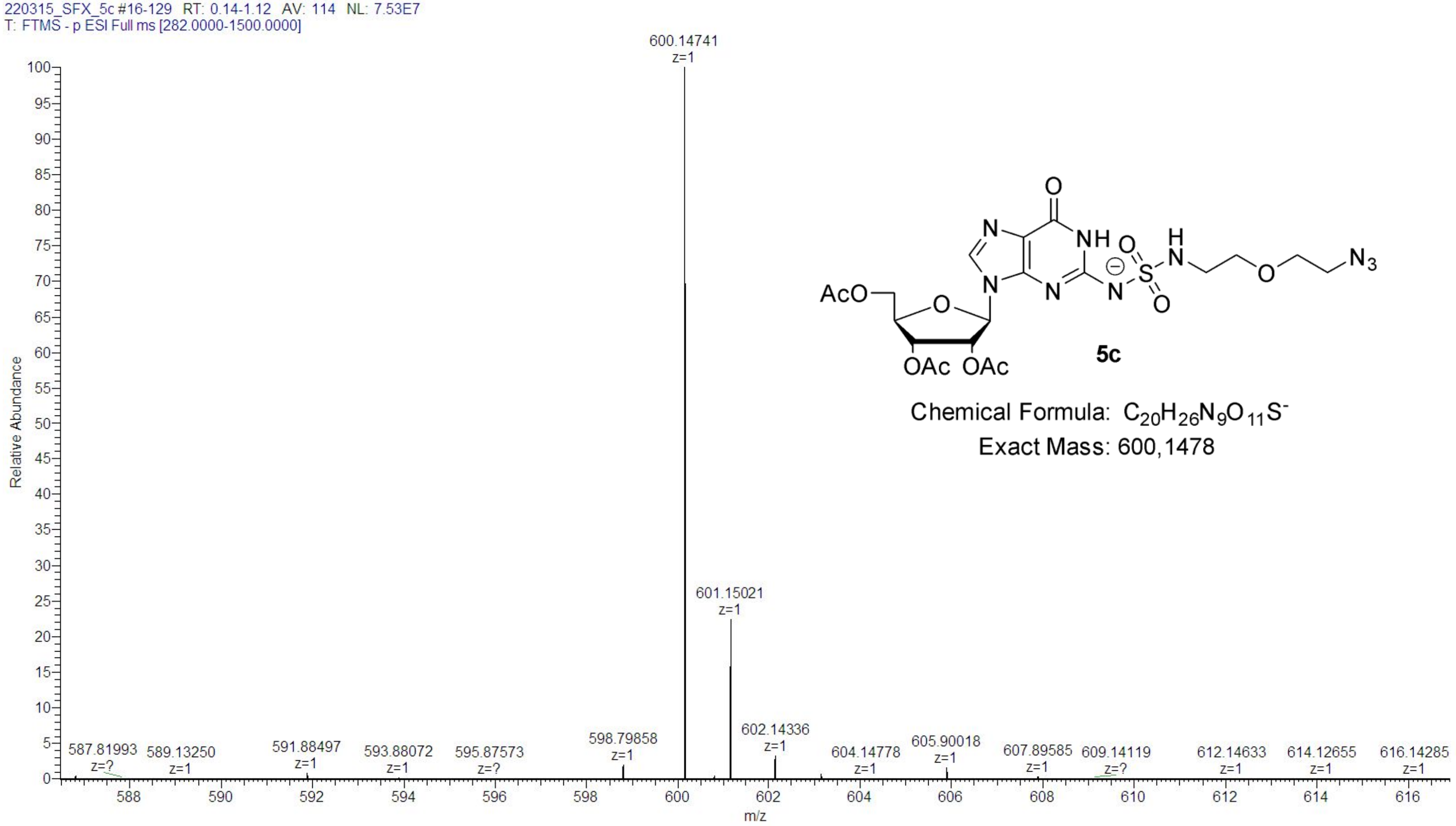
220315_SFX_5c #16-129 RT: 0.14-1.12 AV: 114 NL: 7.53E7
T: FTMS - p ESI Full ms [282.0000-1500.0000]
600.14741 z=1
601.15021 z=1
602.14336 z=1
598.79858 z=1
587.81993 z=?
589.13250 z=1
591.88497 z=1
593.88072 z=1
595.87573 z=?
604.14778 z=1
605.90018 z=1
607.89585 z=1
609.14119 z=?
612.14633 z=1
614.12655 z=1
616.14285 z=1
Relative Abundance
m/z
5c
Chemical Formula: $C_{20}H_{26}N_9O_{11}S^-$
Exact Mass: 600,1478

^1H NMR spectrum (500 MHz) of **5c**

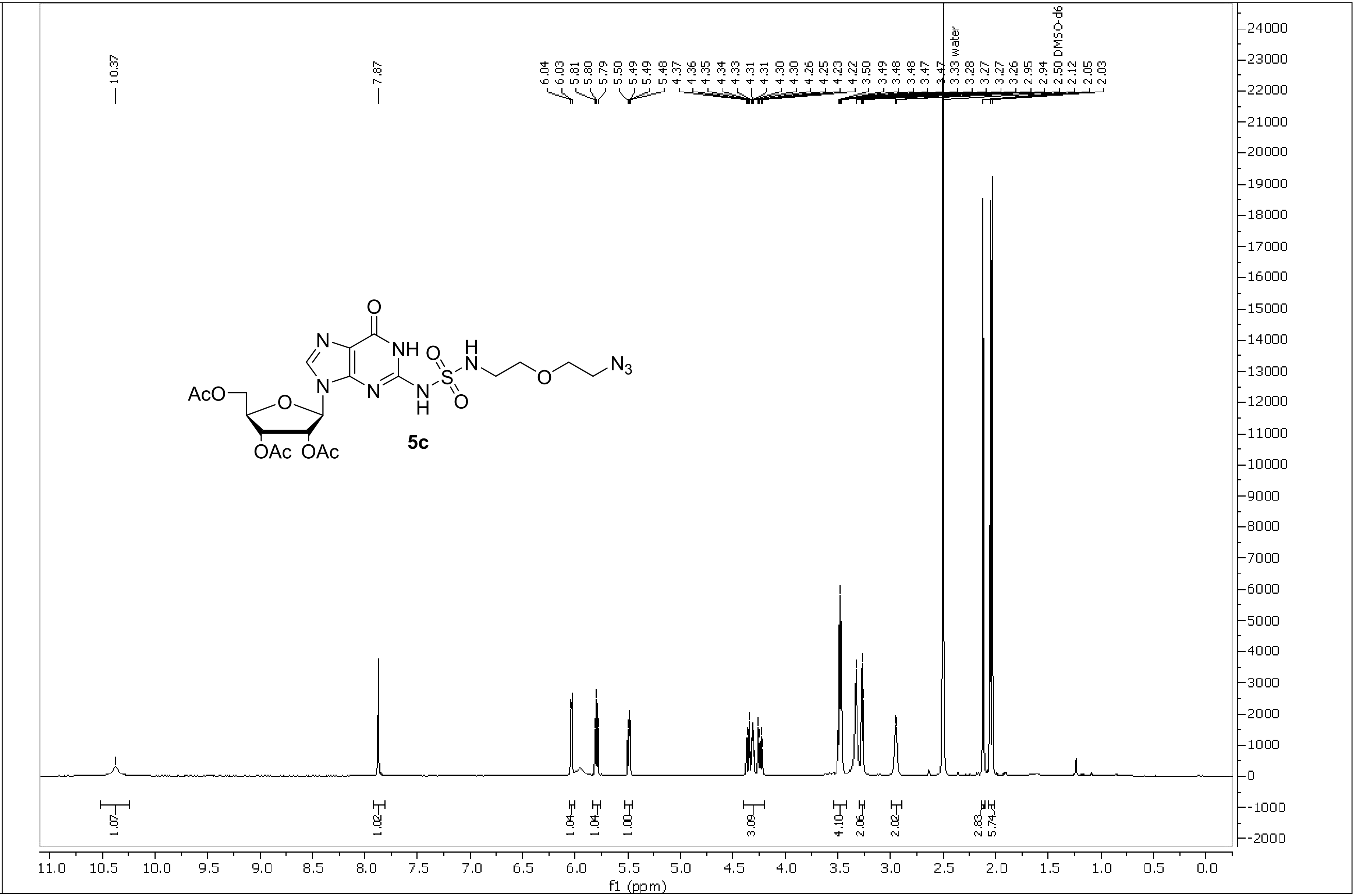

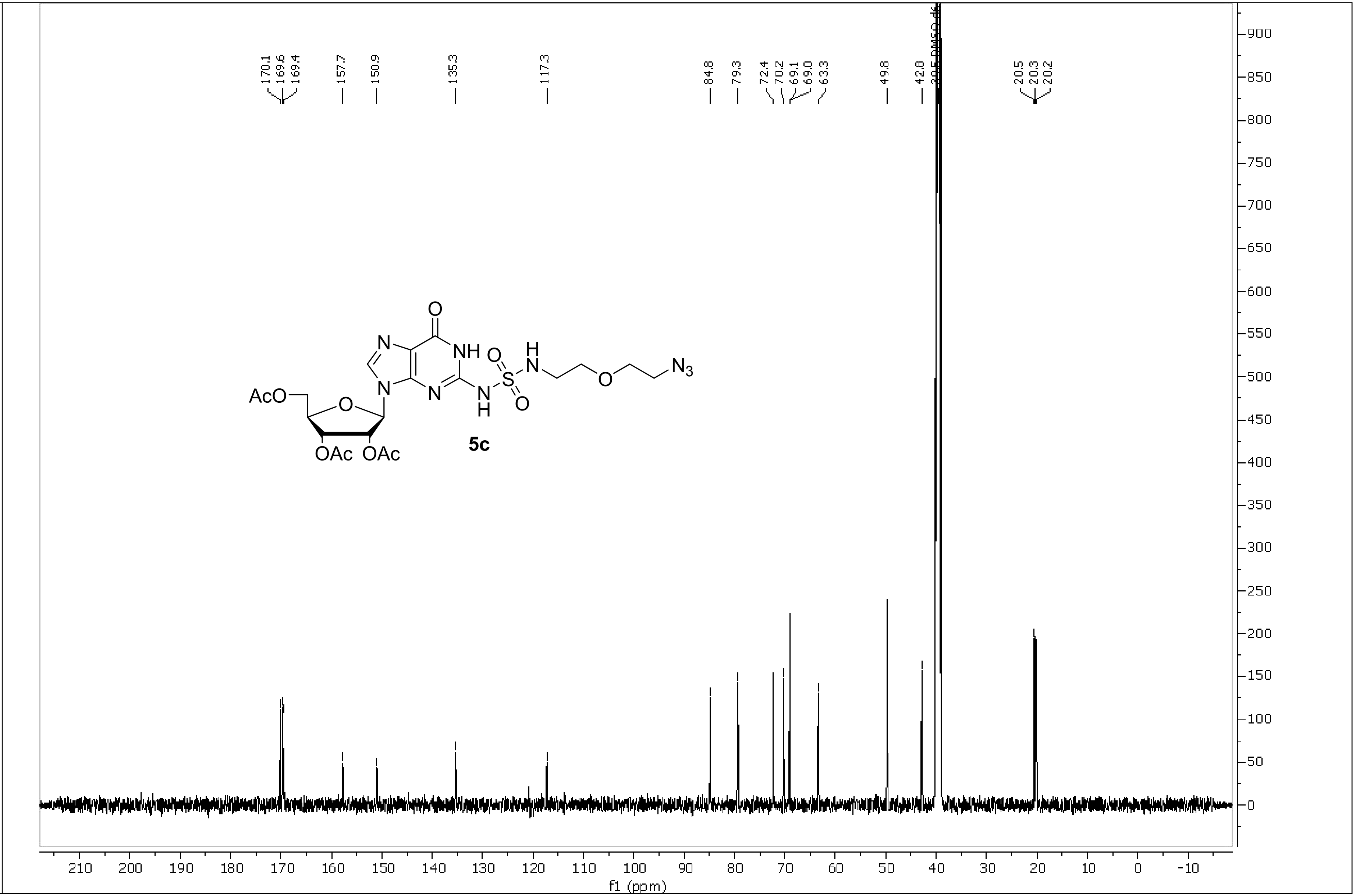

^{13}C NMR spectrum (126 MHz) of **5c**

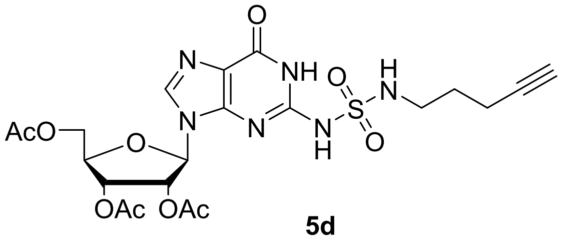
O
N
NH
O
H
N
AcO
O
N
N
N
H
S
O
OAc
OAc

5d

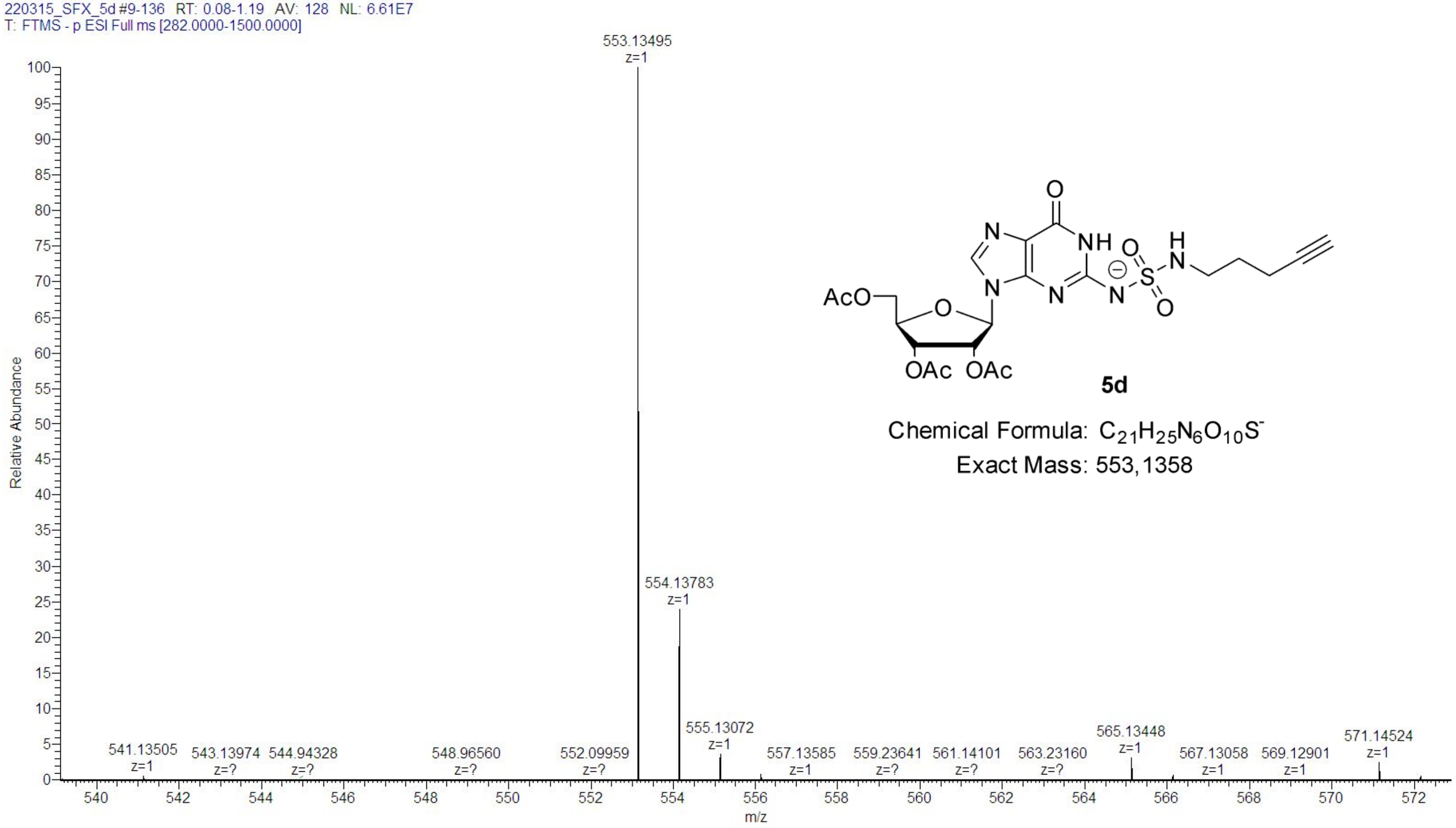
220315_SFX_5d #9-136 RT: 0.08-1.19 AV: 128 NL: 6.61E7
T: FTMS - p ESI Full ms [282.0000-1500.0000]
553.13495 z=1
554.13783 z=1
555.13072 z=1
541.13505 z=1
543.13974 z=?
544.94328 z=?
548.96560 z=?
552.09959 z=?
557.13585 z=1
559.23641 z=?
561.14101 z=?
563.23160 z=?
565.13448 z=1
567.13058 z=1
569.12901 z=1
571.14524 z=1
Relative Abundance
m/z
5d
Chemical Formula: C21H25N6O10S-
Exact Mass: 553,1358

^{1}H NMR spectrum (500 MHz) of **5d**

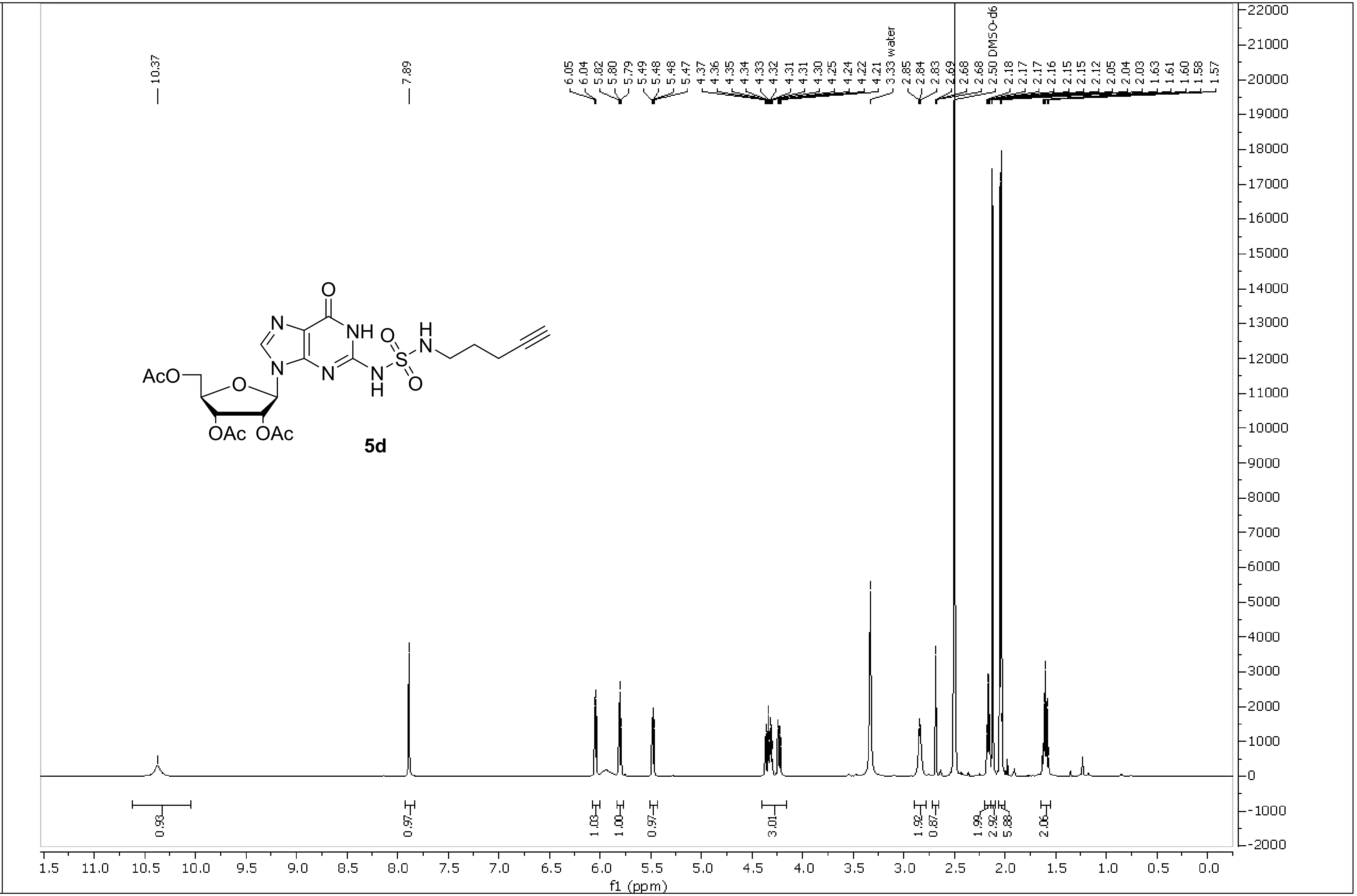

^{13}C NMR spectrum (126 MHz) of **5d**

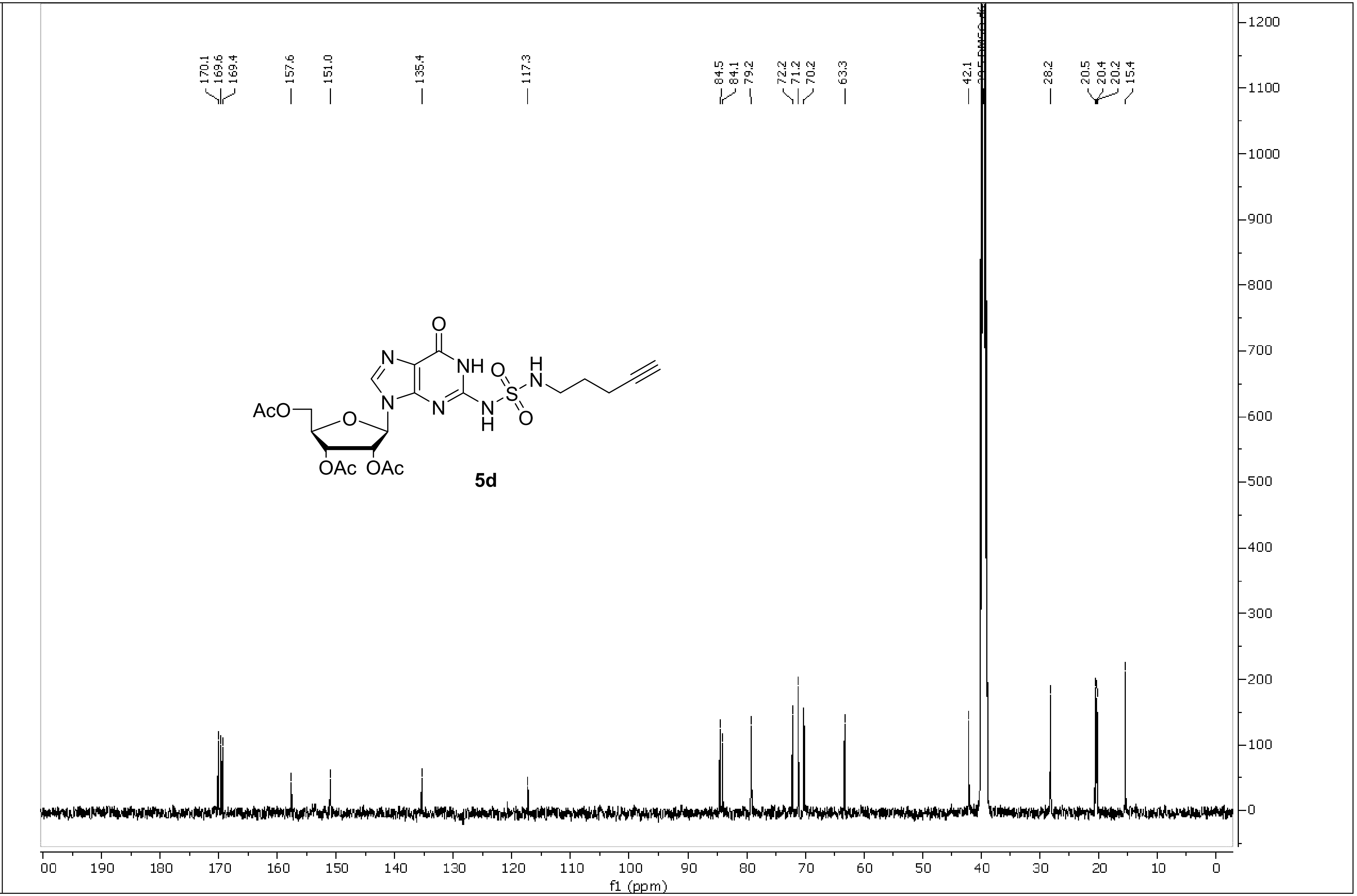

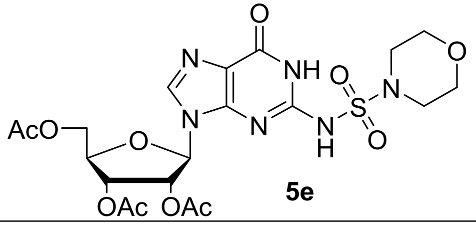

5e

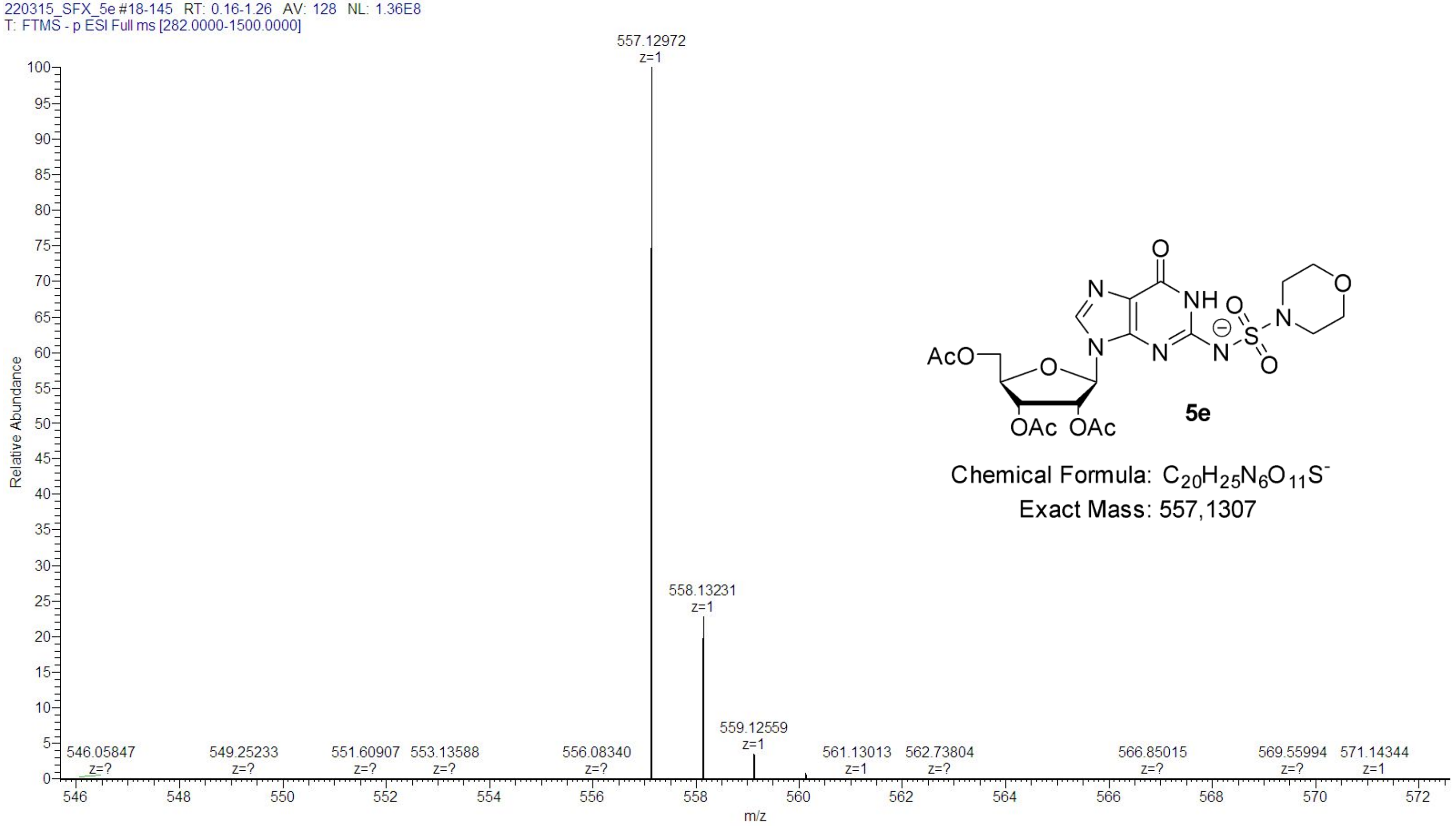
220315_SFX_5e #18-145 RT: 0.16-1.26 AV: 128 NL: 1.36E8
T: FTMS - p ESI Full ms [282.0000-1500.0000]
557.12972 z=1
558.13231 z=1
559.12559 z=1
546.05847 z=?
549.25233 z=?
551.60907 z=?
553.13588 z=?
556.08340 z=?
561.13013 z=1
562.73804 z=?
566.85015 z=?
569.55994 z=?
571.14344 z=1
Relative Abundance
m/z
5e
Chemical Formula: $C_{20}H_{25}N_6O_{11}S^-$
Exact Mass: 557,1307

^{1}H NMR spectrum (500 MHz) of **5e**

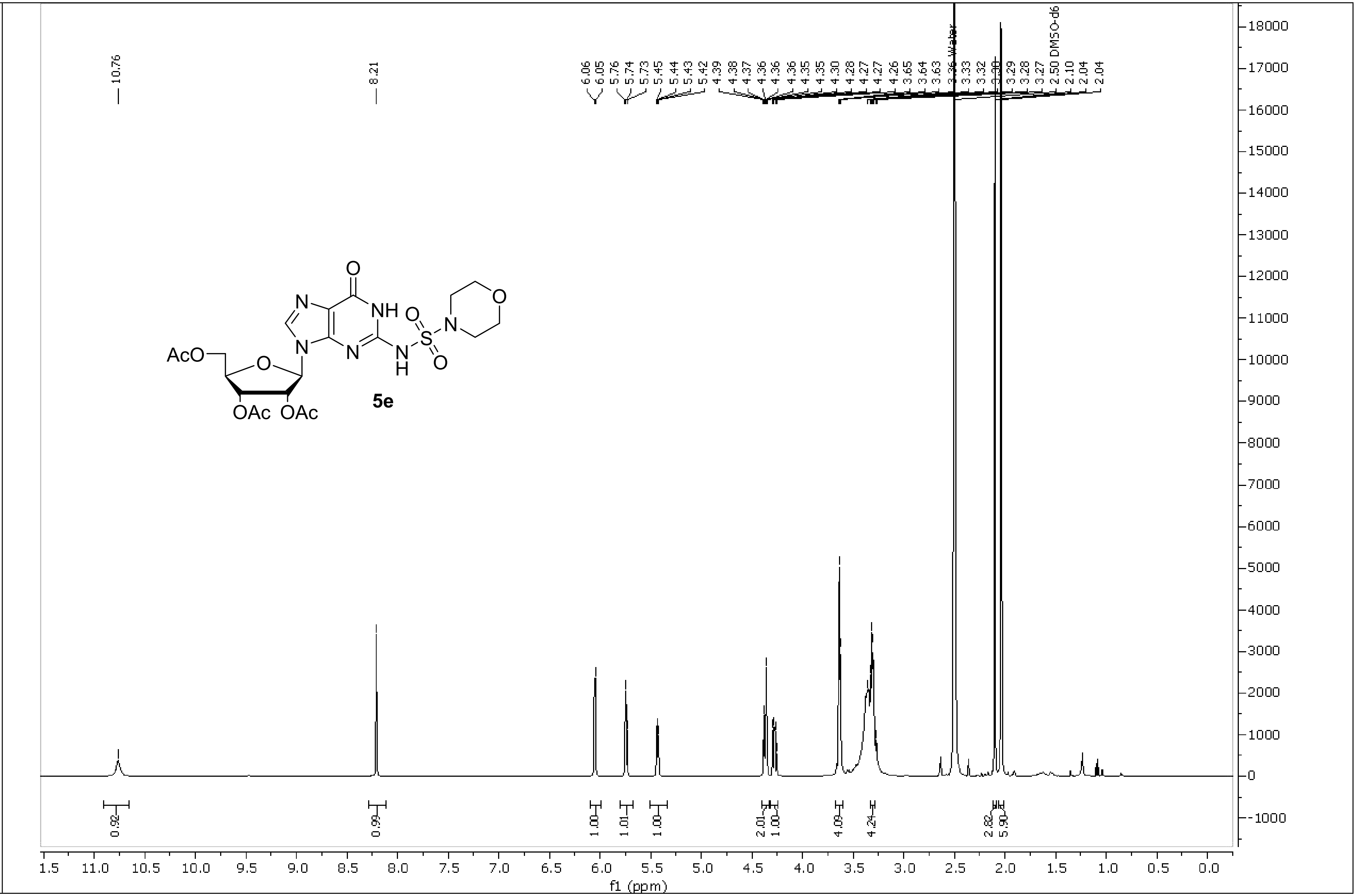

^{13}C NMR spectrum (126 MHz) of **5e**

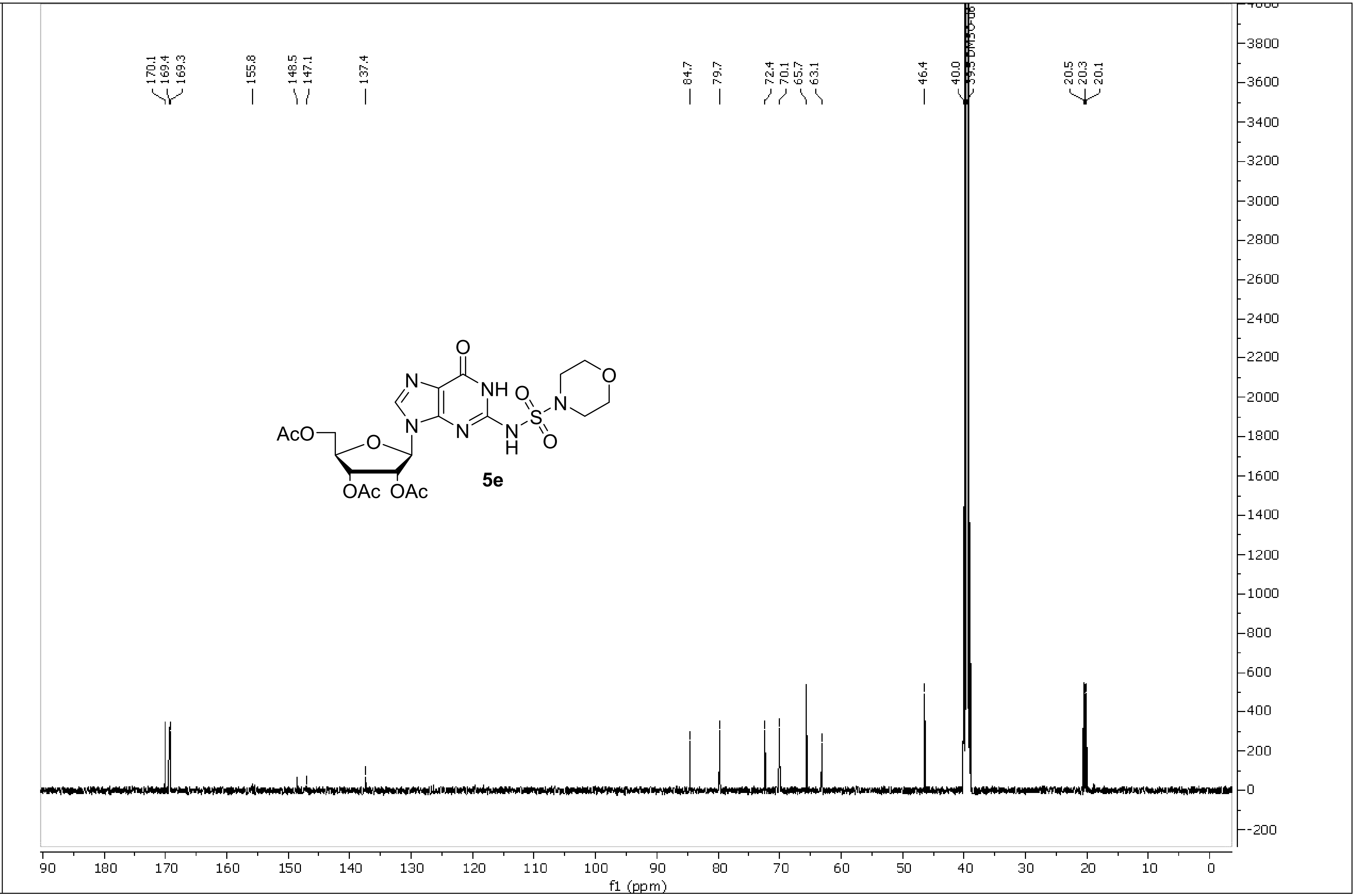

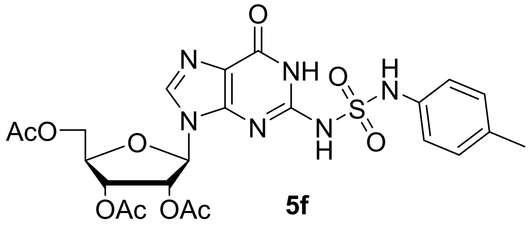
AcO
OAc OAc
5f

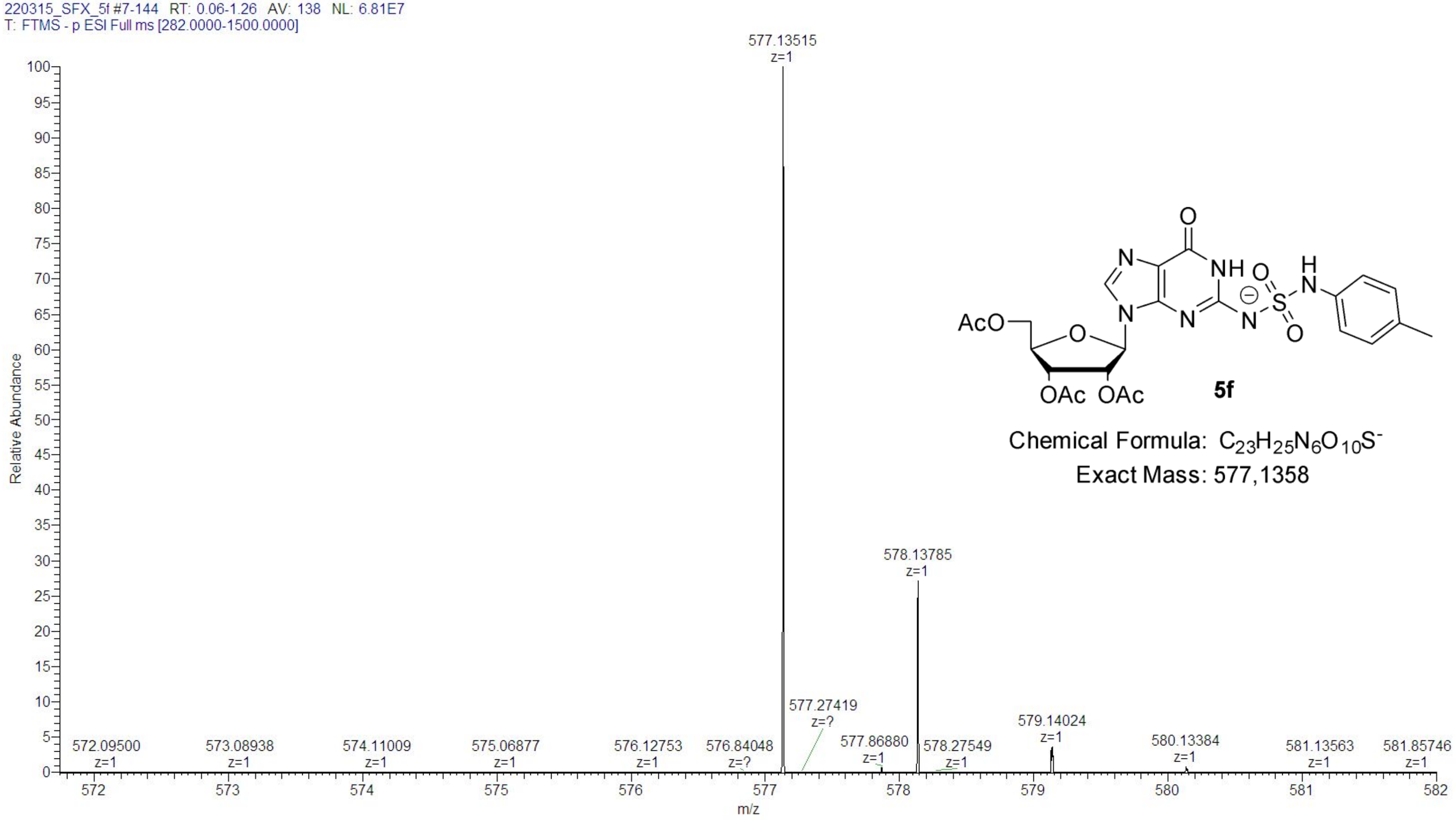
220315_SFX_5f #7-144 RT: 0.06-1.26 AV: 138 NL: 6.81E7
T: FTMS - p ESI Full ms [282.0000-1500.0000]
577.13515
z=1
578.13785
z=1
577.27419
z=?
579.14024
z=1
572.09500
z=1
573.08938
z=1
574.11009
z=1
575.06877
z=1
576.12753
z=1
576.84048
z=?
577.86880
z=1
578.27549
z=1
580.13384
z=1
581.13563
z=1
581.85746
z=1
Relative Abundance
m/z
5f
Chemical Formula: C23H25N6O10S-
Exact Mass: 577,1358

^{1}H NMR spectrum (500 MHz) of **5f**

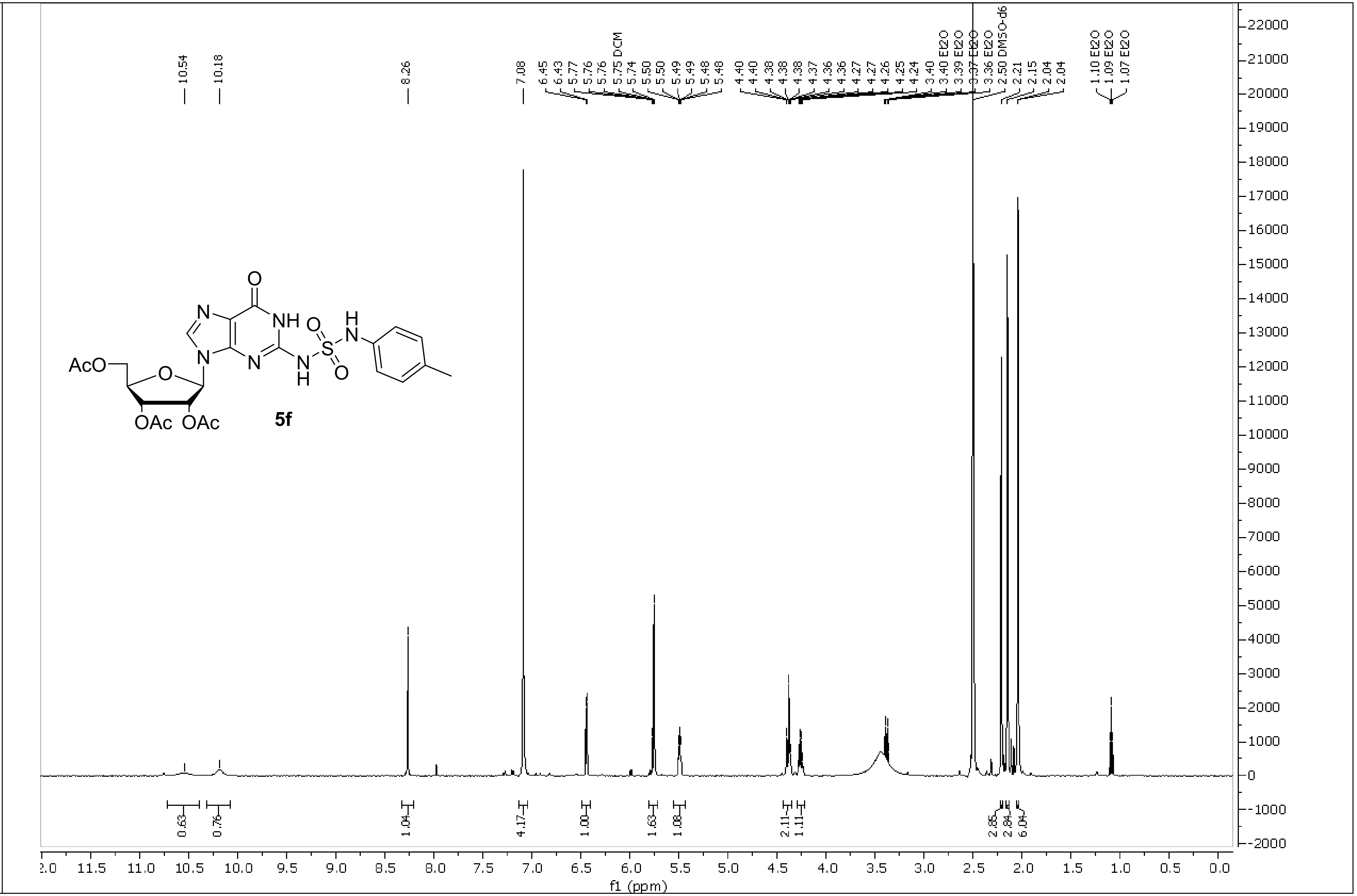

^{13}C NMR spectrum (126 MHz) of **5f**

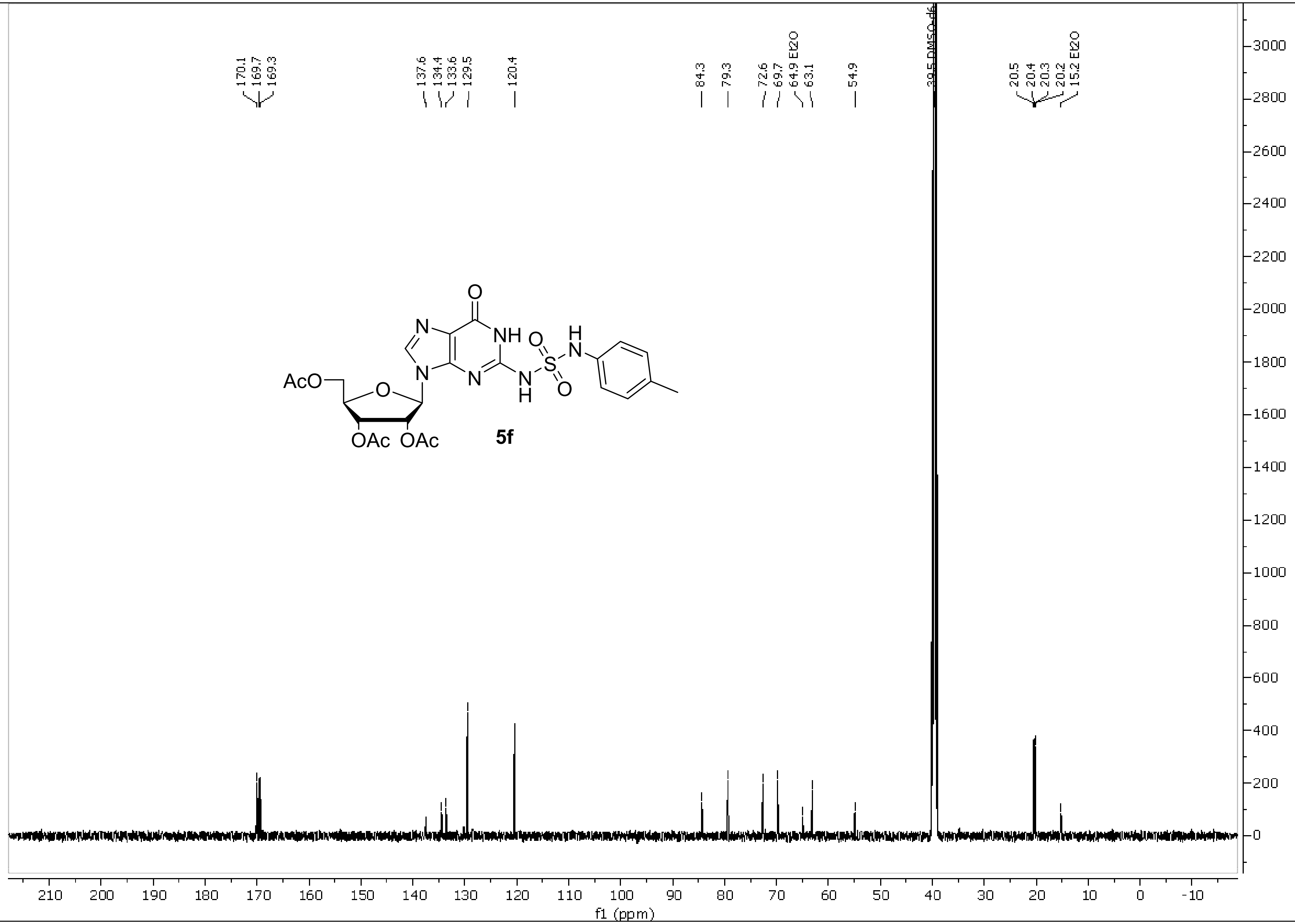

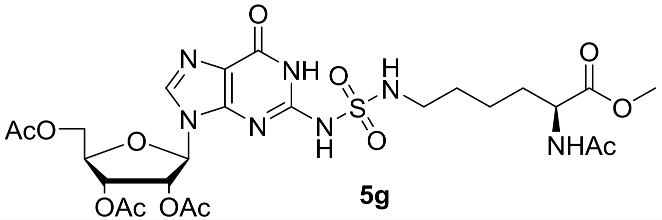

5g

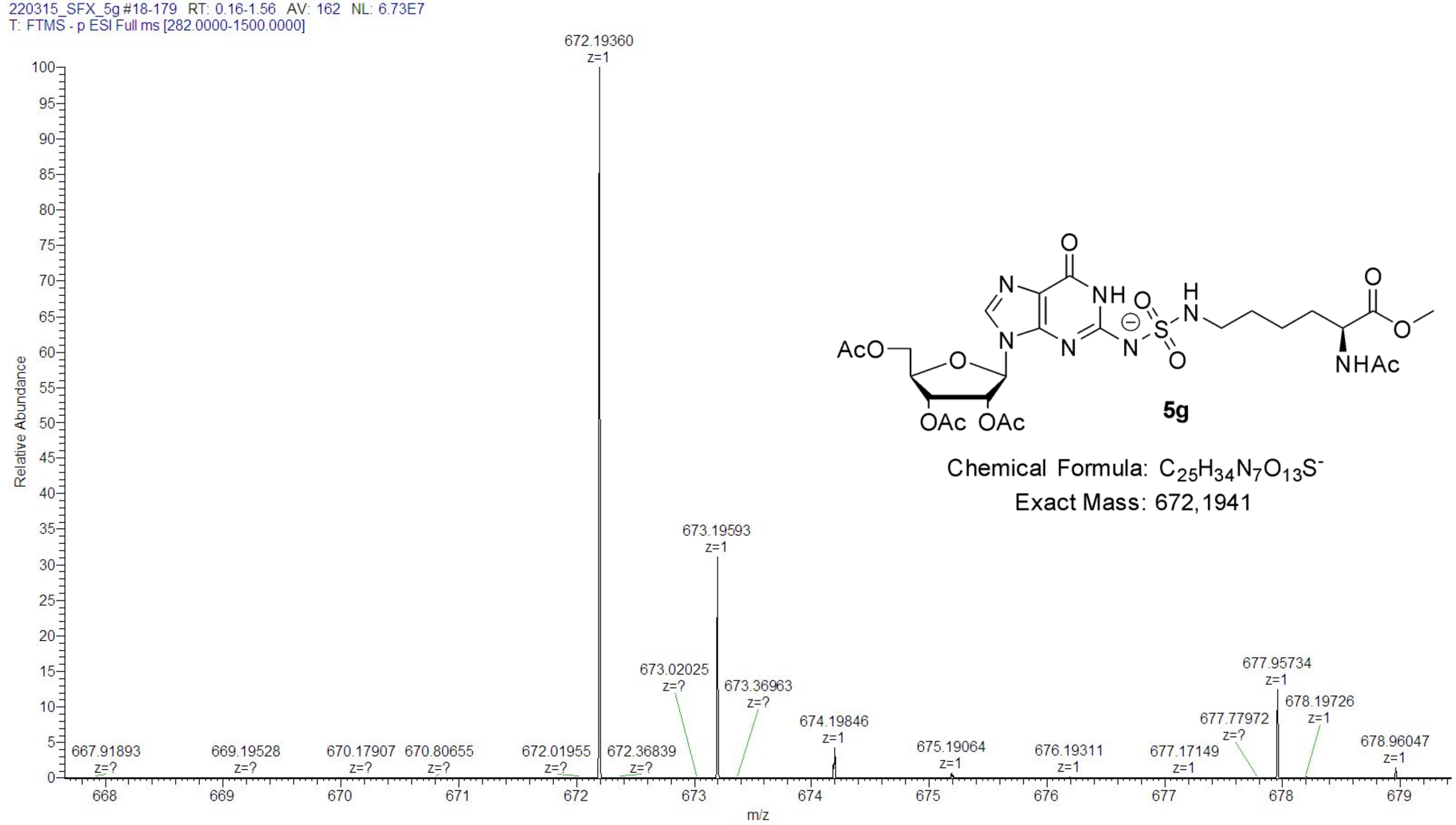

220315_SFX_5g #18-179 RT: 0.16-1.56 AV: 162 NL: 6.73E7
T: FTMS - p ESI Full ms [282.0000-1500.0000]
672.19360 z=1
673.19593 z=1
673.02025 z=?
673.36963 z=?
674.19846 z=1
675.19064 z=1
676.19311 z=1
677.17149 z=1
677.77972 z=?
677.95734 z=1
678.19726 z=1
678.96047 z=1
667.91893 z=?
669.19528 z=?
670.17907 z=?
670.80655 z=?
672.01955 z=?
672.36839 z=?
Relative Abundance
m/z
5g
Chemical Formula: $C_{25}H_{34}N_7O_{13}S^-$
Exact Mass: 672,1941

^{1}H NMR spectrum (500 MHz) of **5g**

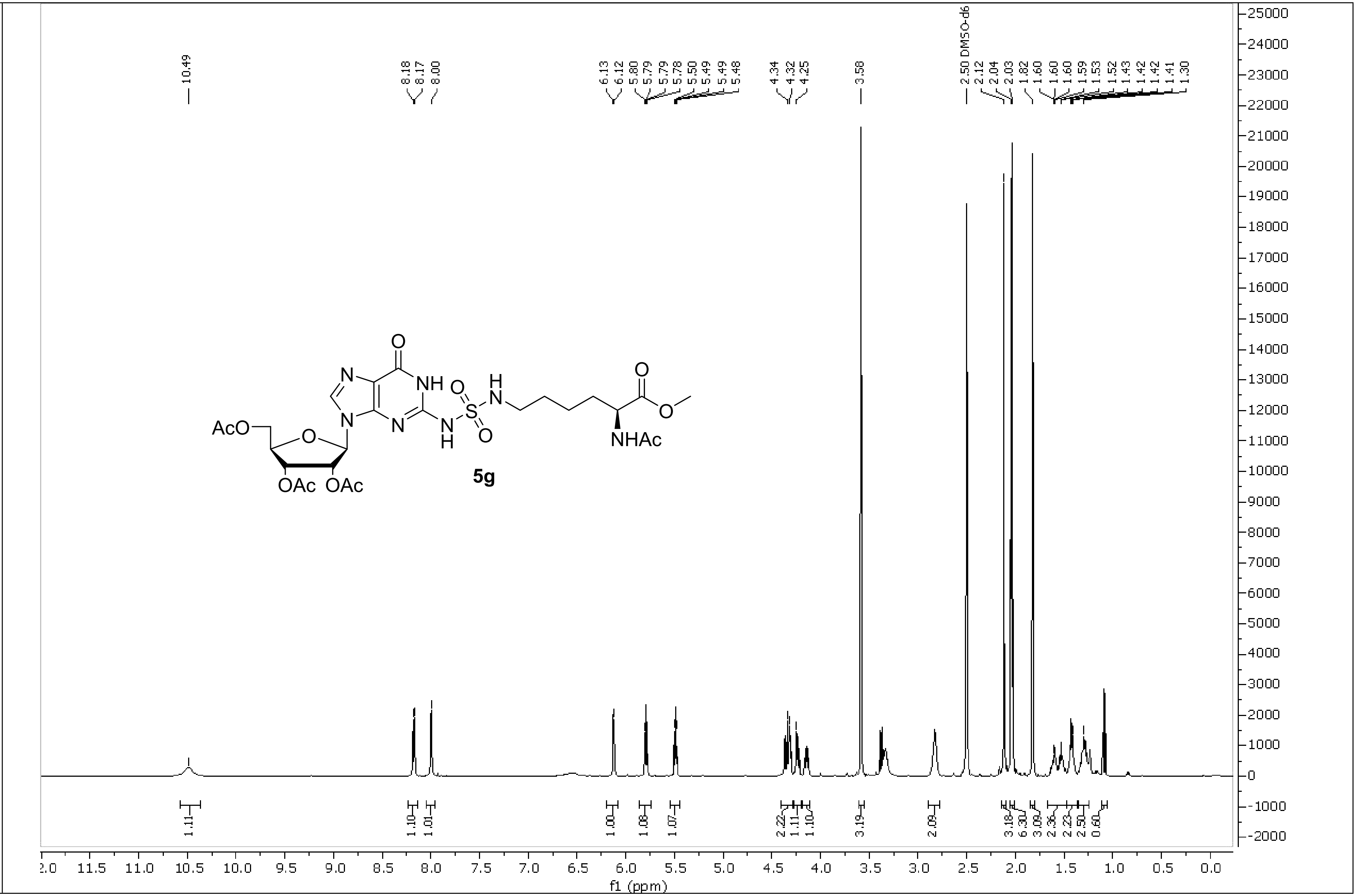

^{13}C NMR spectrum (126 MHz) of **5g**

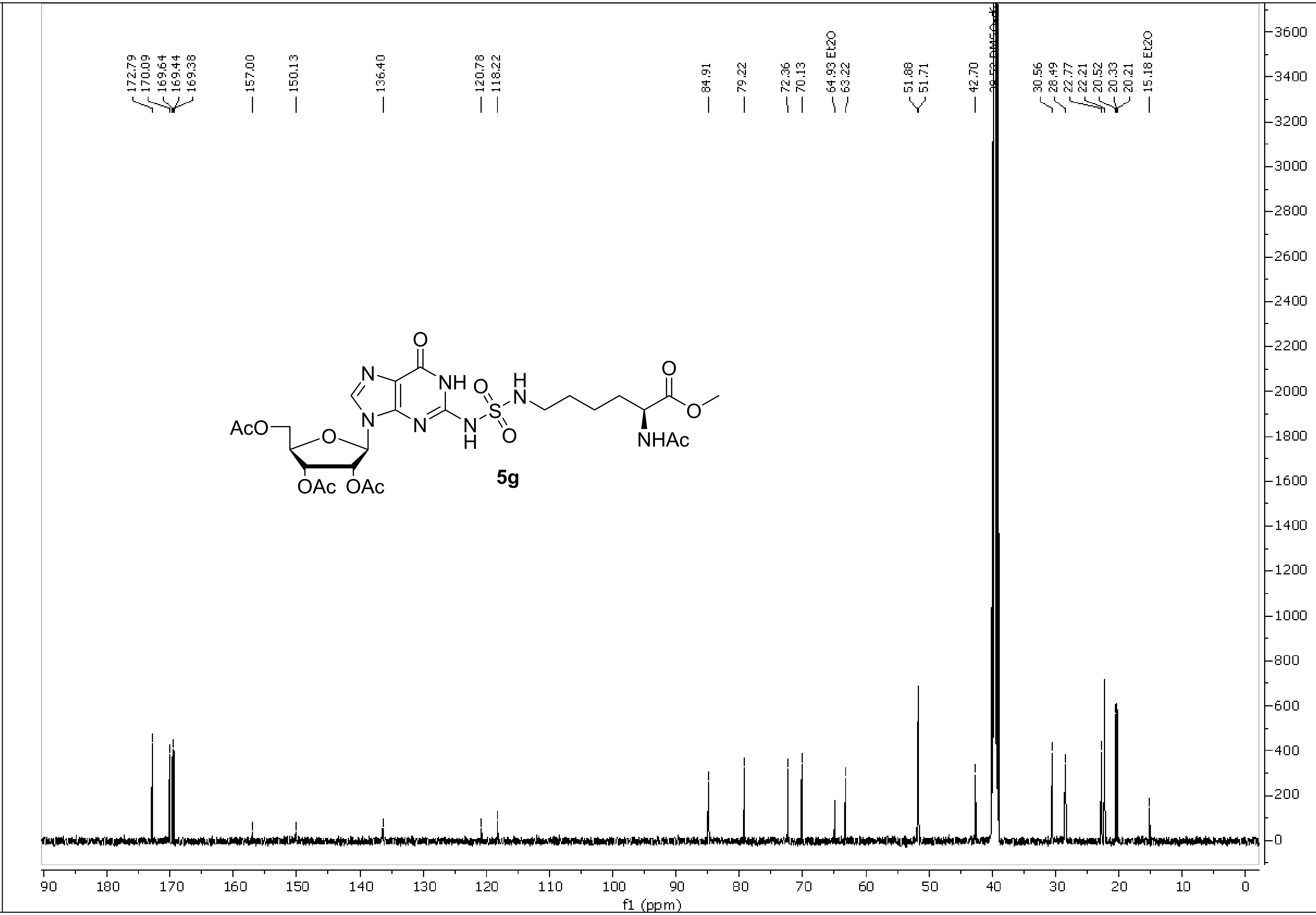

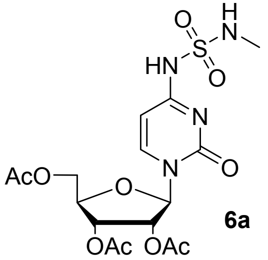

6a

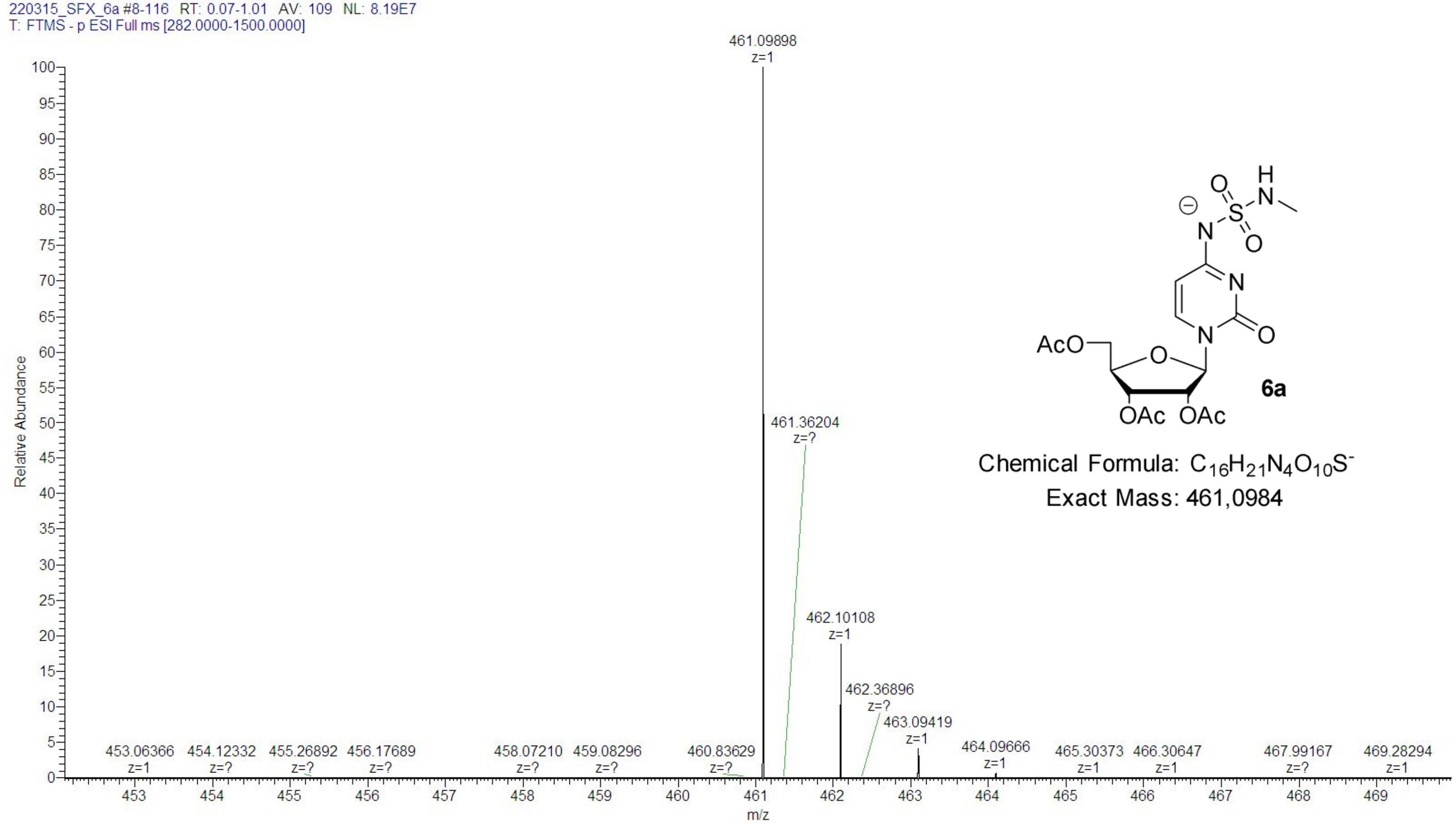

220315_SFX_6a #8-116 RT: 0.07-1.01 AV: 109 NL: 8.19E7
T: FTMS - p ESI Full ms [282.0000-1500.0000]
461.09898
z=1
461.36204
z=?
462.10108
z=1
462.36896
z=?
463.09419
z=1
Relative Abundance
m/z
6a
Chemical Formula: $C_{16}H_{21}N_4O_{10}S^-$
Exact Mass: 461,0984

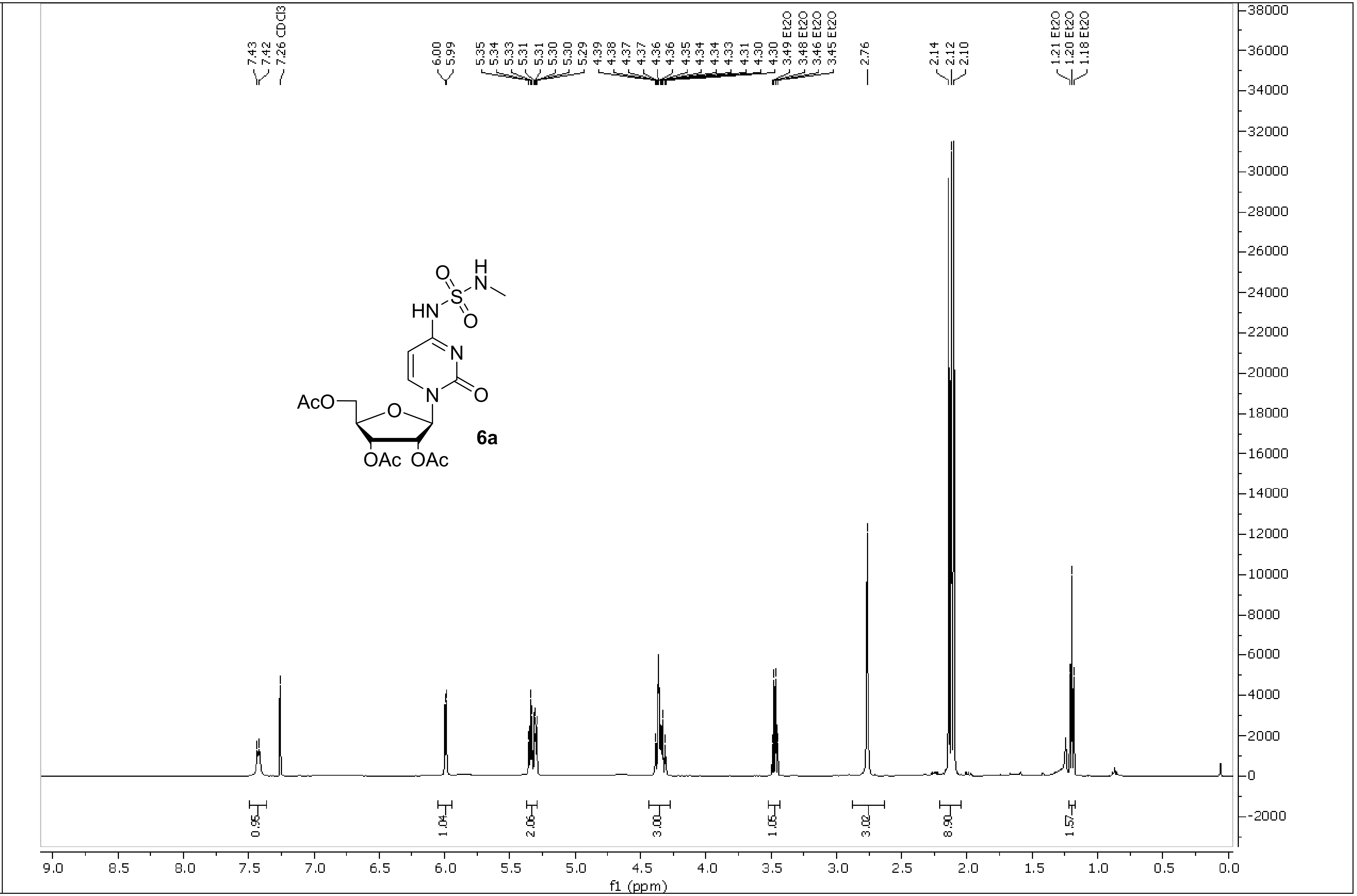

^{1}H NMR spectrum (500 MHz) of **6a**

^{13}C NMR spectrum (126 MHz) of **6a**

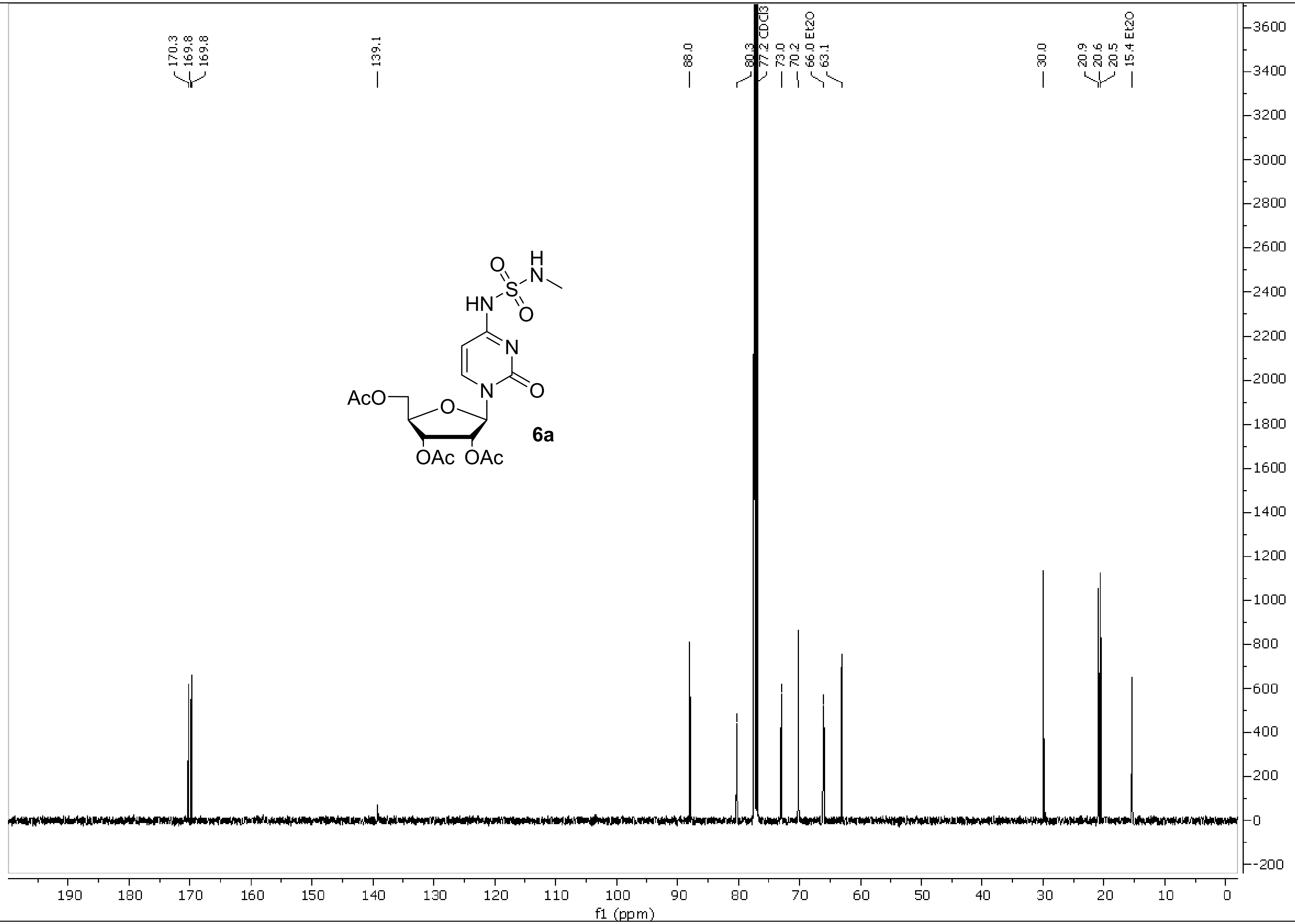

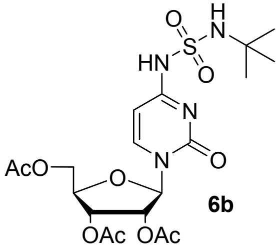

AcO
OAc
OAc
HN
O
S
N
H
6b

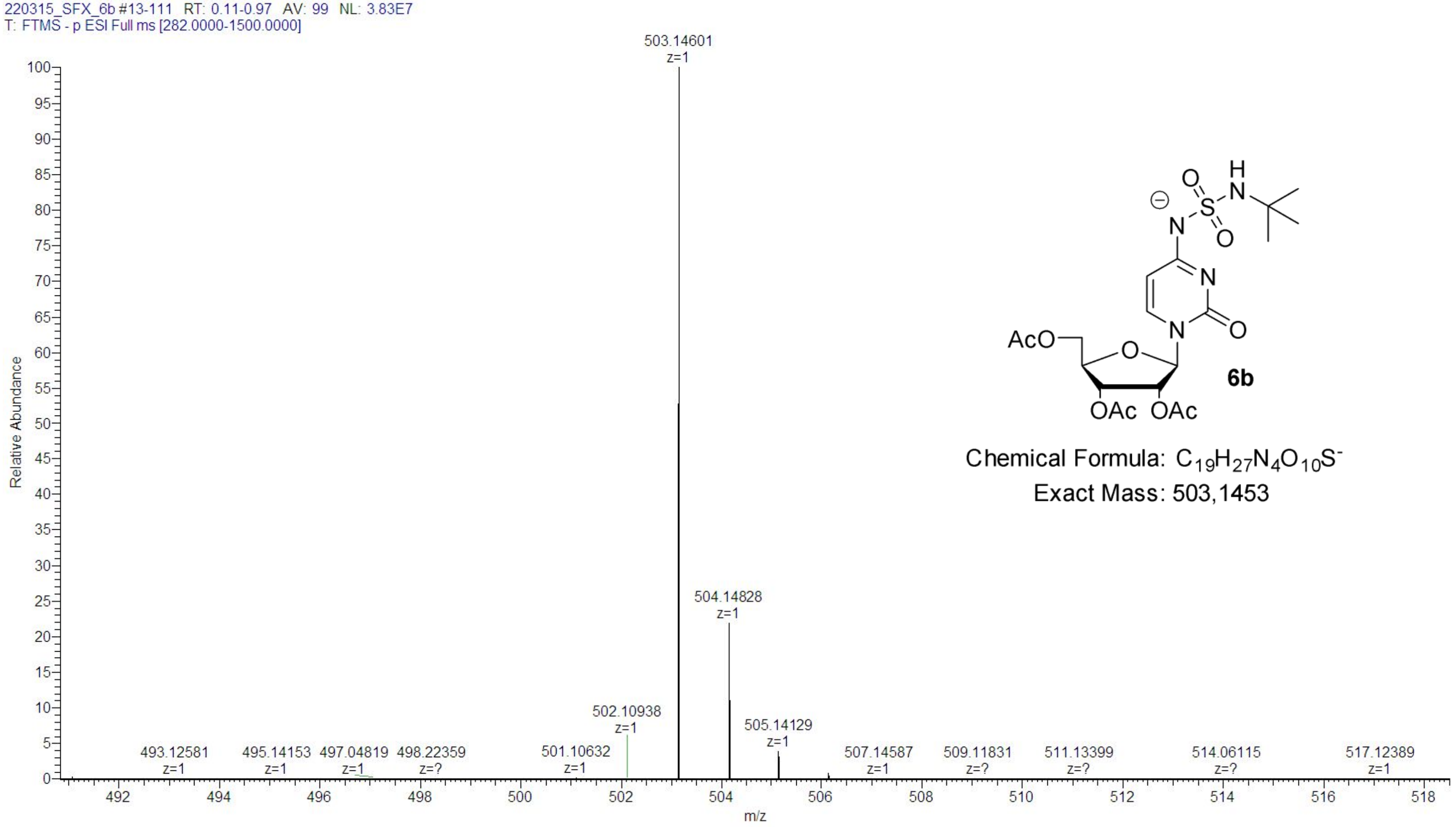

220315_SFX_6b #13-111 RT: 0.11-0.97 AV: 99 NL: 3.83E7
T: FTMS - p ESI Full ms [282.0000-1500.0000]
503.14601
z=1
504.14828
z=1
502.10938
z=1
505.14129
z=1
493.12581
495.14153
497.04819
498.22359
501.10632
507.14587
509.11831
511.13399
514.06115
517.12389
Relative Abundance
m/z
6b
Chemical Formula: $C_{19}H_{27}N_4O_{10}S^-$
Exact Mass: 503,1453

^{1}H NMR spectrum (500 MHz) of **6b**

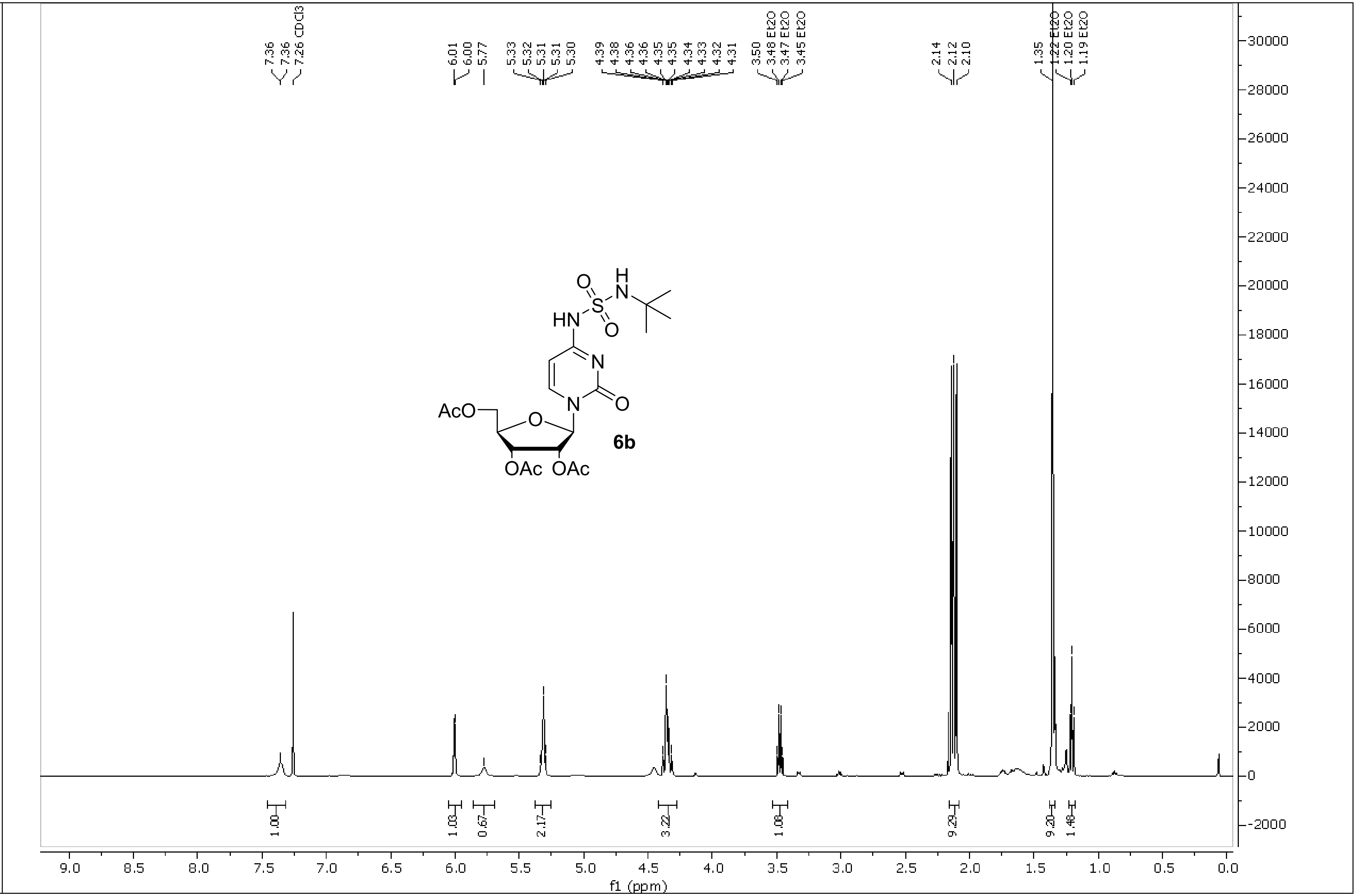

^{13}C NMR spectrum (126 MHz) of **6b**

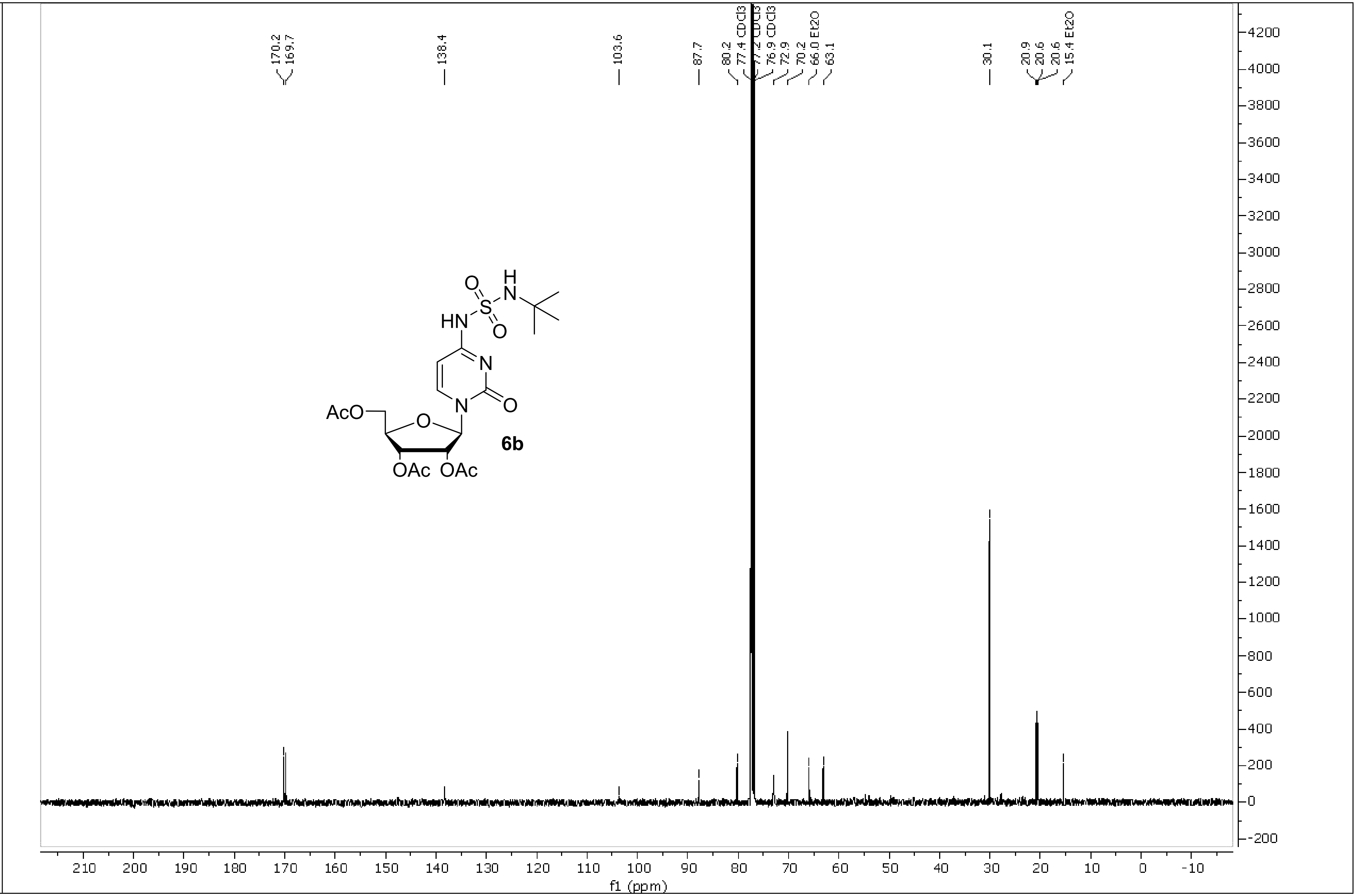

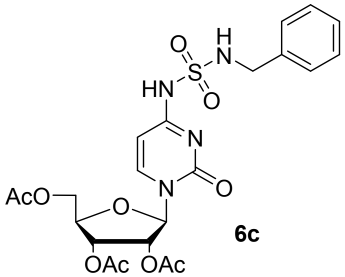

6c

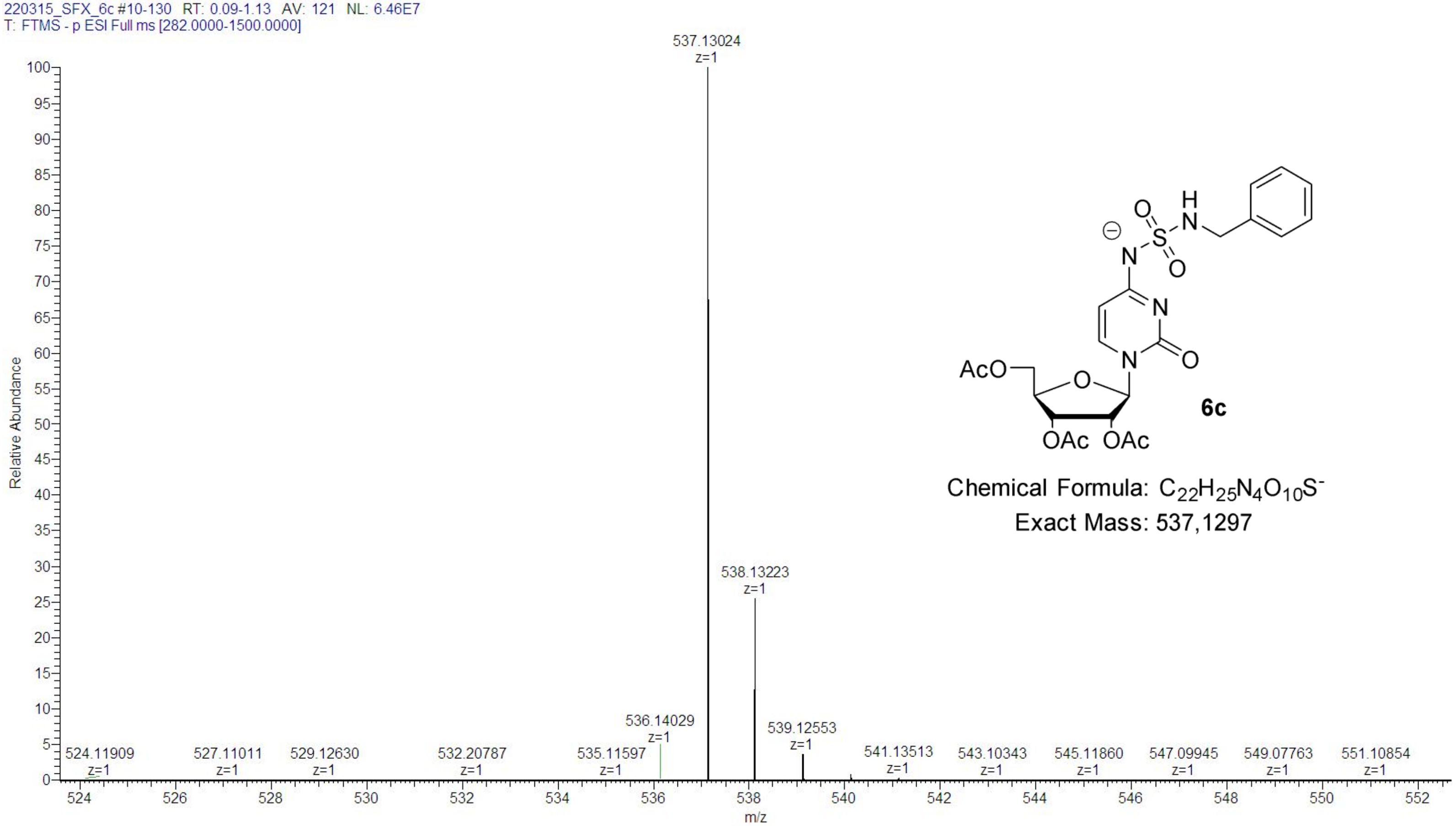

220315_SFX_6c #10-130 RT: 0.09-1.13 AV: 121 NL: 6.46E7
T: FTMS - p ESI Full ms [282.0000-1500.0000]
537.13024 z=1
538.13223 z=1
539.12553 z=1
536.14029 z=1
524.11909 z=1
527.11011 z=1
529.12630 z=1
532.20787 z=1
535.11597 z=1
541.13513 z=1
543.10343 z=1
545.11860 z=1
547.09945 z=1
549.07763 z=1
551.10854 z=1
Relative Abundance
m/z
6c
Chemical Formula: $C_{22}H_{25}N_4O_{10}S^-$
Exact Mass: 537,1297

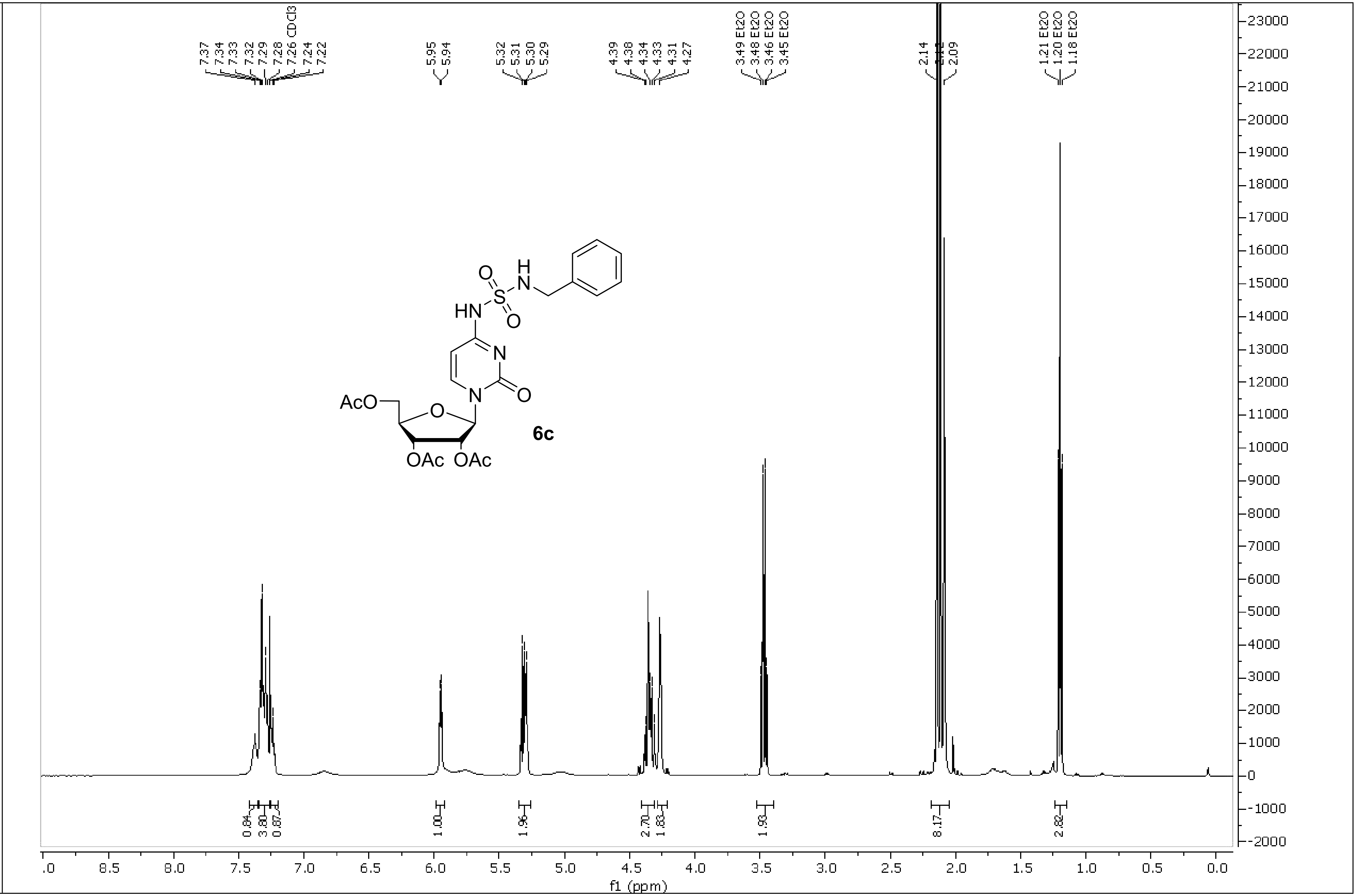

^{1}H NMR spectrum (500 MHz) of **6c**

^{13}C NMR spectrum (126 MHz) of **6c**

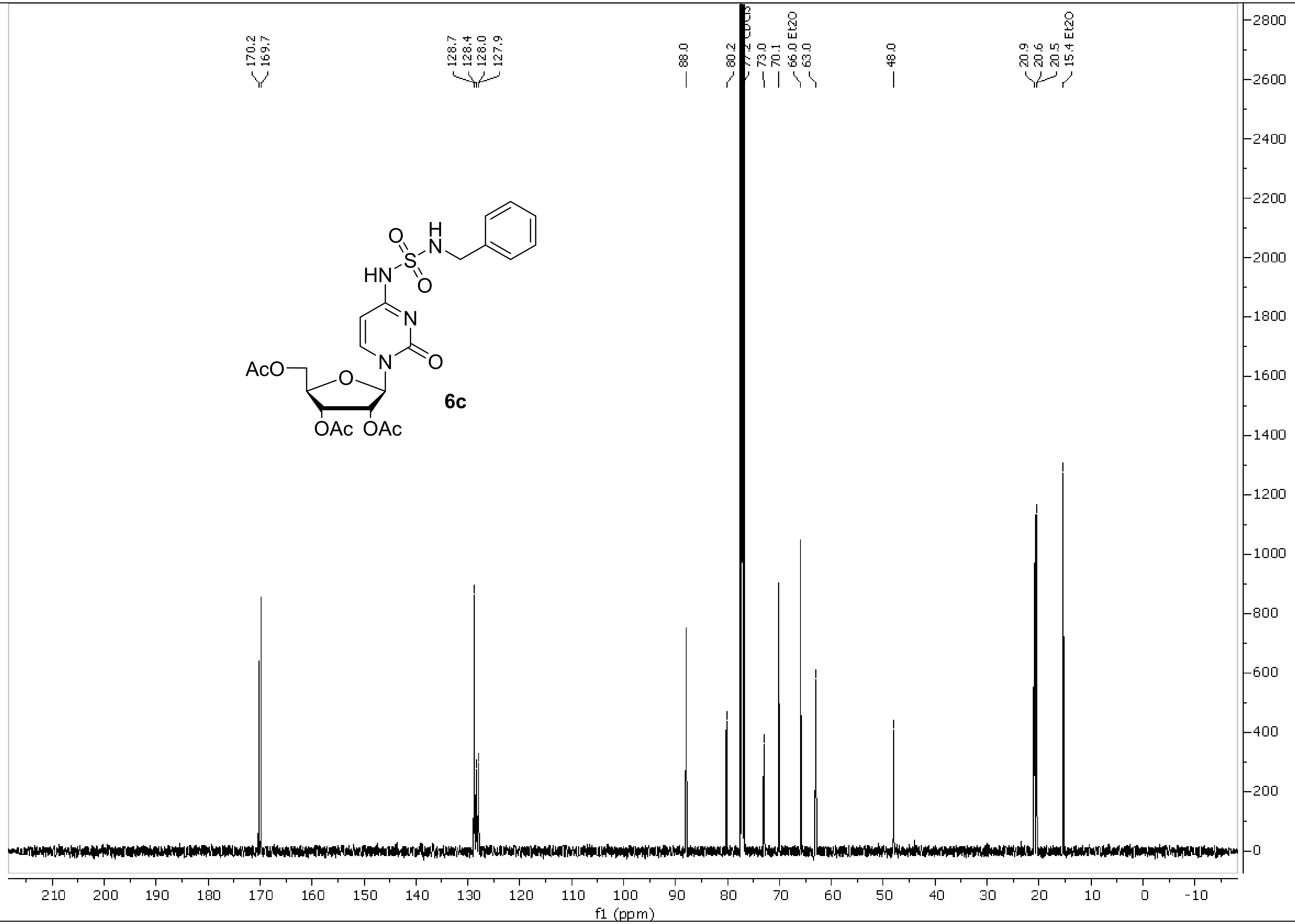

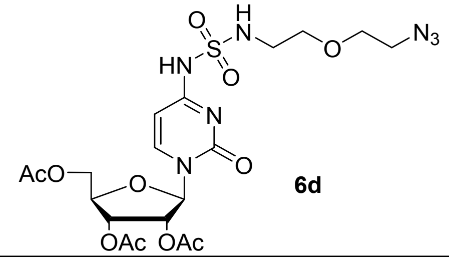

HN
N
N
O
AcO
OAc
OAc
N3

6d

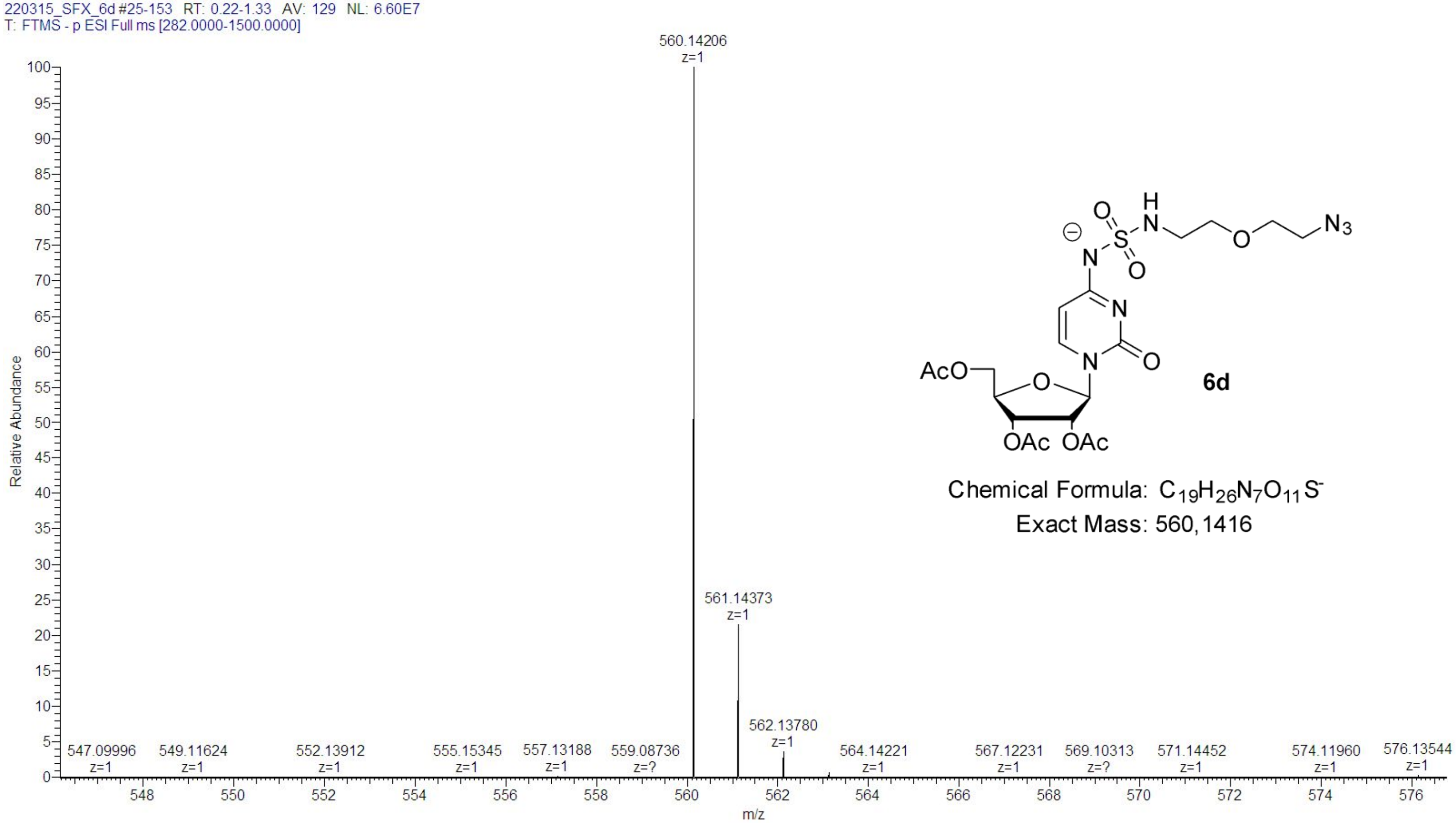

220315_SFX_6d #25-153 RT: 0.22-1.33 AV: 129 NL: 6.60E7
T: FTMS - p ESI Full ms [282.0000-1500.0000]
560.14206 z=1
561.14373 z=1
562.13780 z=1
547.09996 z=1
549.11624 z=1
552.13912 z=1
555.15345 z=1
557.13188 z=1
559.08736 z=?
564.14221 z=1
567.12231 z=1
569.10313 z=?
571.14452 z=1
574.11960 z=1
576.13544 z=1
Relative Abundance
m/z
6d
Chemical Formula: C19H26N7O11S-
Exact Mass: 560,1416

^{1}H NMR spectrum (500 MHz) of **6d**

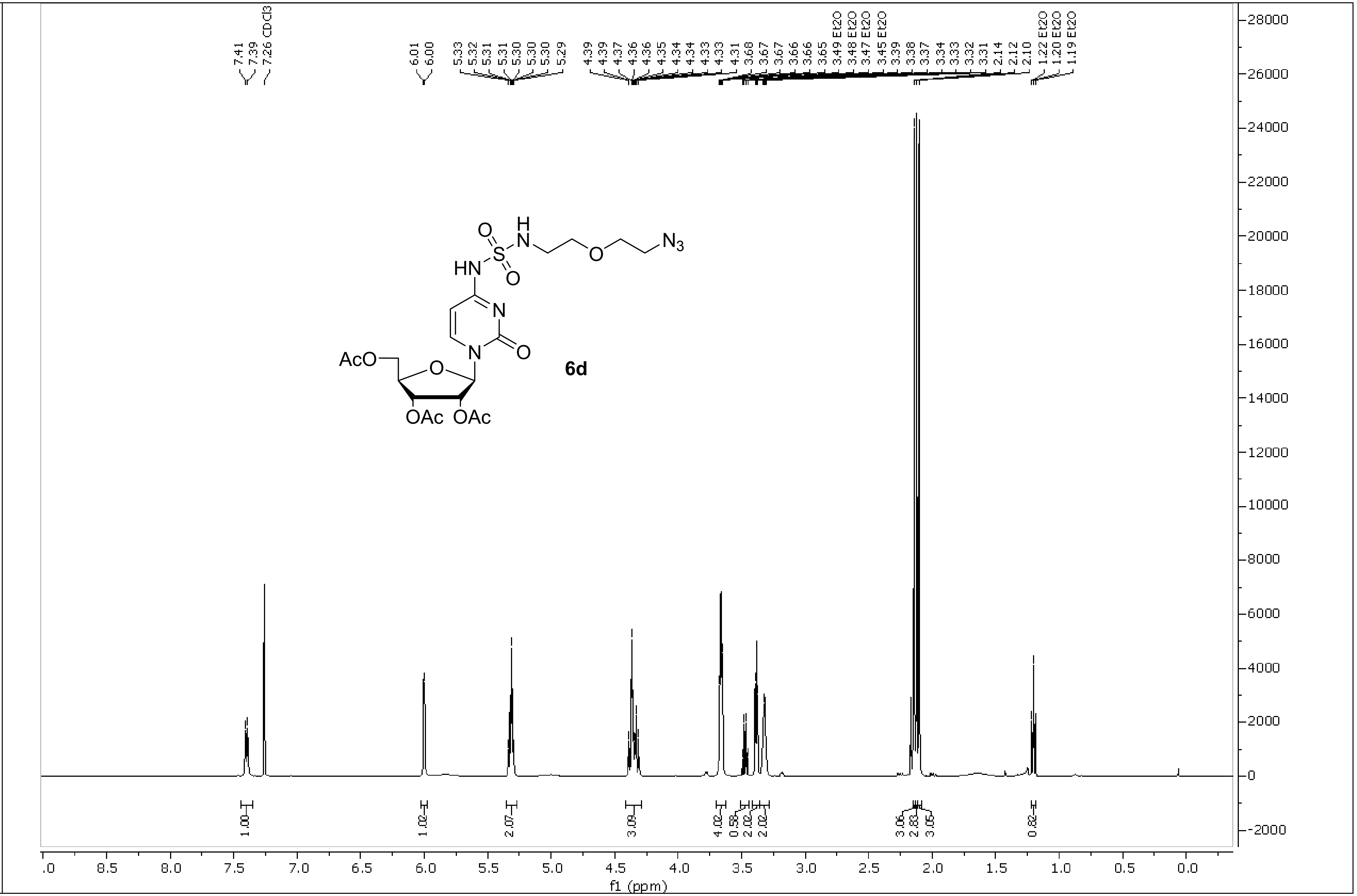

^{13}C NMR spectrum (126 MHz) of **6d**

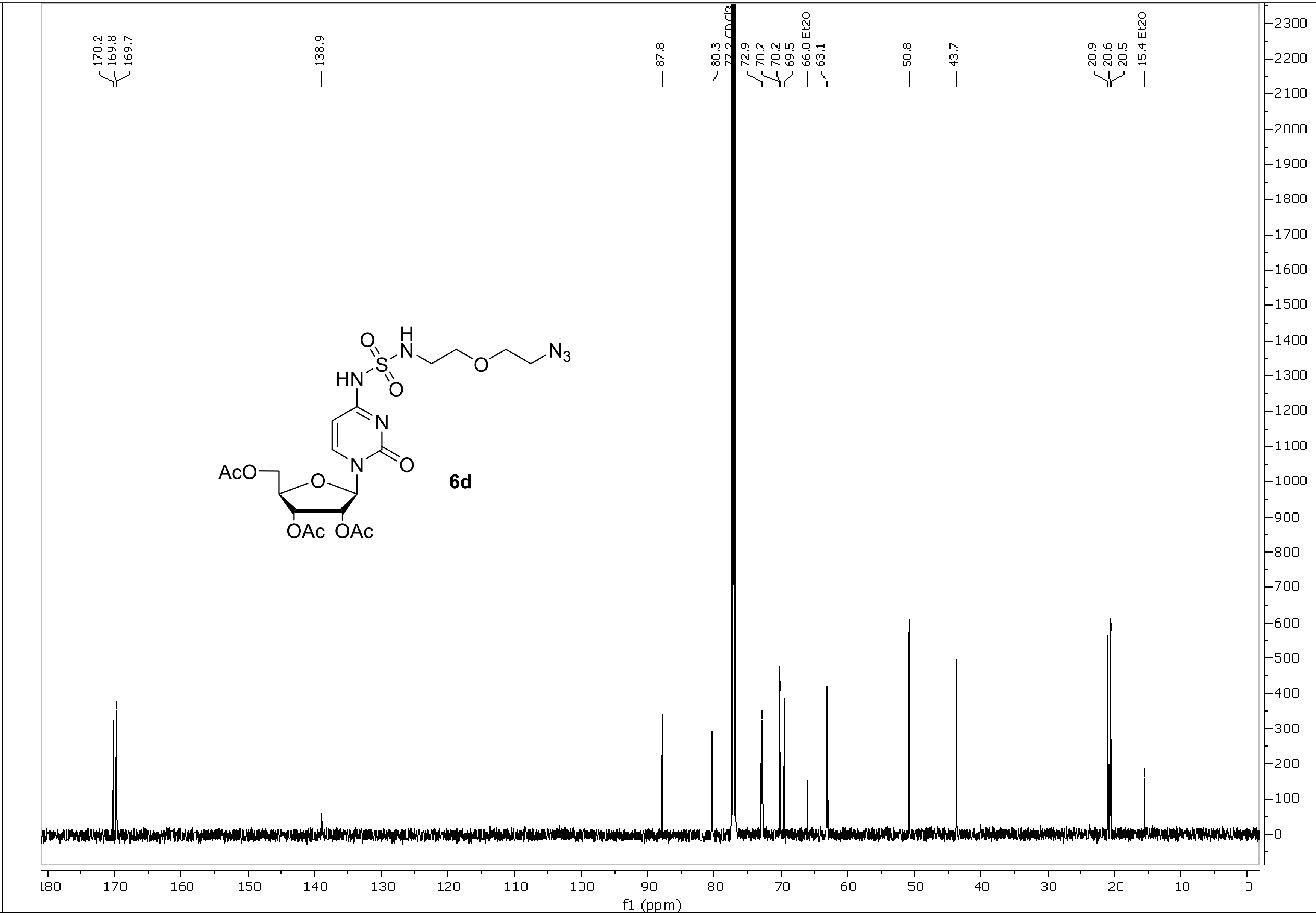

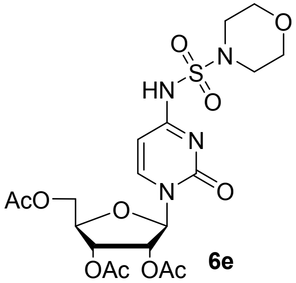

6e

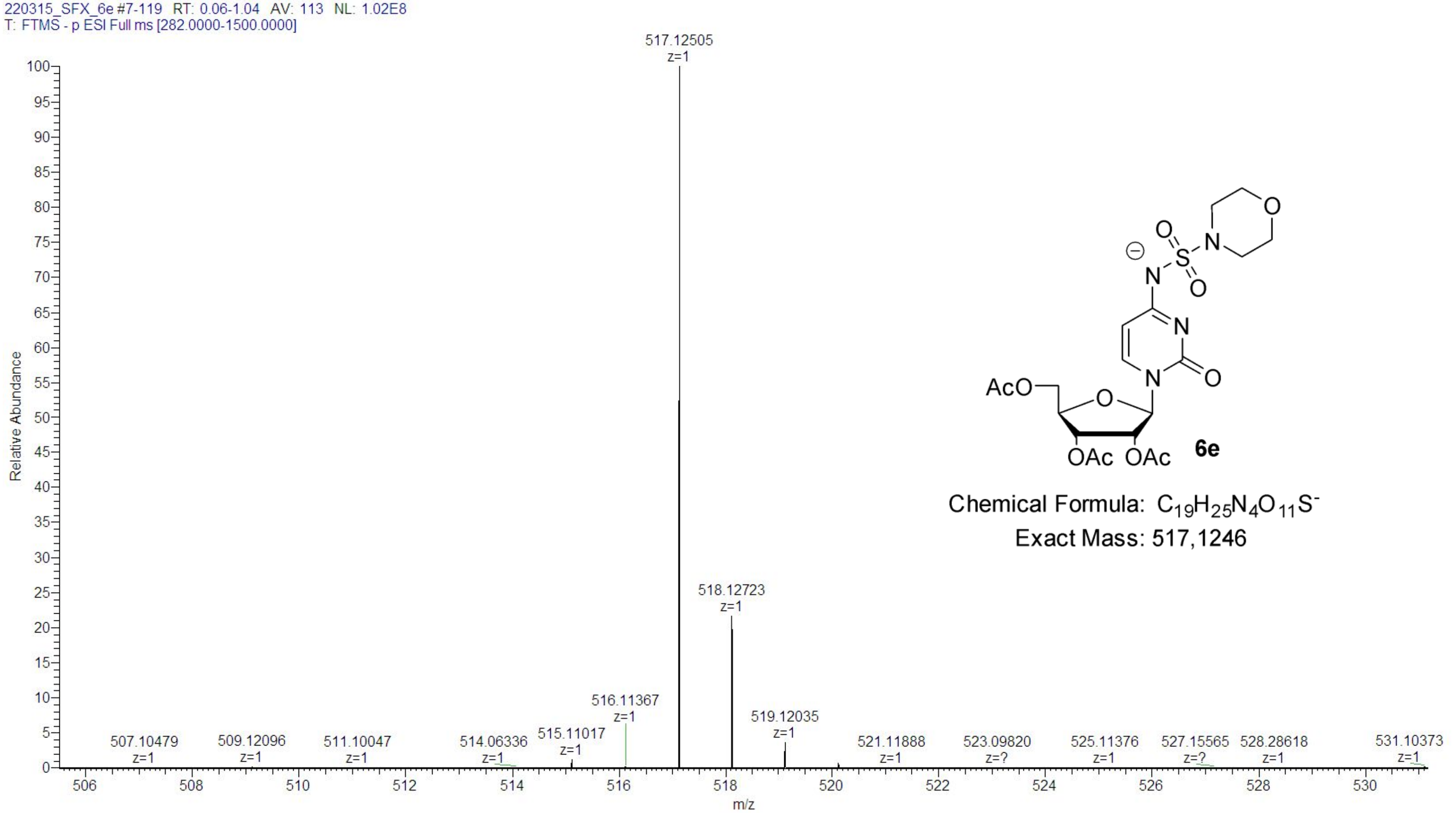

220315_SFX_6e #7-119 RT: 0.06-1.04 AV: 113 NL: 1.02E8
T: FTMS - p ESI Full ms [282.0000-1500.0000]
517.12505 z=1
518.12723 z=1
519.12035 z=1
516.11367 z=1
515.11017 z=1
514.06336 z=1
507.10479 z=1
509.12096 z=1
511.10047 z=1
521.11888 z=1
523.09820 z=?
525.11376 z=1
527.15565 z=?
528.28618 z=1
531.10373 z=1
Relative Abundance
m/z
6e
Chemical Formula: C19H25N4O11S-
Exact Mass: 517,1246

^{1}H NMR spectrum (500 MHz) of **6e**

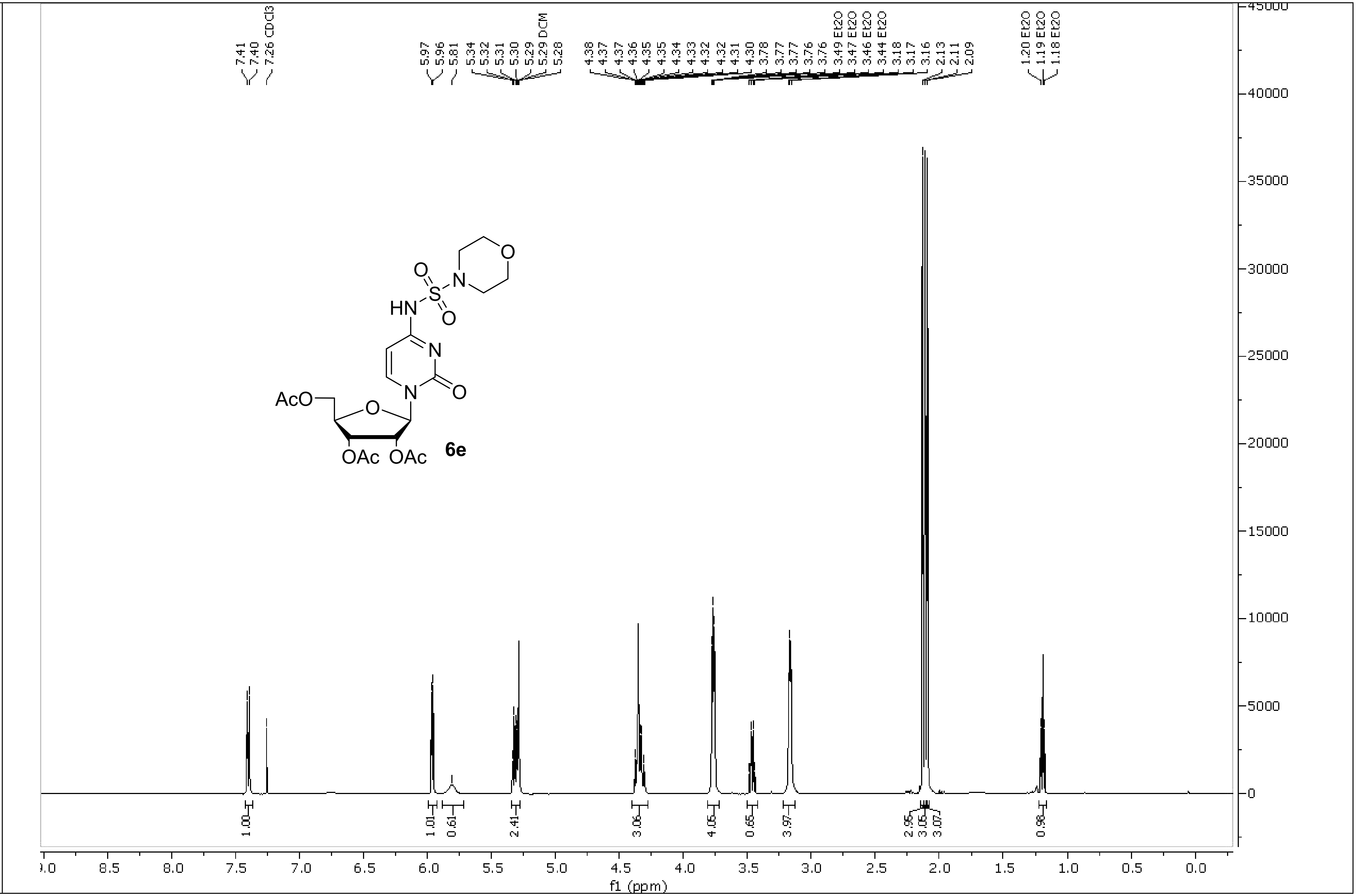

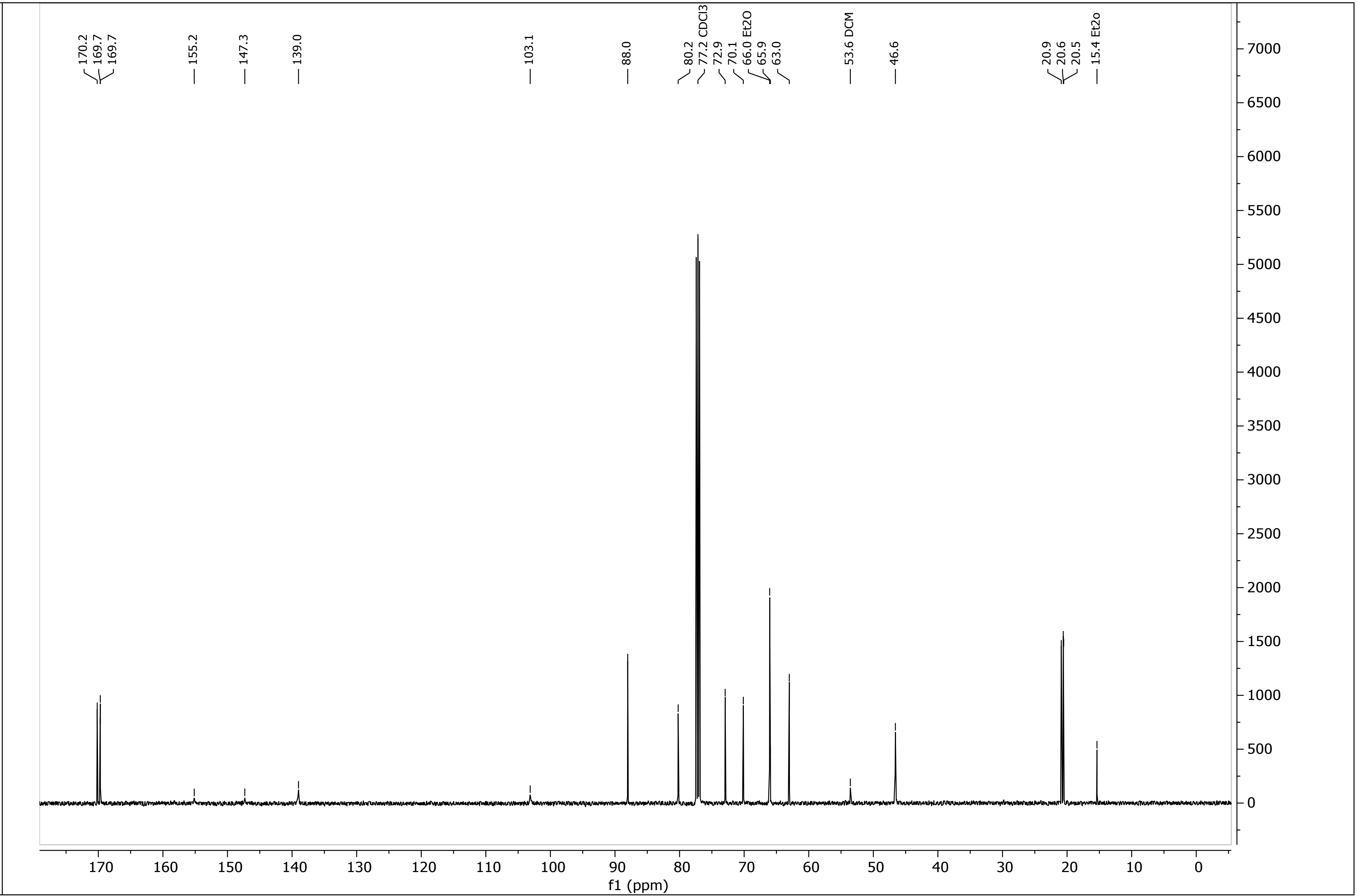

^{13}C NMR spectrum (126 MHz) of **6e**

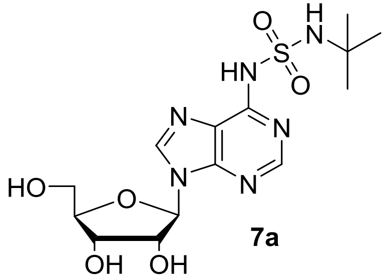

7a

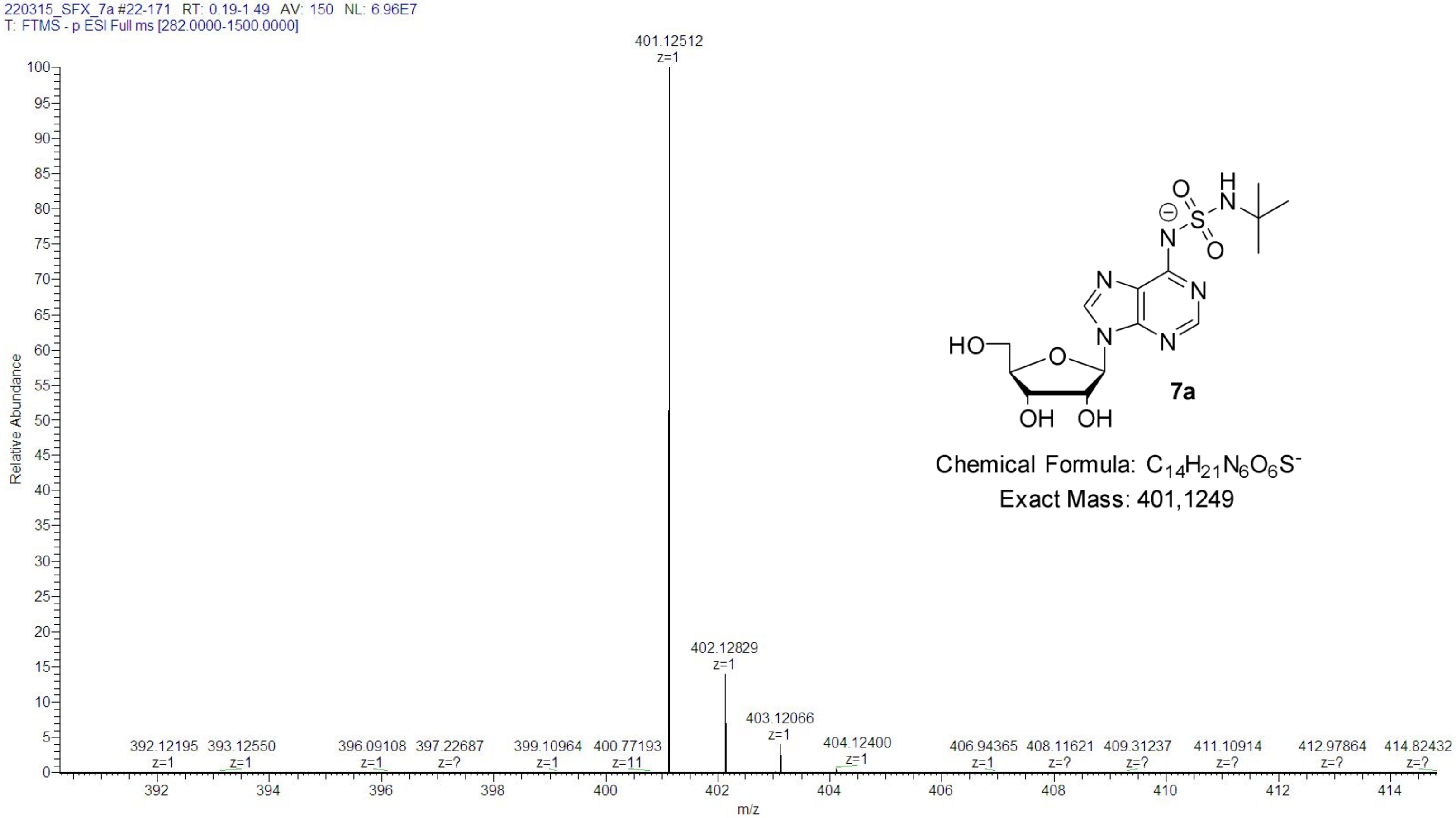

220315_SFX_7a #22-171 RT: 0.19-1.49 AV: 150 NL: 6.96E7
T: FTMS - p ESI Full ms [282.0000-1500.0000]
401.12512
z=1
402.12829
z=1
403.12066
z=1
404.12400
z=1
392.12195
z=1
393.12550
z=1
396.09108
z=1
397.22687
z=?
399.10964
z=1
400.77193
z=11
406.94365
z=1
408.11621
z=?
409.31237
z=?
411.10914
z=?
412.97864
z=?
414.82432
z=?
Relative Abundance
m/z
7a
Chemical Formula: $C_{14}H_{21}N_6O_6S^-$
Exact Mass: 401,1249

^{1}H NMR spectrum (500 MHz) of **7a**

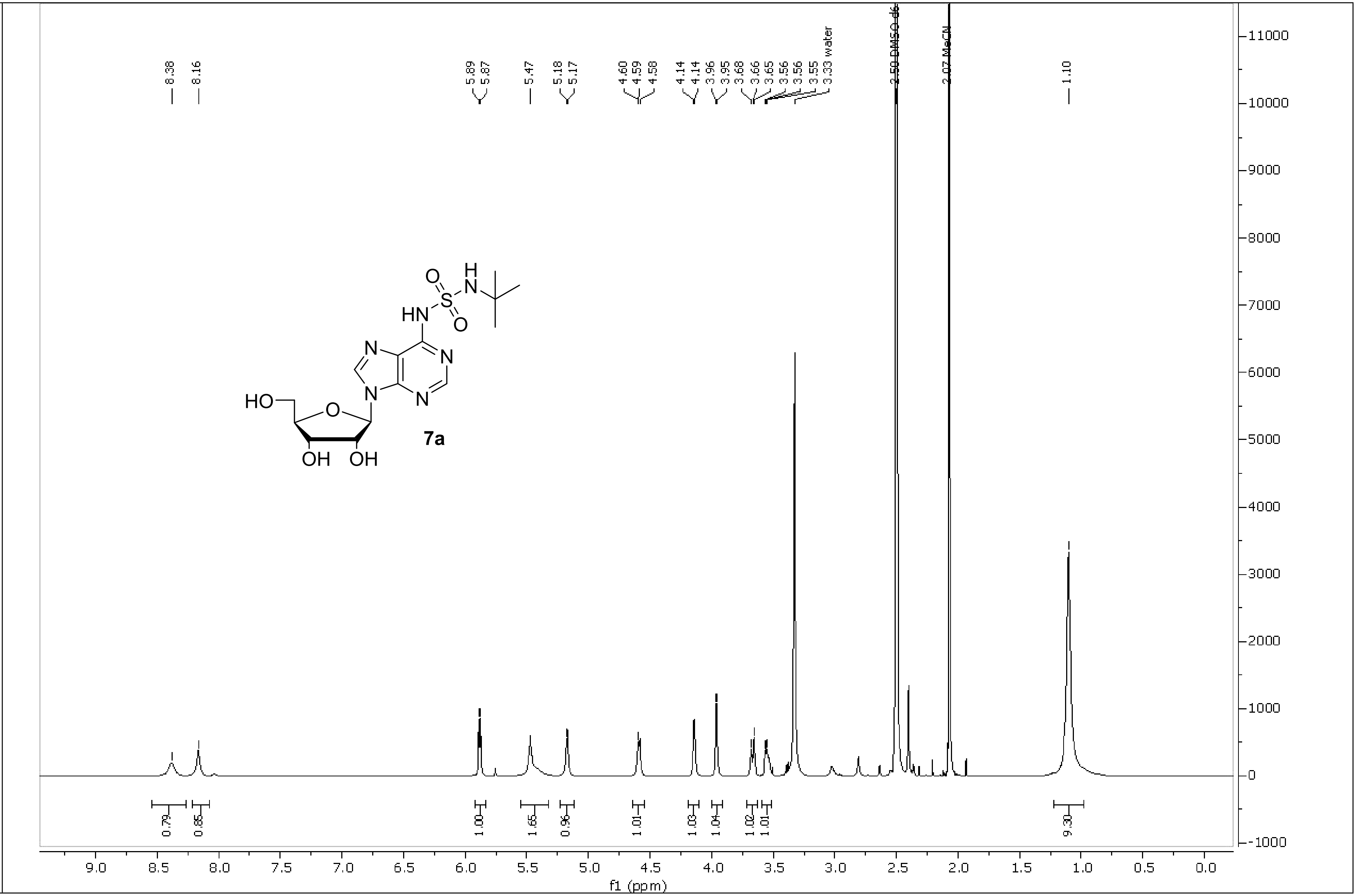

^{13}C NMR spectrum (126 MHz) of **7a**

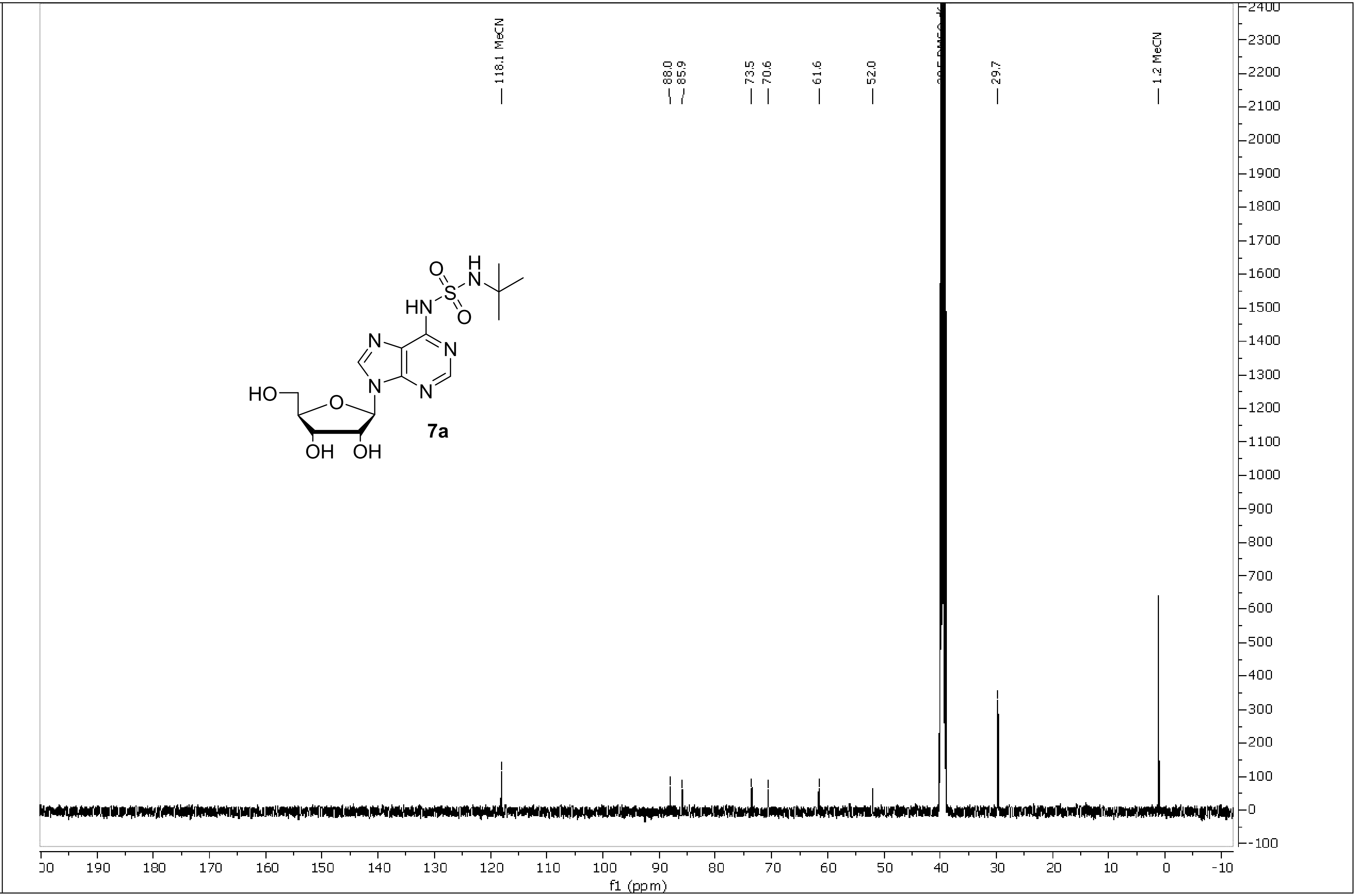

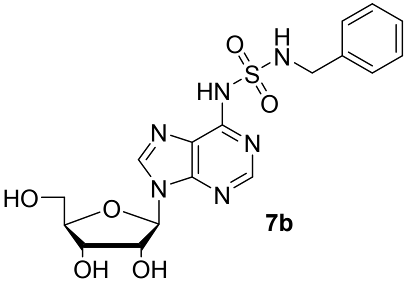

7b

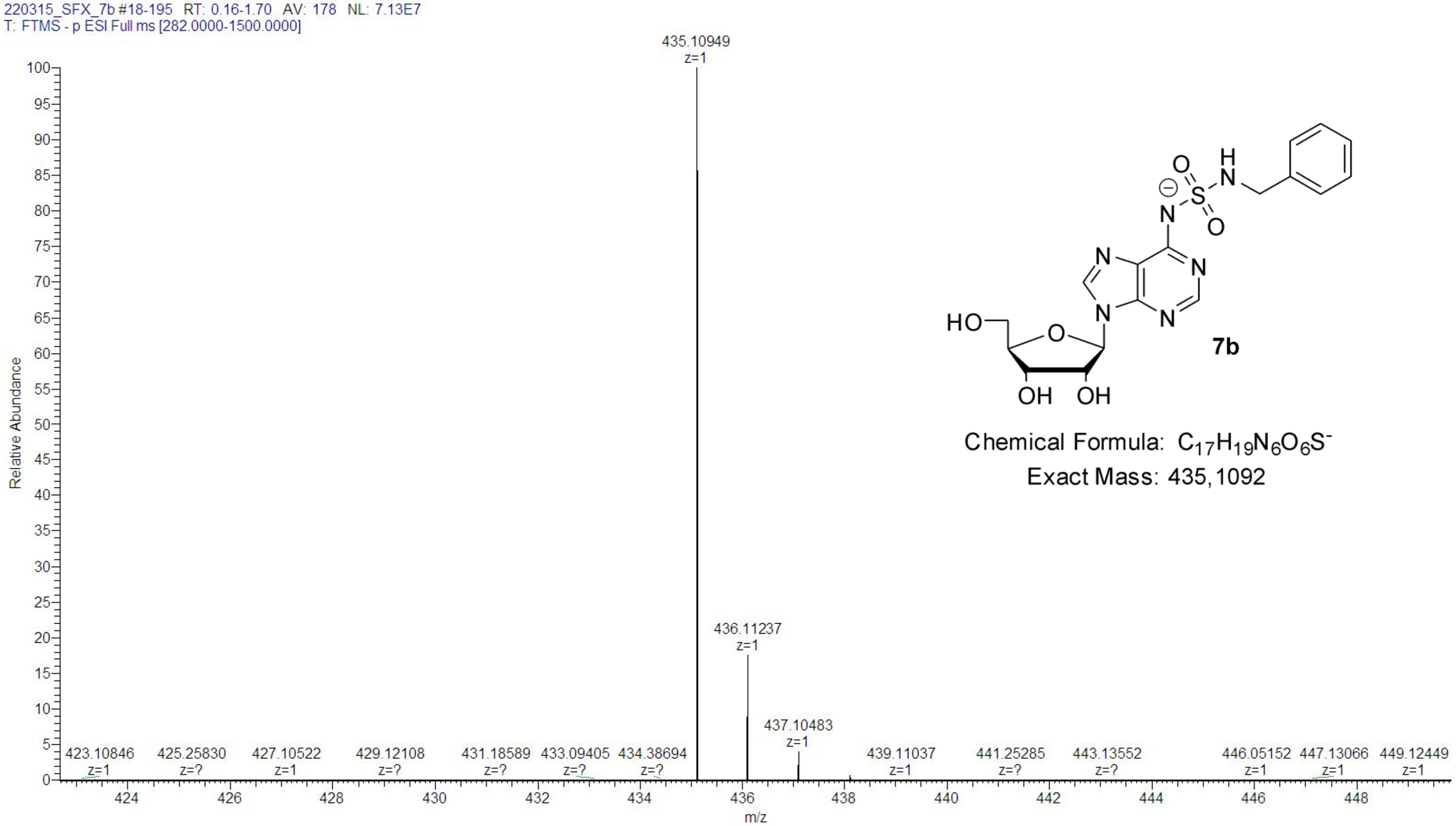

220315_SFX_7b #18-195 RT: 0.16-1.70 AV: 178 NL: 7.13E7
T: FTMS - p ESI Full ms [282.0000-1500.0000]
435.10949
z=1
436.11237
z=1
437.10483
z=1
Relative Abundance
m/z
7b
Chemical Formula: $C_{17}H_{19}N_6O_6S^-$
Exact Mass: 435,1092

^{1}H NMR spectrum (500 MHz) of **7b**

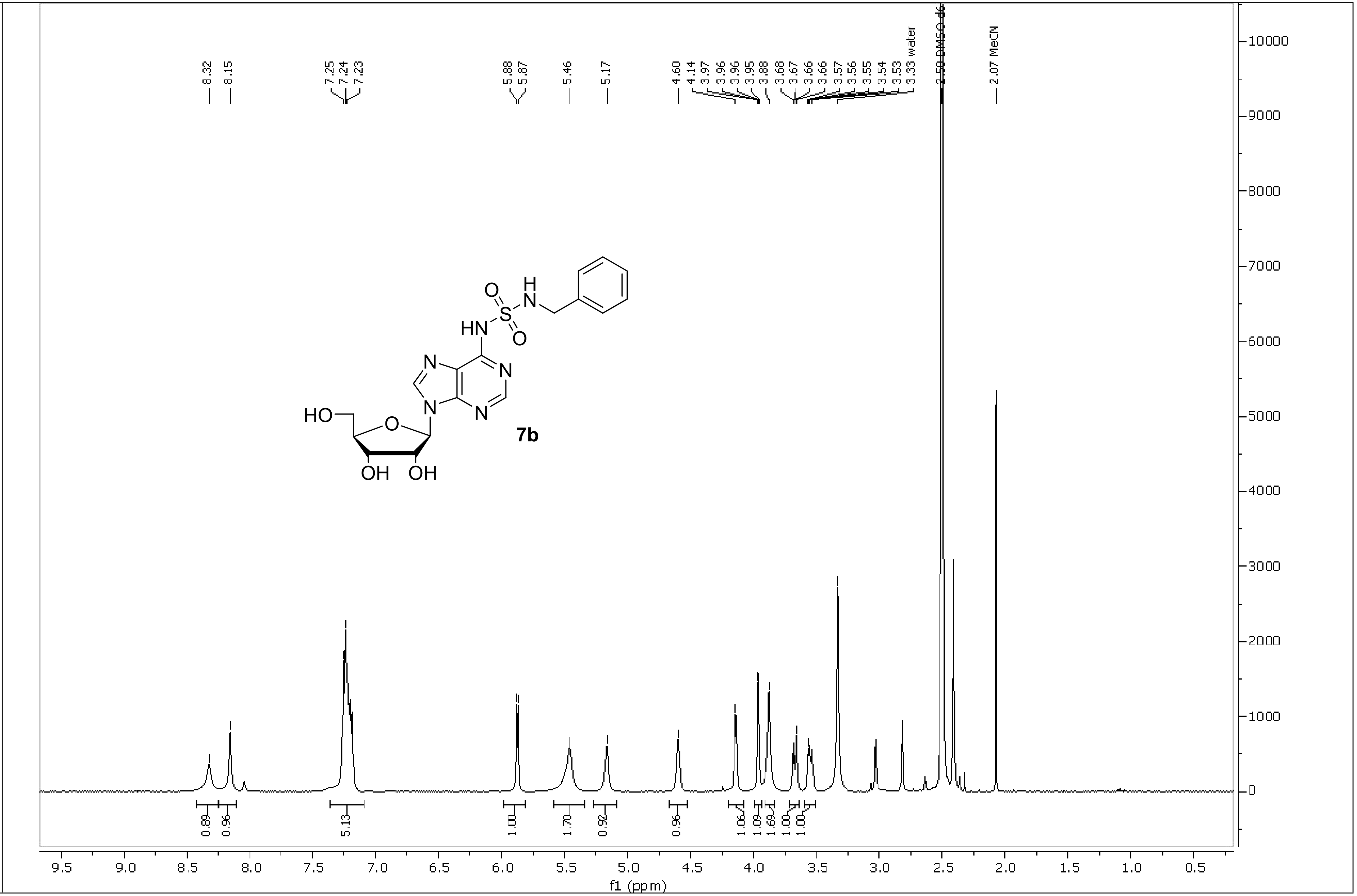

^{1}H NMR spectrum (500 MHz) of **7b**

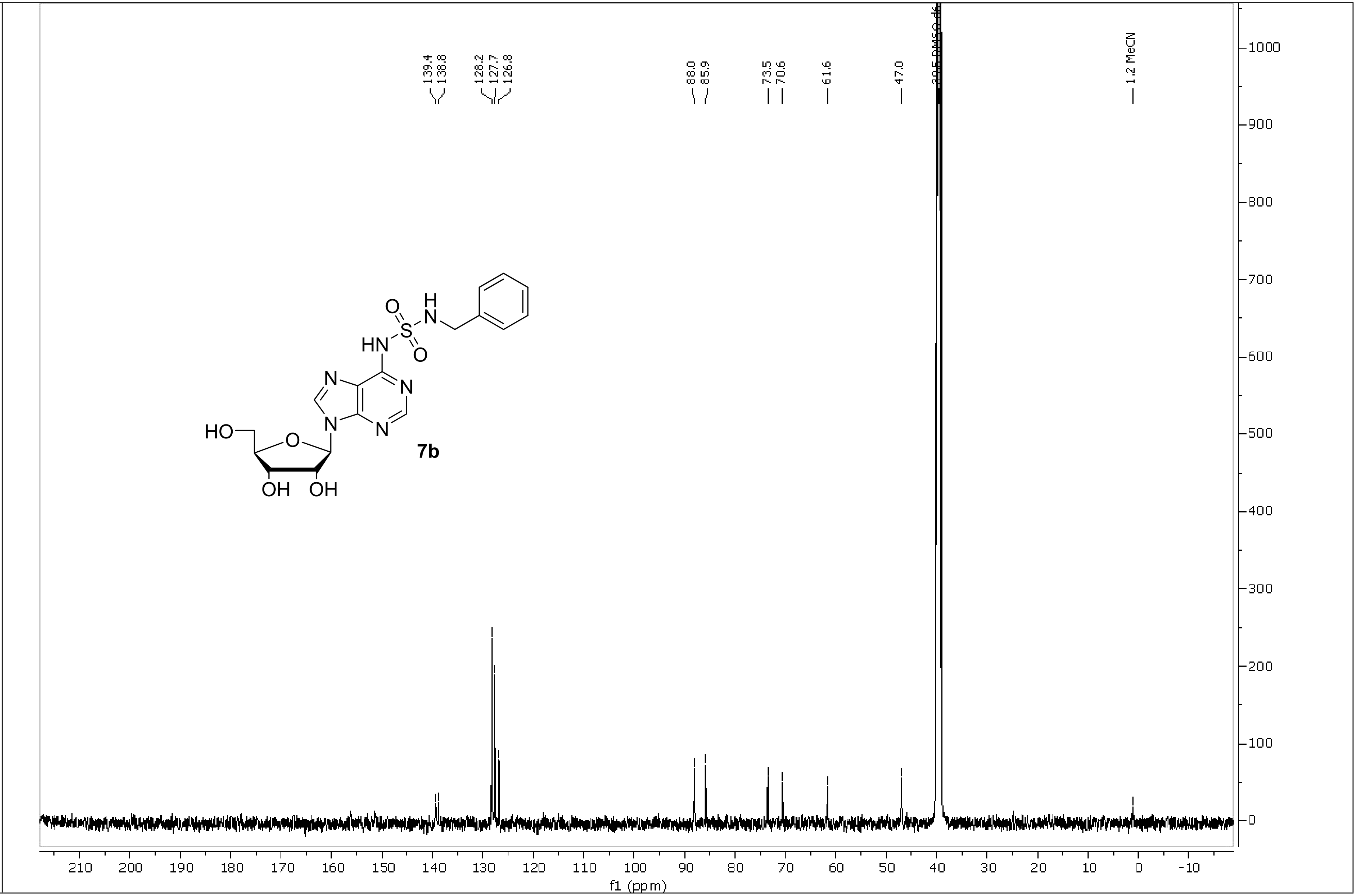

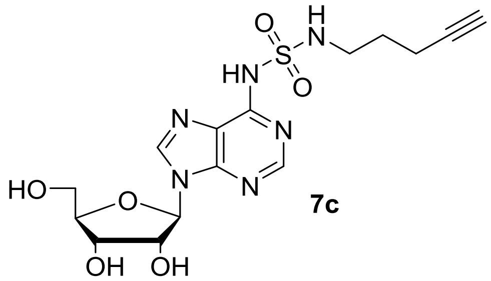
7c

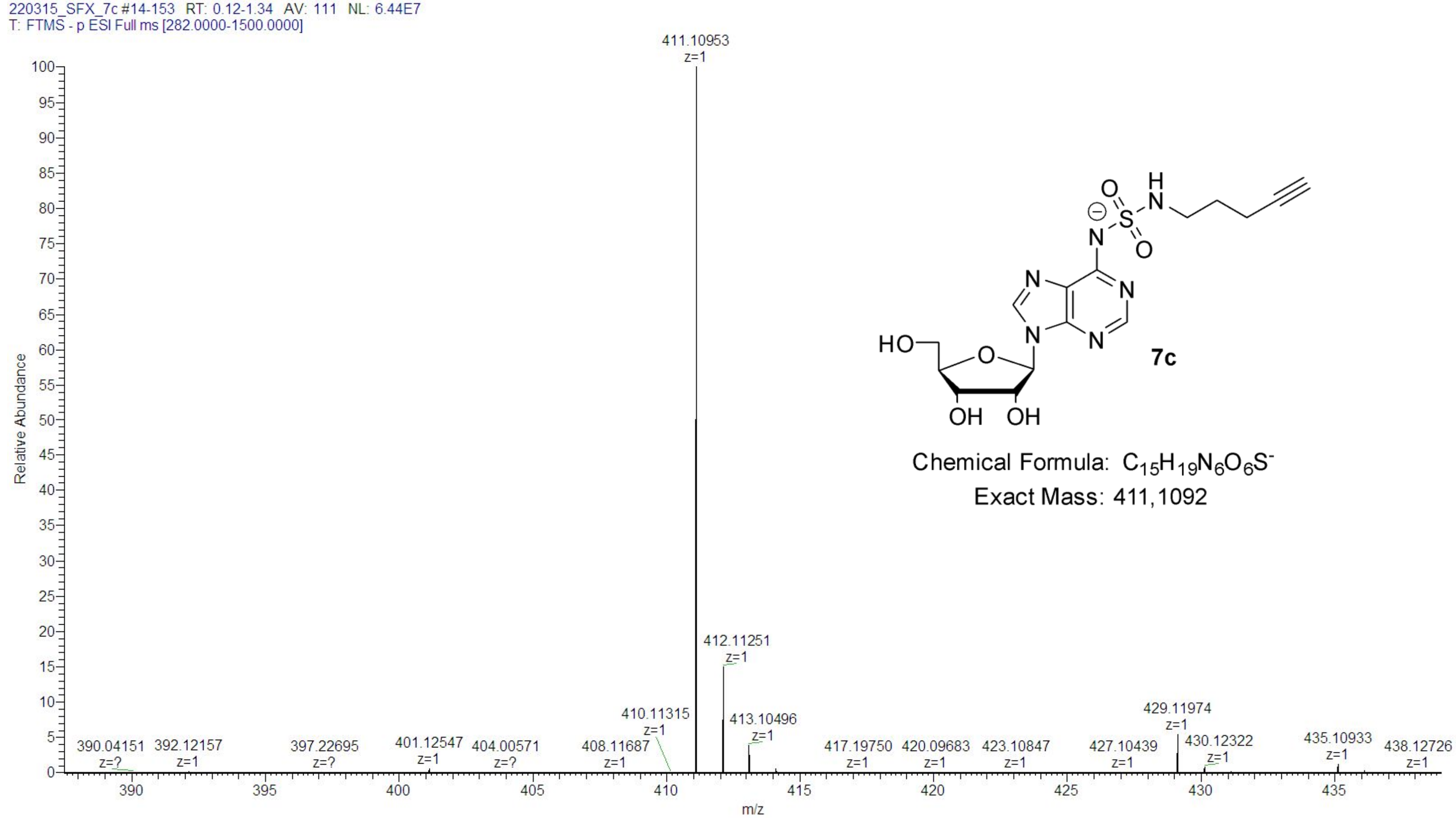
220315_SFX_7c #14-153 RT: 0.12-1.34 AV: 111 NL: 6.44E7
T: FTMS - p ESI Full ms [282.0000-1500.0000]
411.10953 z=1
412.11251 z=1
413.10496 z=1
410.11315 z=1
429.11974 z=1
430.12322 z=1
435.10933 z=1
438.12726 z=1
390.04151 z=?
392.12157 z=1
397.22695 z=?
401.12547 z=1
404.00571 z=?
408.11687 z=1
417.19750 z=1
420.09683 z=1
423.10847 z=1
427.10439 z=1
Relative Abundance
m/z
7c
Chemical Formula: $C_{15}H_{19}N_6O_6S^-$
Exact Mass: 411,1092

^1H NMR spectrum (500 MHz) of **7c**

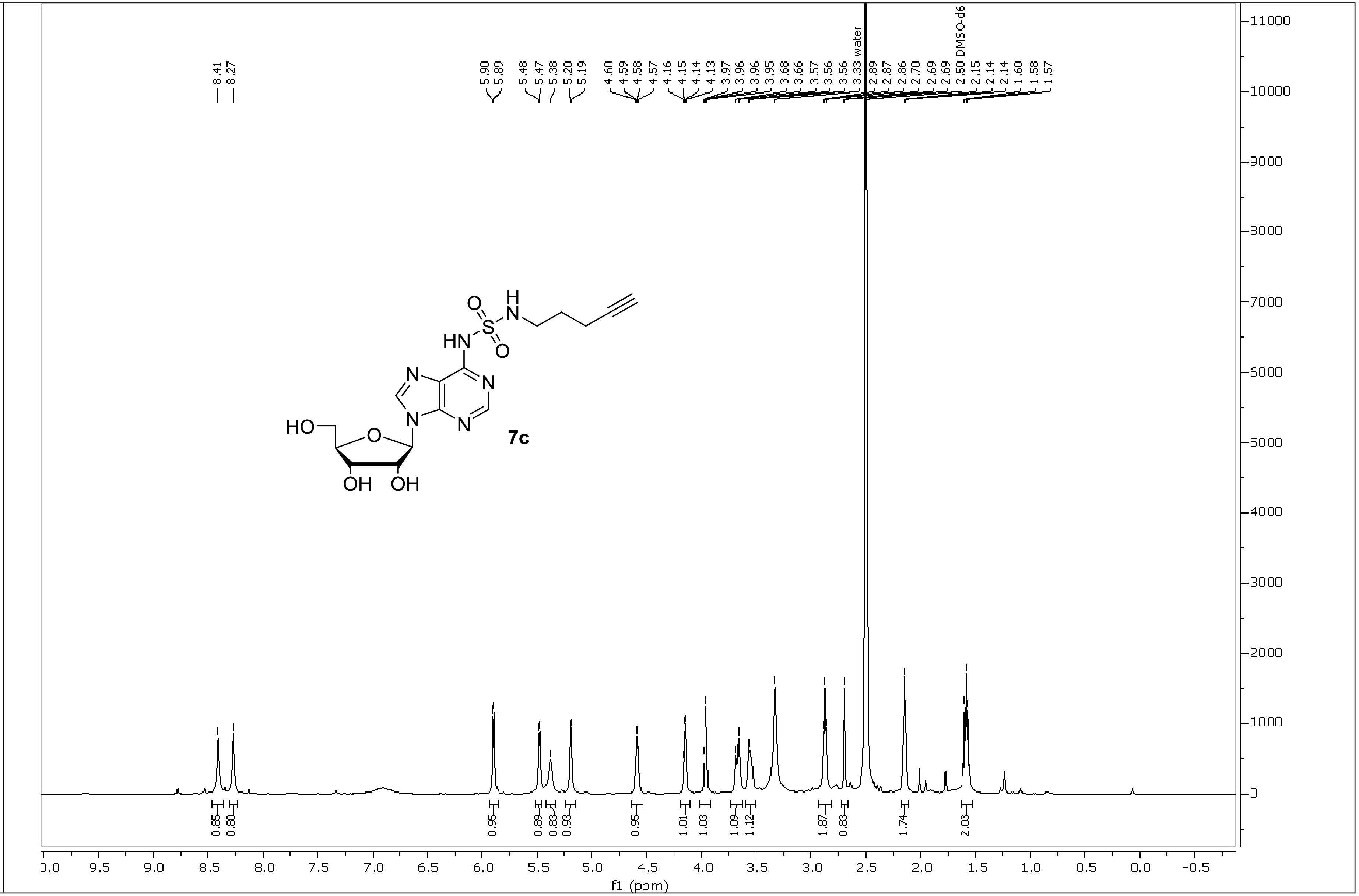

^{13}C NMR spectrum (126 MHz) of **7c**

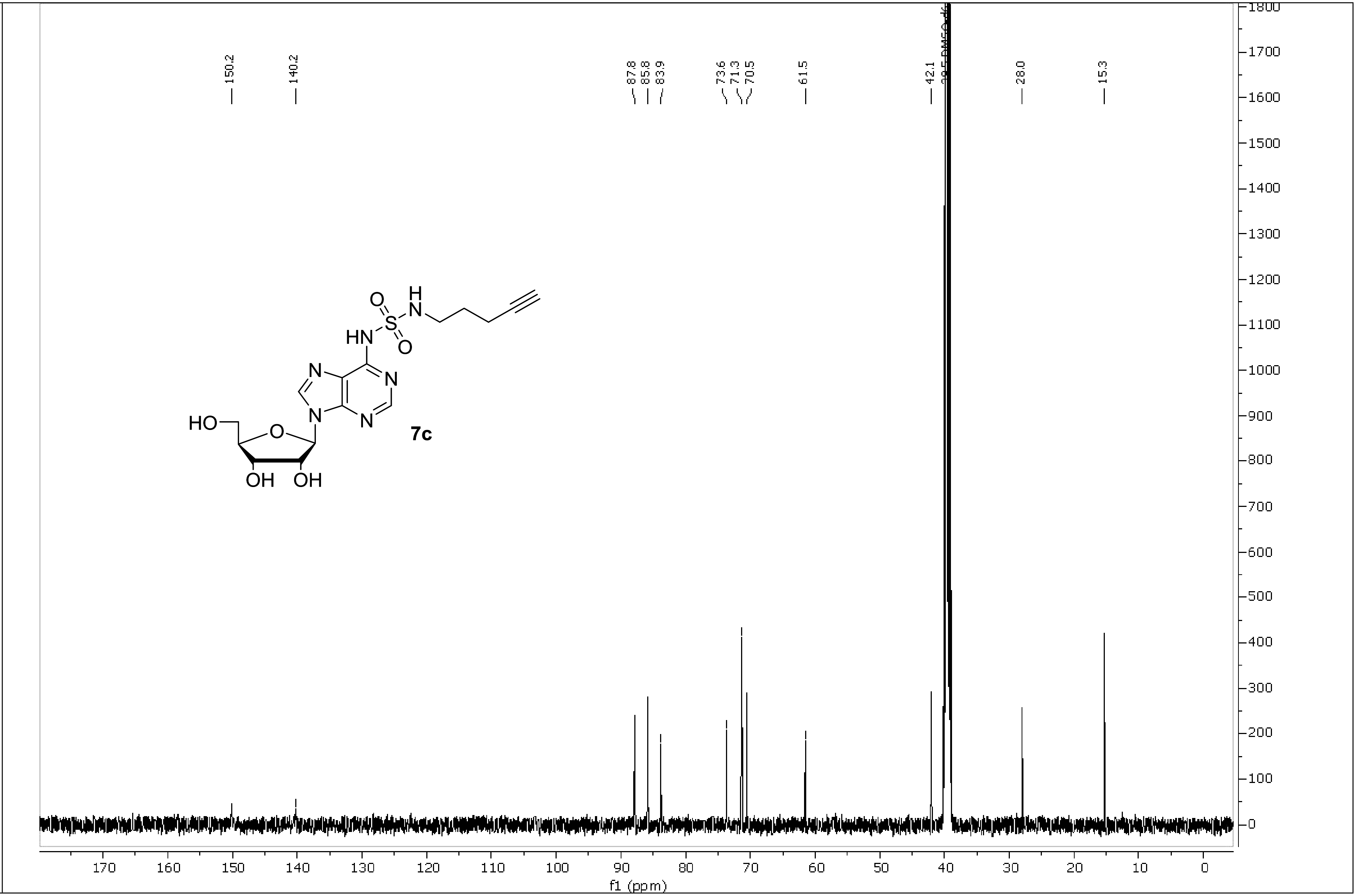

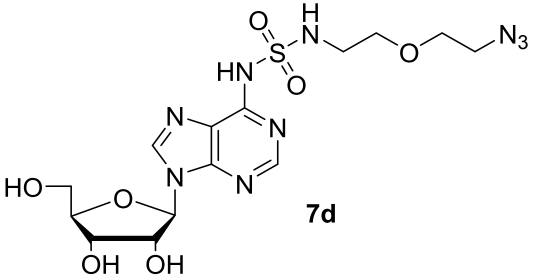
7d

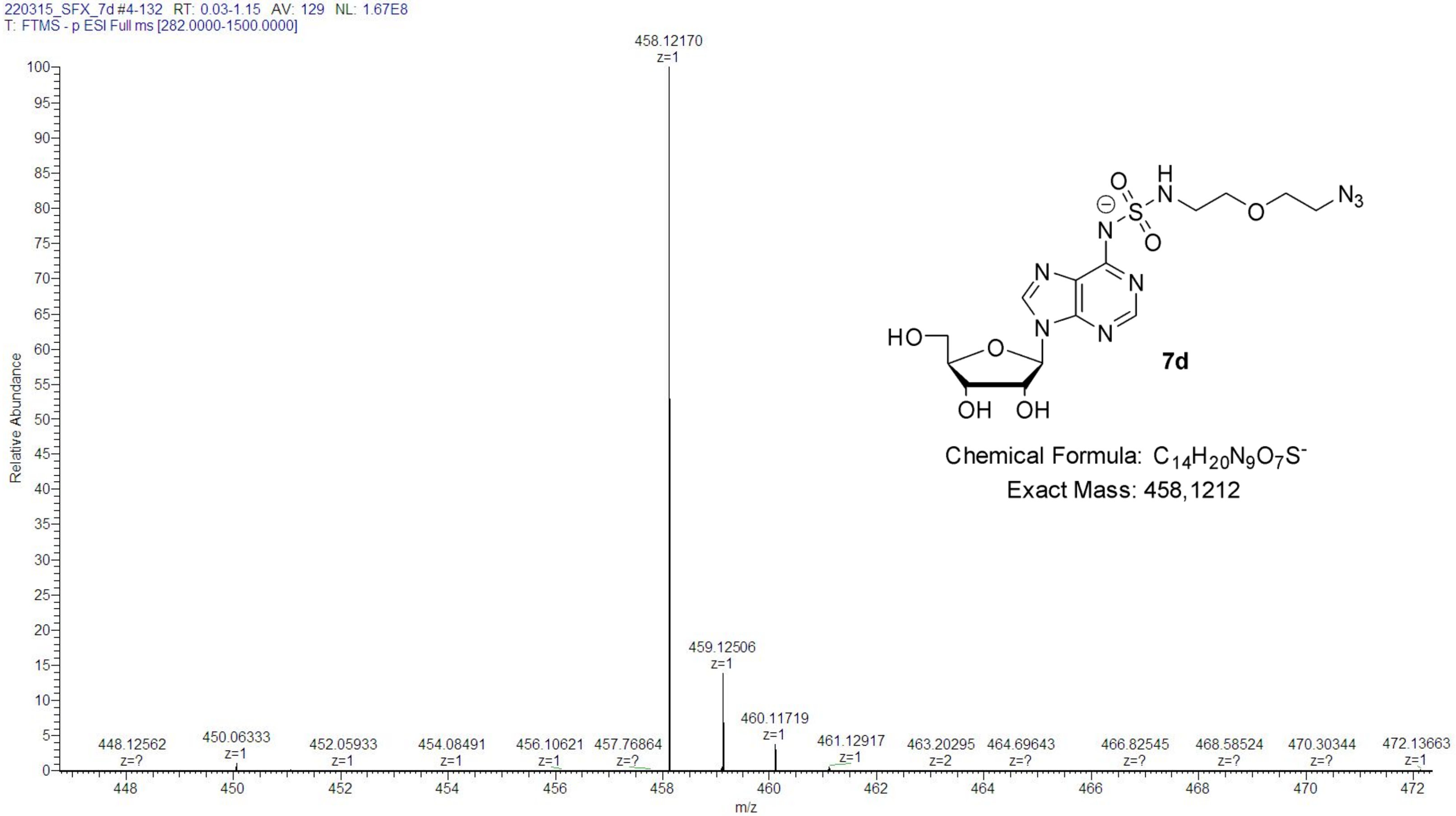
220315_SFX_7d #4-132 RT: 0.03-1.15 AV: 129 NL: 1.67E8
T: FTMS - p ESI Full ms [282.0000-1500.0000]
458.12170
z=1
459.12506
z=1
460.11719
z=1
Relative Abundance
m/z
7d
Chemical Formula: C14H20N9O7S-
Exact Mass: 458,1212

^{1}H NMR spectrum (500 MHz) of **7d**

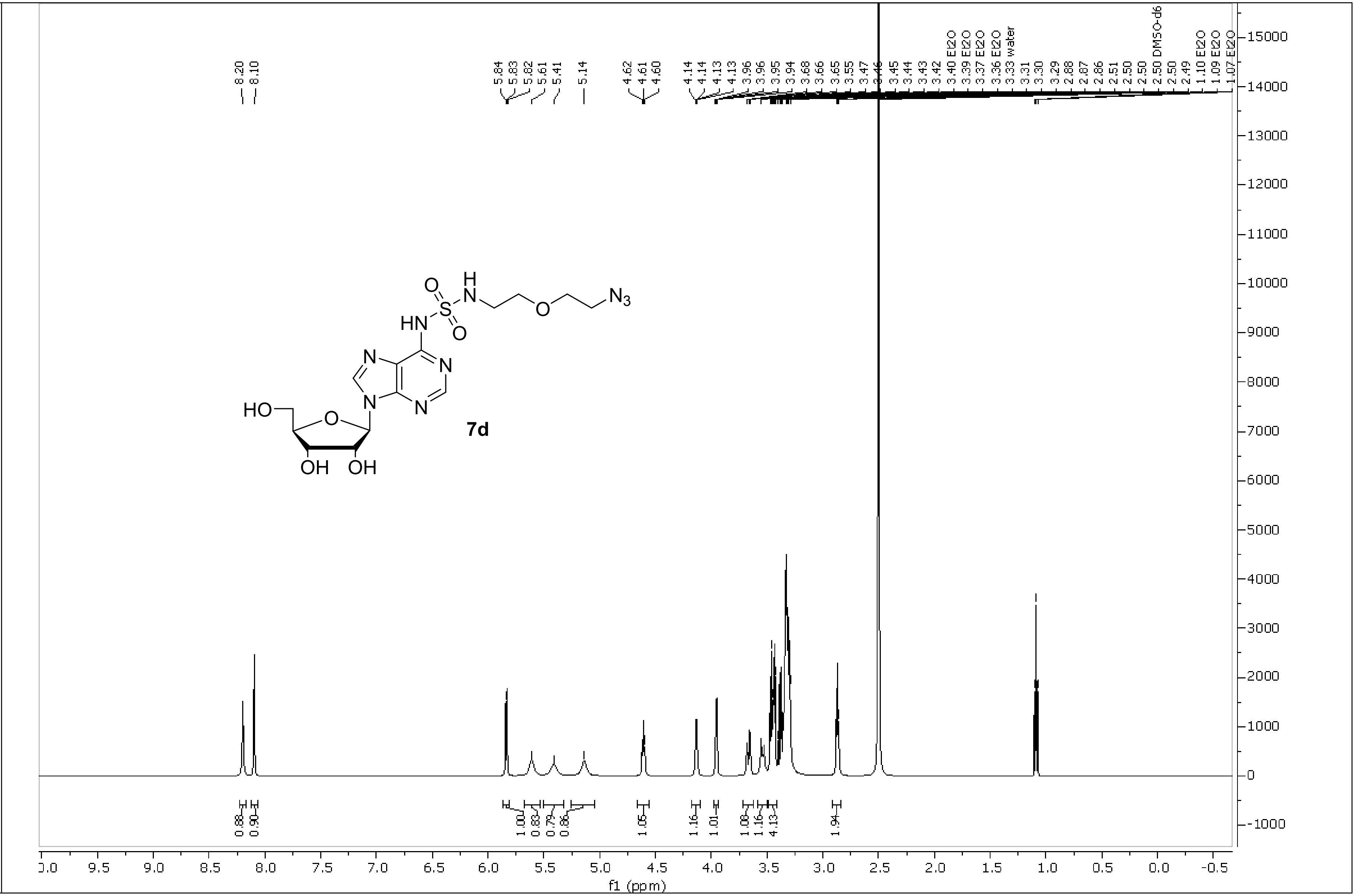

^{13}C NMR spectrum (126 MHz) of **7d**

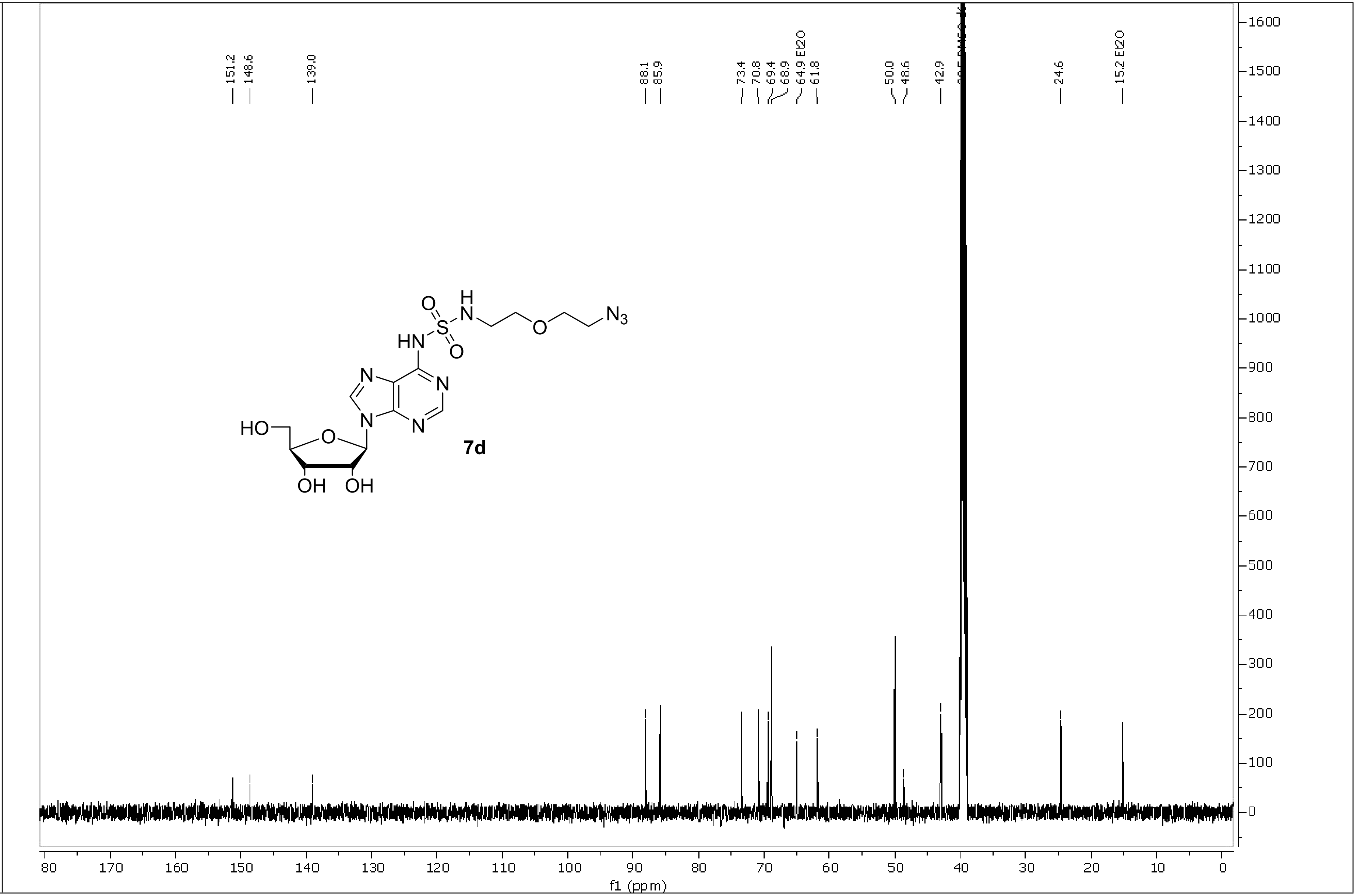

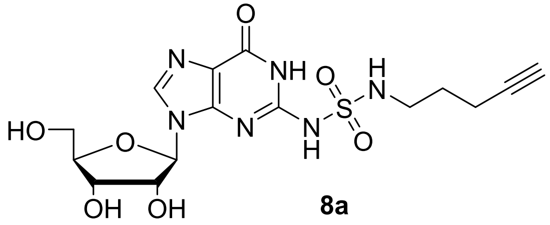

HO
O
N
N
NH
O
N
N
H
S
O
O
H
N
OH
OH

8a

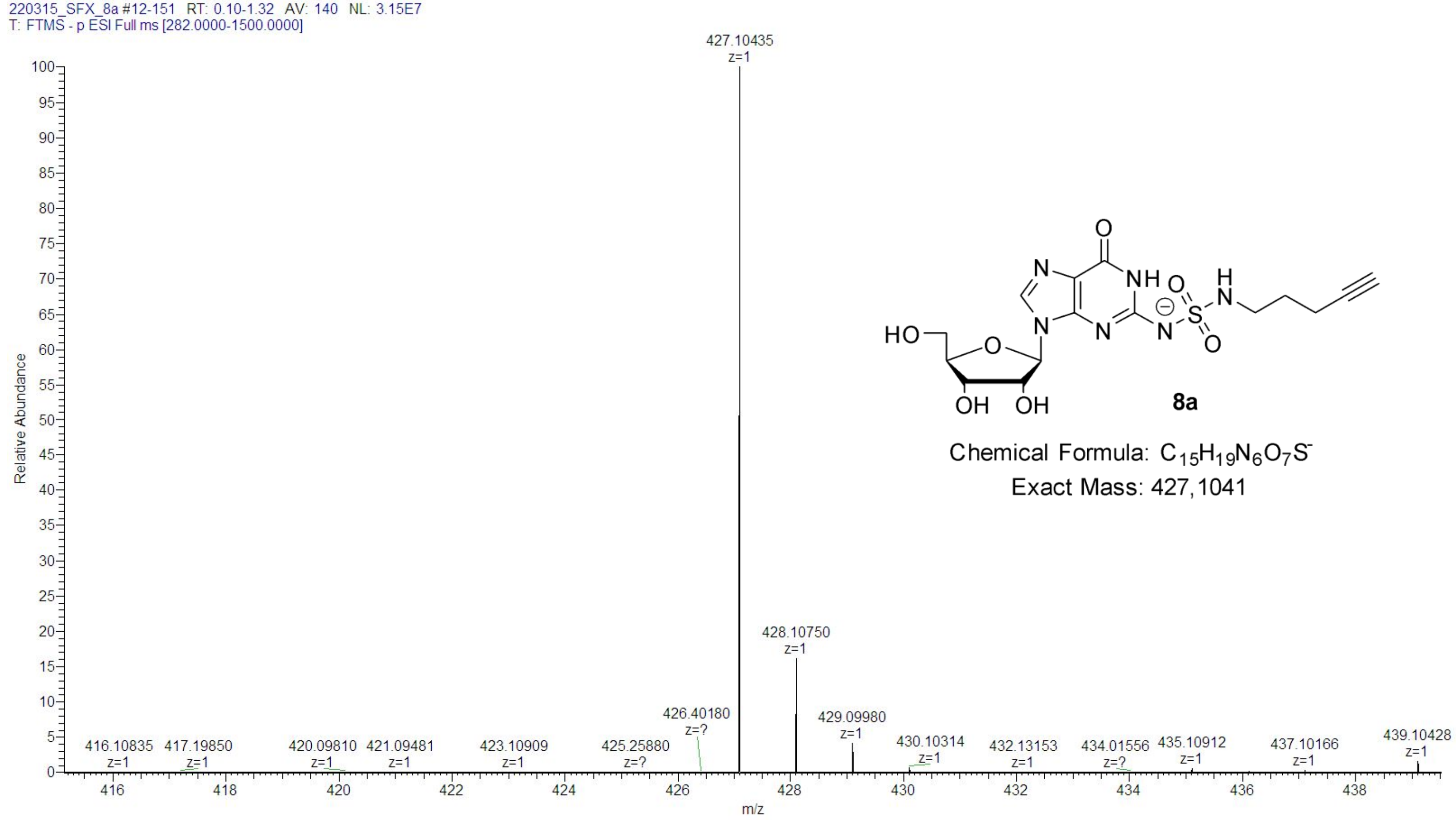

220315_SFX_8a #12-151 RT: 0.10-1.32 AV: 140 NL: 3.15E7
T: FTMS - p ESI Full ms [282.0000-1500.0000]
427.10435
z=1
428.10750
z=1
429.09980
z=1
426.40180
z=?
416.10835
417.19850
420.09810
421.09481
423.10909
425.25880
430.10314
432.13153
434.01556
435.10912
437.10166
439.10428
Relative Abundance
m/z
8a
Chemical Formula: $C_{15}H_{19}N_6O_7S^-$
Exact Mass: 427,1041

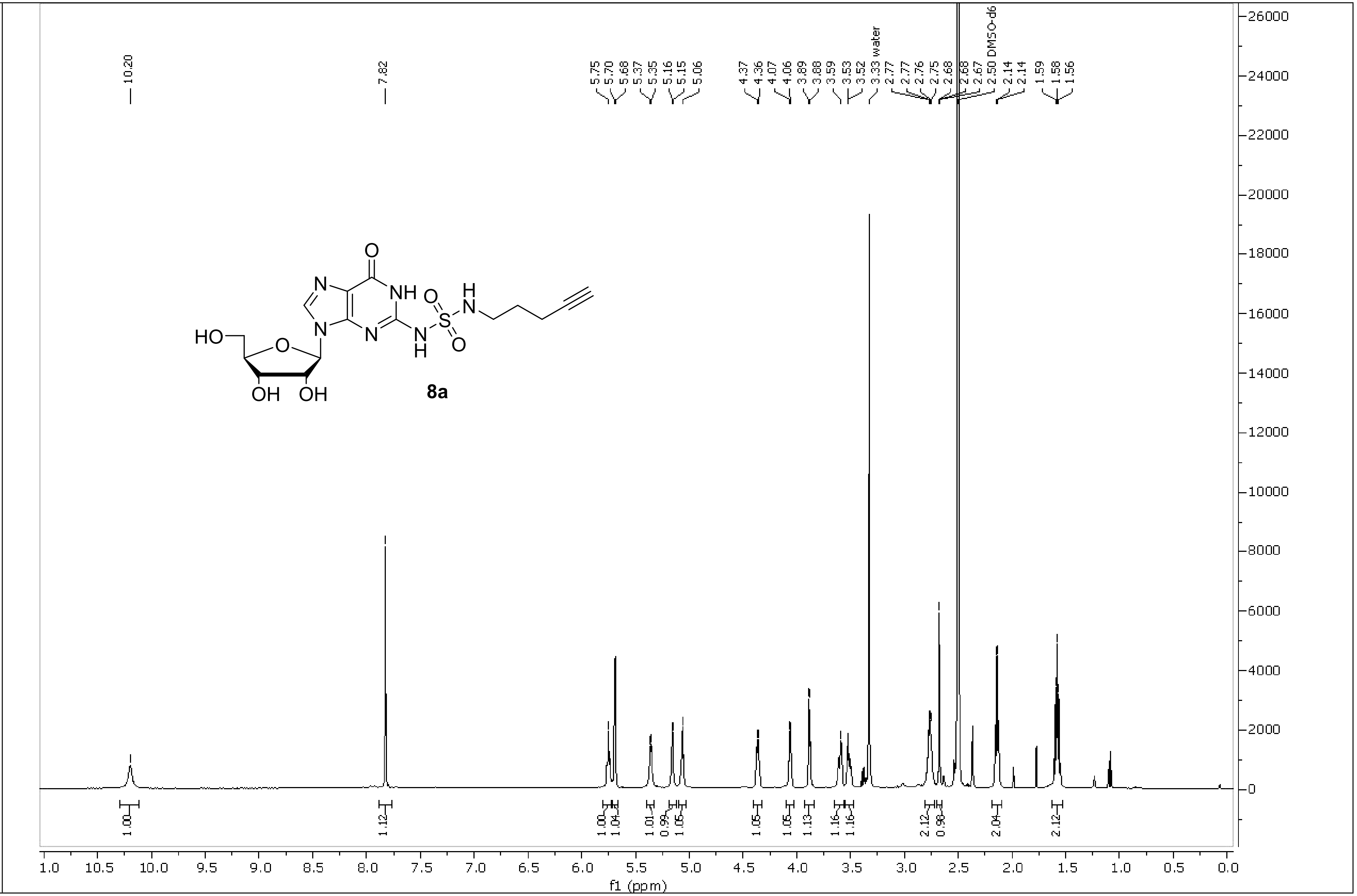

^{1}H NMR spectrum (500 MHz) of **8a**

^{13}C NMR spectrum (126 MHz) of **8a**

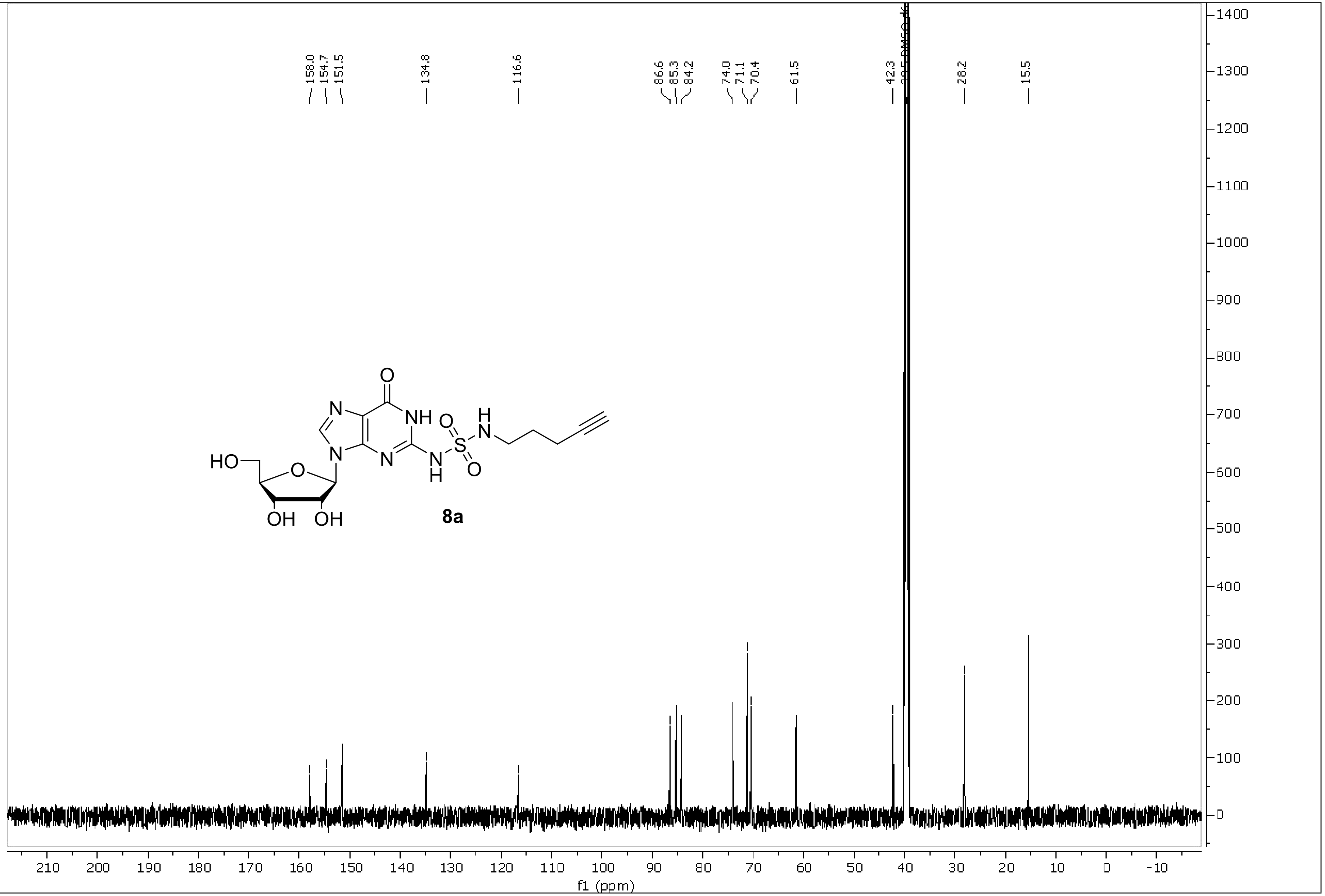

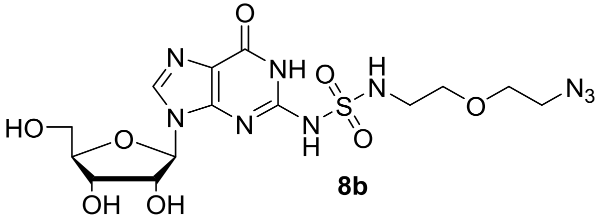

8b

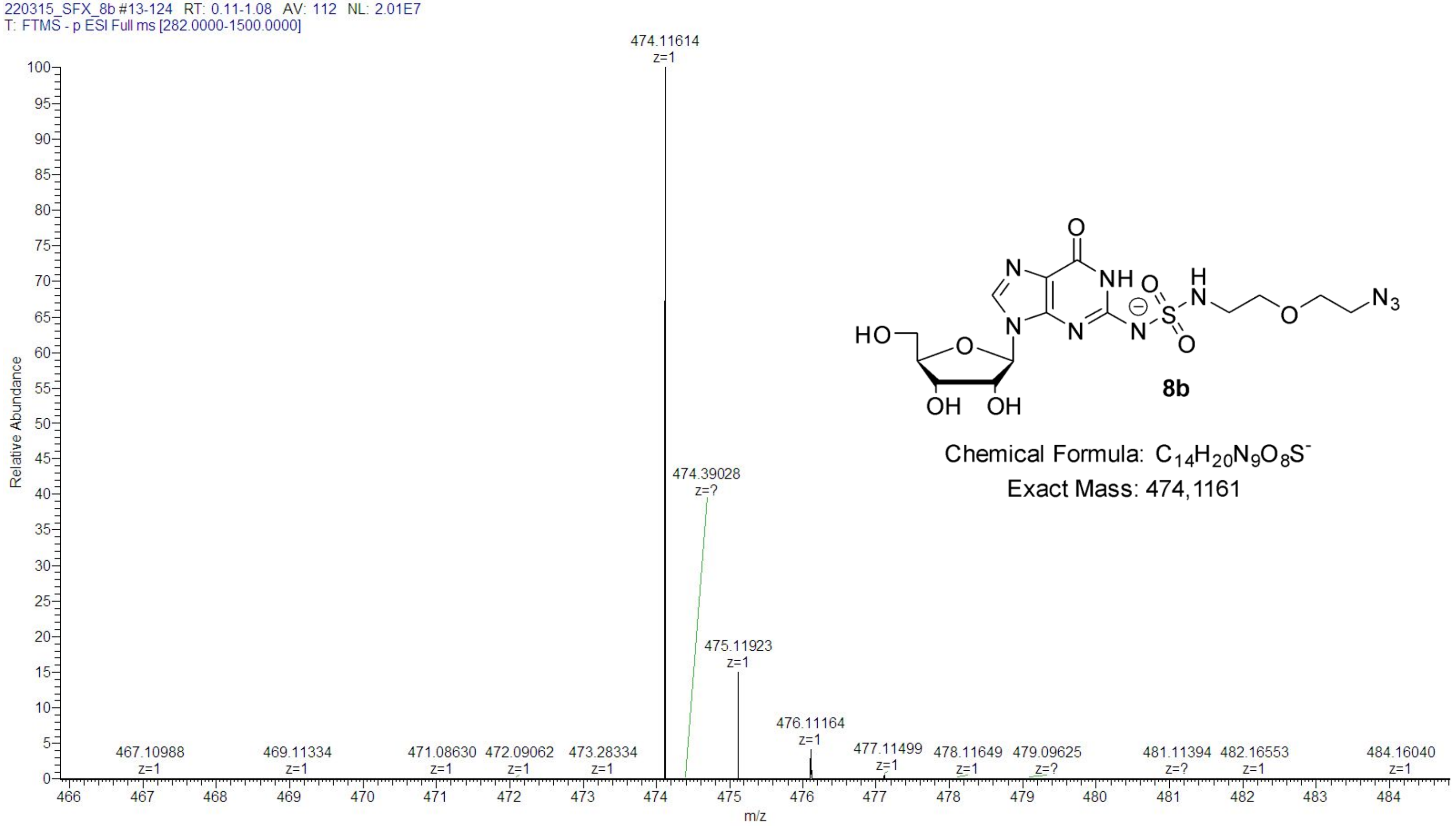

220315_SFX_8b #13-124 RT: 0.11-1.08 AV: 112 NL: 2.01E7
T: FTMS - p ESI Full ms [282.0000-1500.0000]
474.11614 z=1
474.39028 z=?
475.11923 z=1
476.11164 z=1
467.10988 z=1
469.11334 z=1
471.08630 z=1
472.09062 z=1
473.28334 z=1
477.11499 z=1
478.11649 z=1
479.09625 z=?
481.11394 z=?
482.16553 z=1
484.16040 z=1
Relative Abundance
m/z
8b
Chemical Formula: C14H20N9O8S-
Exact Mass: 474,1161

^1H NMR spectrum (500 MHz) of **8b**

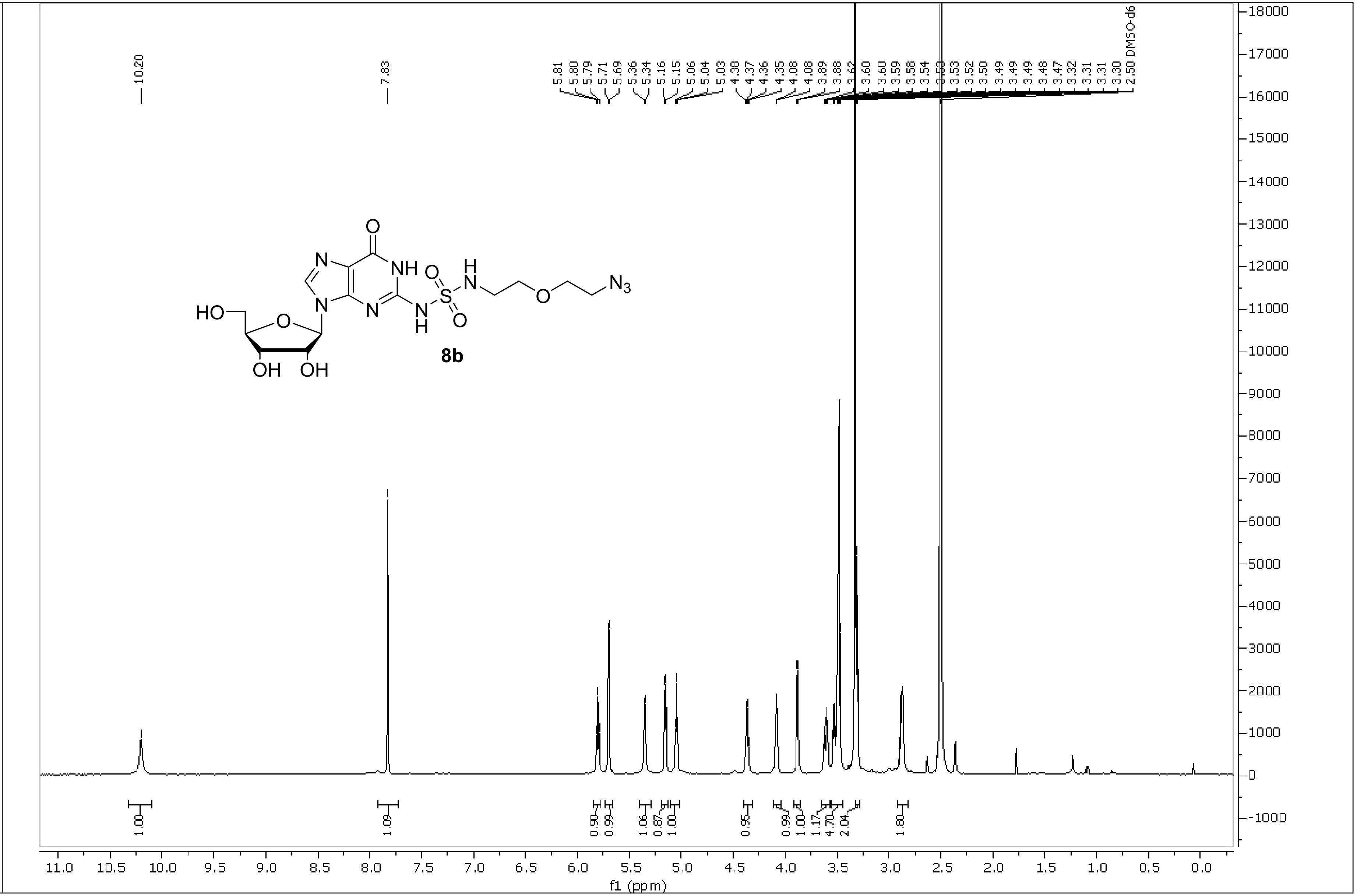

^{13}C NMR spectrum (126 MHz) of **8b**

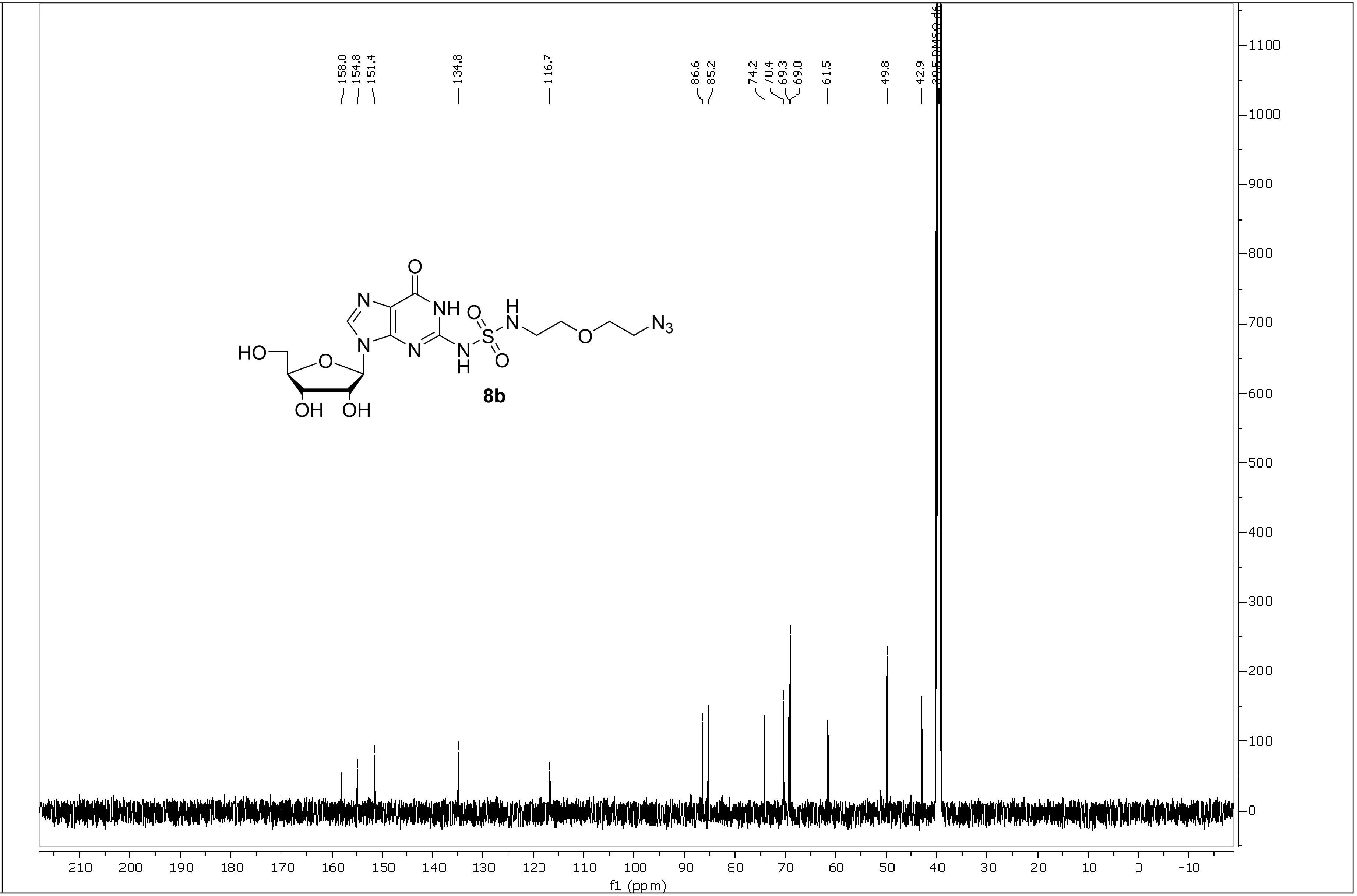

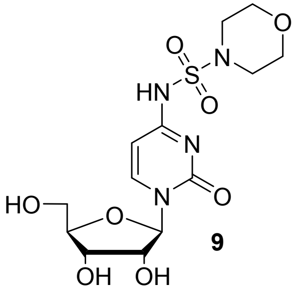

9

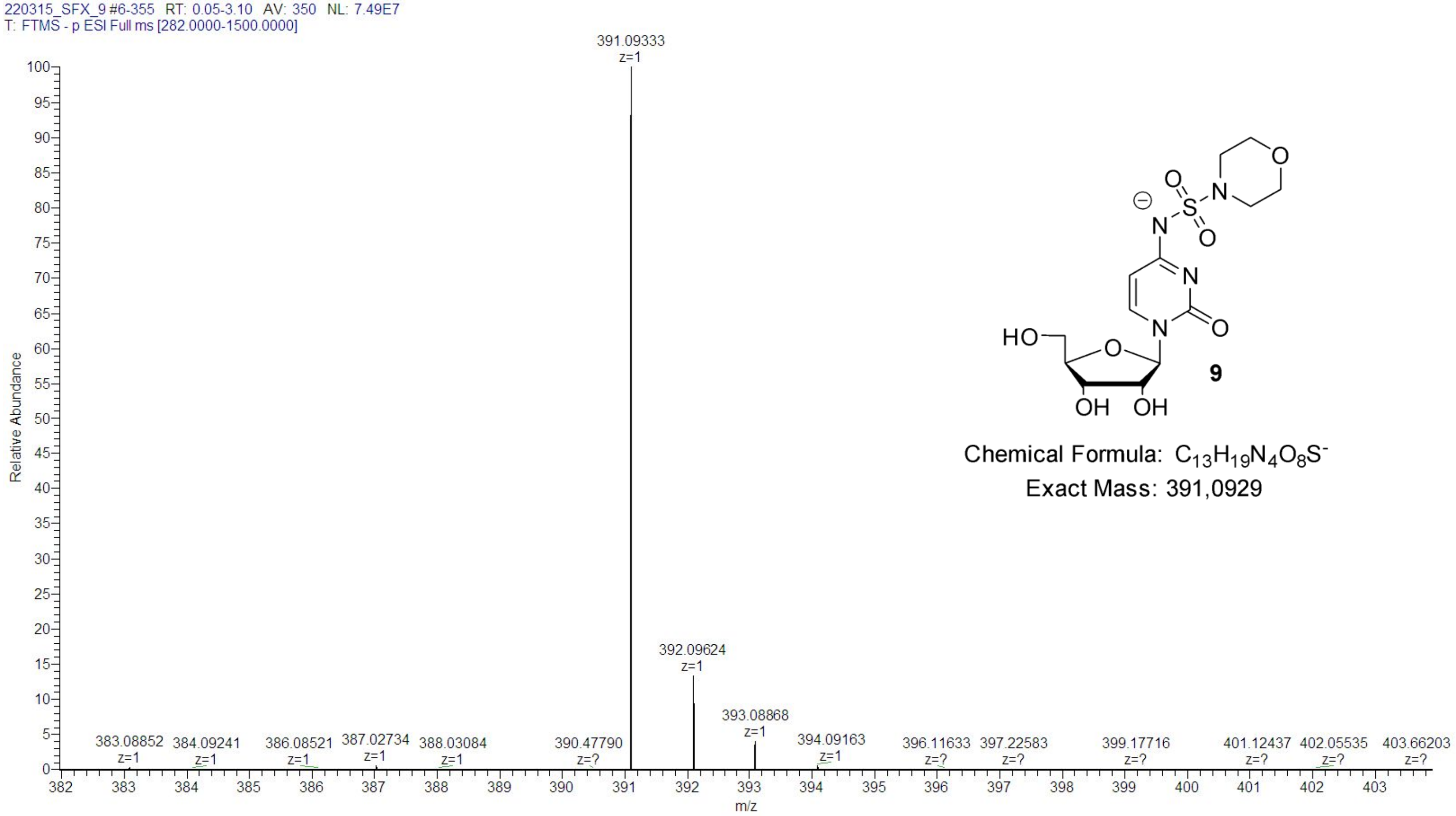

220315_SFX_9 #6-355 RT: 0.05-3.10 AV: 350 NL: 7.49E7
T: FTMS - p ESI Full ms [282.0000-1500.0000]
391.09333 z=1
392.09624 z=1
393.08868 z=1
394.09163 z=1
383.08852 z=1
384.09241 z=1
386.08521 z=1
387.02734 z=1
388.03084 z=1
390.47790 z=?
396.11633 z=?
397.22583 z=?
399.17716 z=?
401.12437 z=?
402.05535 z=?
403.66203 z=?
Relative Abundance
m/z
9
Chemical Formula: $C_{13}H_{19}N_4O_8S^-$
Exact Mass: 391,0929

^{1}H NMR spectrum (500 MHz) of **9**

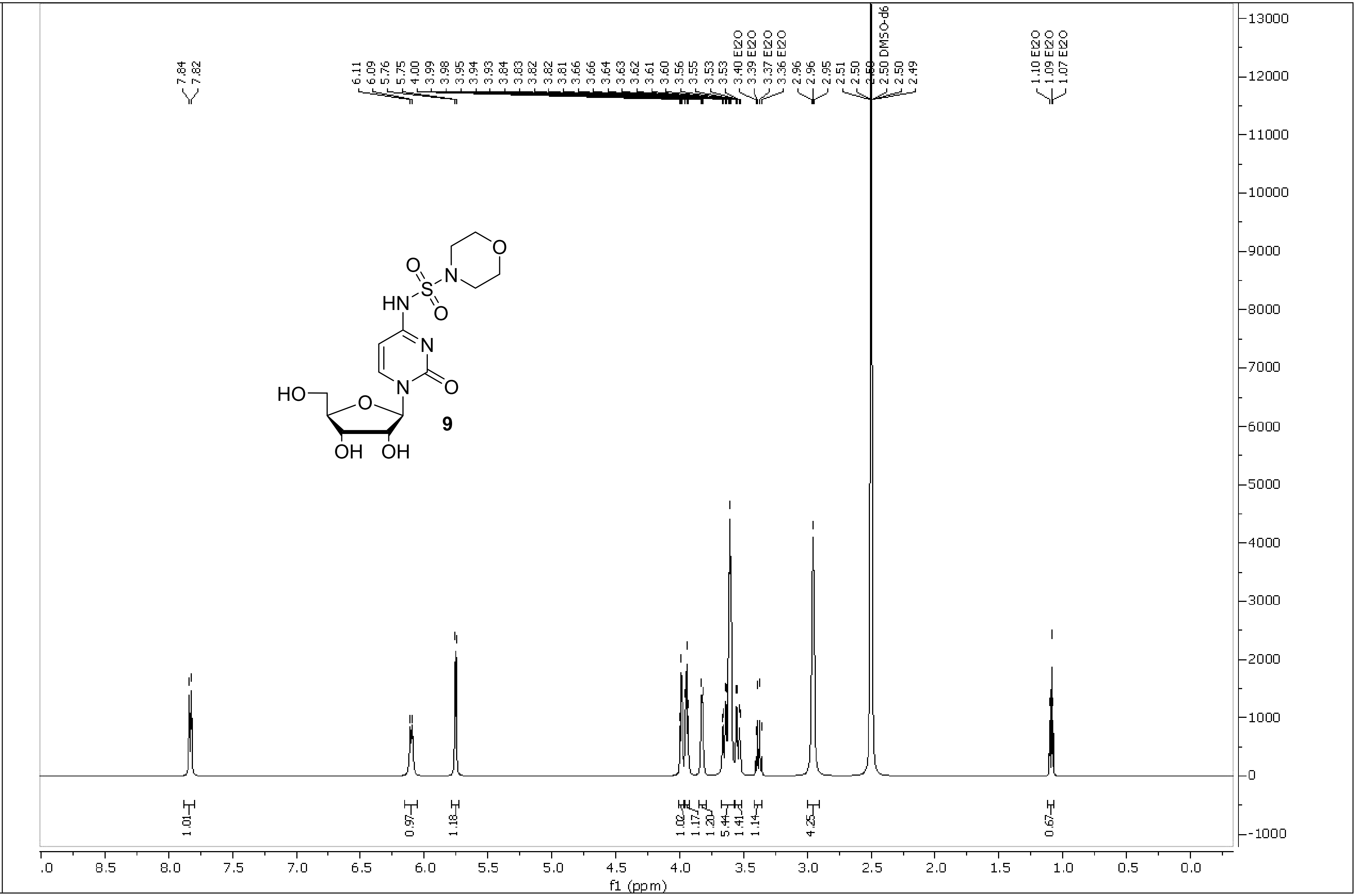

^{13}C NMR spectrum (126 MHz) of **9**

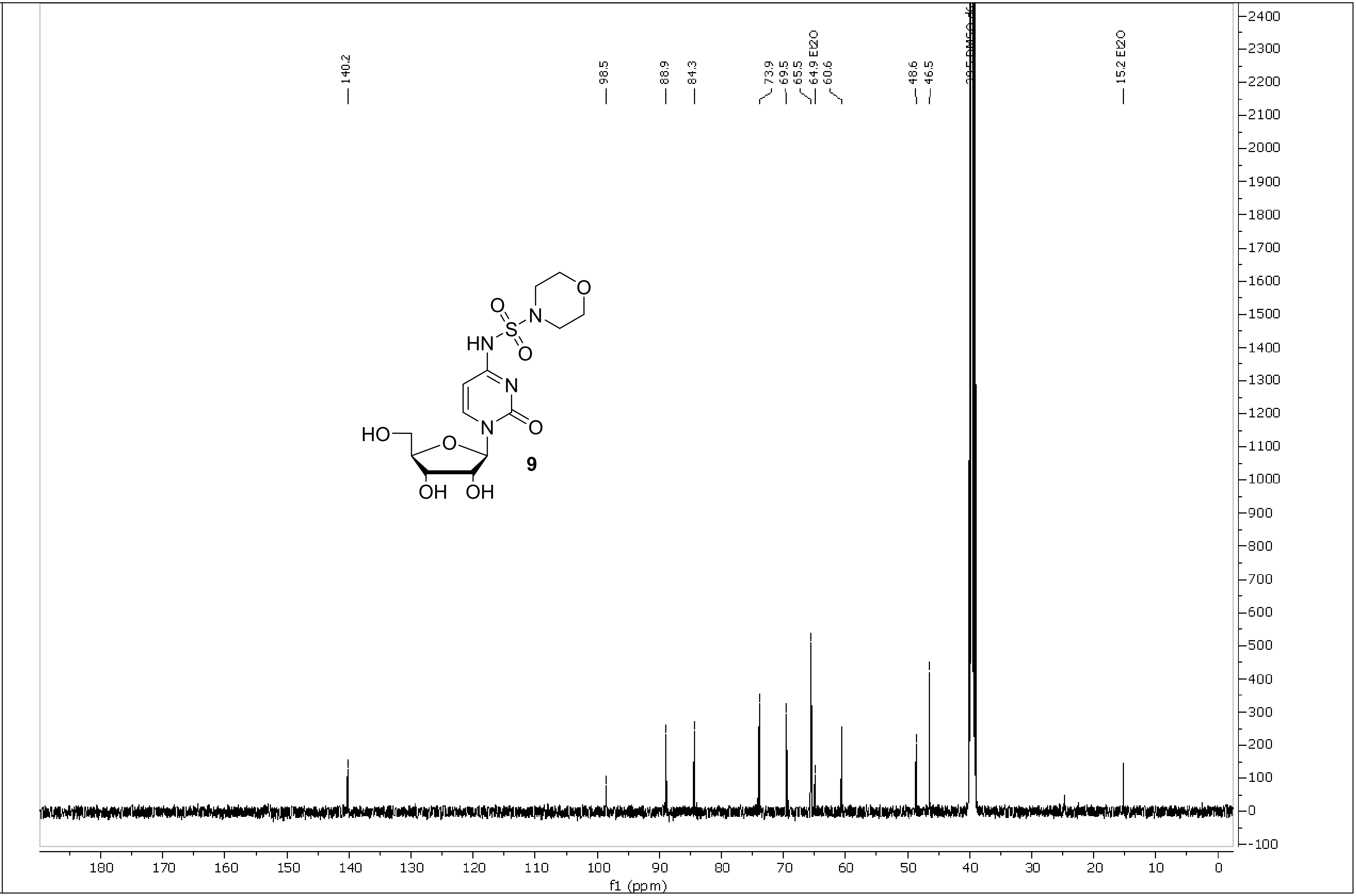

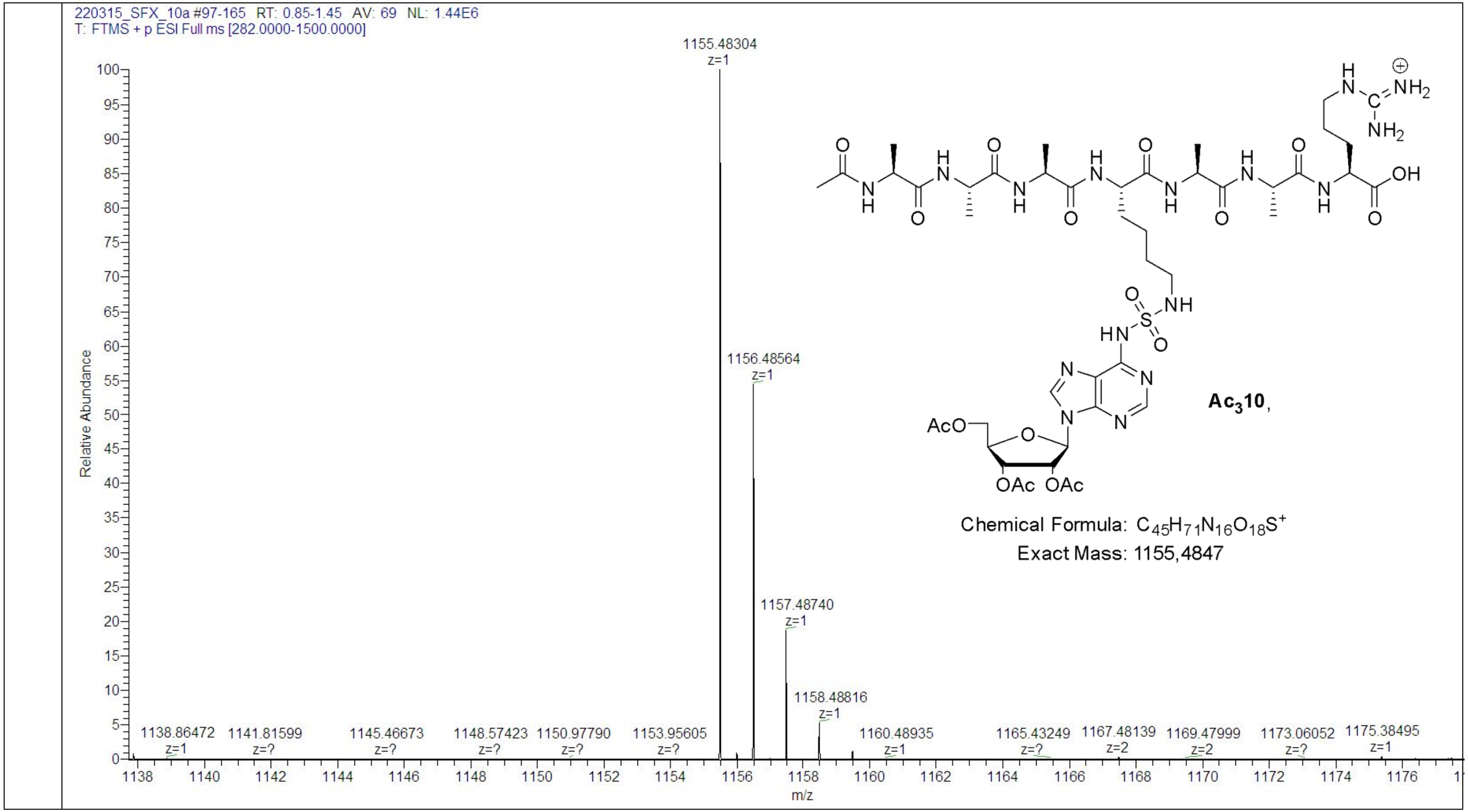

Ac$_3$10,

Chemical Formula: $C_{45}H_{71}N_{16}O_{18}S^{+}$

Exact Mass: 1155,4847

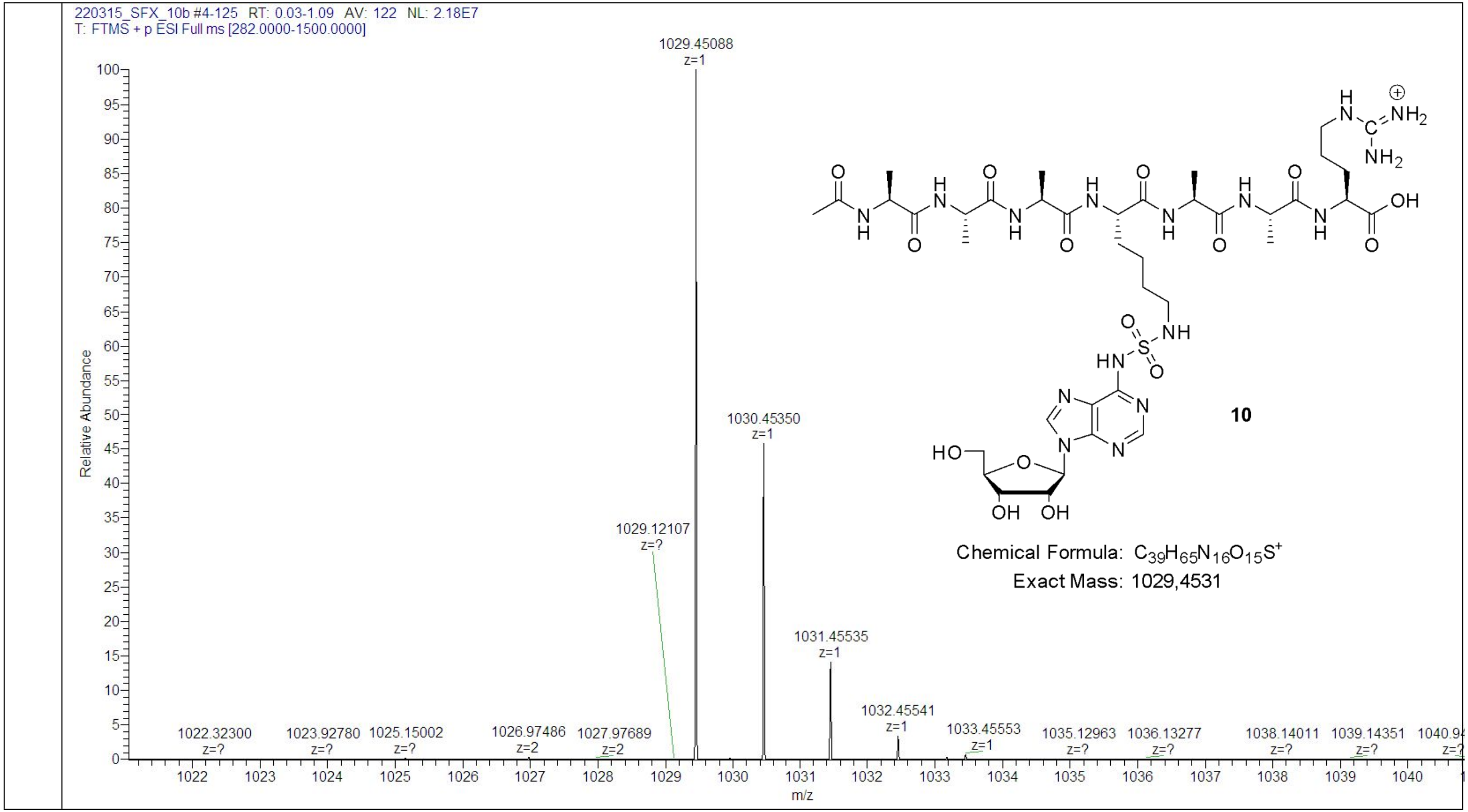
220315_SFX_10b #4-125 RT: 0.03-1.09 AV: 122 NL: 2.18E7
T: FTMS + p ESI Full ms [282.0000-1500.0000]
1029.45088 z=1
1030.45350 z=1
1029.12107 z=?
1031.45535 z=1
1032.45541 z=1
1033.45553 z=1
1022.32300 z=?
1023.92780 z=?
1025.15002 z=?
1026.97486 z=2
1027.97689 z=2
1035.12963 z=?
1036.13277 z=?
1038.14011 z=?
1039.14351 z=?
Relative Abundance
m/z
10
Chemical Formula: C39H65N16O15S+
Exact Mass: 1029,4531

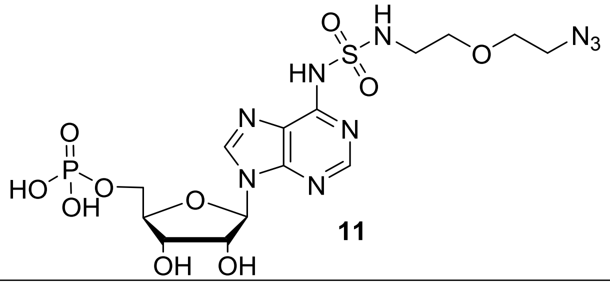

11

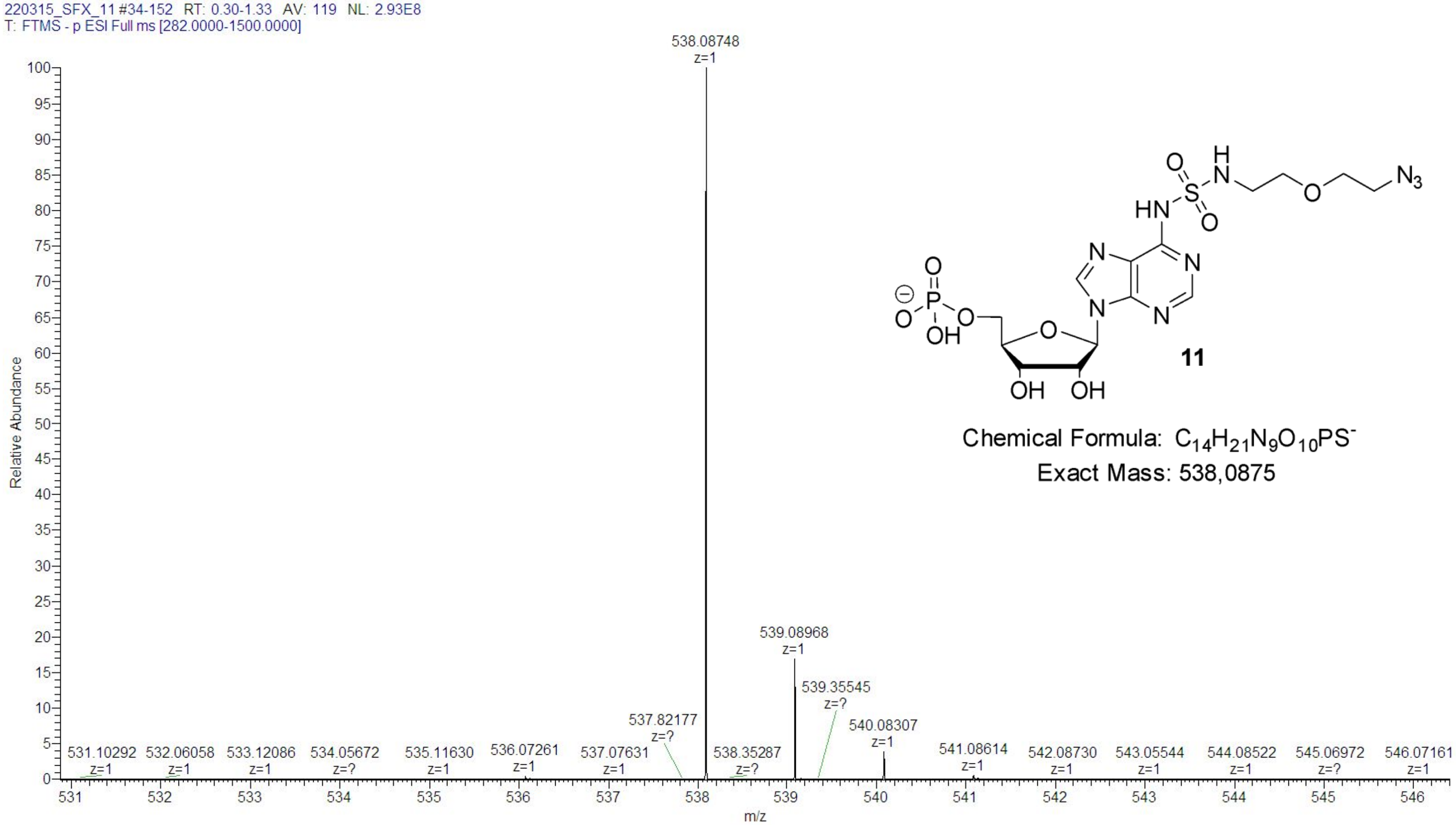

220315_SFX_11 #34-152 RT: 0.30-1.33 AV: 119 NL: 2.93E8
T: FTMS - p ESI Full ms [282.0000-1500.0000]
538.08748 z=1
539.08968 z=1
539.35545 z=?
540.08307 z=1
537.82177 z=?
531.10292 z=1
532.06058 z=1
533.12086 z=1
534.05672 z=?
535.11630 z=1
536.07261 z=1
537.07631 z=1
538.35287 z=?
541.08614 z=1
542.08730 z=1
543.05544 z=1
544.08522 z=1
545.06972 z=?
546.07161 z=1
Relative Abundance
m/z
11
Chemical Formula: C14H21N9O10PS-
Exact Mass: 538,0875

^{1}H NMR spectrum (500 MHz) of **11**

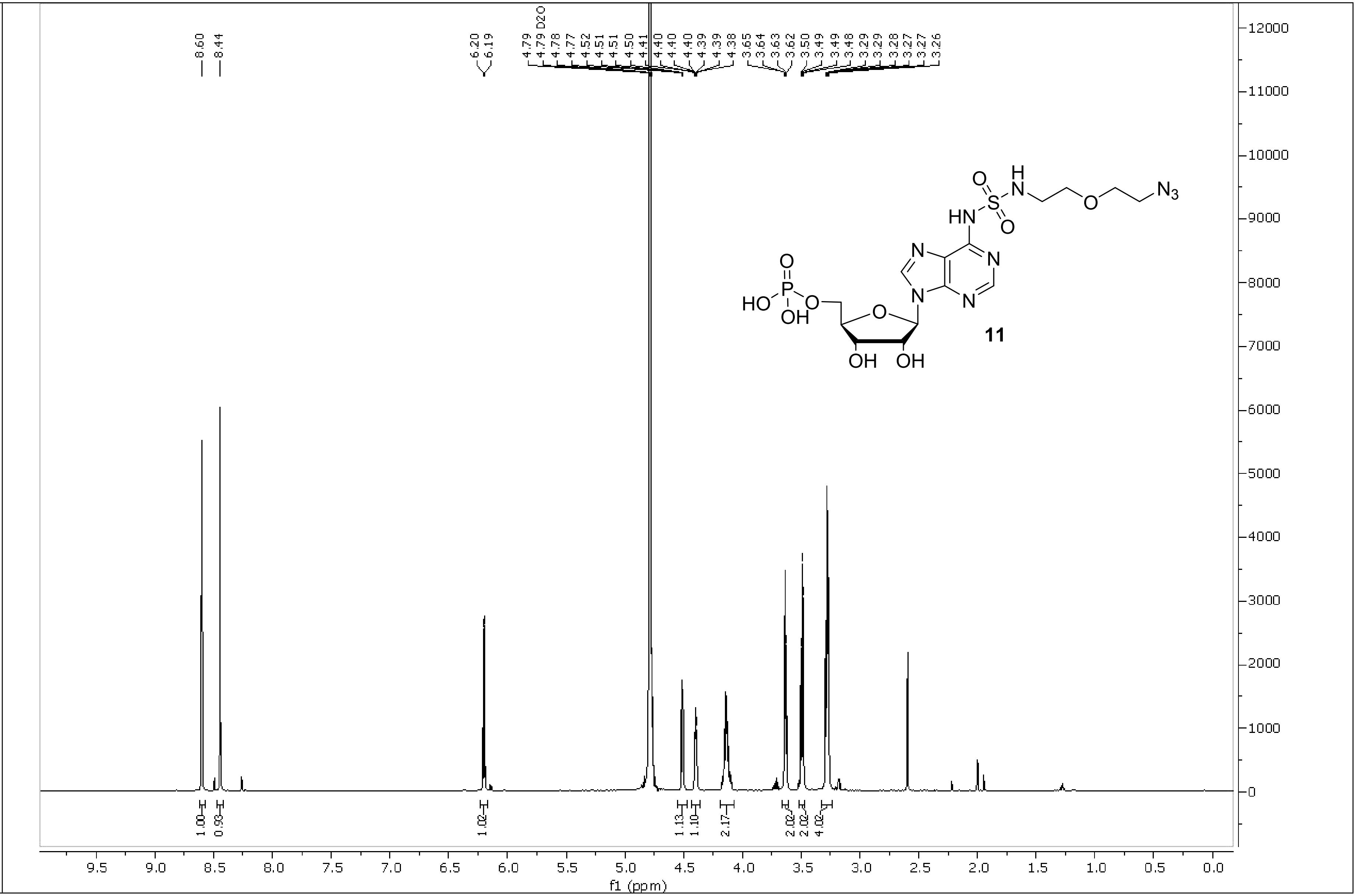

^{13}C NMR spectrum (126 MHz) of **11**

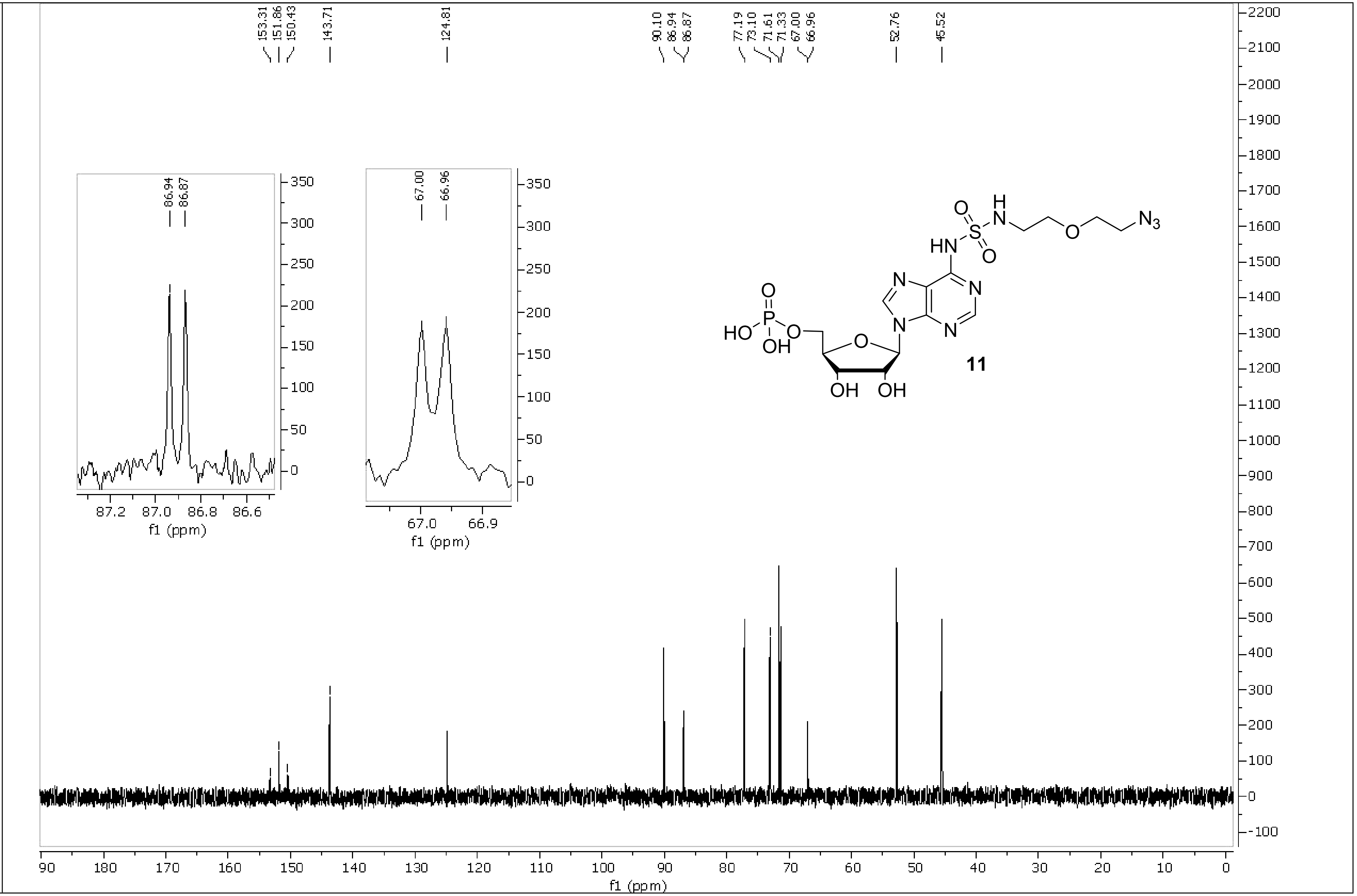

COSY NMR spectrum of **11**

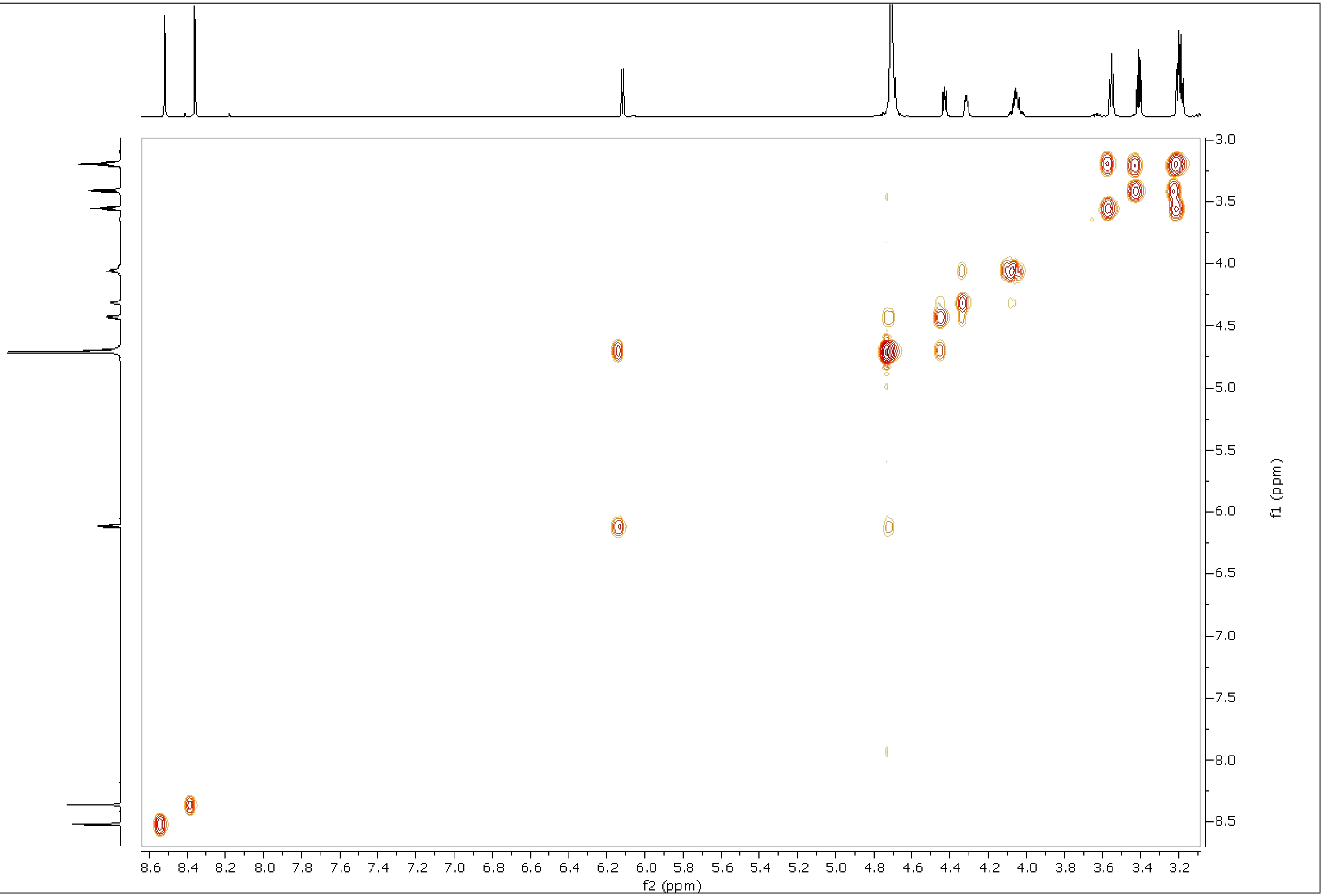

^{31}P NMR spectrum (202 MHz) of **11**

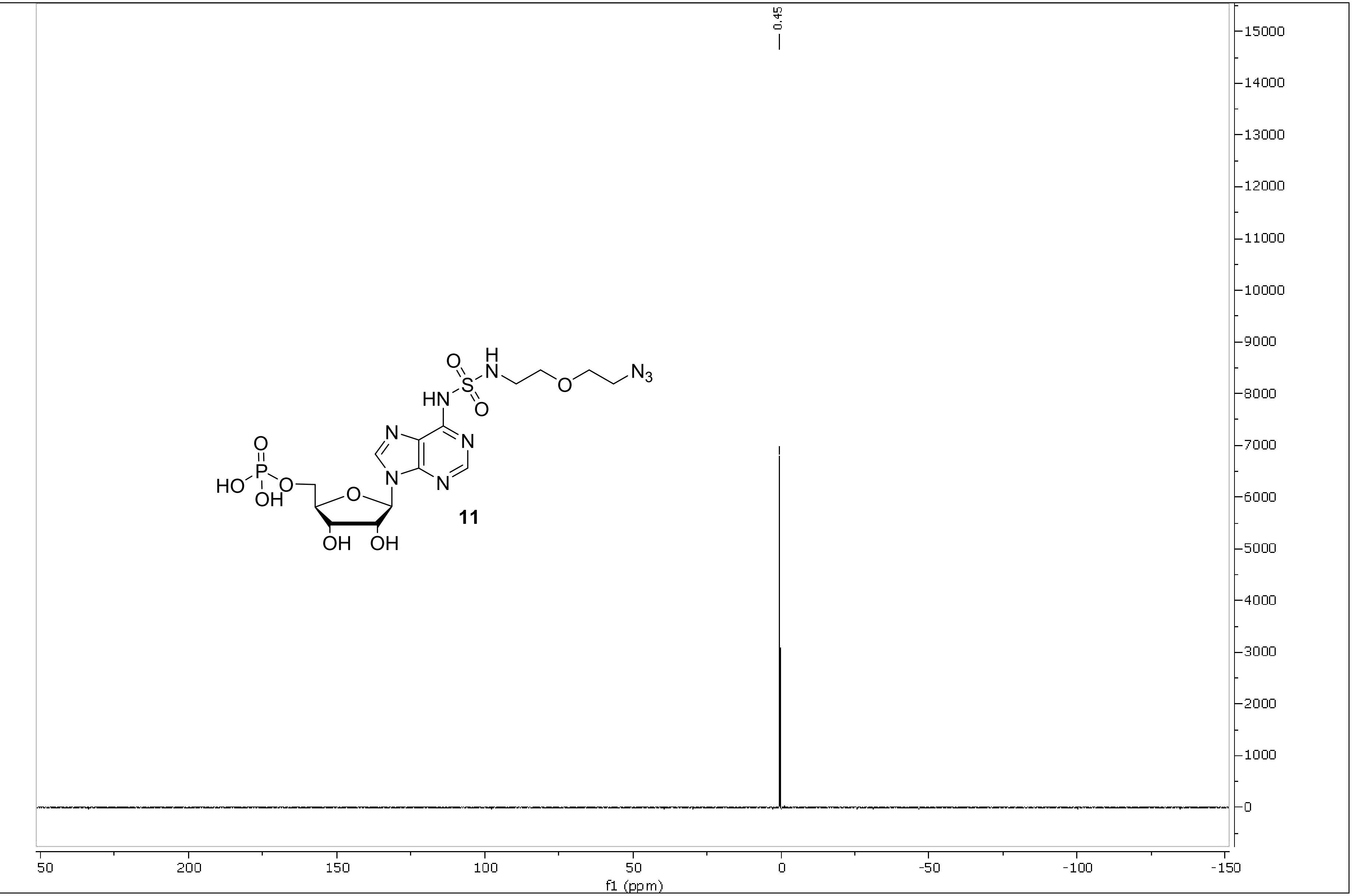

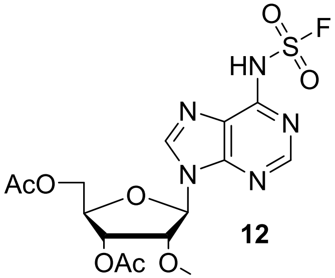

12

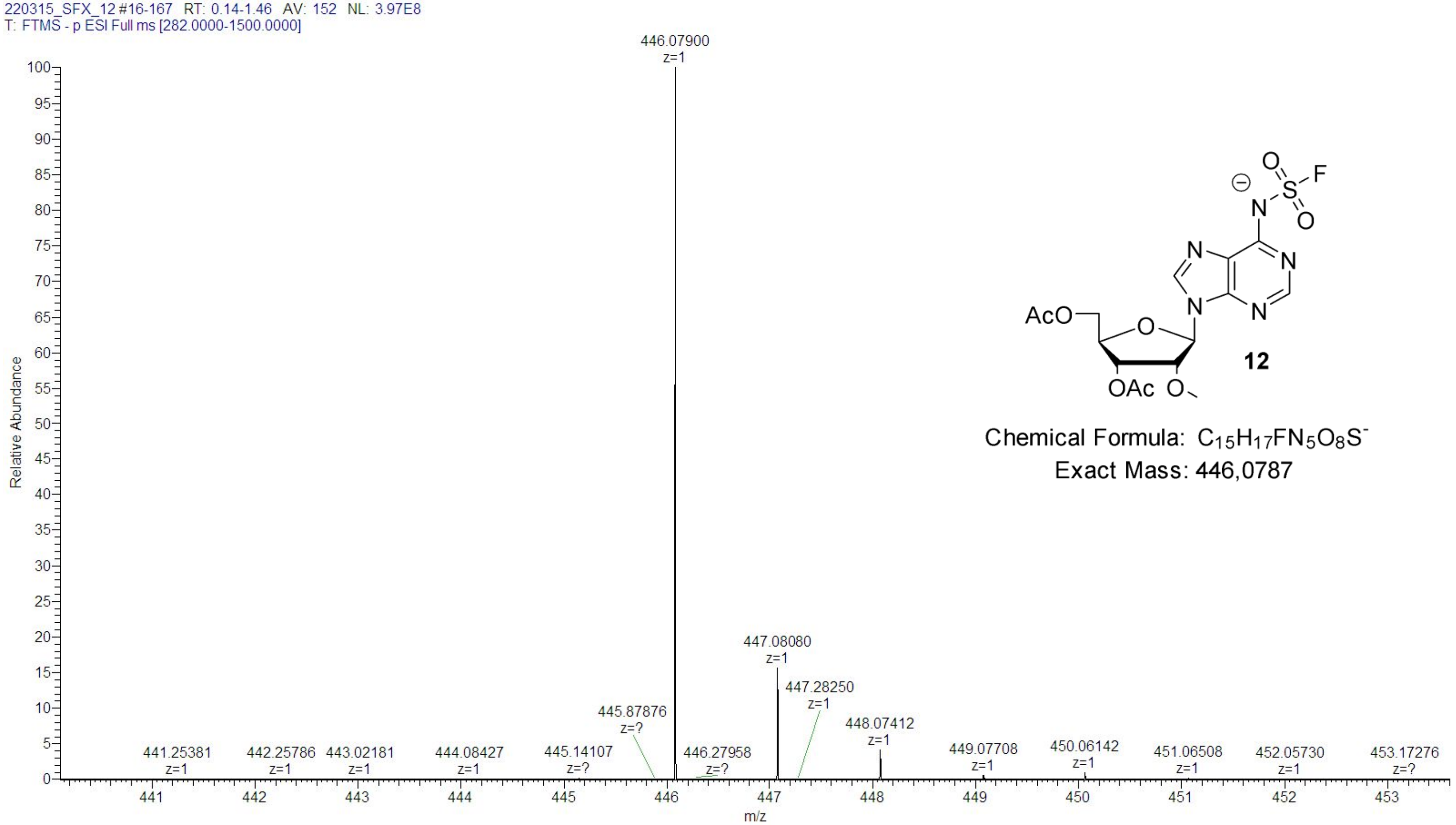

12

Chemical Formula: $C_{15}H_{17}FN_5O_8S^-$
Exact Mass: 446,0787

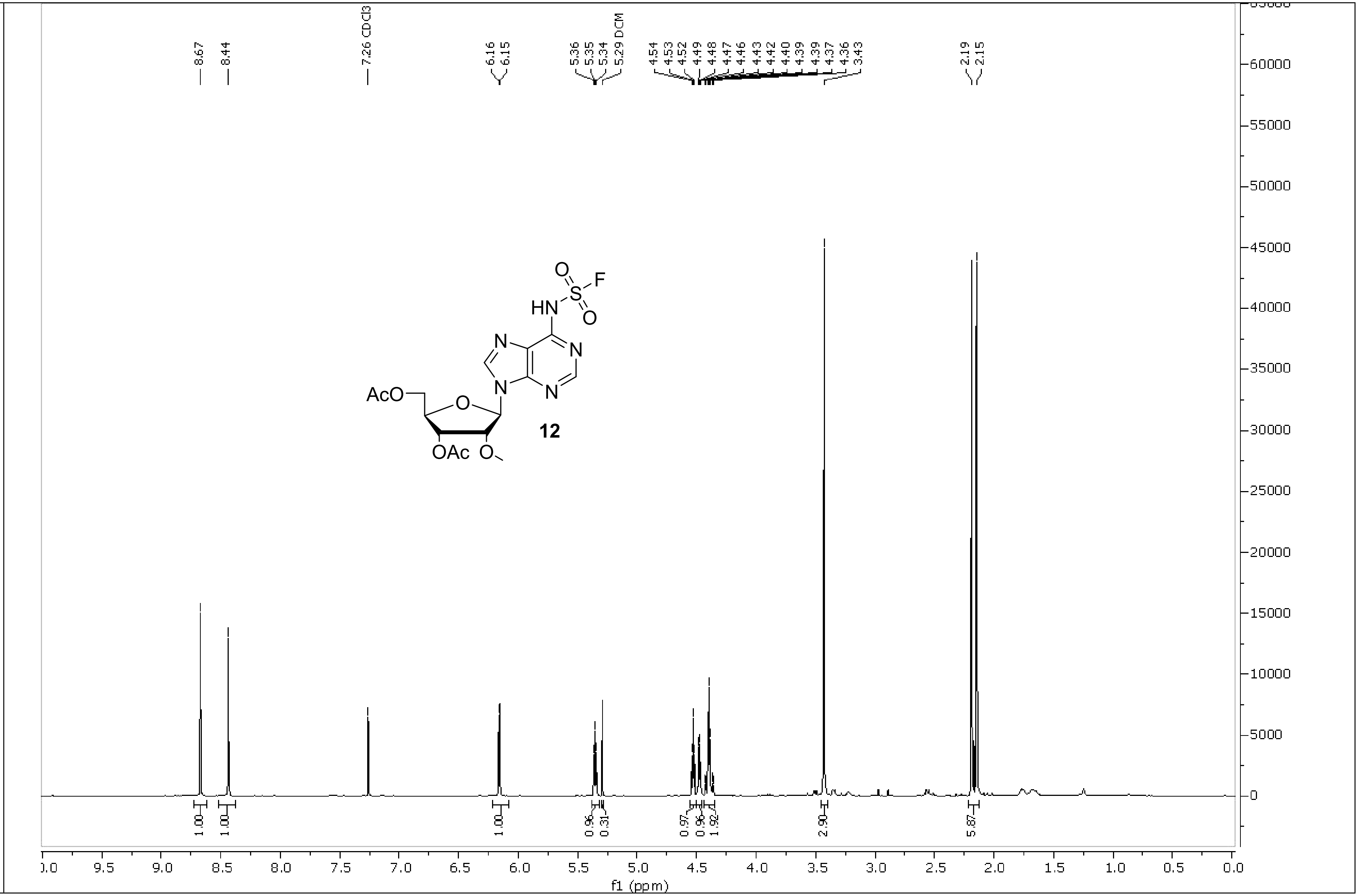

^{1}H NMR spectrum (500 MHz) of **12**

^{13}C NMR spectrum (126 MHz) of **12**

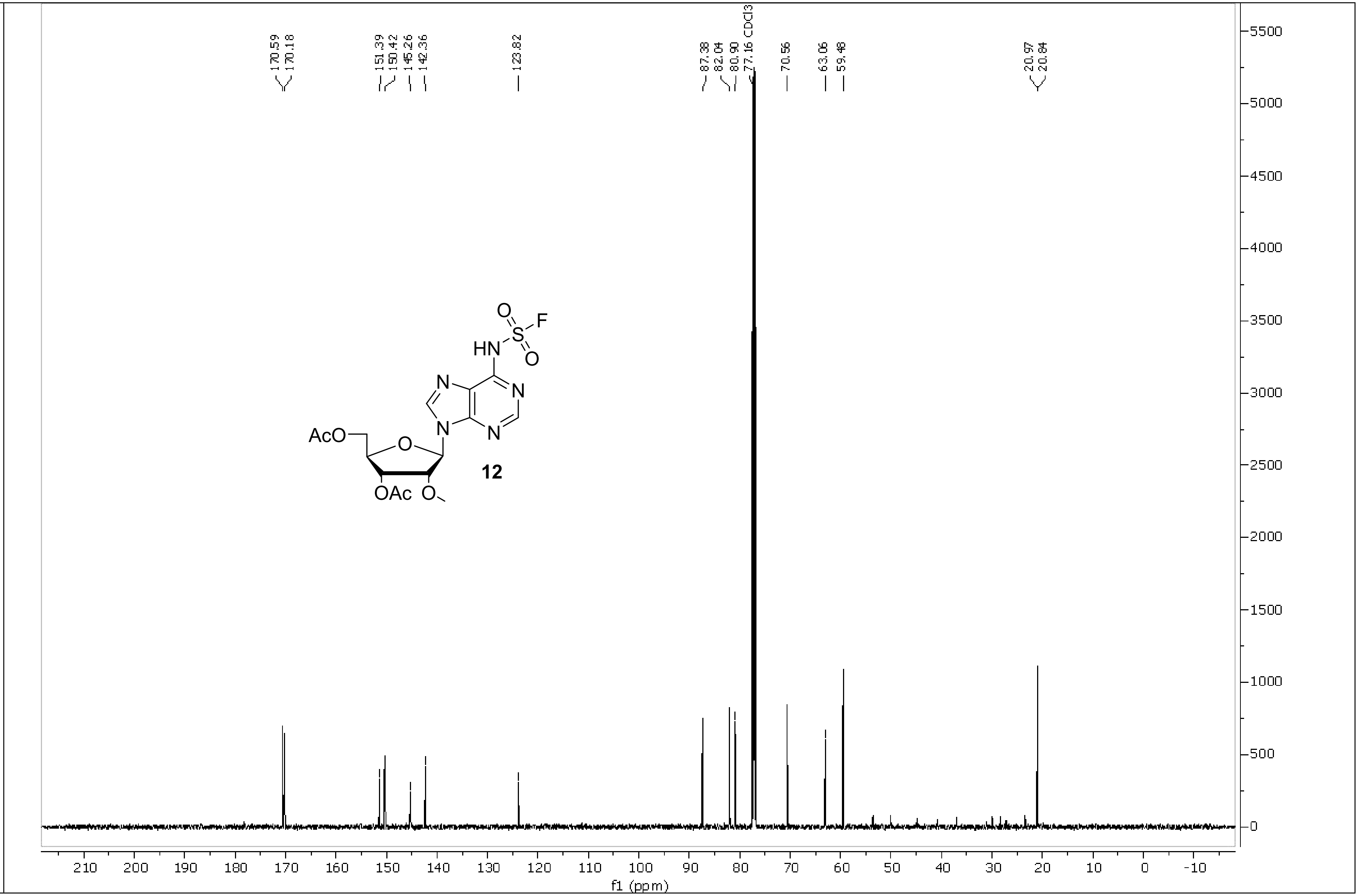

^{19}F NMR spectrum (471 MHz) of **12**

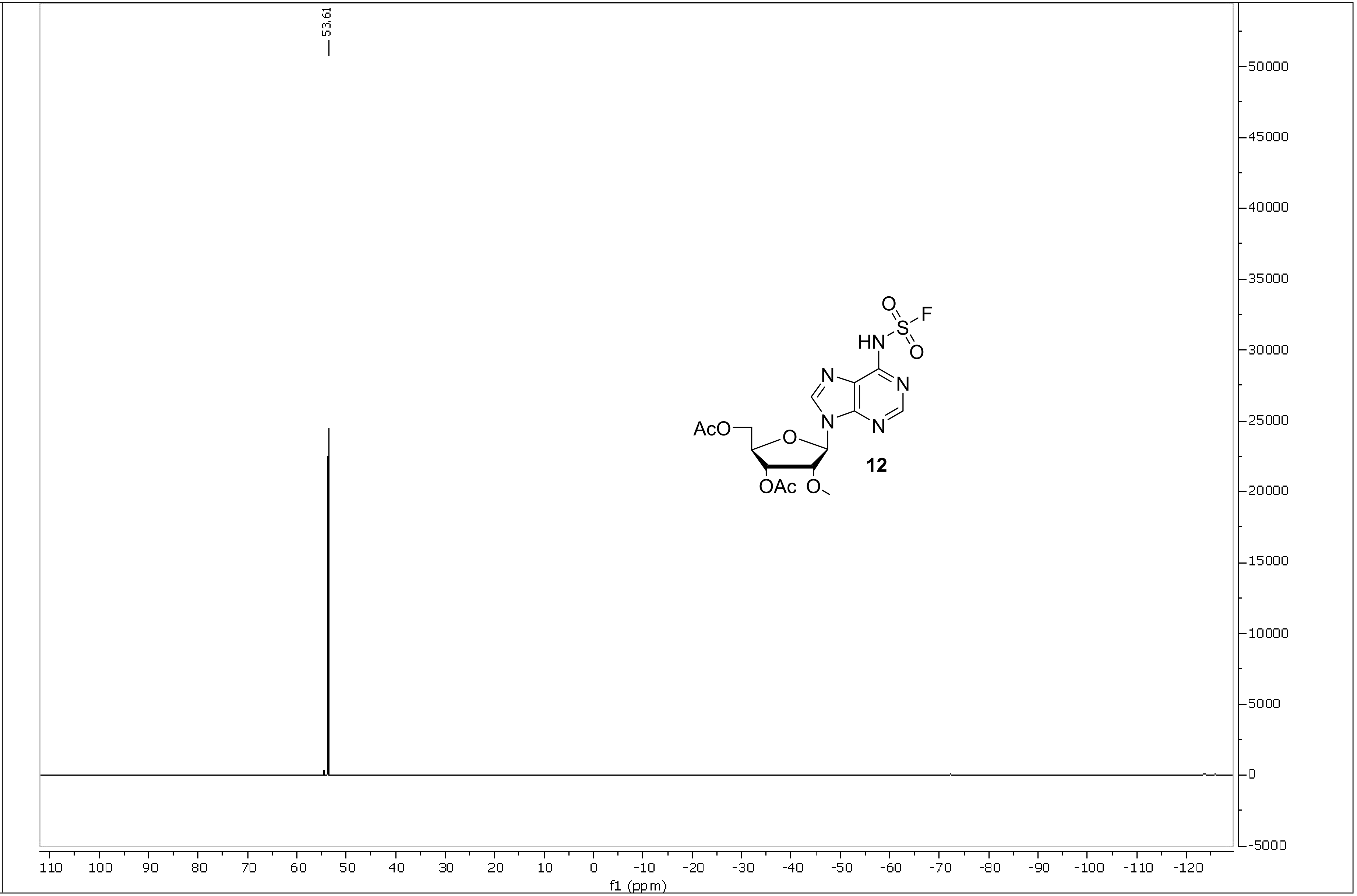

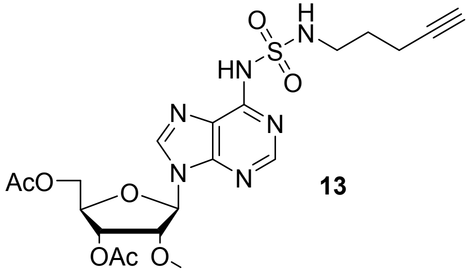

13

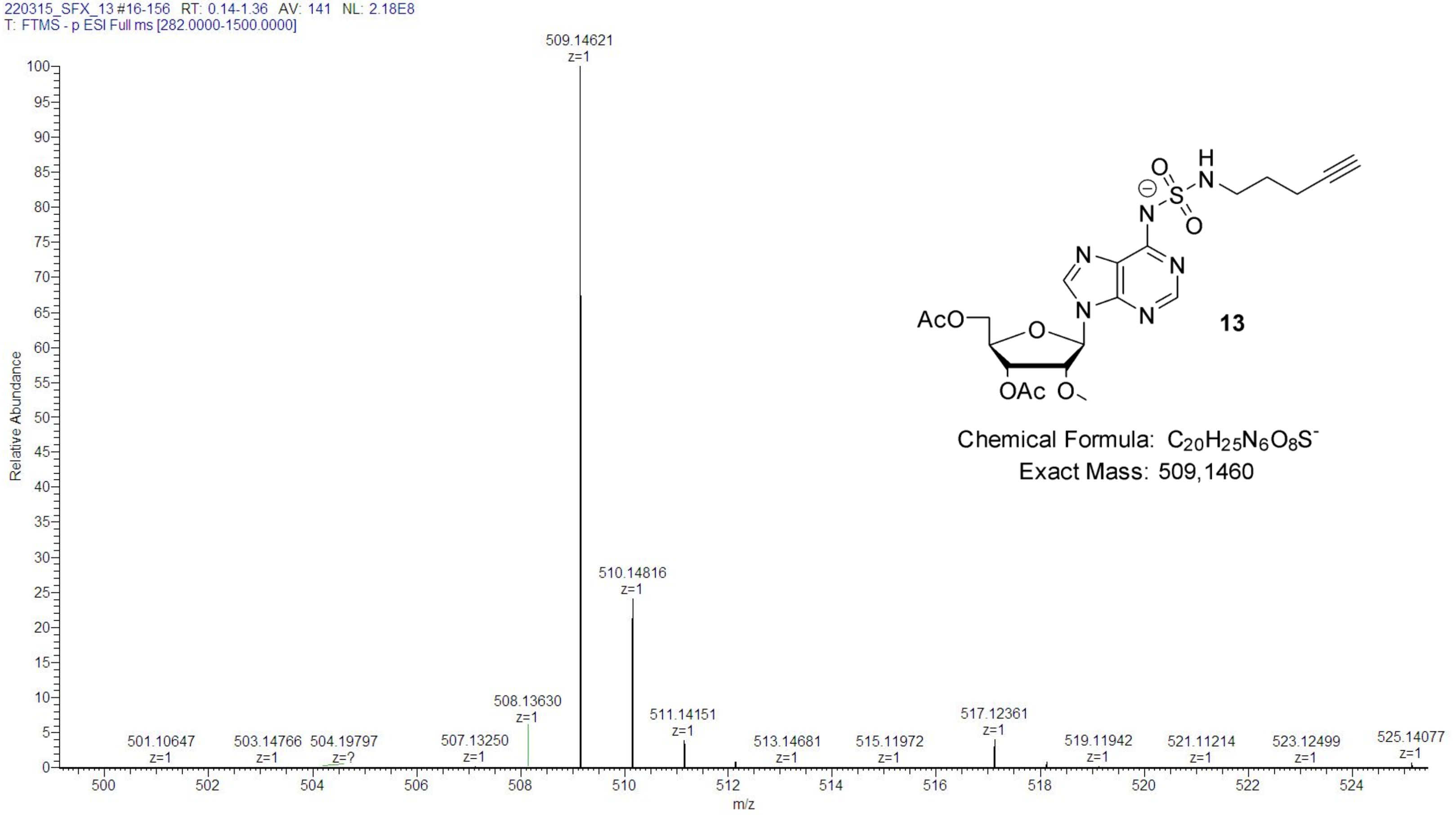

220315_SFX_13 #16-156 RT: 0.14-1.36 AV: 141 NL: 2.18E8
T: FTMS - p ESI Full ms [282.0000-1500.0000]
509.14621 z=1
510.14816 z=1
508.13630 z=1
511.14151 z=1
517.12361 z=1
501.10647 z=1
503.14766 z=1
504.19797 z=?
507.13250 z=1
513.14681 z=1
515.11972 z=1
519.11942 z=1
521.11214 z=1
523.12499 z=1
525.14077 z=1
Relative Abundance
m/z
13
Chemical Formula: $C_{20}H_{25}N_6O_8S^-$
Exact Mass: 509,1460

^{1}H NMR spectrum (500 MHz) of **13**

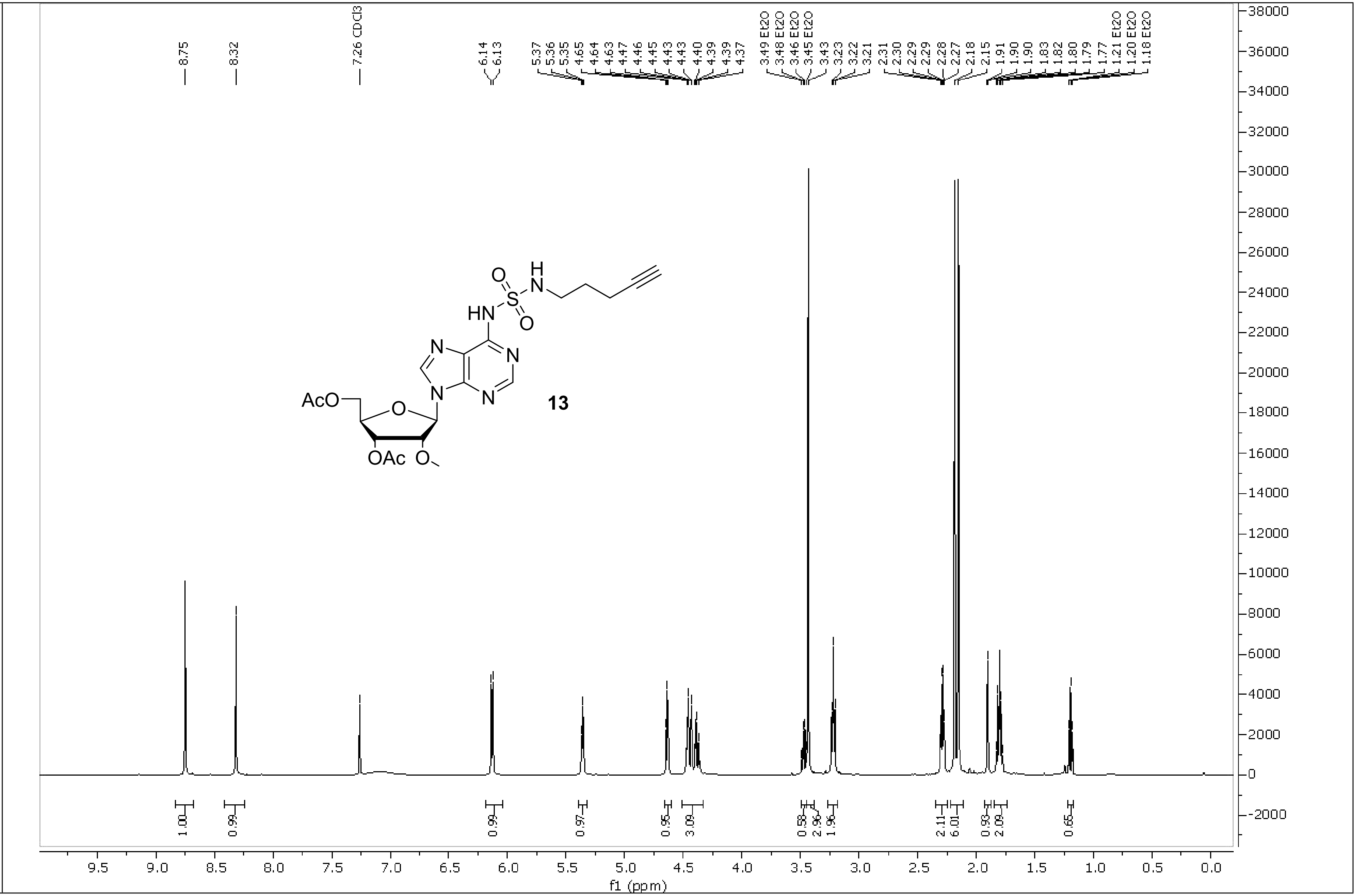

^{13}C NMR spectrum (126 MHz) of **13**

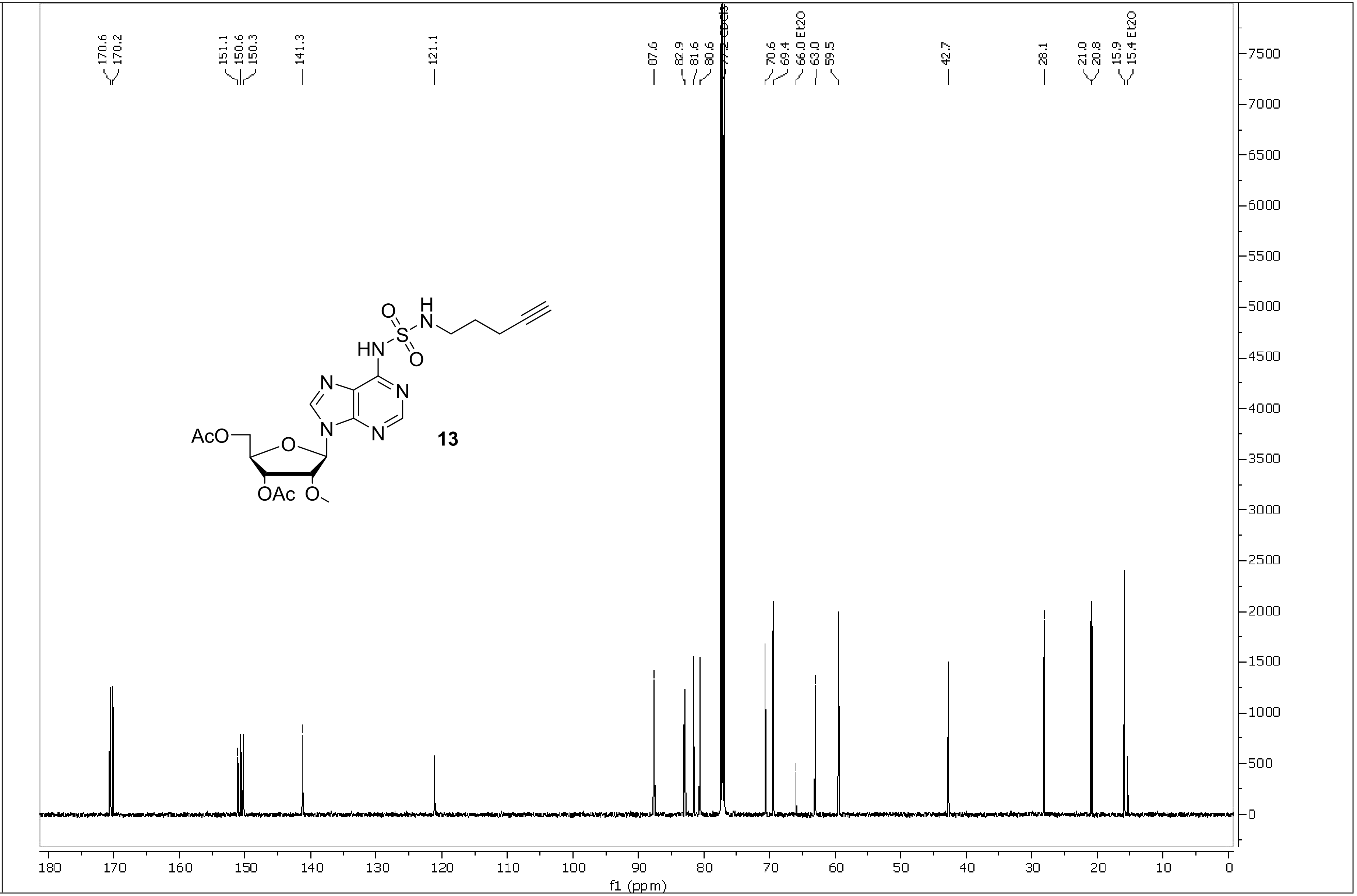

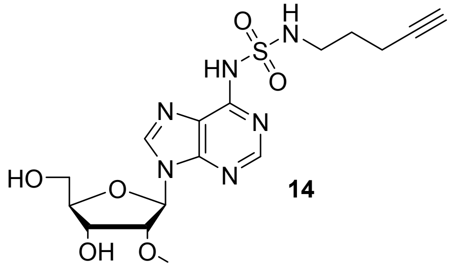

14

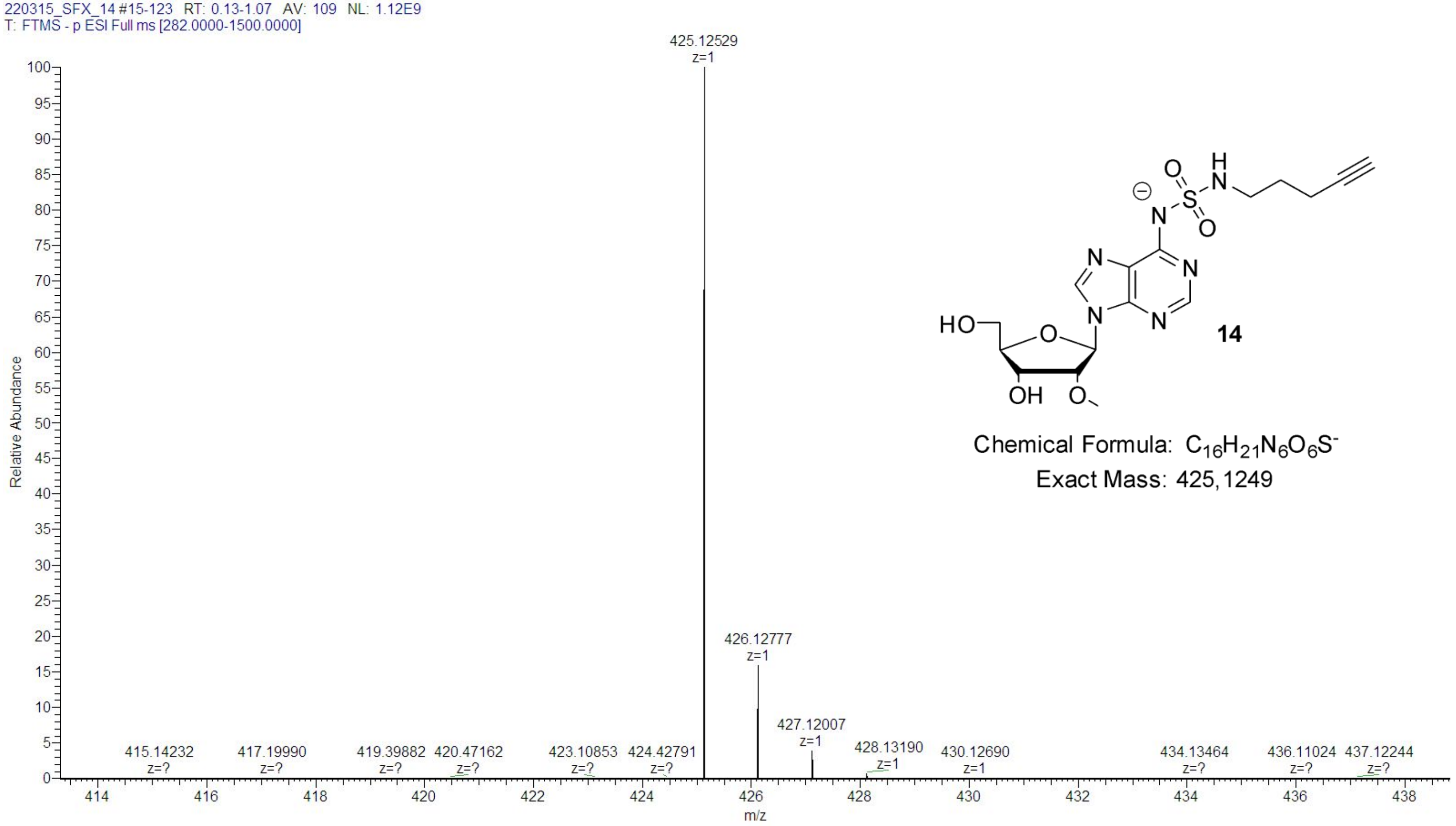

14

Chemical Formula: $C_{16}H_{21}N_6O_6S^-$

Exact Mass: 425,1249

^{1}H NMR spectrum (500 MHz) of **14**

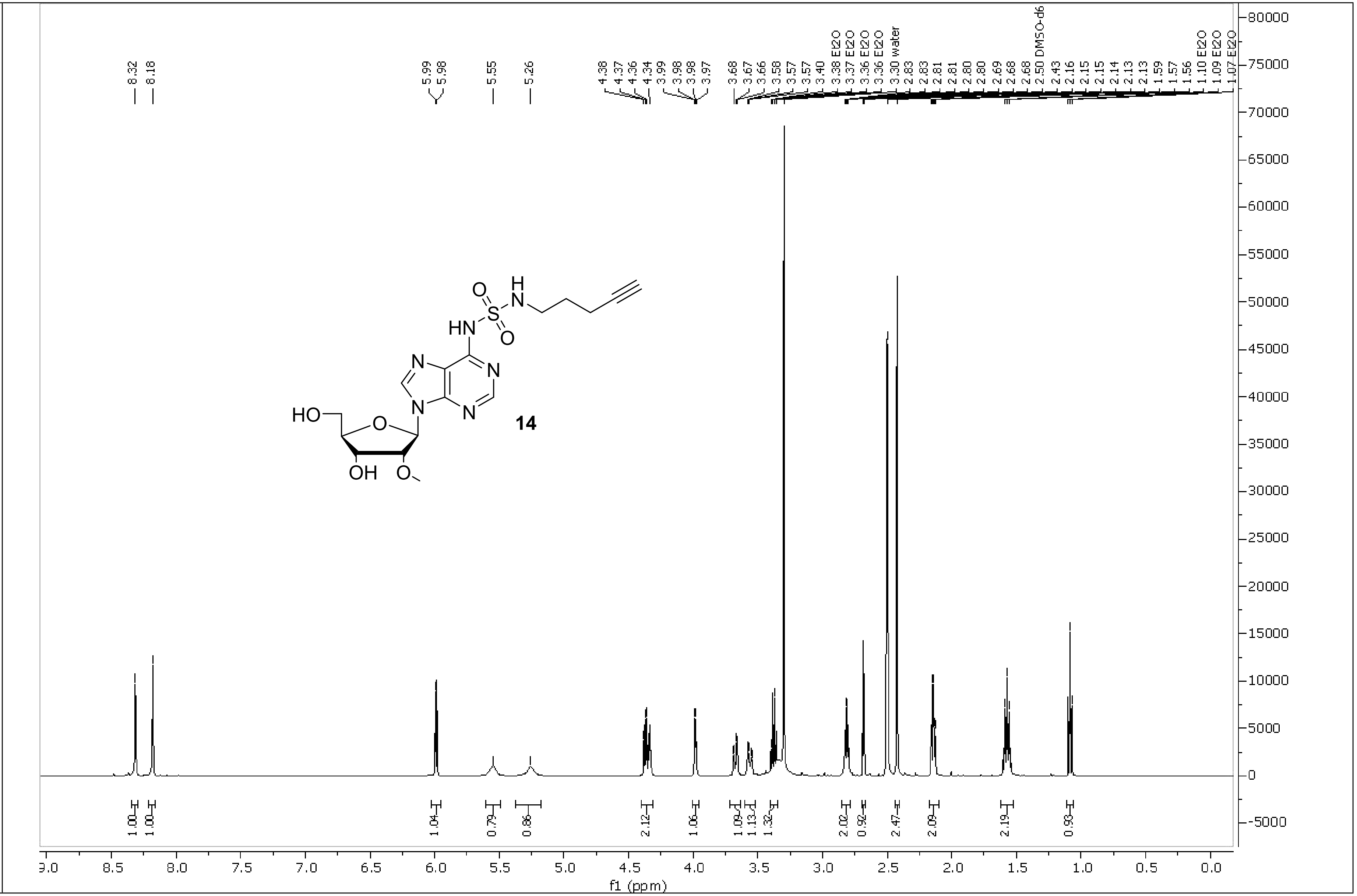

^{13}C NMR spectrum (126 MHz) of **14**

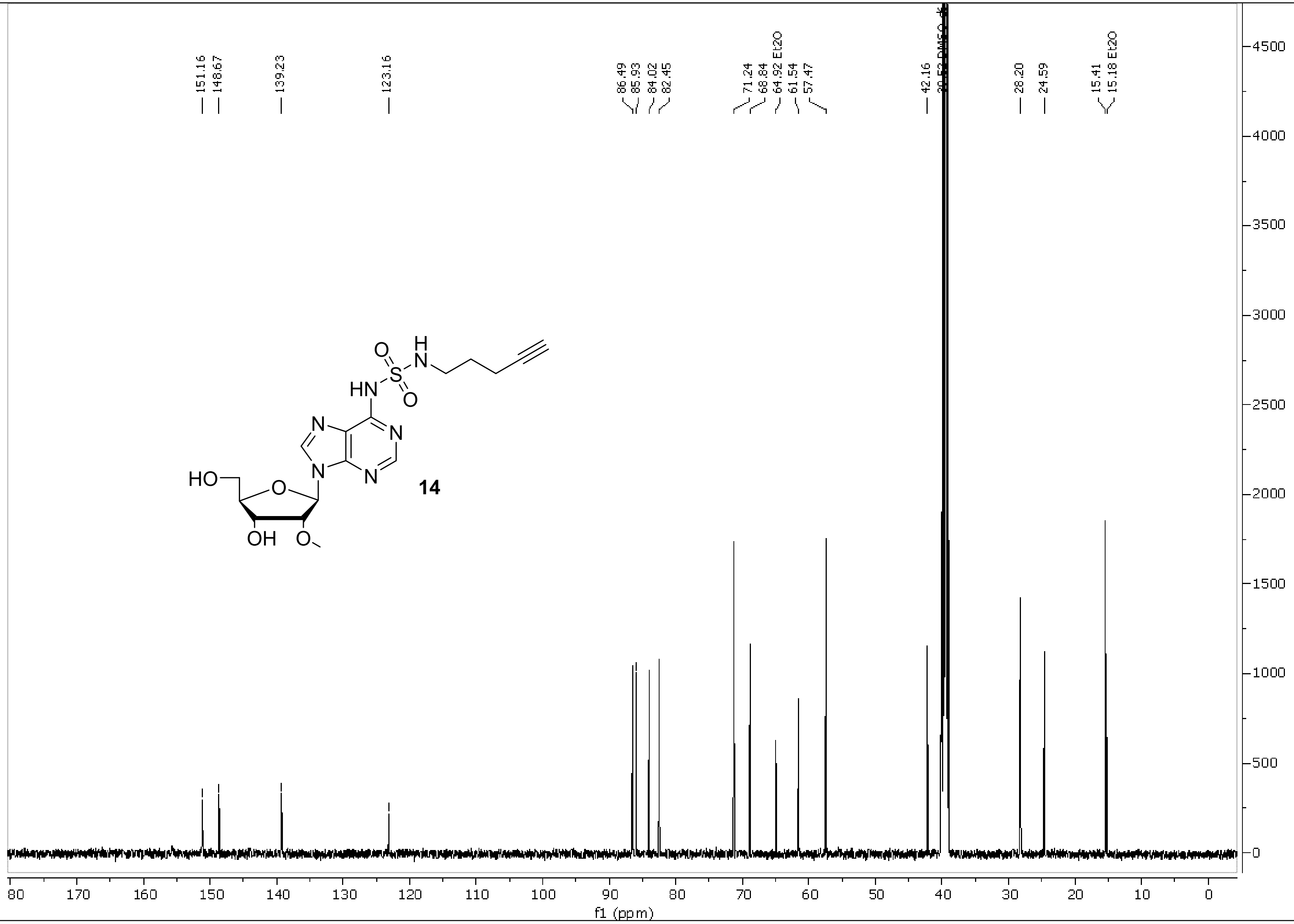

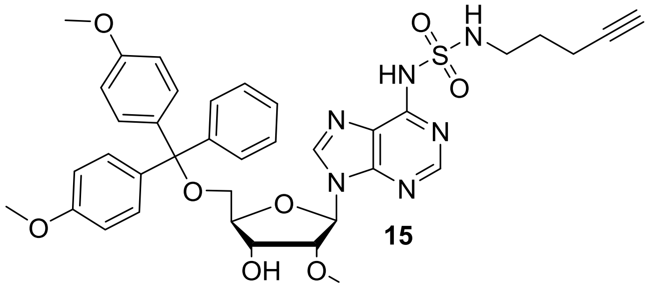
15

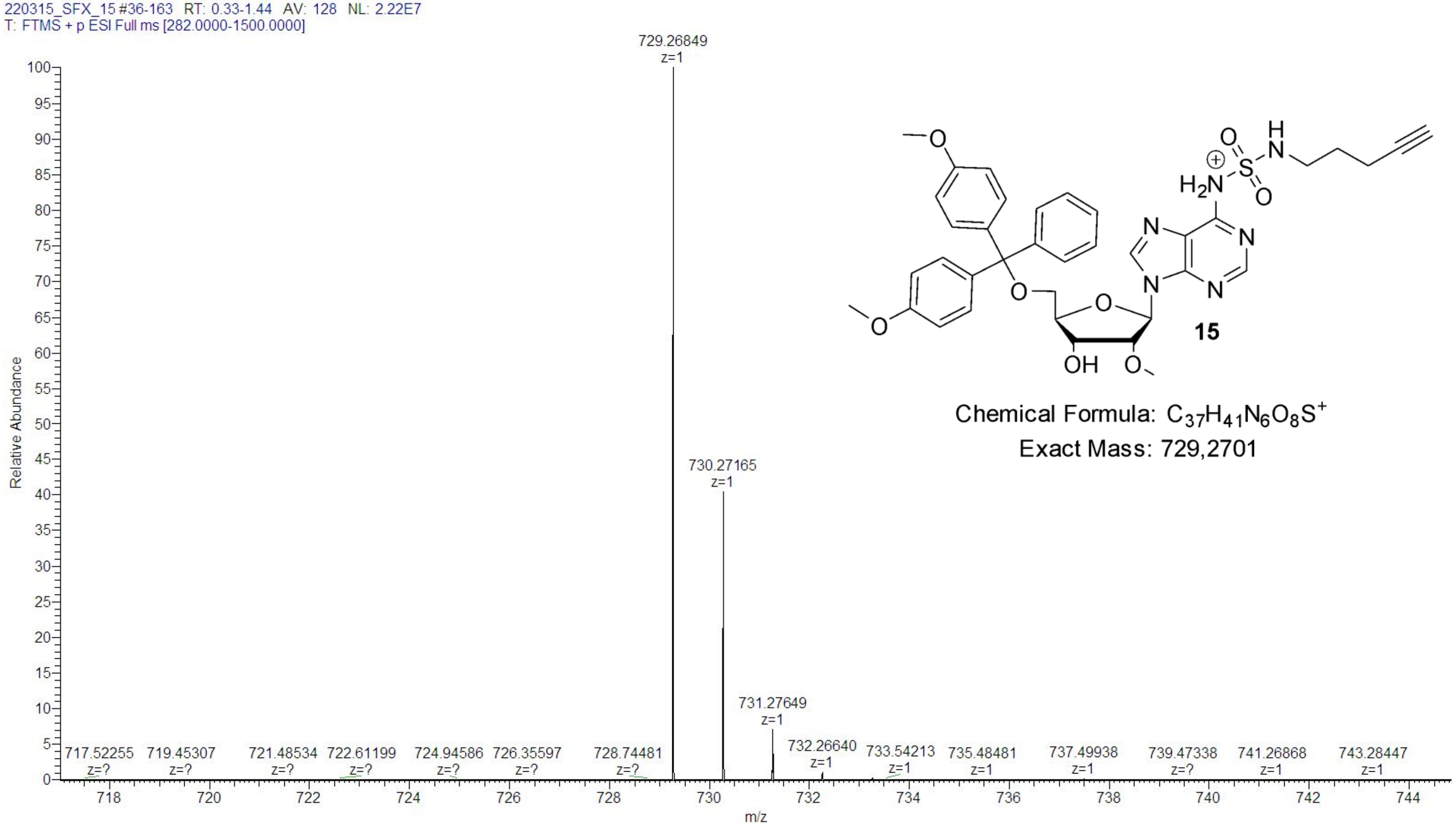

Chemical Formula: $C_{37}H_{41}N_6O_8S^+$
Exact Mass: 729,2701

^{1}H NMR spectrum (500 MHz) of **15**

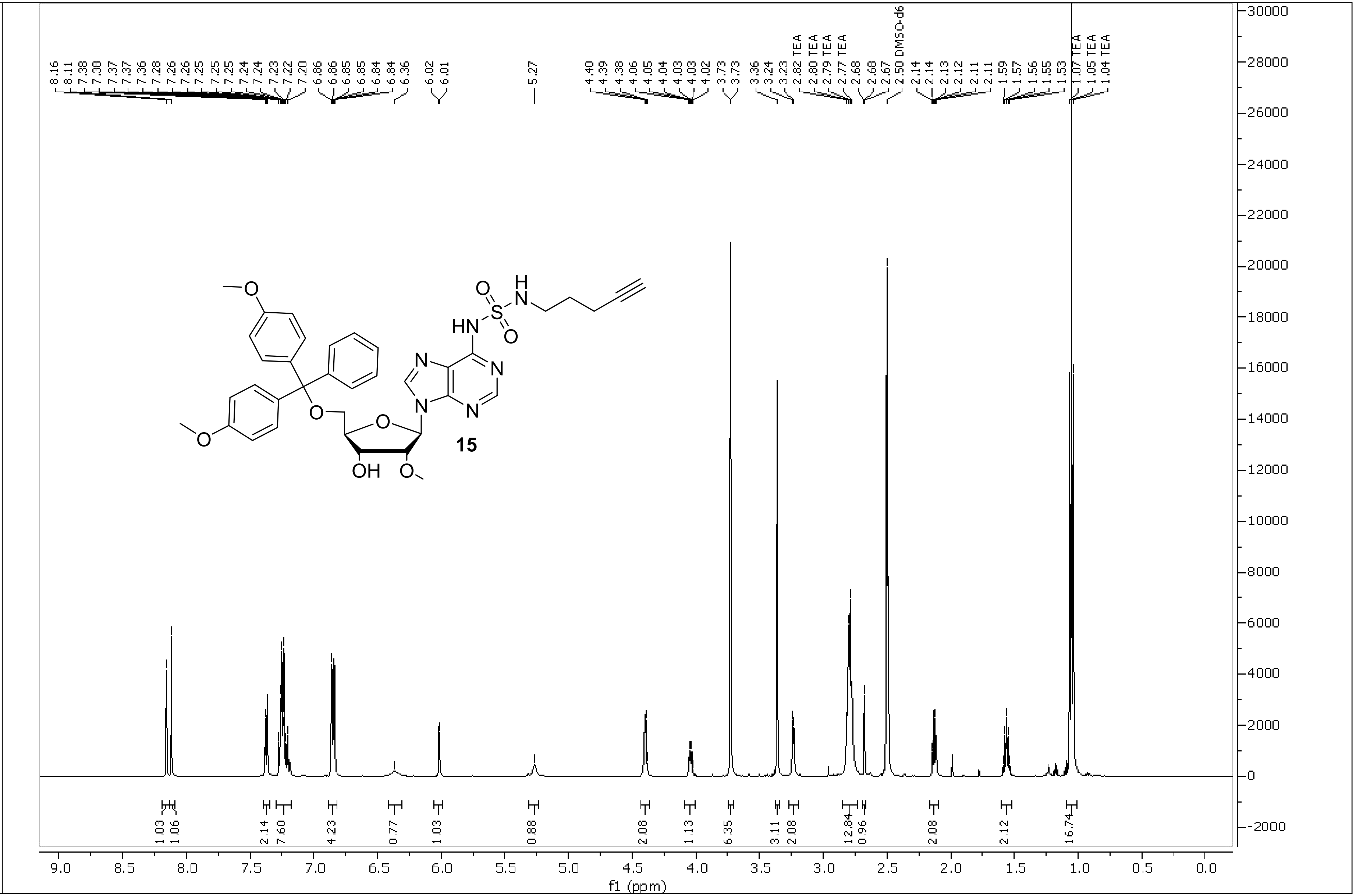

^{13}C NMR spectrum (126 MHz) of **15**

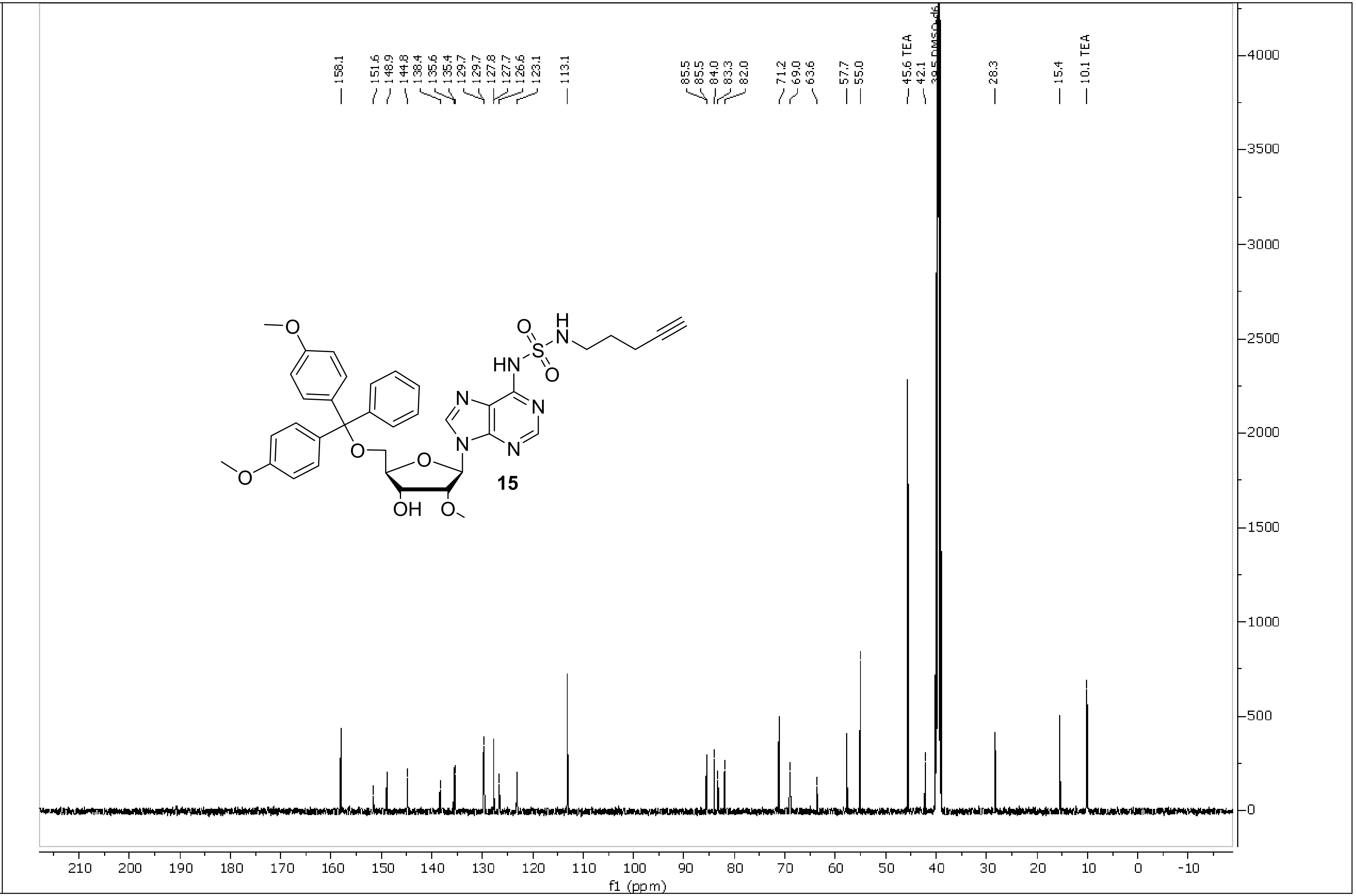

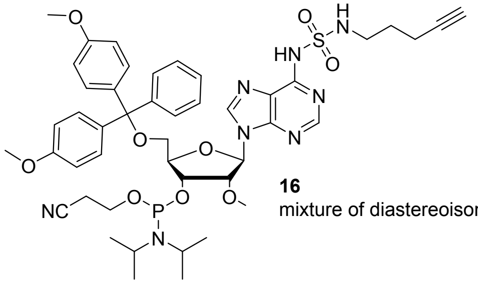

16
mixture of diastereoisomers

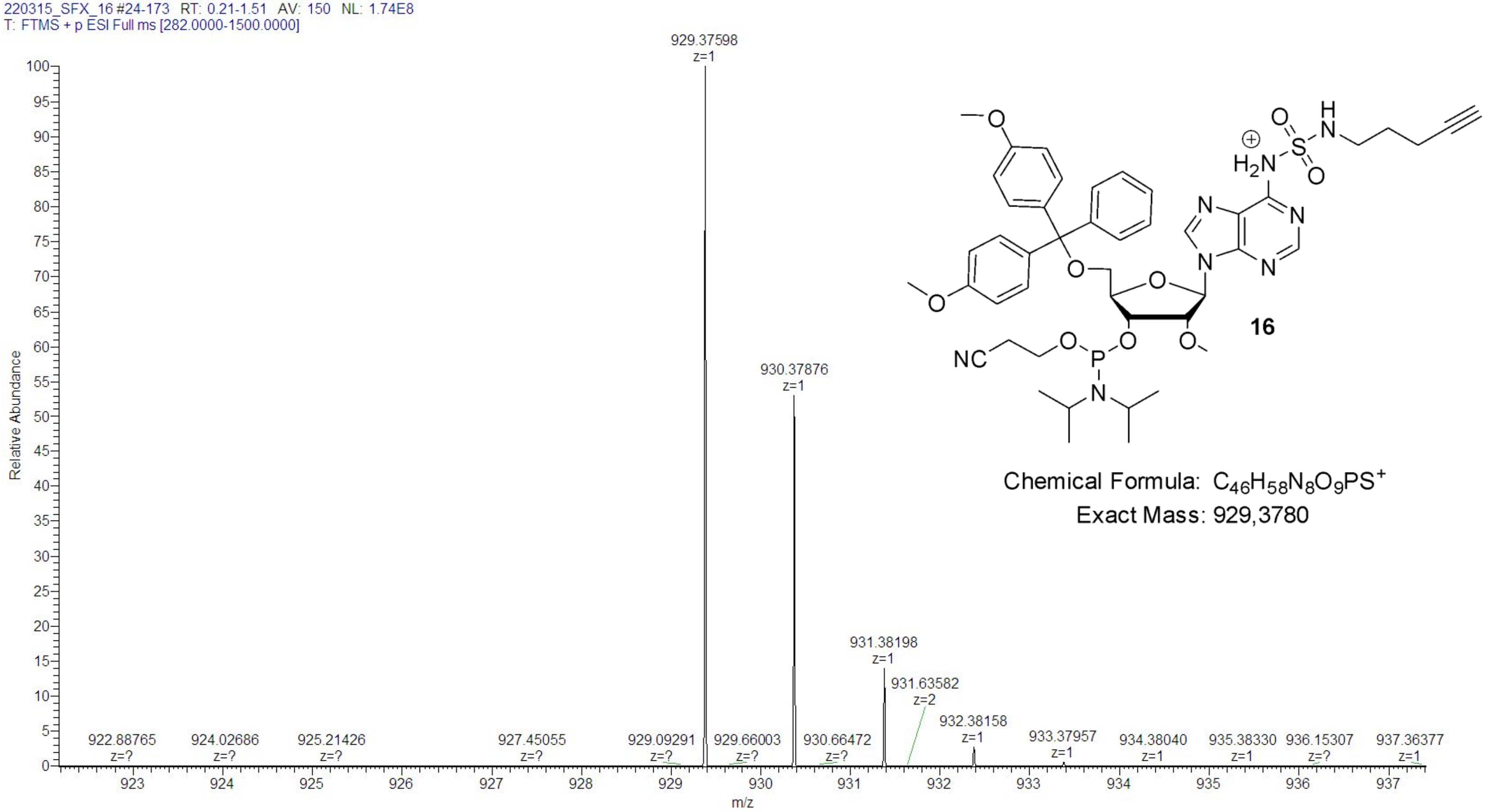

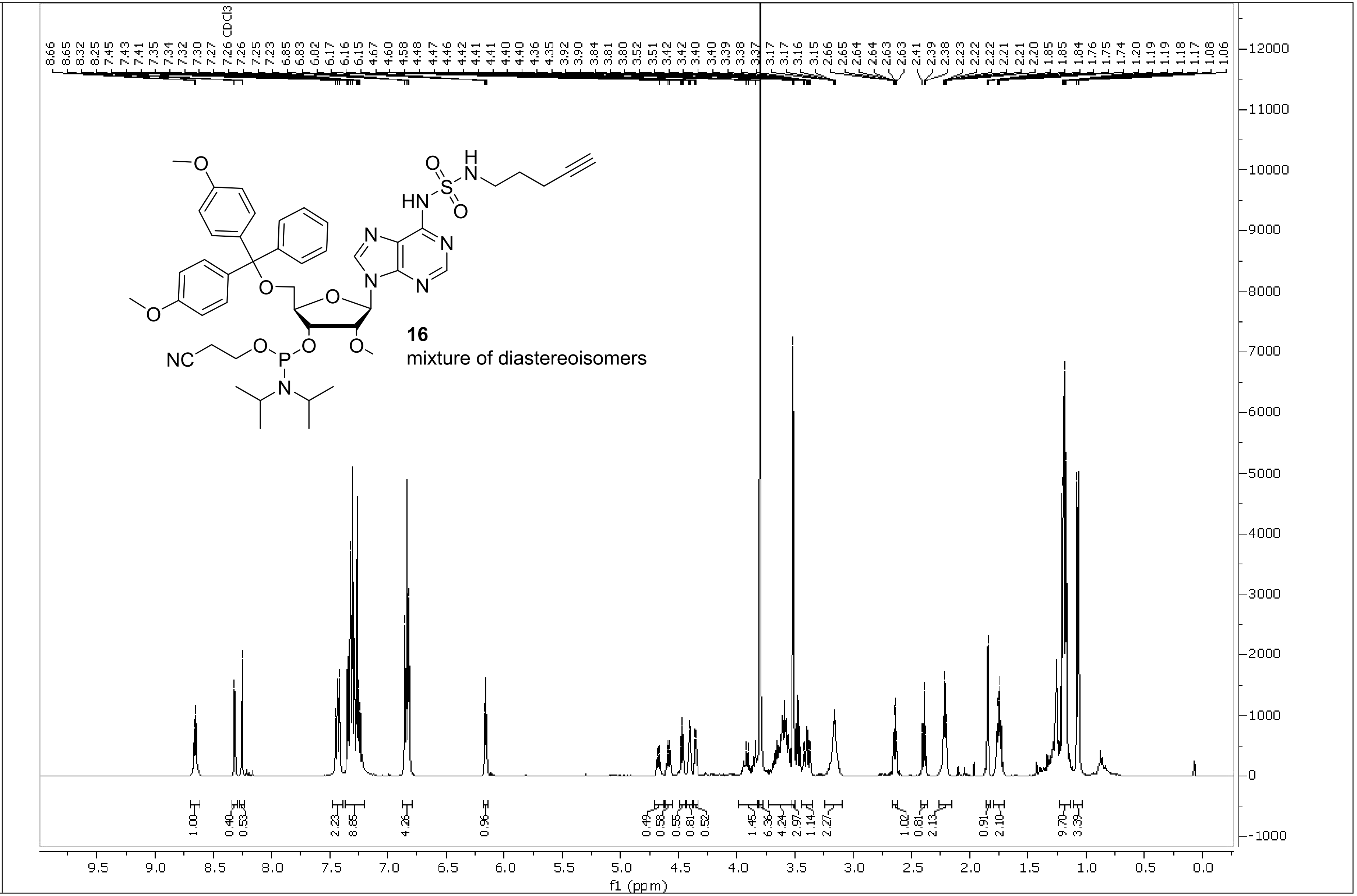

^{1}H NMR spectrum (500 MHz) of **16**

^{13}C NMR spectrum (126 MHz) of **16**

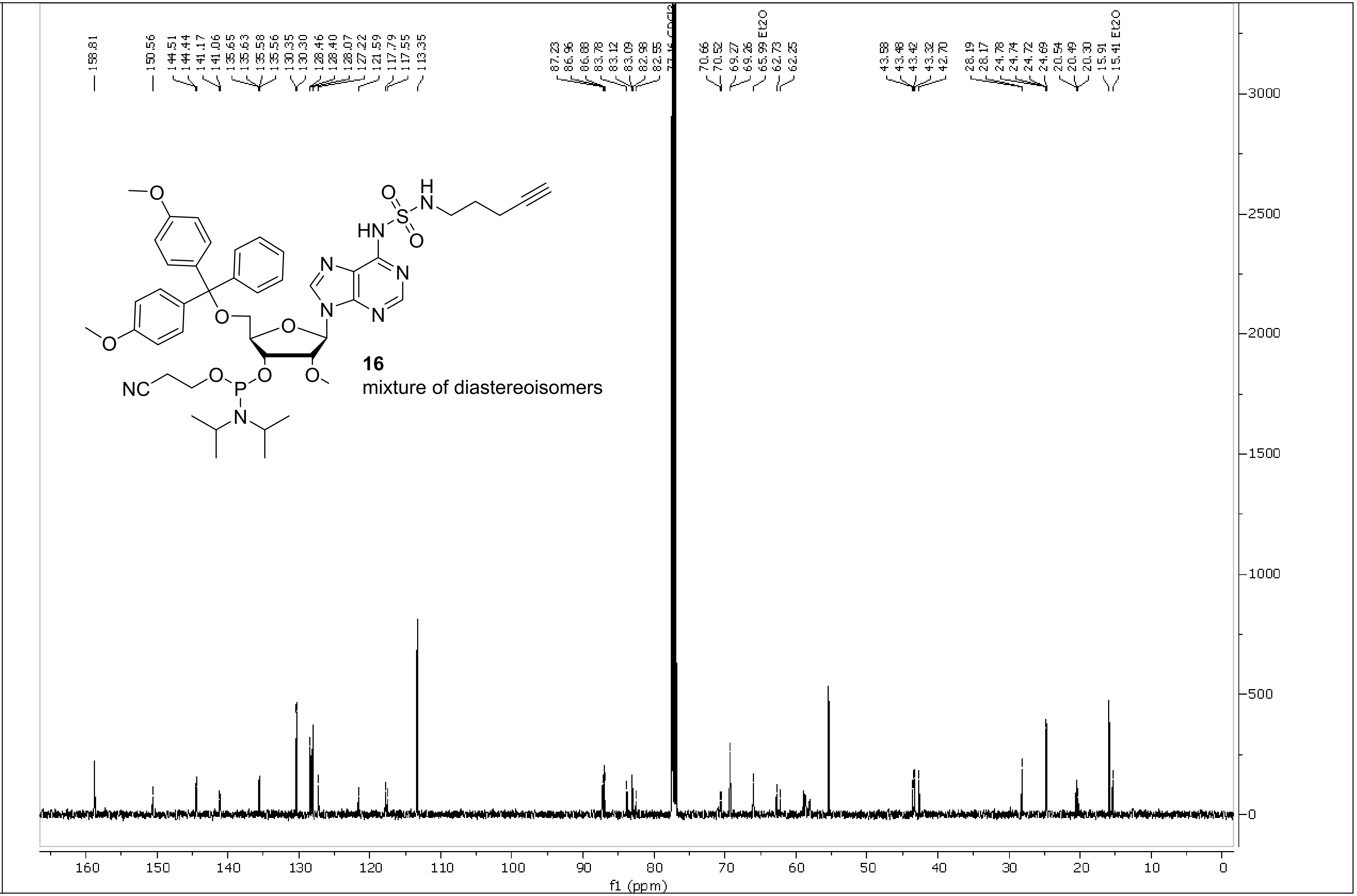

COSY NMR spectrum of **16**

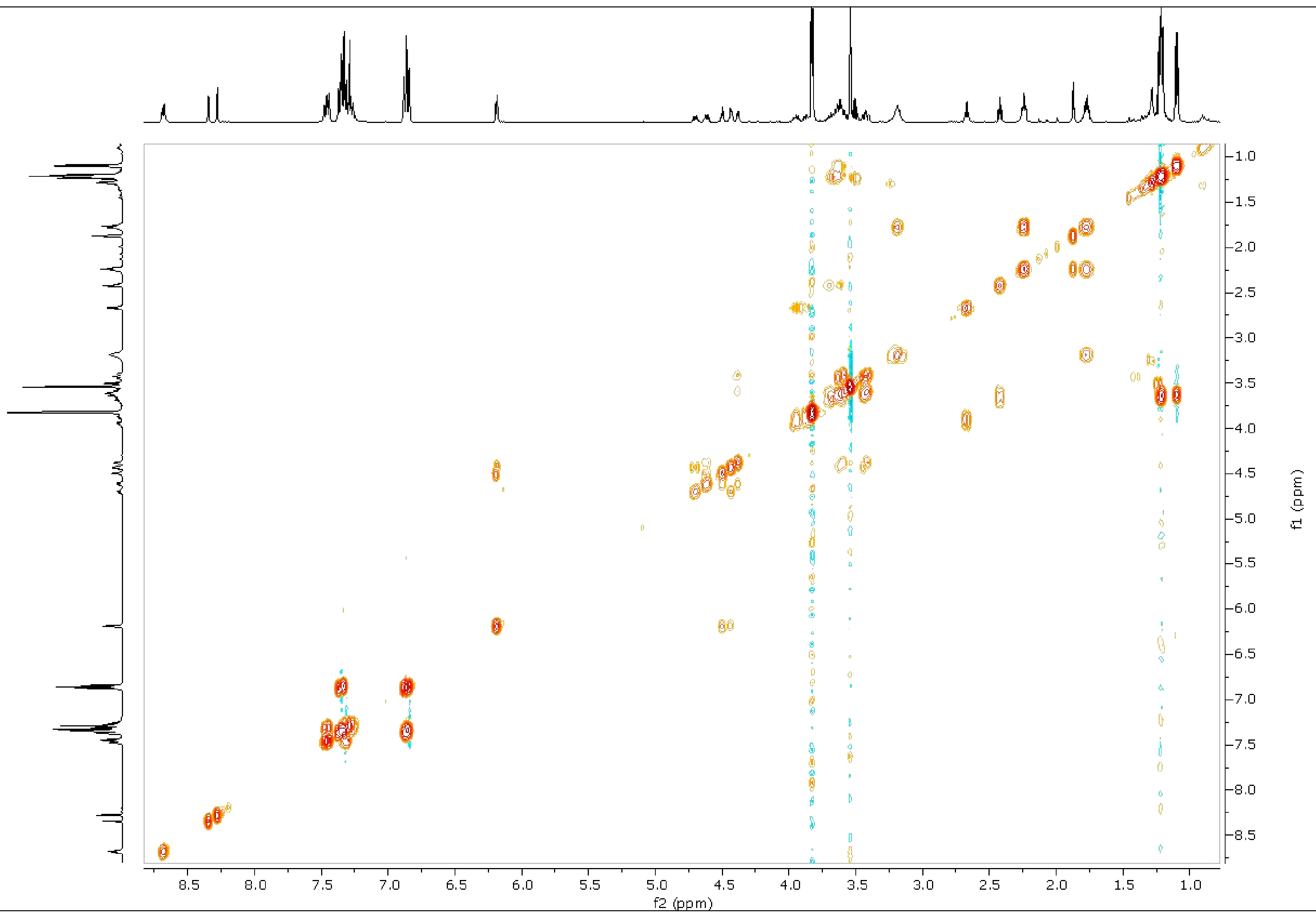

^{1}H – ^{13}C HSQC NMR spectrum of **16**

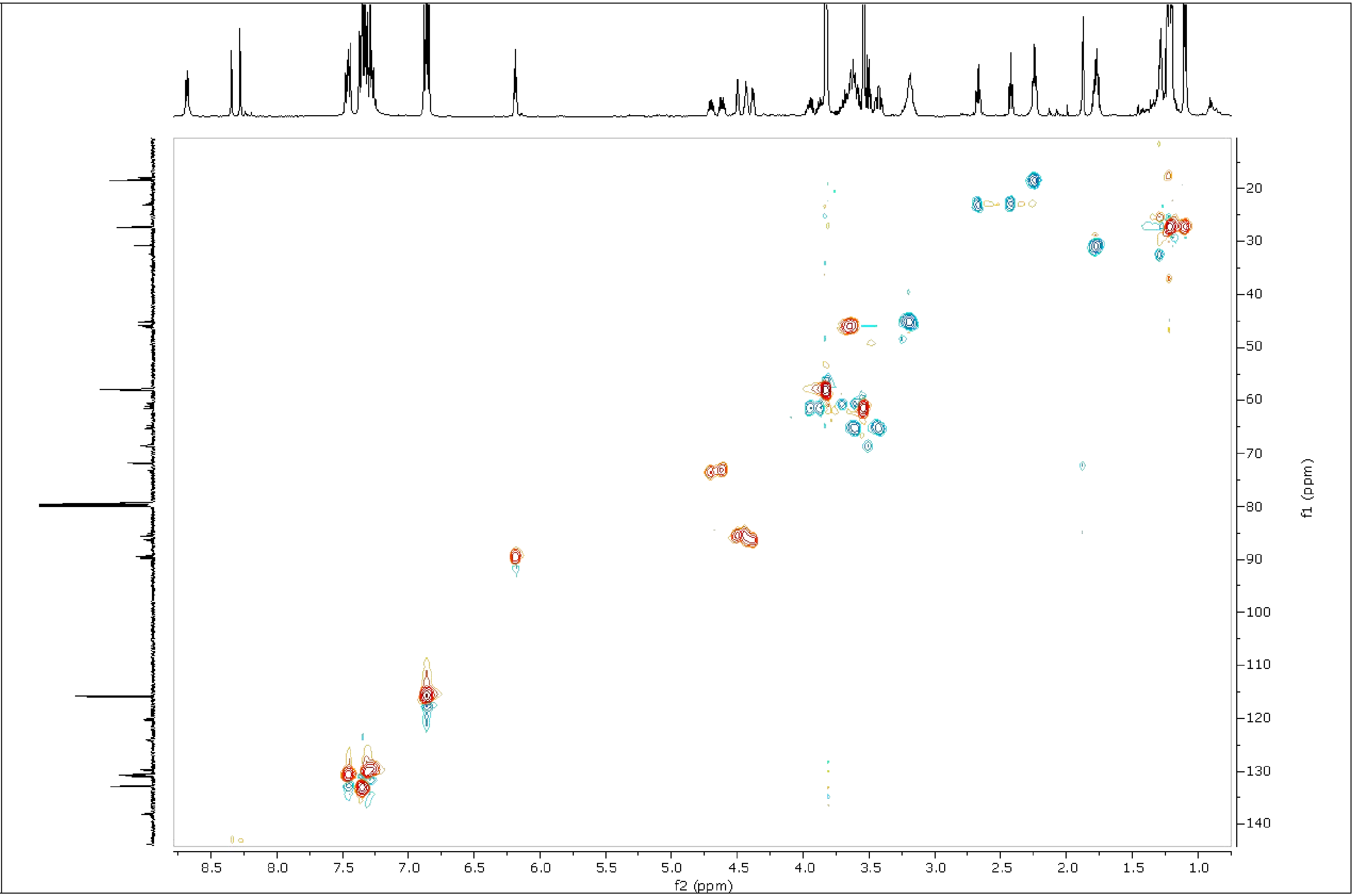

^{1}H – ^{31}P HMBC NMR spectrum of **16**

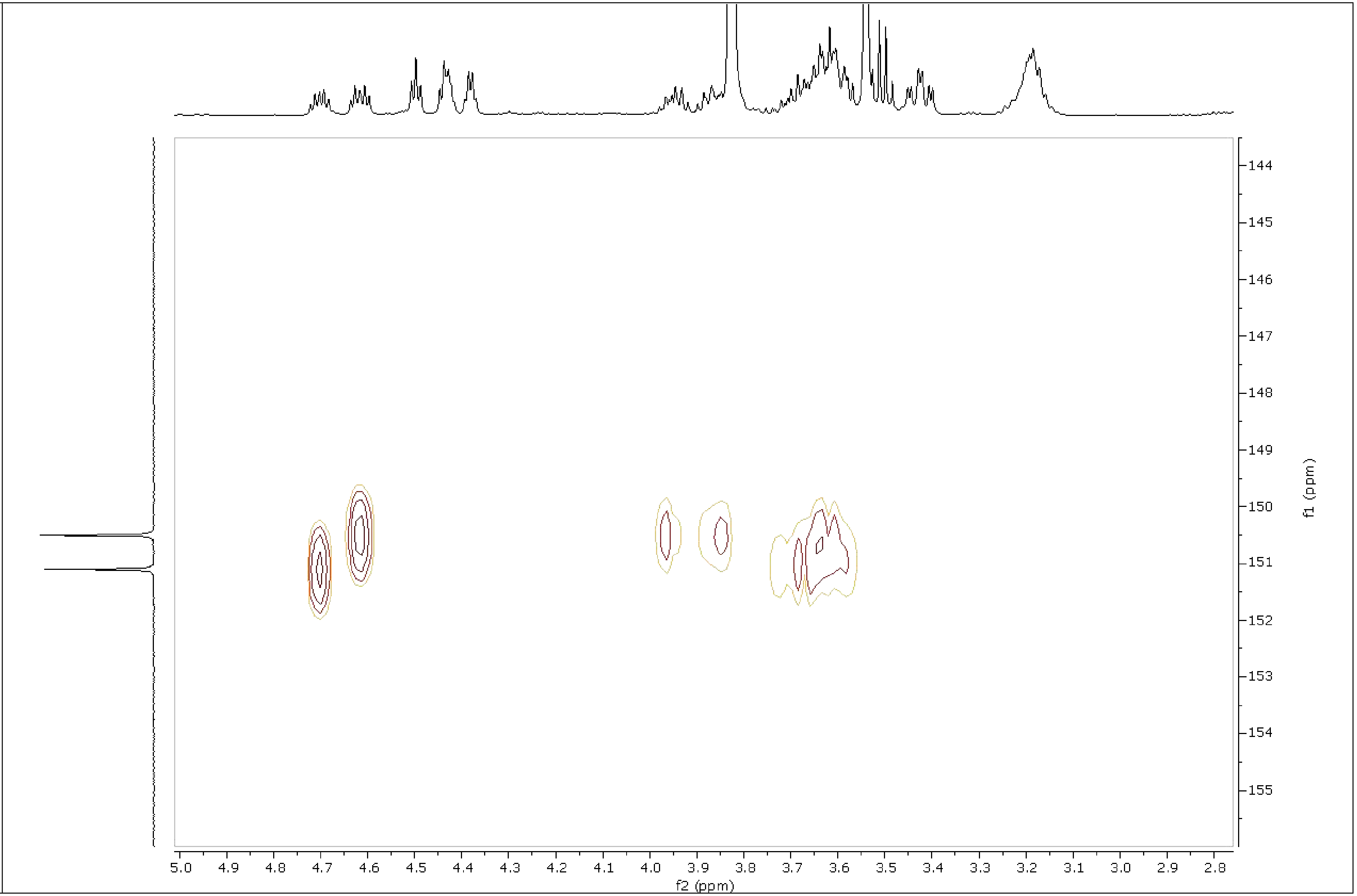

^{31}P NMR spectrum (202 MHz) of **16**

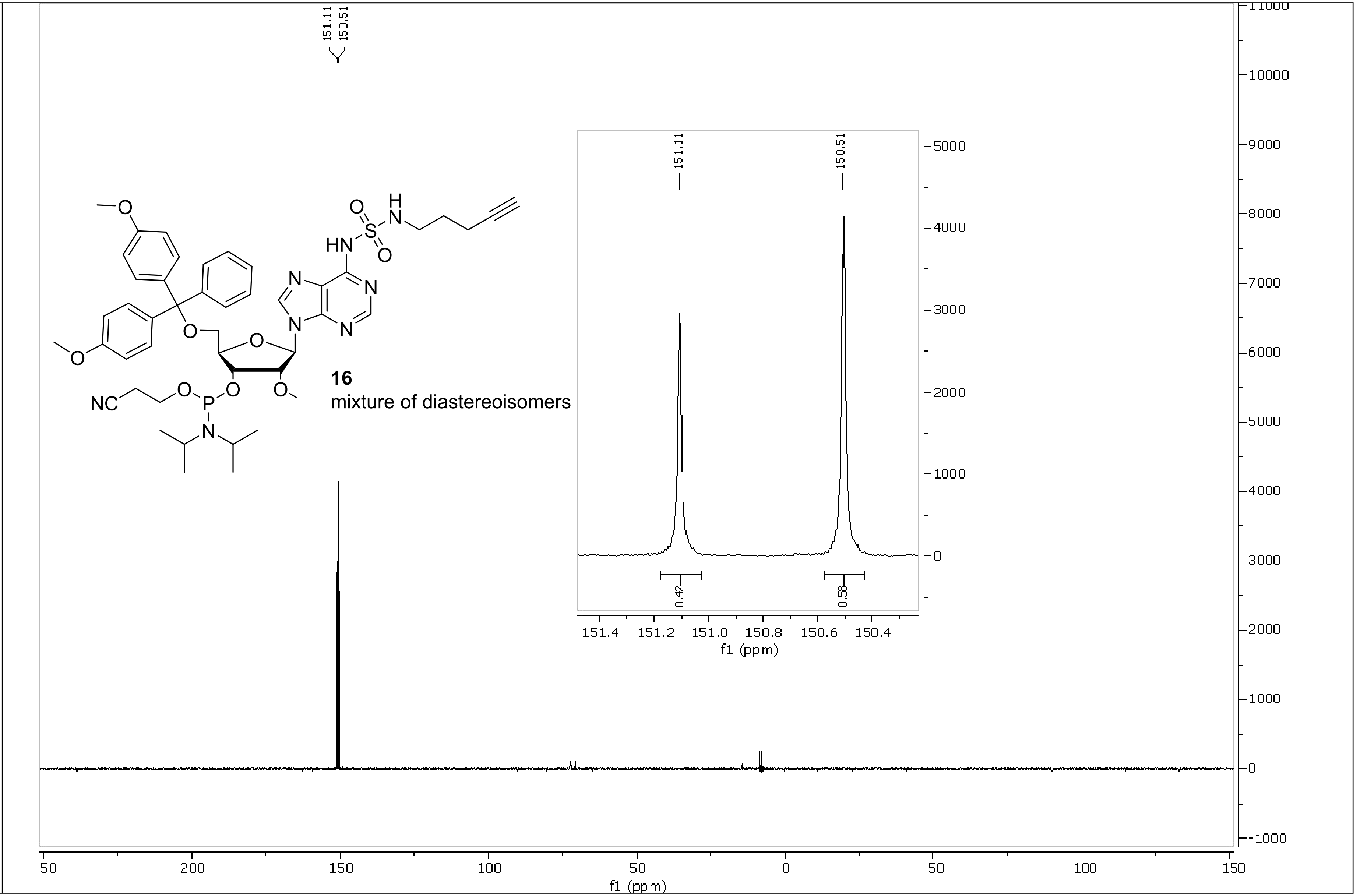

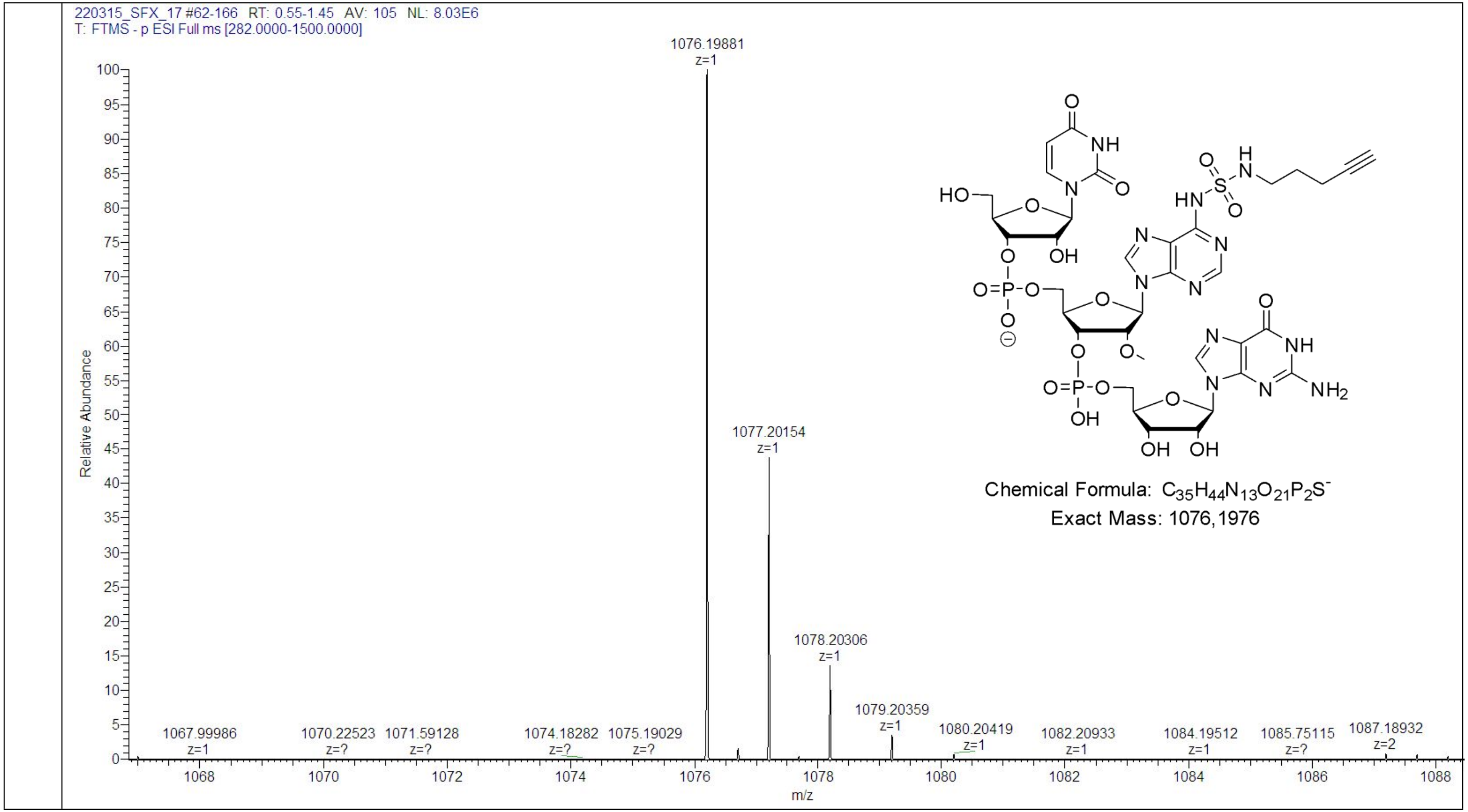
220315_SFX_17 #62-166 RT: 0.55-1.45 AV: 105 NL: 8.03E6
T: FTMS - p ESI Full ms [282.0000-1500.0000]
1076.19881 z=1
1077.20154 z=1
1078.20306 z=1
1079.20359 z=1
1080.20419 z=1
1082.20933 z=1
1084.19512 z=1
1085.75115 z=?
1087.18932 z=2
1067.99986 z=1
1070.22523 z=?
1071.59128 z=?
1074.18282 z=?
1075.19029 z=?
Relative Abundance
m/z
Chemical Formula: $C_{35}H_{44}N_{13}O_{21}P_2S^-$
Exact Mass: 1076,1976

^1H NMR spectrum (500 MHz) of **17**

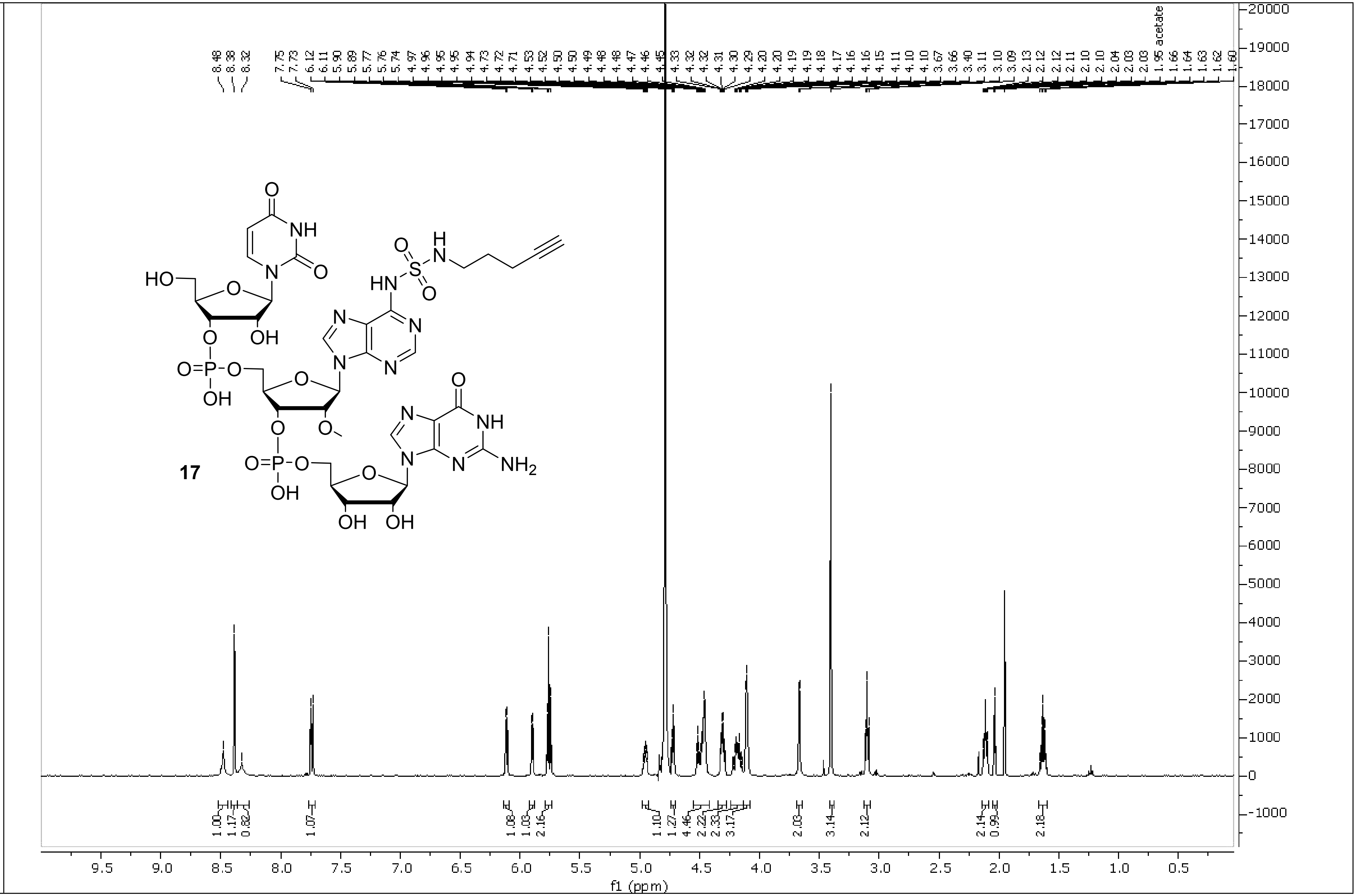

^{1}H NMR spectrum (500 MHz) of **17** (expand)

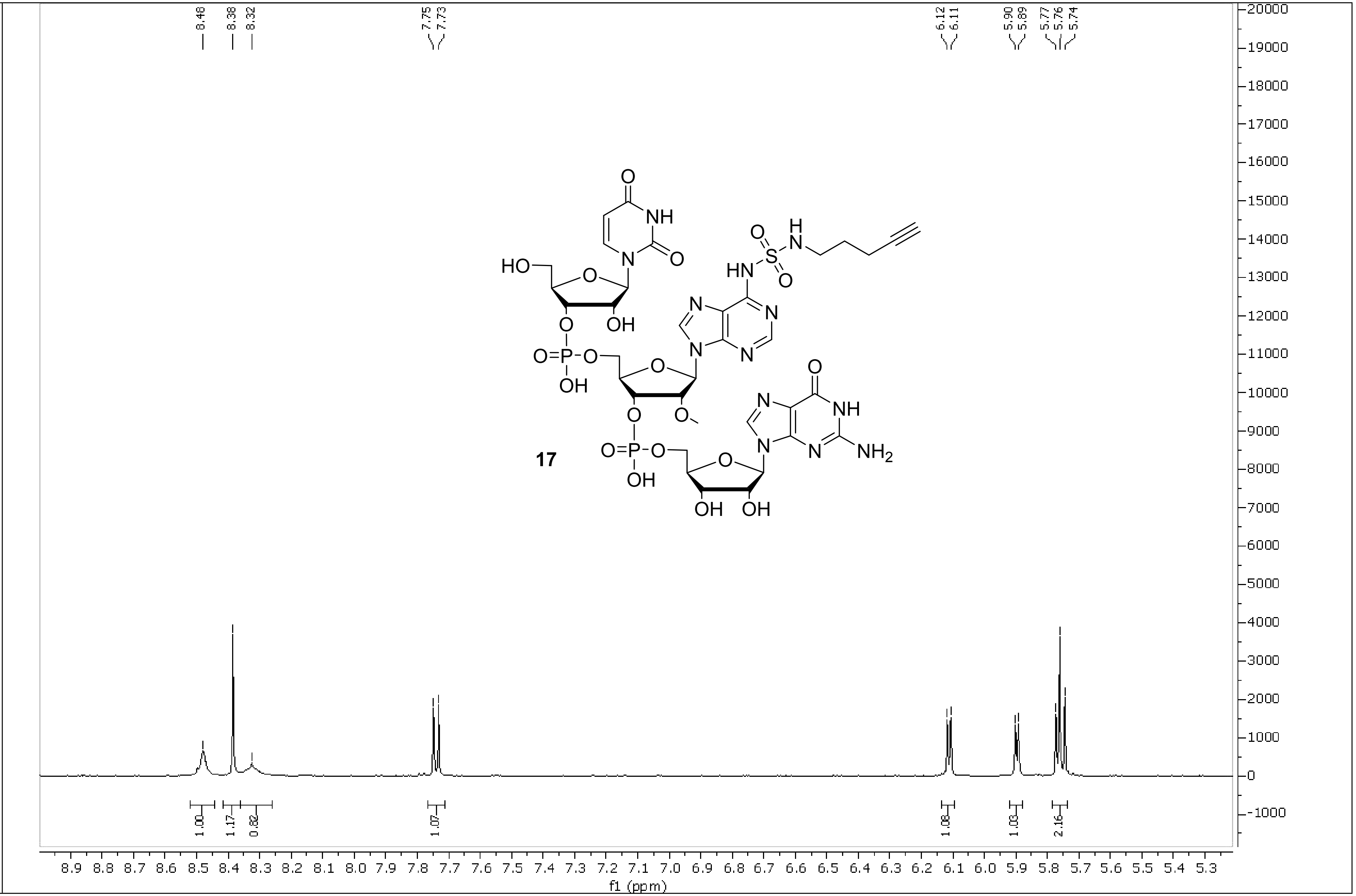

^{1}H NMR spectrum (500 MHz) of **17** (expand)

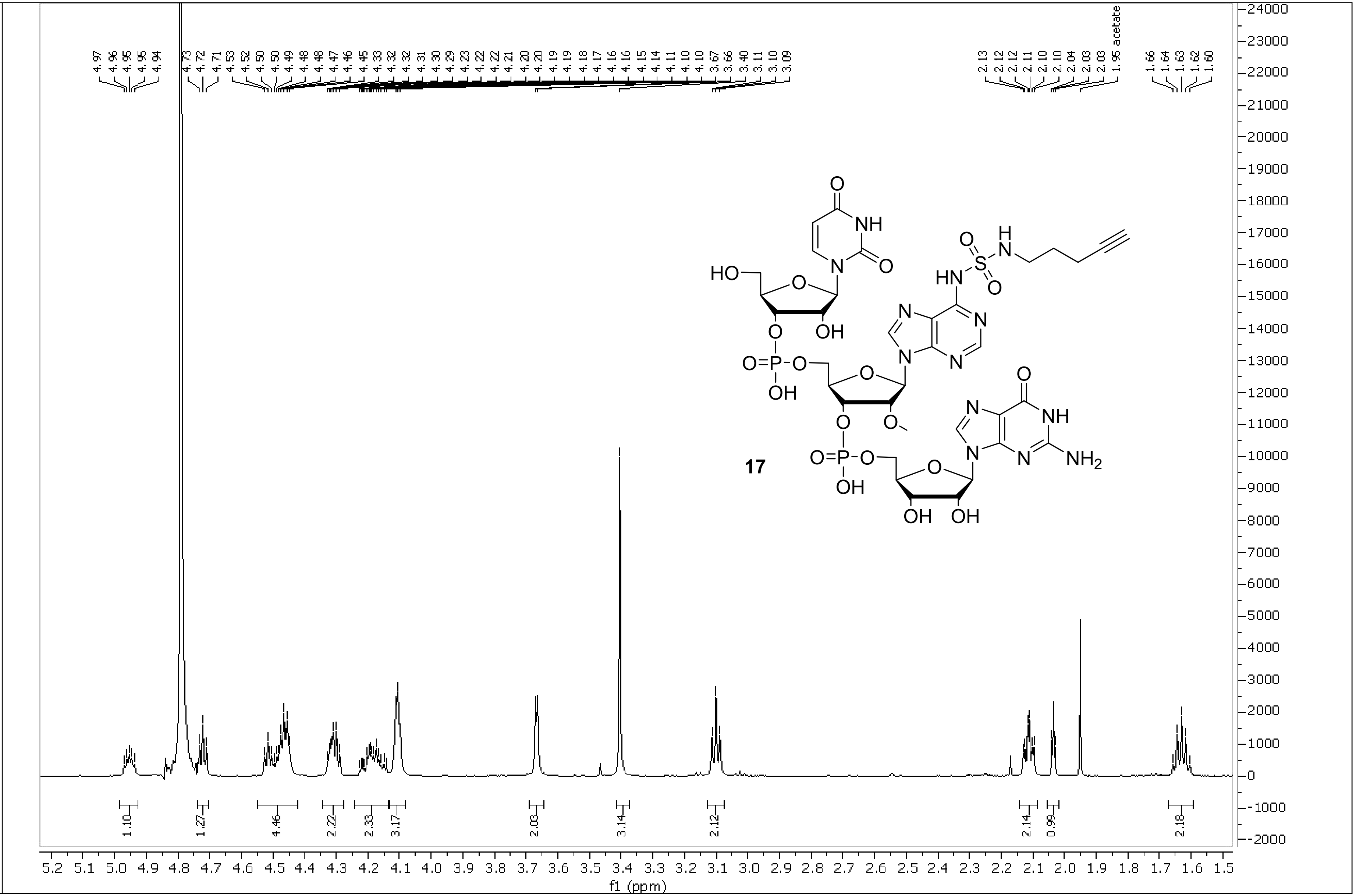

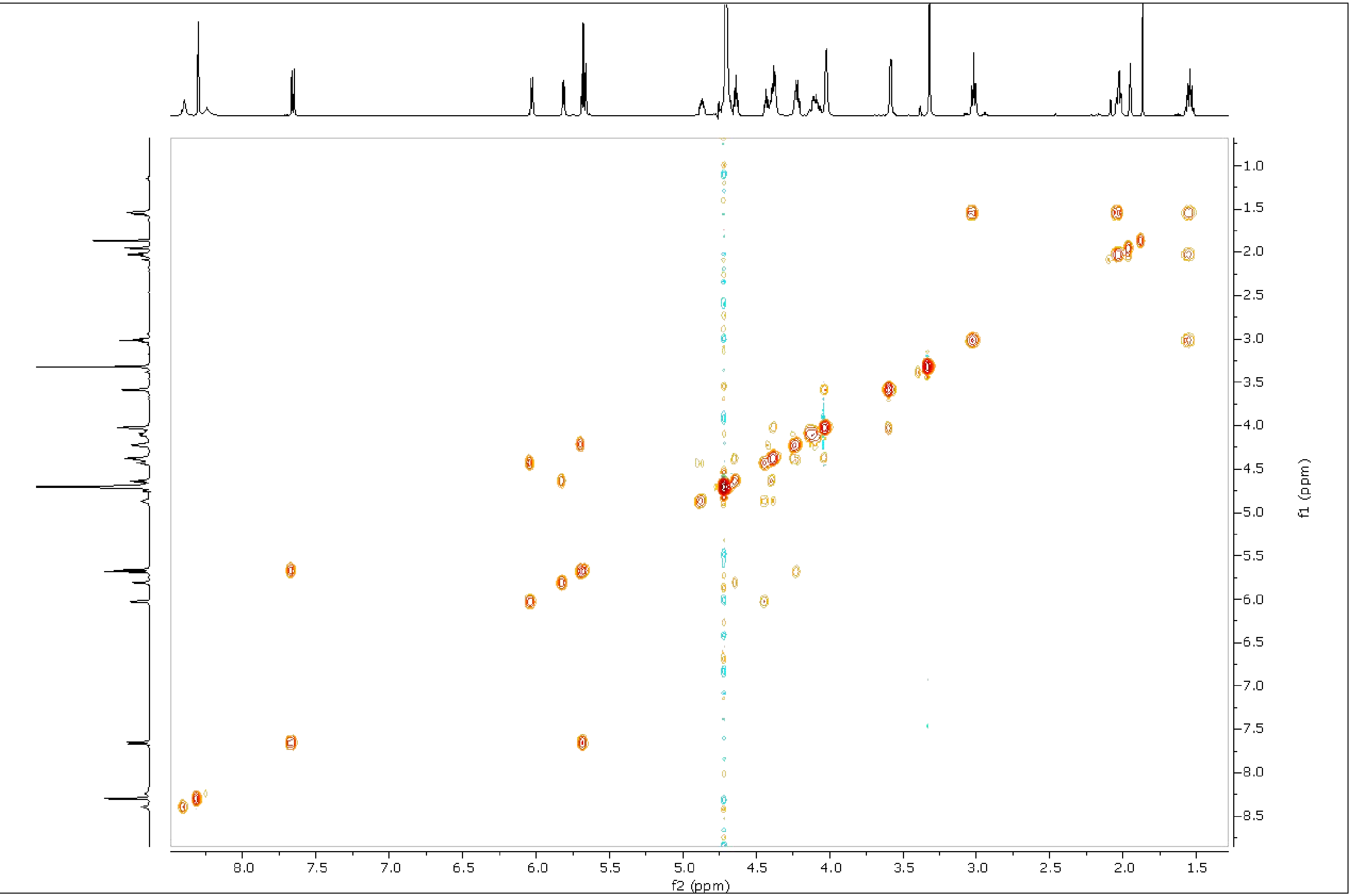

COSY NMR spectrum of **17**

^{1}H – ^{13}C HSQC NMR spectrum of **17**

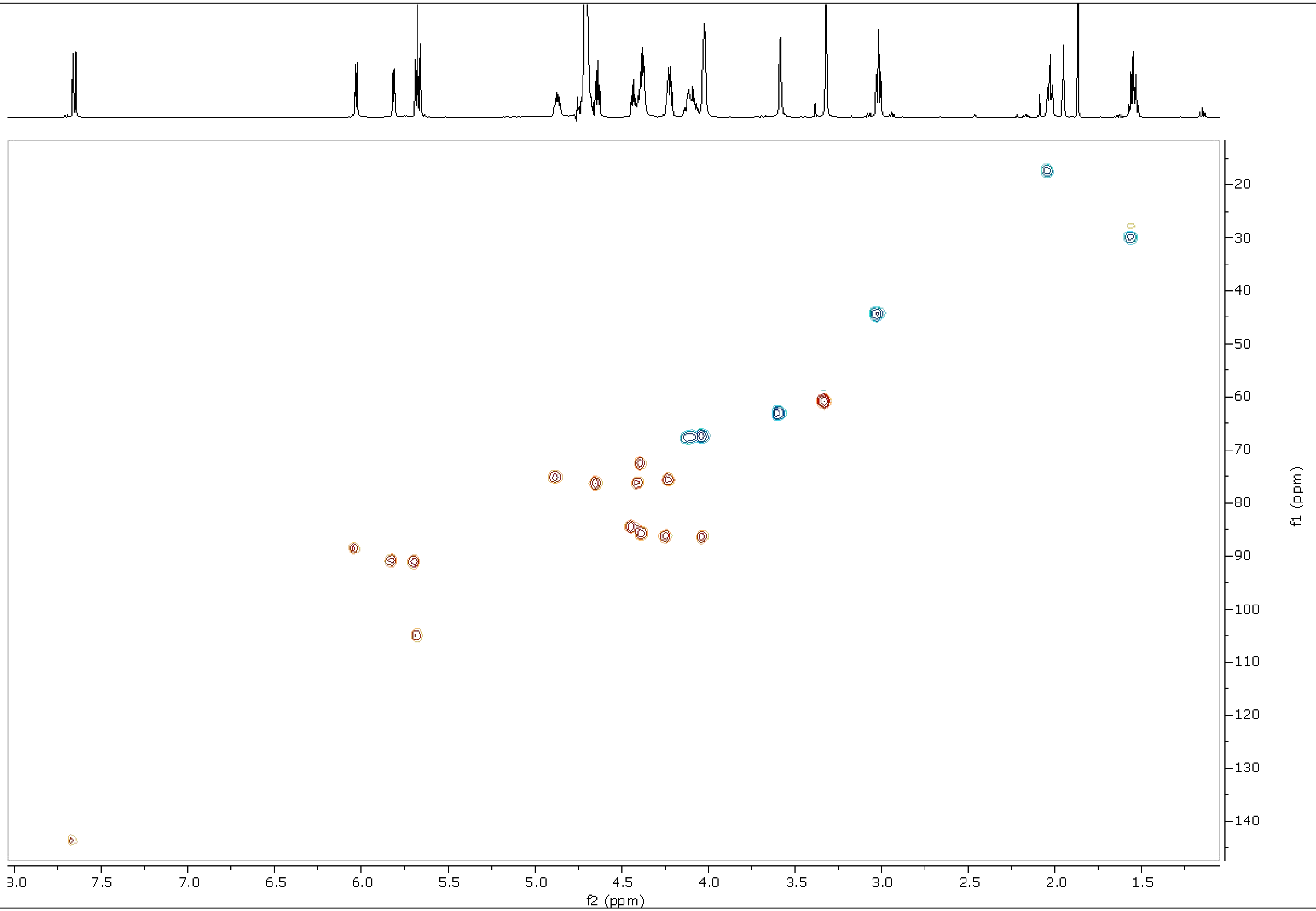

$^{1}H – ^{13}C$ HMBC NMR spectrum of **17**

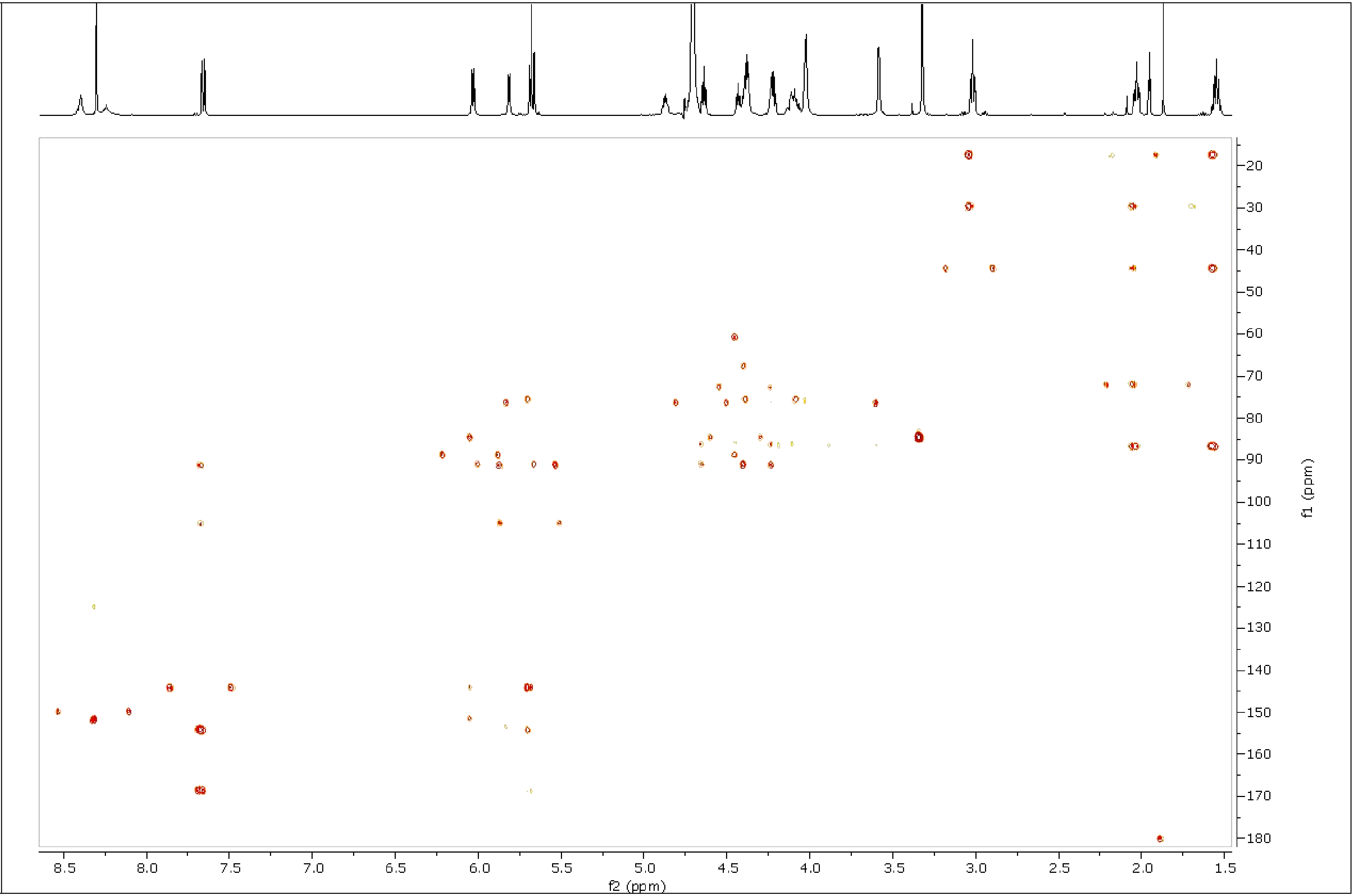

^{31}P NMR spectrum (202 MHz) of **17**

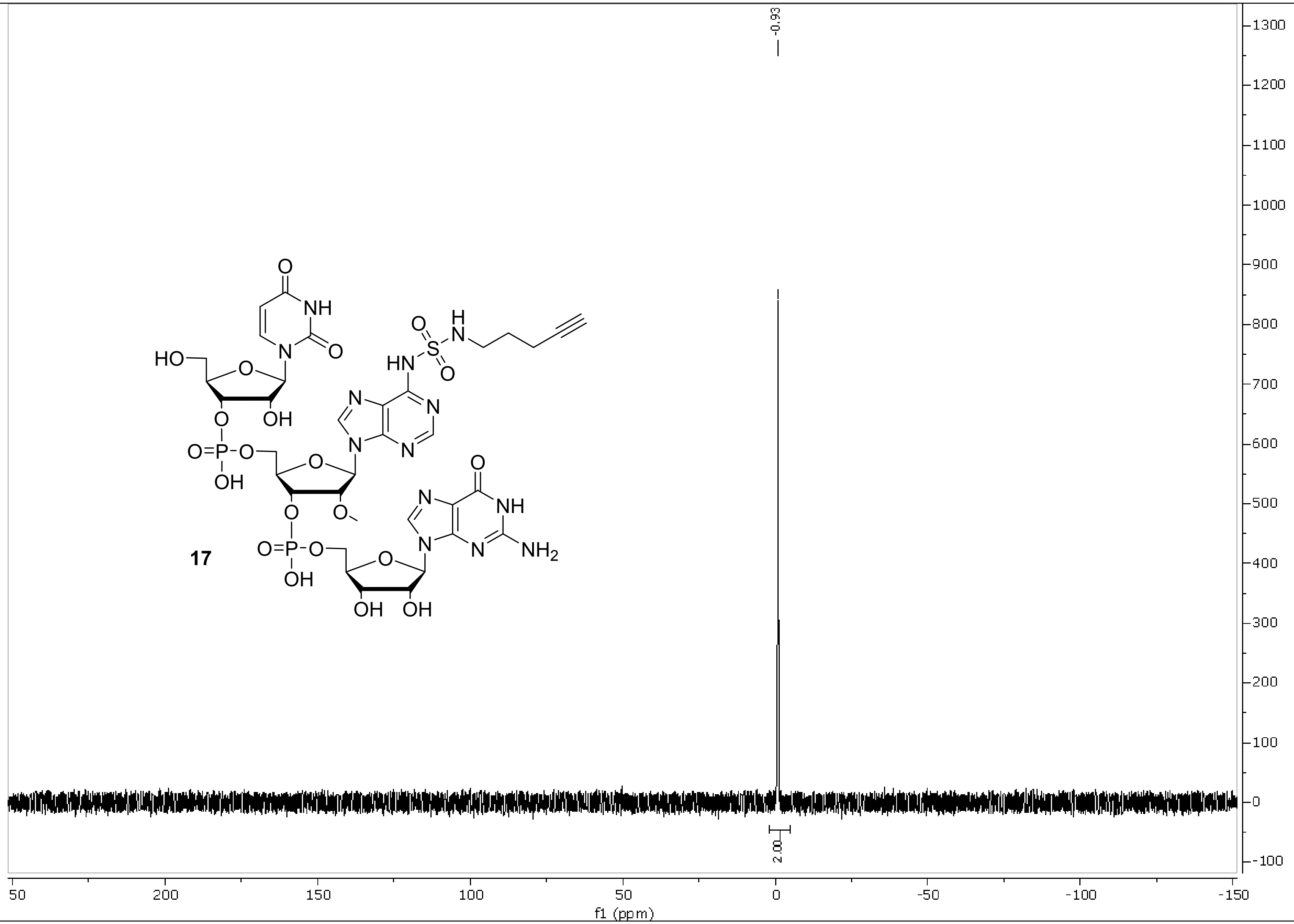

^{1}H – ^{31}P HMBC NMR spectrum of **17**

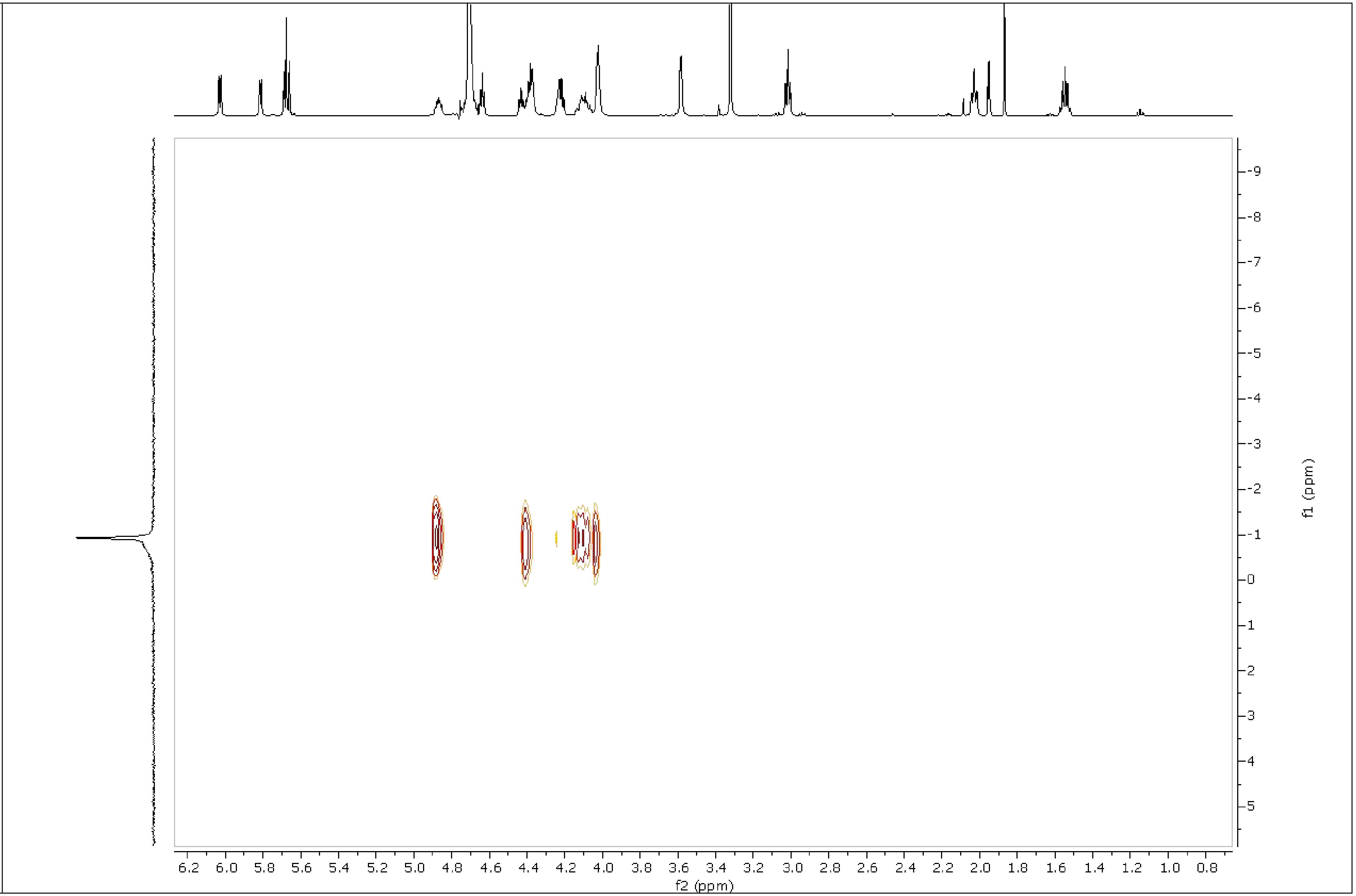

Supplement: Supplementary file 1 — ol2c02034_si_001.pdf [file ol2c02034_si_001.pdf]
